# 3-Alkynylindoles as Building Blocks for the Synthesis of Electronically Tunable Indole-Based Push–Pull Chromophores

**DOI:** 10.1021/acs.joc.2c00067

**Published:** 2022-03-01

**Authors:** Kübra Erden, Cagatay Dengiz

**Affiliations:** Department of Chemistry, Middle East Technical University, 06800 Ankara, Turkey

## Abstract

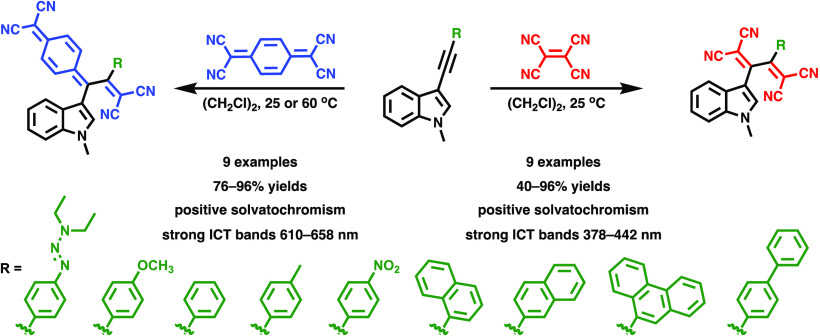

In this study, two different classes of push–pull chromophores were synthesized in modest to excellent yields by formal [2+2] cycloaddition-retroelectrocyclization (CA-RE) reactions. *N*-Methyl indole was introduced as a new donor group to activate alkynes in the CA-RE transformations. Depending on the side groups’ size and donor/acceptor characteristics, *N*-methyl indole-containing compounds exhibited λ_max_ values ranging between 378 and 658 nm. The optoelectronic properties of the reported D–A-type structures were studied by UV/vis spectroscopy and computational studies. The complete regioselectivity observed in the products was elaborated by one-dimensional (1D) and two-dimensional (2D) NMR studies, and the electron donor strength order of *N*-alkyl indole and triazene donor groups was also established. The intramolecular charge-transfer characteristics of the target push–pull chromophores were investigated by frontier orbital depictions, electrostatic potential maps, and time-dependent density functional theory calculations. Overall, the computational and experimental results match each other. Integrating a new donor group, *N*-alkyl indole, into the substrates used in formal [2+2] cycloaddition-retroelectrocyclizations has significant potential to overcome the limited donor-substituted substrate scope problem of CA-RE reactions.

## Introduction

A growing number of studies on the relationships between conjugated organic compounds and their electronic properties provide a better understanding of existing optoelectronic devices and support the logical design of ideal materials to fabricate next-generation ones.^1−4^ Considering the applications in high-technology fields such as organic solar cells (OSCs),^5,6^ organic light-emitting diodes (OLEDs),^7^ organic photodetectors,^8^ and organic sensors,^9^ the design and synthesis of easily accessible conjugated molecules is of great importance. Almost all synthetic strategies to access target conjugated structures involve multiple cross-coupling reactions requiring expensive transition-metal catalysts.^10^ There is a growing need for a synthetic approach to overcome these limitations. Short, click-type transformations are prime candidates to replace long synthetic protocols with environmentally friendly, atom/cost-economic nature.^11,12^ Azide–alkyne Huisgen cycloadditions,^13^ Diels–Alder reactions,^14^ and alkene hydrothiolations^15^ are among the most well-known and used click-type transformations in the literature. The formation of nonconjugated products in alkene hydrothiolations, explosive nature of the organic azides utilized in Huisgen cycloadditions, and high-temperature requirement encountered in a significant number of Diels–Alder reactions are still substantial issues to be resolved.^16^ Formal [2+2] cycloaddition-retroelectrocyclizations (CA-RE) have lately received prominent attention as an alternative to the current click-type methods in synthesizing conjugated molecules.^17^ Nonplanar push–pull chromophores obtained by this efficient and catalyst-free strategy draw significant attention with critical features such as intense intramolecular charge-transfer (ICT) bands, redox activity, good solubility in organic solvents, and thermal stability.^18^ Following the first report of the CA-RE between transition metal ruthenium-substituted acetylides and electron-poor alkenes by Bruce et al. in 1981,^19^ Diederich and co-workers successfully demonstrated that metal-free substrates could also participate in these transformations with their pioneer work published in 2005.^20^ Interestingly, only a few studies have been reported on metal-free substrates between 1981 and 2005.^21−23^ With the studies conducted between 2005 and 2021, CA-RE was applied to the synthesis of various push–pull targets, such as dendrimer-like structures,^24,25^ active layer material in organic solar cells,^26,27^ NLOphores,^28,29^ luminescent push–pull chromophores with fluorophore-conjugated^30−32^ and nonconjugated TCBDs,^33−36^ polymers,^17,37,38^ sensors for metal-ion detections,^39^ and Aviram-Ratner-type dyads.^40^ The most straightforward strategy to tune the optoelectronic properties of push–pull materials obtained via CA-RE is the variation of the structural designs by the appropriate choice of donor and acceptor groups.^17^ Unfortunately, CA-RE transformations suffer from a relatively limited donor-substituted substrate scope.

**Figure 1:**
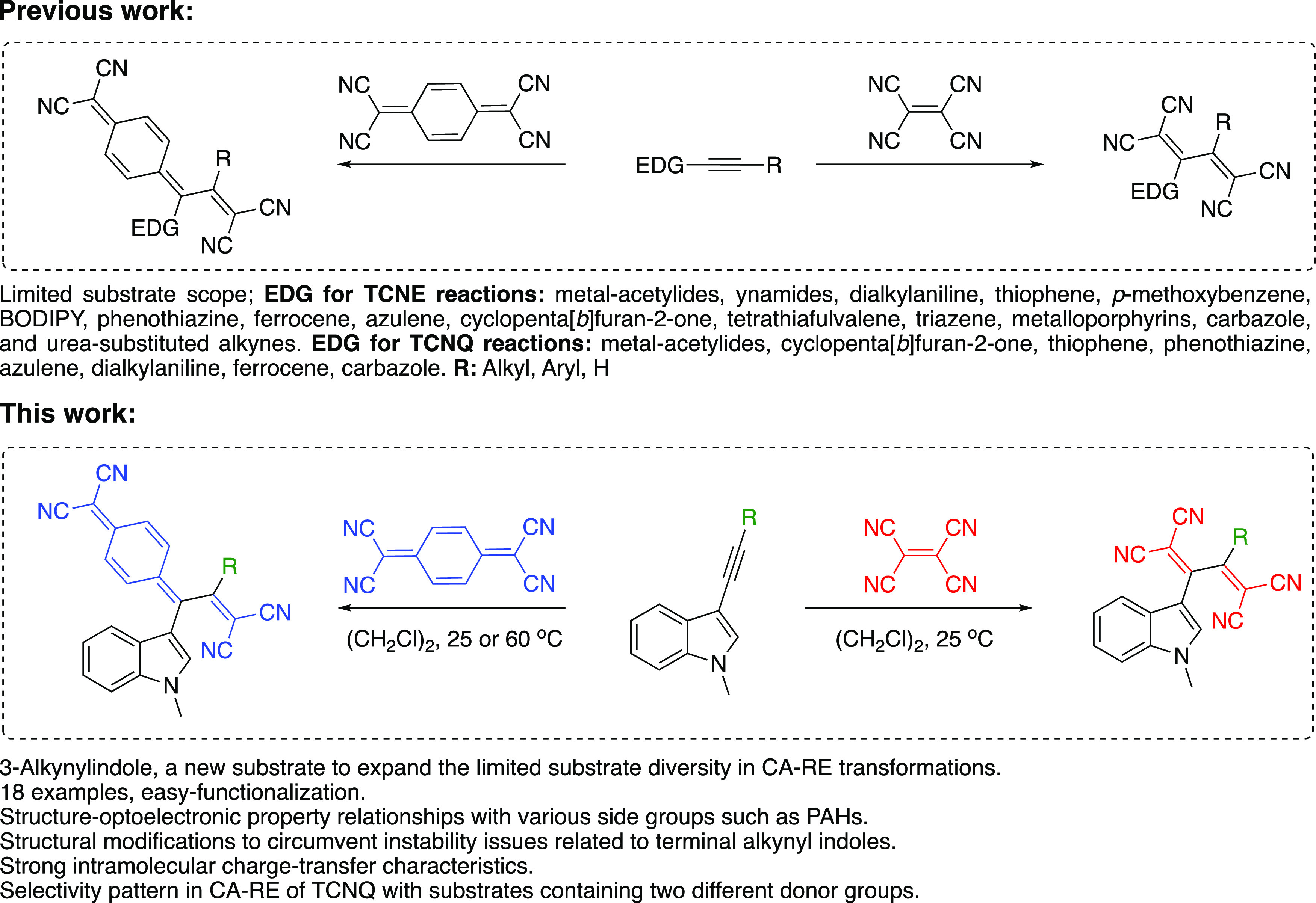
[2+2] Cycloaddition-retroelectrocyclizations of EDG-substituted alkynes with electron acceptors TCNE and TCNQ.

Electron-deficient alkenes that have recently been utilized in CA-RE chemistry can be listed as tetracyanoethylene (TCNE),^41^ 7,7′,8,8′-tetracyanoquinodimethane (TCNQ),^42^ 2,3,5,6-tetrafluoro-7,7,8,8-tetracyanoquinodimethane (F_4_-TCNQ),^18^ tetracyanoethyleneoxide (TCNEO),^43^*N*,*N*′-dicyanoquinone diimides (DCNQIs),^44^ 6,6-dicyanopentafulvenes (DCFs),^45^ and 2- (dicyanomethylene)indan-1,3-dione (DCID).^46^ Similarly, long-term storage limitations and instability reduce the diversity of donor-substituted alkynes used in CA-RE. Metal acetylides,^19,47^ dialkylaniline,^20^ ferrocene,^23^ thiophene,^48^ p-methoxybenzene,^48^ 4,4-difluoro-4-bora-3a,4a-diaza-s-indacene (BODIPY),^49^ cyclopenta[*b*]furan-2-one,^50^ metalloporphyrins,^51^ carbazole-substituted alkynes,^52^ ynamides,^53^ tetrathiafulvalene,^17^ azulene,^54^ phenothiazine,^55^ triazene,^56,57^ and ureas^33^ are donor substrates displaying sufficient reactivity in CA-RE reactions with TCNE. Surprisingly, the donor substrate scope (dialkylaniline,^42^ ferrocene,^58^ cyclopenta[*b*]furan-2-one,^59^ carbazole,^60^ metal acetylides,^61^ azulene,^62^ phenothiazine,^63^ thiophene^64^) is much more limited in CA-RE reactions with TCNQ (Figure 1). Herein, we hypothesized that *N*-alkyl indole derivatives could sufficiently activate alkynes and expand the limited substrate diversity by participating in the [2+2] CA-RE reactions. Indole donor groups offer several advantages, such as easy functionalization,^65^ potential biological,^66^ and nonlinear optical activities.^67^ Indole motifs have also continuously been investigated as classical pharmacophores.^68^ However, the use of indoles in material science is quite limited. Although indole groups are utilized as donor groups in some D–A-type push–pull systems,^69^ it was surprising that *N*-alkyl indole derivatives have never been tested in CA-RE transformations. As reported by Anderson and co-workers,^70^ terminal alkynylindoles are quite susceptible to decompositions, which could be why these species are overlooked for click-type CA-RE. Our initial assumption is that it would be possible to circumvent this limitation by increasing the molecular weight of the alkyne substrates by adding bulky substituents. Accordingly, *N*-alkyl indole-activated alkynes with various side groups, such as polyaromatic hydrocarbons and electron-rich and electron-poor phenyl groups, have been synthesized using Sonogashira cross-coupling reactions and tested for CA-RE with TCNE and TCNQ. The effects of different acceptor and side groups on the optoelectronic properties of indole-substituted push–pull chromophores were studied by density functional theory (DFT). The charge-transfer behavior of the target structures was further investigated by highest occupied molecular orbital (HOMO)/lowest unoccupied molecular orbital (LUMO) representations, electrostatic potential maps, and time-dependent density functional theory (TD-DFT) calculations.

## Results and Discussion

### Synthesis and Characterizations

**Scheme 1:**
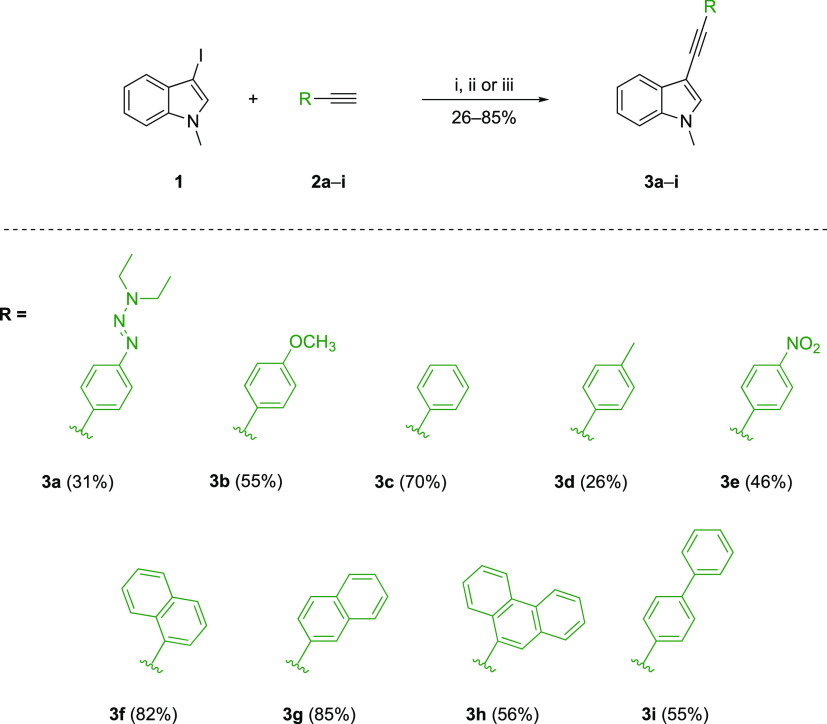
Synthesis of *N*-Methyl Indole-Activated Alkynes **3a**–**i** Reagents and conditions: (i) Pd(PPh_3_)_2_Cl_2_, CuI, Et_3_N, 25 °C for **3a**, **3b**, **3c**, **3e**. (ii) Pd(PPh_3_)_2_Cl_2_, CuI, toluene, DIPA, 25 °C, **3f**, **3g**, **3h**. (iii) Pd(PPh_3_)_2_Cl_2_, CuI, toluene, DIPA, 60 °C, **3d**, **3i**.

To bypass the reported issues with the stability of terminal alkynylindoles,^70^ the Sonogashira cross-coupling strategy of 3-iodo-1-methyl-1*H*-indole (**1**) and a variety of alkynes **2a**–**i** has been employed (Scheme 1). 3-Iodo-1-methyl-1*H*-indole (**1**) has been accessed following the two-step protocol described in the literature.^71,72^ The synthesis of **1** started from indole, which was treated with MeI for the essential protection step. Following the regioselective 3-iodination **1** was obtained in 74% yield. At the same time, alkynes substituted with electron-rich and electron-poor phenyl groups **2a**–**e**, PAHs **2f**–**i**, required for the synthesis of *N*-alkyl indole-based substrates **3a**–**i** have also been synthesized using literature procedures.^73−78^ With iodo-indole **1** and terminal alkynes **2a**–**i** in hand, the Sonogashira cross-coupling step has been performed. While preparing disubstituted alkynes **3a**, **3b**, **3c**, and **3e**, cross-coupling reactions occurred at room temperature. On the contrary, the reactions were performed in toluene in the presence of *N*,*N*-diisopropylamine (DIPA) as a base for the synthesis of **3f**, **3g**, and **3h**, presumably due to the low solubility of substrates in NEt_3_. Substrates **3d** and **3i** required slightly elevated temperatures for the completion of the reactions. All alkynes **3a**–**i** were highly stable and stored under ambient conditions without any precaution for a prolonged period. These results confirm the validity of our proposal regarding the stability problems of indole-substituted terminal alkynes.

**Scheme 2:**
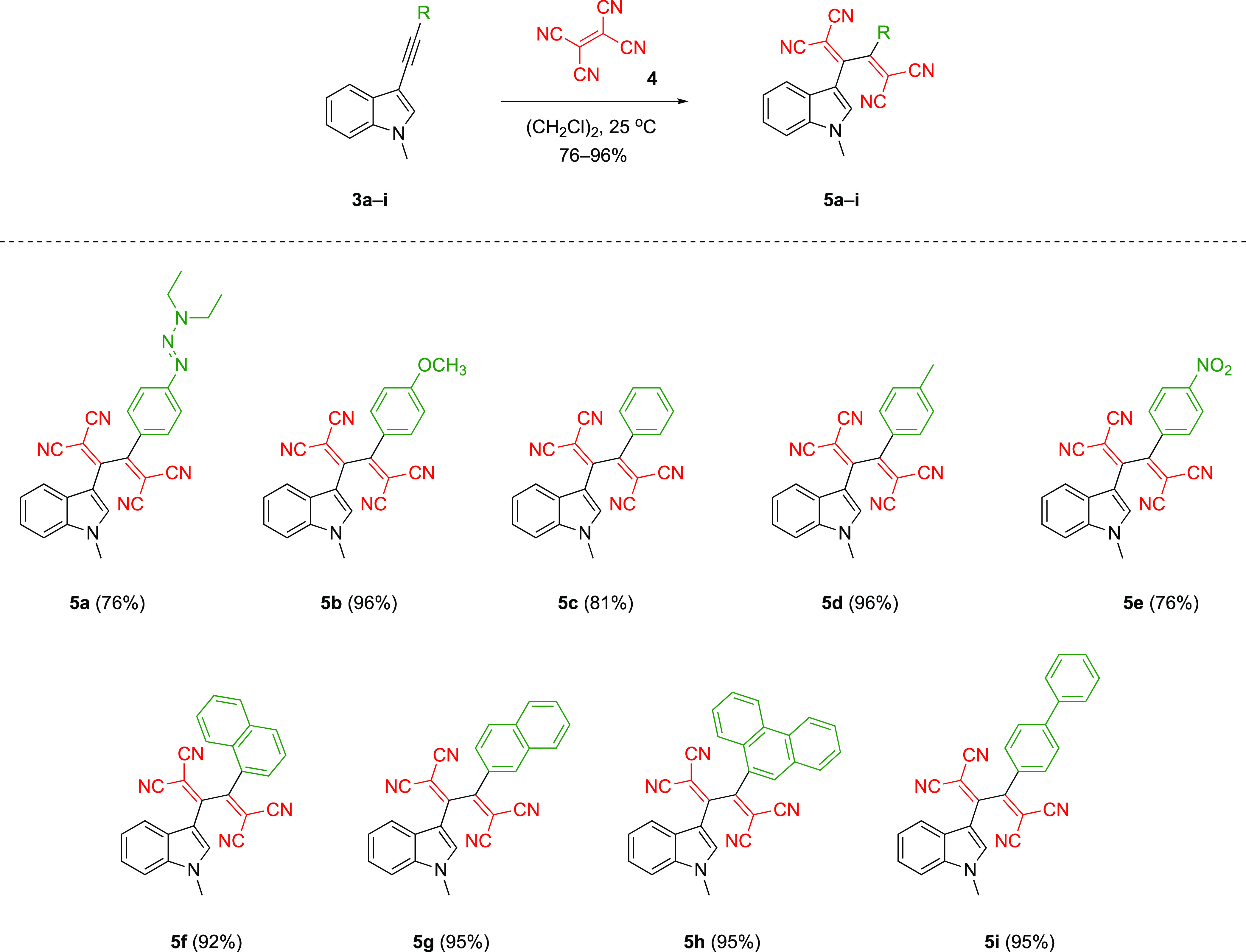
Formal [2+2] CA-RE between **3a**–**i** and TCNE **4**

After successfully preparing the stable indole-substituted alkynes **3a**–**i**, we turned our attention to whether the *N*-alkyl indole group could sufficiently activate alkynes for CA-RE transformations (Scheme 2). Initially, **3a** was tested as a substrate in CA-RE with electron-deficient TCNE **4**, and target push–pull chromophore **5a** was isolated in 76% yields. At this point, it was still unclear whether the group that activates the alkyne for the reaction was *N*-alkyl indole or the triazene,^57,73,79^ which is known to be an efficient electron donor in the literature. To confirm the electron donor role of *N*-alkyl indoles, **3c** and **3e** containing electron-withdrawing groups (phenyl and nitrophenyl) were treated with TCNE. Gratifyingly, substrates **3c** and **3e** also reacted smoothly with TCNE, allowing the synthesis of target products **5c** (81%) and **5e** (76%) respectively. This result was an indisputable proof that *N*-alkyl indole is a new type of donor group that can be exploited to activate alkynes used in CA-RE transformations. In the next stage, a systematic study was carried out where the indole donor group was kept fixed, and the side groups were altered. Regardless of the identity of the side groups, the target push–pull compounds **5a**–**i** were obtained in very high yields ranging from 76 to 96%. The relatively low yield (76%) seen in compound **5e** can be explained by the presence of the nitro group reducing the electron concentration on the alkyne unit. A similar situation observed in the case of compound **5a** is due to the difficulties encountered in isolation.

**Scheme 3:**
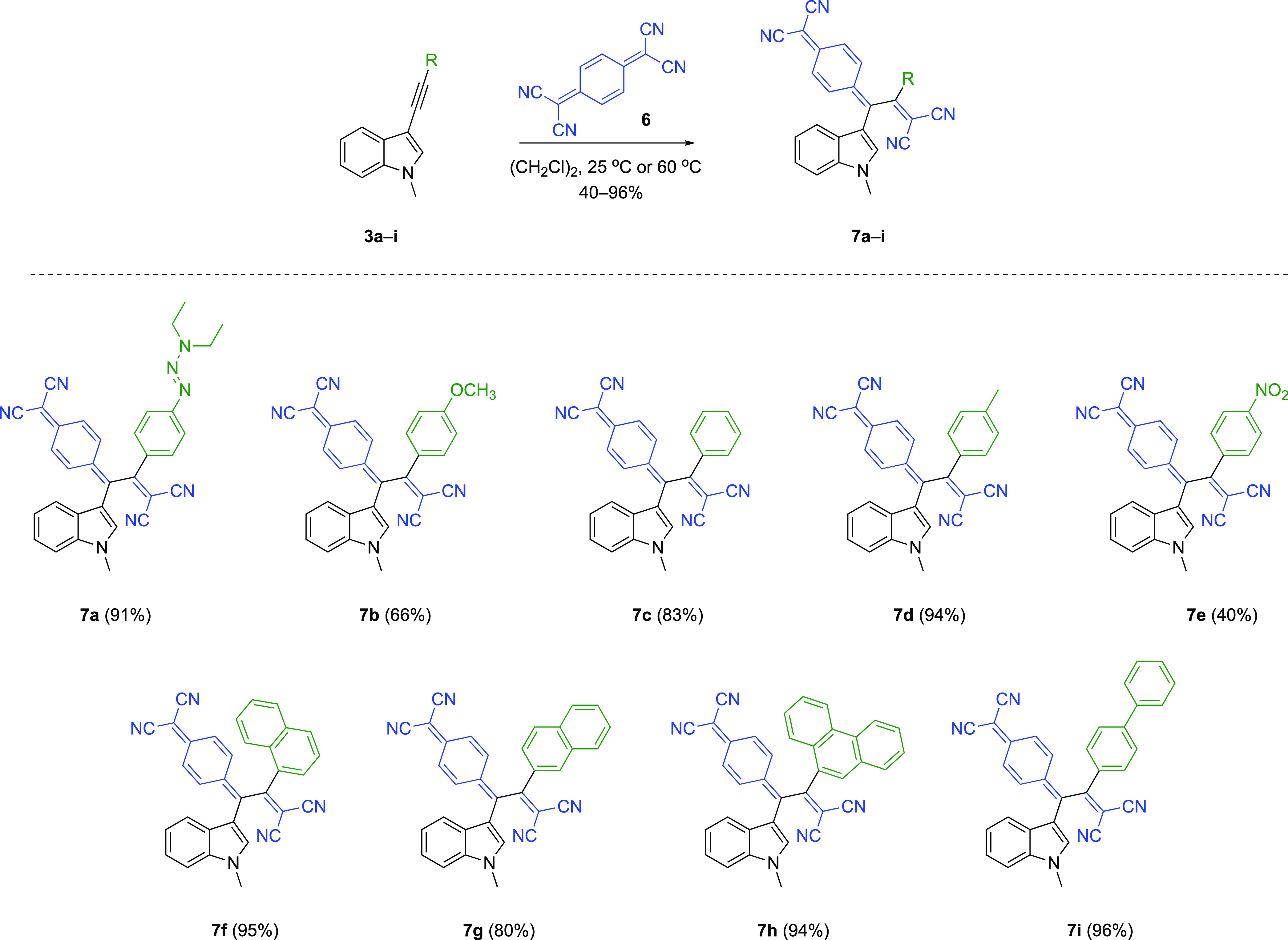
Formal [2+2] CA-RE between **3a**–**i** and TCNQ **6**

After confirming the donor behavior of *N*-alkyl indoles, substrates **3a**–**i** were also subjected to CA-RE with another well-known electron acceptor, TCNQ **6** (Scheme 3). Unlike reactions with TCNE, some substrates (**3f**, **3g**, and **3h**) reacted with TCNQ under relatively higher temperatures. The reason for this high-temperature requirement is presumably due to steric hindrance originating from bulky naphthalene and phenanthrene groups.^80^ Target push–pull compounds were obtained in moderate to excellent yields (40–96%). As previously mentioned, nitrobenzene-substituted alkyne **3e** also reacted with TCNQ in a relatively low yield (40%). The slight yield differences observed in the reactions of other substrates are related to problems encountered during the isolation step.

**Scheme 4:**
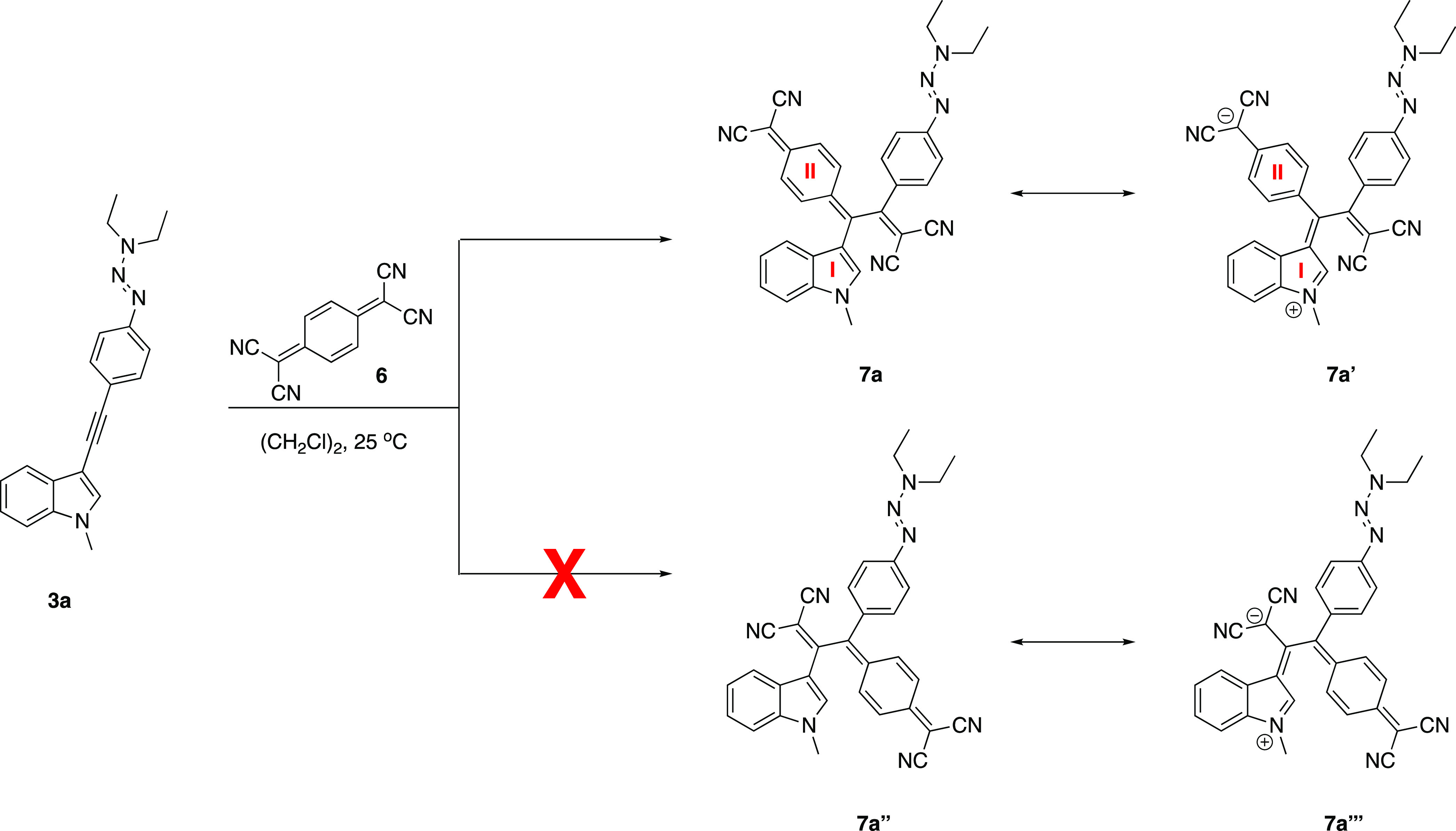
Regioselectivity in the Reaction between TCNQ **6** and Alkyne **3a**

Theoretically, two possible regioisomers **7a** and **7a**″ would be expected to be formed during the reaction of TCNQ **6** and unsymmetrical alkyne **3a**, which possess two different donor units (Scheme 4). However, complete regioselectivity was observed, and only compound **7a** was isolated as confirmed by two-dimensional (2D) HMBC (heteronuclear multiple quantum coherence) NMR spectroscopy (see Figure S38 in the Supporting Information (SI)).^81^ ICT breaks the aromaticity of indole ring (I) while generating a new one (II), as in the case of **7a**′. Therefore, the quinoidal unit prefers to be in close proximity with the strong donor as in **7a**. These results demonstrate that the *N*-alkyl indole unit is a superior electron donor compared to the triazene moiety. The reason why *N*-alkyl indoles show enhanced donor ability than triazenes can be explained by the fact that the benzene ring in triazenes (requires more energy) and the pyrrole ring in *N*-alkyl indoles (requires less energy) lose their aromaticity during intramolecular electron transfer.

### UV/vis Spectroscopy

**Figure 2:**
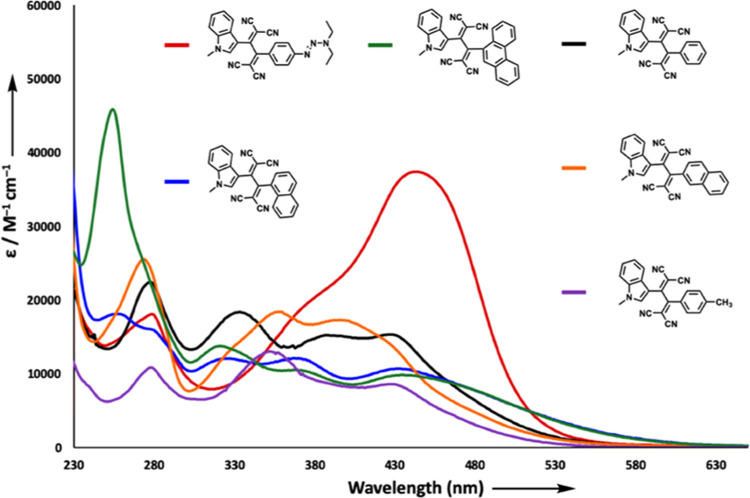
UV/vis spectra (CH_2_Cl_2_, 25 °C) of the representative chromophores **5a**, **5c**, **5d**, **5f**, **5g**, and **5h**.

All *N*-alkyl indole-substituted chromophore **5a**–**i** and **7a**–**i** solutions were intensely colored, resulting from broad intramolecular charge-transfer absorptions in the visible region of the electromagnetic spectrum. Absorption spectra for the representatives of 1,1,4,4-tetracyanobuta-1,3-diene (TCBD) derivatives **5a**, **5c**, **5d**, **5f**, **5g**, and **5h** are shown in Figure 2 (see Figure S81 in the SI for the rest of the TCBDs). TCBDs **5a**–**i** possess two distinct low-energy absorption bands λ_max,1_ = 383 nm (2.08 × 10^4^ M^–1^ cm^–1^) and λ_max,2_ = 442 nm (3.73 × 10^4^ M^–1^ cm^–1^) for **5a**; λ_max,1_ = 387 nm (1.52 × 10^4^ M^–1^ cm^–1^) and λ_max,2_ = 426 nm (1.53 × 10^4^ M^–1^ cm^–1^) for **5c**; λ_max,1_ = 352 nm (1.29 × 10^4^ M^–1^ cm^–1^) and λ_max,2_ = 427 nm (8.50 × 10^3^ M^–1^ cm^–1^) for **5d**; λ_max,1_ = 367 nm (1.20 × 10^4^ M^–1^ cm^–1^) and λ_max,2_ = 431 nm (1.06 × 10^4^ M^–1^ cm^–1^) for **5f**; λ_max,1_ = 359 nm (1.83 × 10^4^ M^–1^ cm^–1^) and λ_max,2_ = 395 nm (1.72 × 10^4^ M^–1^ cm^–1^) for **5g**; λ_max,1_ = 367 nm (1.04 × 10^4^ M^–1^ cm^–1^) and λ_max,2_ = 434 nm (9.70 × 10^3^ M^–1^ cm^–1^) for **5h**. The origin of these bands is likely due to the electron transfer from donor indole to the acceptor TCBD unit. The D–A–D-type chromophore **5a** showed the most bathochromically shifted ICT band (λ_max,2_ = 442 nm) in the TCBD series. Compounds **5f** and **5h** follow **5a** with λ_max,2_ values 431 and 434 nm, respectively. The large difference observed in the λ_max,2_ values of **5f** (32.5°, dihedral angle in between indole and dicyanovinyl units, obtained from optimized geometries, Table S19) and **5g** (27.5°) with structurally similar naphthalene substituents indicates that λ_max,2_ values are presumably more affected by sterics than electronics. Accordingly, the observed difference in the λ_max,2_ values of **5f** (32.5°) and **5h** (30.5°) compared to other chromophores **5c** (27.7°), **5d** (27.9°), and **5g** (27.5°) can be explained by considering the relatively large dihedral angles between donor and acceptor units. Although planarity is essential for the efficient overlap of π-orbitals and generally results in an increase in molar absorptivity and λ_max_ values, there are examples in the literature showing exceptional advantages of nonplanar chromophores, as in this study.^82^ These results demonstrate another advantage of nonplanar push–pull chromophores over planar counterparts, where the dihedral angle between donor and acceptor groups can be easily controlled by substituent modifications. Besides conformational control, the donor or acceptor nature of side groups can also be used to tune the absorption of the chromophores. While **5e** with nitrobenzene side group possesses CT band at around 407 nm, **5b** and **5d** with methoxy and methylbenzene groups bathochromically shifted bands at 428 and 427 nm, respectively (Figures 2 and S81 in the SI).

**Figure 3:**
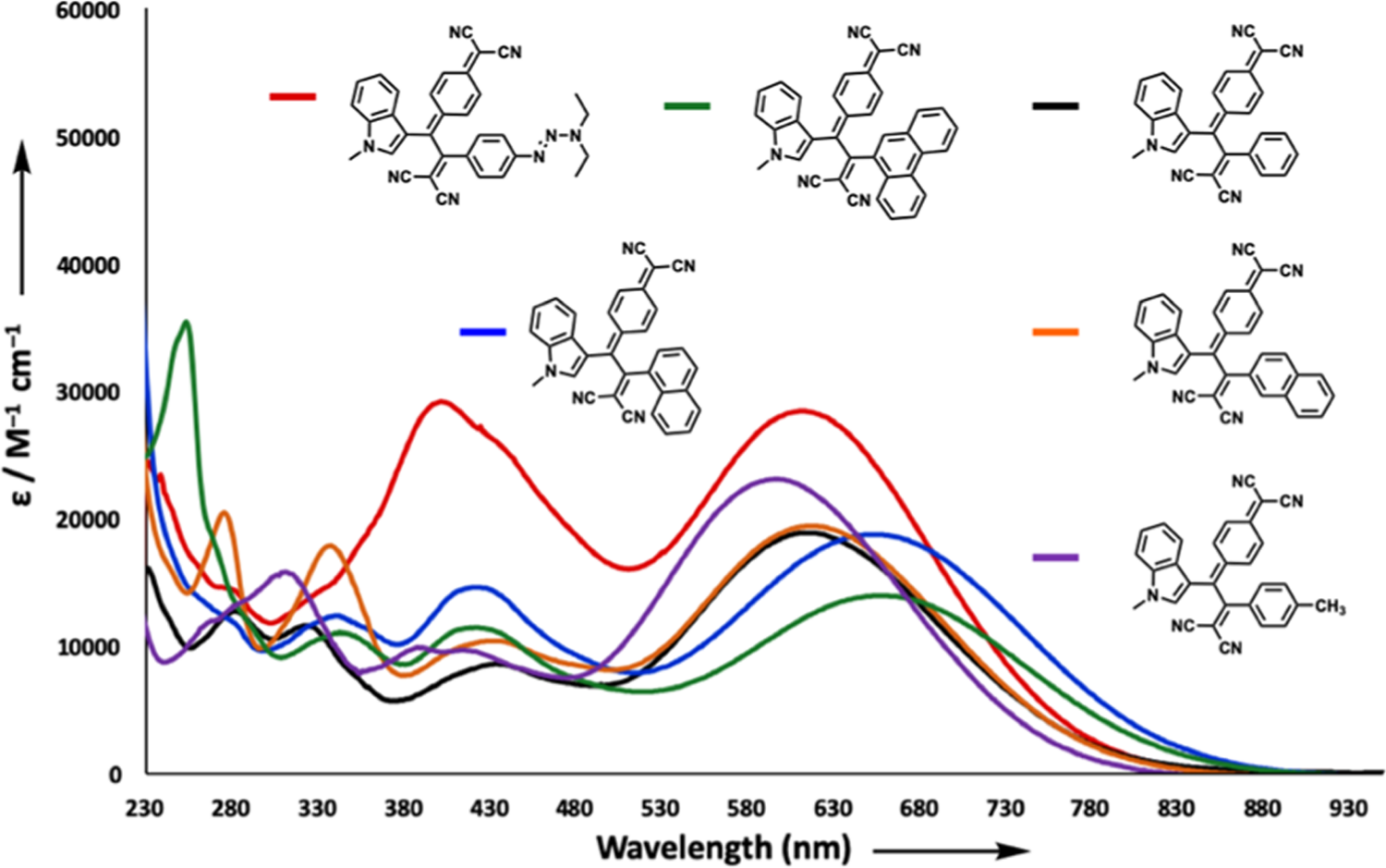
UV/vis spectra (CH_2_Cl_2_, 25 °C) of the representative chromophores **7a**, **7c**, **7d**, **7f**, **7g**, and **7h**.

TCNQ adducts **7a**–**i** showed stronger ICT absorption bands in the near-IR region with the help of extended π-conjugation in their structure compared to TCBDs **5a**–**i**. Similar to the absorption spectra of **5a**–**i**, compounds **7a**–**i** also feature two low-energy absorption bands (λ_max,1_ between 402 and 434 nm; 2.86–3.08 eV/λ_max,2_ between 612 and 658 nm; 1.88–2.03 eV). The UV/vis spectra of the selected chromophores **7a**, **7c**, **7d**, **7f**, **7g**, and **7h** are shown in Figure 3 [λ_max,1_ = 402 nm (2.91 × 10^4^ M^–1^ cm^–1^) and λ_max,2_ = 612 nm (2.84 × 10^4^ M^–1^ cm^–1^) for **7a**; λ_max,1_ = 434 nm (8.60 × 10^3^ M^–1^ cm^–1^) and λ_max,2_ = 615 nm (1.88 × 10^4^ M^–1^ cm^–1^) for **7c**; λ_max,1_ = 407 nm (9.90 × 10^3^ M^–1^ cm^–1^) and λ_max,2_ = 614 nm (2.32 × 10^4^ M^–1^ cm^–1^) for **7d**; λ_max,1_ = 423 nm (1.46 × 10^4^ M^–1^ cm^–1^) and λ_max,2_ = 653 nm (1.88 × 10^4^ M^–1^ cm^–1^) for **7f**; λ_max,1_ = 433 nm (1.05 × 10^4^ M^–1^ cm^–1^) and λ_max,2_ = 617 nm (1.95 × 10^4^ M^–1^ cm^–1^) for **7g**; λ_max,1_ = 422 nm (1.15 × 10^4^ M^–1^ cm^–1^) and λ_max,2_ = 658 nm (1.40 × 10^4^ M^–1^ cm^–1^) for **7h**]. A similar trend in λ_max_ values of TCBDs is also seen in TCNQ products **7f** (34.2°, dihedral angle in between indole and quinoidal units, obtained from optimized geometries; Table S19) and **7h** (35.4°) that possess the most red-shifted absorption bands among TCNQ products **7a** (31.0°), **7c** (31.3°),**7d** (31.4°), and **7g** (31.1°). Similar to the TCNE products, this observation can be attributed to conformational distortions caused by large dihedral angles.

**Figure 4:**
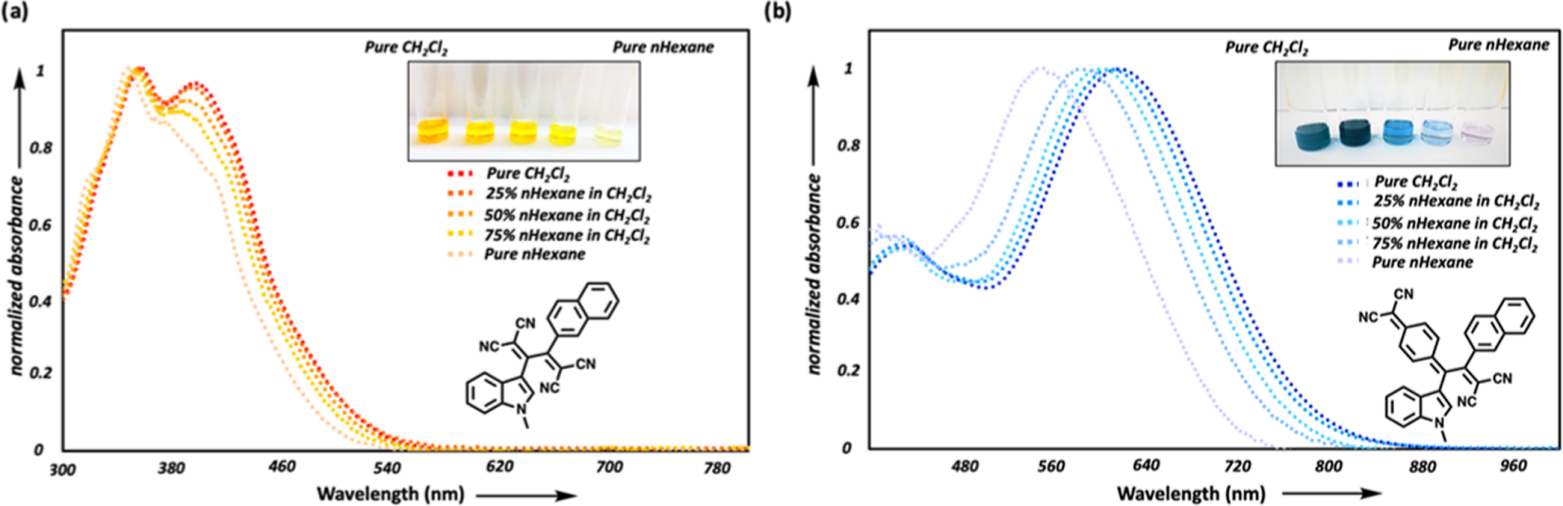
UV/vis spectra of chromophores **5g** and **7g** in CH_2_Cl_2_/*n*-hexane mixtures at 25 °C.

Both TCNE and TCNQ products show positive solvatochromism (see selected two examples **5g** and **7g** in Figure 4a,b).^83^ When the solvent is changed from polar (CH_2_Cl_2_) to nonpolar (*n*-hexane), the color of the solution of **5g** changes from dark orange to pale yellow, and the ICT band shifts from 395 nm (3.14 eV) to 389 nm (3.19 eV). On the other hand, a substantial change in ICT bands of **7g** is observed [from 617 nm (2.01 eV) to 549 nm (2.26 eV)] with the color change from turquoise to pale purple when the solvent is changed from polar (CH_2_Cl_2_) to nonpolar (*n*-hexane). The reason behind these solvatochromic behaviors of dyes **5g** and **7g** can simply be explained by the stabilization of the excited states more than the ground states by polar solvents. The deviation from planarity at different rates in different solvents should also not be ignored.

### Computational Studies

**Figure 5:**
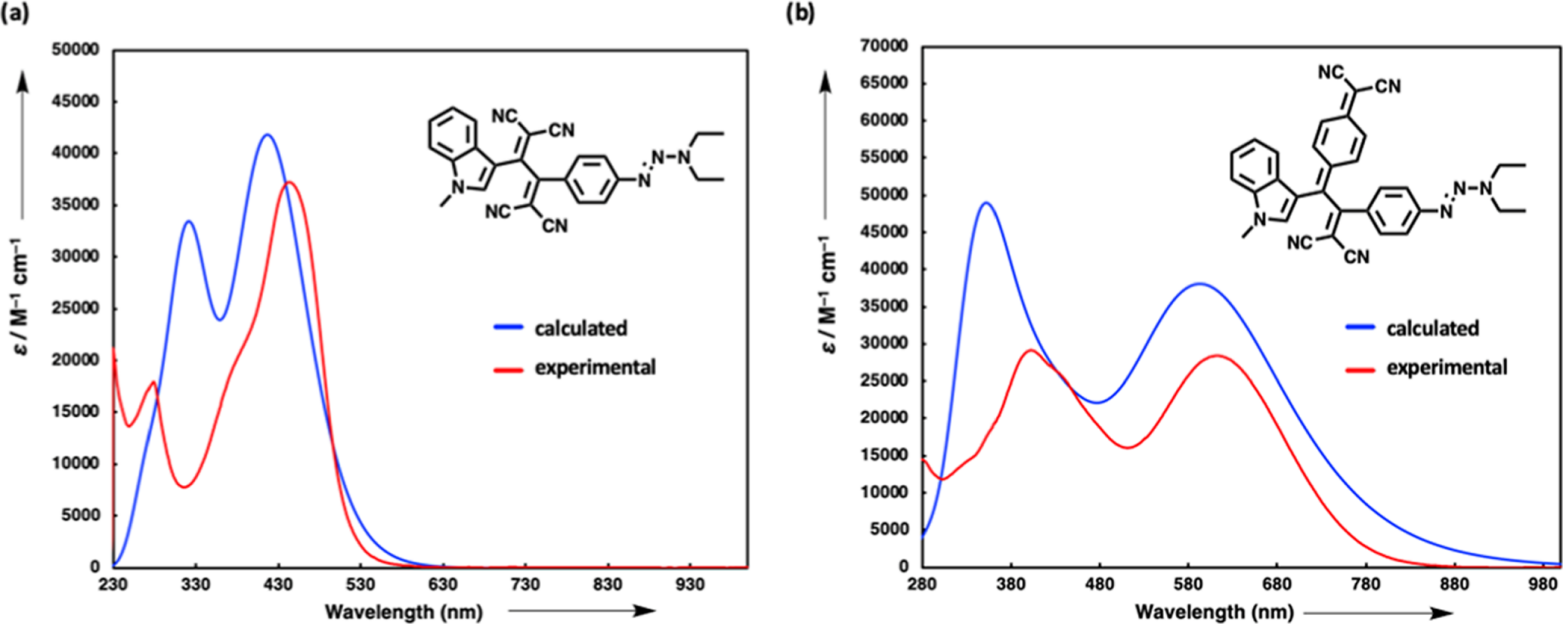
(a) Calculated (blue line) TD-DFT:CAM-B3LYP/6–31G* in CH_2_Cl_2_ and experimental UV/vis spectrum of **5a** in CH_2_Cl_2_ (red line). (b) Calculated (blue line) TD-DFT:CAM-B3LYP/6–31G* in CH_2_Cl_2_ and experimental UV/vis spectrum of **7a** in CH_2_Cl_2_ (red line).

The charge-transfer characteristics of the push–pull chromophores were further studied by time-dependent density functional theory (TD-DFT) calculations, visualizations of highest occupied molecular orbitals (HOMOs)-lowest unoccupied molecular orbitals (LUMOs), and electrostatic potential maps. Density functional theory (DFT) calculations were achieved at the B3LYP/6-31G* level of theory with CPCM solvation in CH_2_Cl_2_ using the Gaussian 09 program package.^84^ Low-energy absorption bands and their corresponding oscillator strengths (see Tables S1–S18 in the SI for all of the details) were calculated using TD-DFT at the CAM-B3LYP/6-31G* level of theory on optimized geometries at the B3LYP/6-31G* level of theory with CPCM solvation in CH_2_Cl_2_. The low-energy absorption bands (see selected examples in Figures 2 and 3) can be assigned to ICT transitions between electron-rich indole group and electron-poor cyano-rich acceptor units. In all cases, these intense bands are attributed to HOMO–LUMO transitions. Figure 5 shows both calculated and experimental UV/vis spectra of the two representative chromophores. The overall shapes of the calculated and experimental spectra of **5a** and **7a** match each other well. In both cases, calculated extinction coefficients appear to be slightly overestimated. On the other hand, the calculated λ_max_ values are somewhat lower than the experimental ones, although the results are well within the expected error range for similar chromophores.^85^

**Figure 6:**
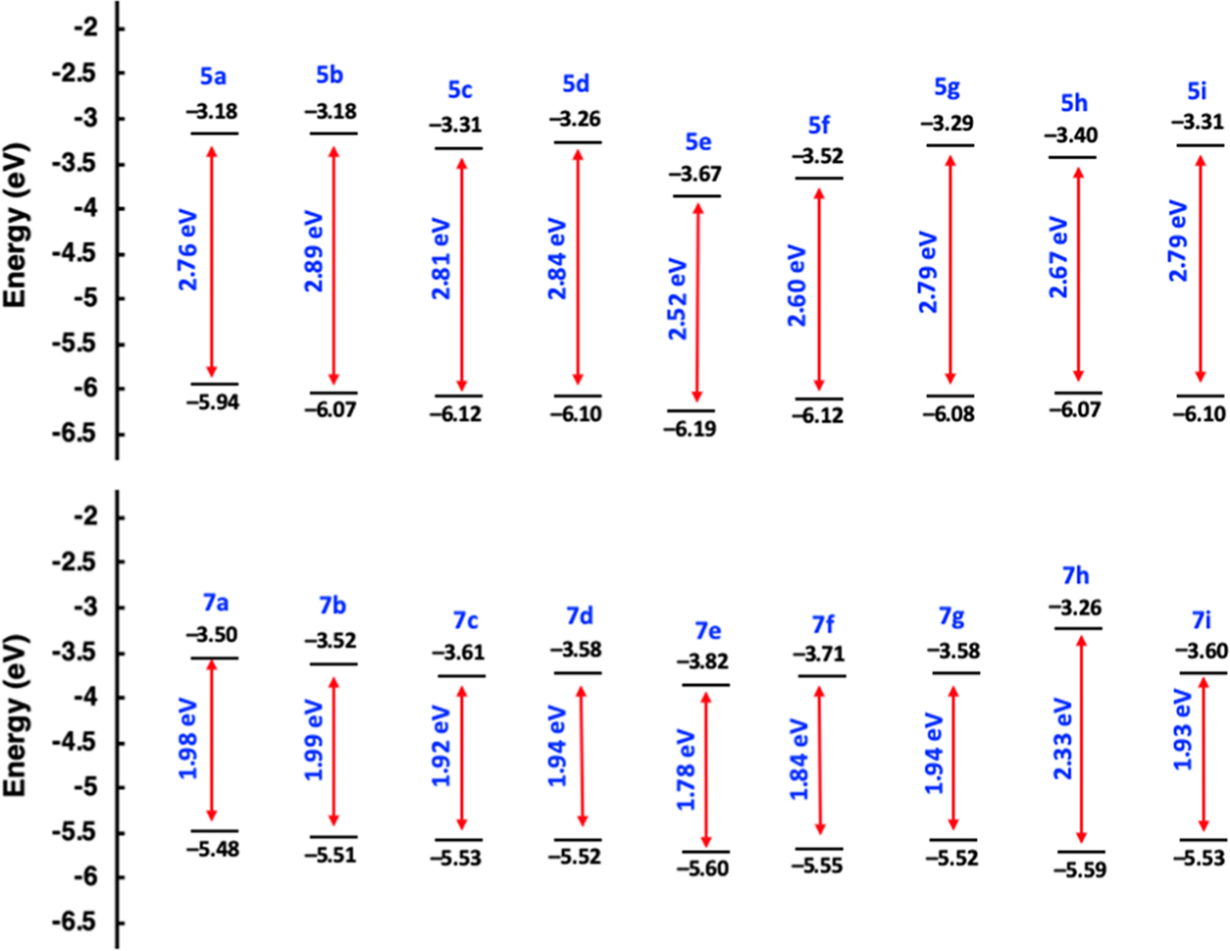
Energy-level diagram of the HOMOs and LUMOs of push–pull dyes **5a**–**i** and **7a**–**i** estimated by DFT studies.

When Figure 5a,b is examined in detail, it is noteworthy that the chromophores obtained by TCNQ have significantly red-shifted lower energy absorption bands compared to those obtained by TCNE. These results are in excellent agreement with the calculated band gap values for chromophores **5a–i** and **7a–i** (Figure 6). Calculated band gap values for TCNE products range between 2.52 and 2.89 eV, while TCNQ products have lower band gap values (1.78 and 2.33 eV) compared to **5a–i**. Both groups’ lowest band gap values were found in nitrobenzene-containing chromophores **5e** and **7e**. These results can be explained by the fact that nitrobenzene is a very powerful electron acceptor compared to other substituent groups utilized in this study.

As another proof of ICT behavior of push–pull chromophores, frontier orbital depictions of six selected compounds are given in Table 1. As mentioned earlier, the lowest-energy absorption bands mainly involve HOMO–LUMO transitions. In all cases, the electron density distribution is located on the donor indole part. On the other hand, the electron density in LUMOs is mainly concentrated on electron-poor cyano-rich regions. Both HOMO and LUMO depictions highlight small but distinct overlap describing the transfer of electrons from electron-rich indole to the electron-poor cyano-rich core. Besides frontier orbital analysis, electrostatic potential maps (ESPs) were also utilized to further discuss ICT interactions. ESP visualizations give an overall idea about the charge density and polarity of the push–pull chromophores.^86^ While red-colored regions show electronically the most negative locations, the blue-colored zones highlight positive areas. As expected, the blue areas are located on the electron-rich indole ring. In contrast, the red areas are located in the electron-poor but cyano-rich core regions, supporting the ICT behavior of push–pull chromophores.

**Table 1:**
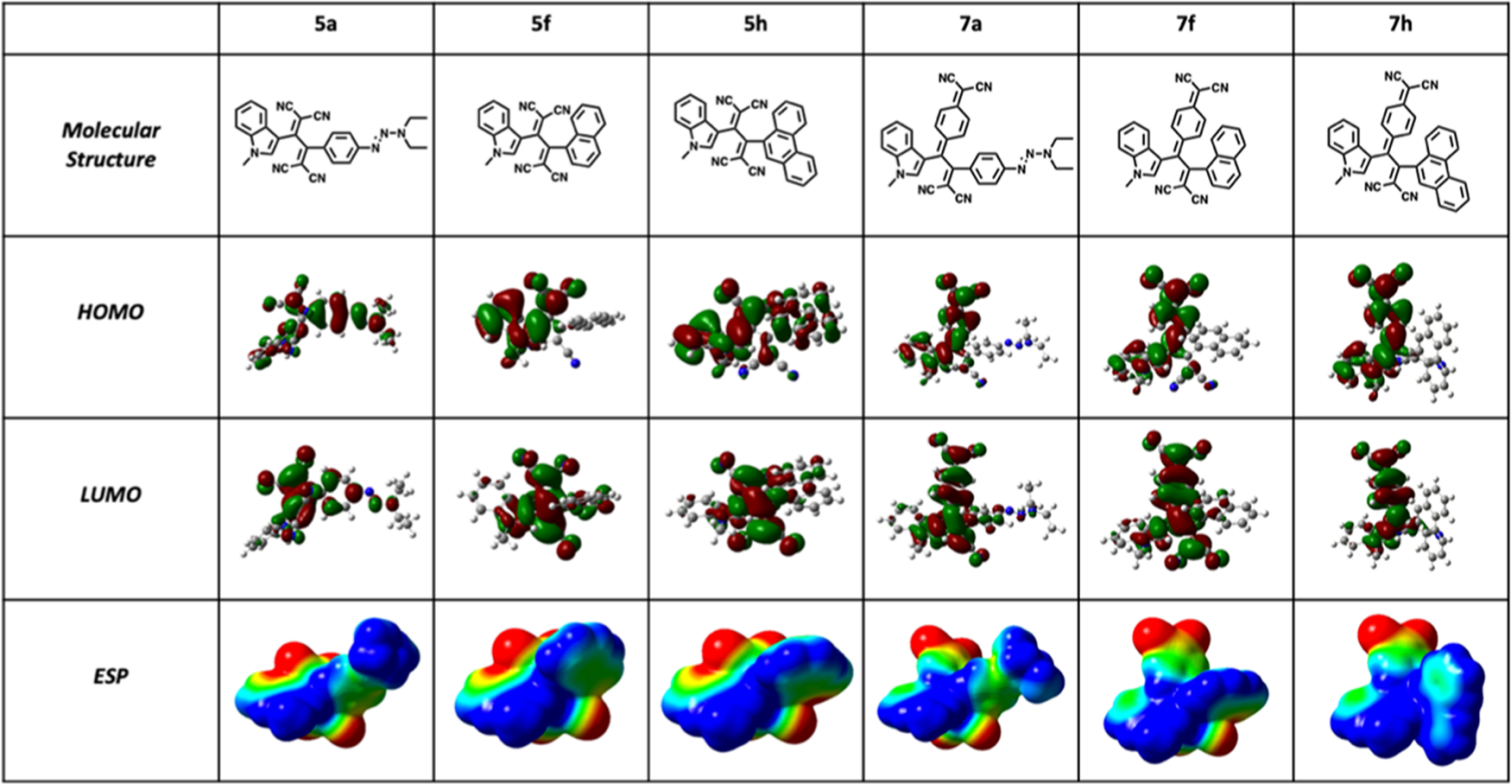
Structures, Frontier Orbital Visualizations, and Electrostatic Potential Maps [−0.03 a.u (Red) to 0.03 a.u (Blue), DFT:B3LYP/6-31G* Level of Theory] of Representative Chromophores **5a**, **5f**, **5h**, **7a**, **7f**, and **7h**^a^

a The red color represents the most negative regions. On the other hand, the blue color highlights the most positive regions.

## Conclusions

In this study, we prepared two new series of push–pull chromophores by formal [2+2] cycloaddition-retroelectrocyclizations. The electron-rich *N*-methyl indoles were utilized for the first time to activate alkynes for CA-RE transformations. With the reported synthetic approach, a significant contribution was made to improve the common limitations of the CA-RE strategy, such as limited substrate diversity and instability of substrates. Eighteen different D–A-type push–pull chromophores were isolated using two different electron-poor alkenes (TCNE and TCNQ) and nine different side groups. The wide structural diversity in this study provided insight into the structure–optical property relationships of the nonplanar push–pull chromophores. While λ_max_ values of chromophores obtained with TCNE vary between 395 nm and 442 nm, products obtained with TCNQ have λ_max_ values between 612 and 658 nm approaching the near-IR region. Additionally, it was confirmed that both groups of compounds show positive solvatochromism, a common property of push–pull-type compounds. The optical properties of the synthesized materials were also confirmed by computational methods. The ICT characteristics of the push–pull chromophores were clearly demonstrated using TD-DFT calculations, HOMO–LUMO visualizations, and ESPs. The observed differences in low-energy absorptions of TCNE and TCNQ adducts were also confirmed by calculated band gaps. In summary, all of these results show that indole-containing push–pull systems have a significant potential to find use in optoelectronic applications.

## Experimental Section

### General

Commercially available chemicals were purchased, and no additional purification has been performed. Compounds **2a**,^73^**2b**,^75^**2d**,^76^**2e**,^77^**2f**,^74^**2g**,^74^**2h**,^74^ and **2i**(78) were prepared according to literature procedures. Solvents (dichloromethane, hexanes, and ethyl acetate) used for extraction or column chromatography procedures were distilled. Cross-coupling reactions were performed under N_2_ atmosphere with oven-dried glassware. Column chromatography (CC, SiO_2_-60 mesh) was used for the purification of target compounds. Analytical thin-layer chromatography (TLC) was carried out on aluminum sheets coated with 0.2 mm silica gel 60 F254; a UV lamp (254 or 366 nm) was utilized for the visualization. Solvents were evaporated in vacuo at 25–60 °C and 900–10 mbar. ^1^H and ^13^C{^1^H} nuclear magnetic resonance (NMR) spectra were obtained at 400 MHz (^1^H) and 100 MHz (^13^C{^1^H}), respectively. Structural assignments were made with additional information from gCOSY, gHSQC, and gHMBC experiments. Chemical shifts δ are given in parts per million (ppm) downfield from tetramethylsilane using the residual deuterated solvent signal as an internal reference (CDCl_3_: δ_H_ = 7.26 ppm, δ_C_ = 77.0 ppm). For ^1^H NMR, the resonance multiplicity is described as s (singlet), d (doublet), t (triplet), q (quartet), quint (quintet), sext (sextet), sept (septet), m (multiplet), and br. (broad). Additionally, coupling constants *J* are given in hertz. All spectra were recorded at room temperature. High-resolution mass spectrometry (HRMS) was carried out by the mass spectrometry service of the Central Laboratory at Middle East Technical University, Turkey. Masses are reported in *m*/*z* units as the molecule ion as [M + H]^+^.

#### Synthesis of 3-Alkynylindoles **3a–i**

##### Synthesis of **3a**, **3b**, **3c**, and **3e:** Condition (**i**)

Iodo-indole **1** (258 mg, 1.0 mmol, 1 equiv), bis(triphenylphosphine)palladium(II) dichloride (0.030 mmol, 0.03 equiv), and copper iodide (0.030 mmol, 0.03 equiv) were added to a two-neck round-bottom flask and stirred for 30 min under inert nitrogen atmosphere. Then, triethylamine (20 mL per 1.0 mmol **1**) was added into the flask *via* a syringe and the solution was degassed for an additional 15 min with nitrogen. Terminal alkynes **2a**, **2b**, **2c**, and **2e** (1.1 mmol, 1.1 equiv) in triethylamine (8 mL per 1.0 mmol **1**) were added into the reaction medium. After stirring overnight at 25 °C, the reaction mixture was quenched with water, extracted with dichloromethane (3 × 50 mL), dried over MgSO_4_, and filtered. The solvent was removed under reduced pressure, and coupling products **3a**, **3b**, **3c**, and **3e** were isolated by performing column chromatography (CC) (SiO_2_; 9:1 *n*-hexane/ethyl acetate).

##### Compound **3a**

Yield: 102 mg; yellow amorphous solid; 31%. *R_f_* = 0.37 (SiO_2_; 9:1 *n*-hexane/ethyl acetate); m.p. = 79–81 °C. ^1^H NMR (400 MHz, CDCl_3_, 298 K): δ = 7.80–7.85 (m, 1H), 7.51 (quasi d, XX’part of AA’XX’-system, *J* = 8.9 Hz, 2H), 7.40 (quasi d, AA’part of AA’XX’-system, *J* = 8.9 Hz, 2H, 2H), 7.36–7.10 (m, 4H), 3.89–3.65 (m, 7H), 1.27–1.20 ppm (m, 6H); ^13^C{^1^H} NMR (100 MHz, CDCl_3_, 298 K): δ = 150.6, 136.4, 132.1, 129.4, 124.0, 122.8, 121.0, 120.8, 120.5, 120.43, 120.41, 109.6, 97.6, 91.7, 82.8, 47.5, 43.0, 33.2, 13.2 ppm; IR (ATR): ν̃ = 2200 (w), 1647 (w), 1595 (m), 1326 (s), 1233 (m), 740 (s) cm^–1^; HRMS (ESI) *m*/*z*: [M + H]^+^ calcd for C_21_H_23_N_4_^+^ 331.1923; found 331.1923.

##### Compound **3b**(87)

Yield: 144 mg; brown oil; 55%. *R_f_* = 0.32 (SiO_2_; 9:1 *n*-hexane/ethyl acetate); ^1^H NMR (400 MHz, CDCl_3_, 298 K): δ = 7.81 (d, *J* = 7.7 Hz, 1H), 7.49 ppm (quasi d, XX’part of AA’XX’-system*, J* = 8.5 Hz, 2H), 7.32 (m, 3H), 7.21 (t, *J* = 7.3 Hz, 1H), 6.88 (quasi d, AA’part of AA’XX’-system, *J* = 8.5 Hz, 2H), 3.84 (s, 3H), 3.81 ppm (s, 3H); ^13^C{^1^H} NMR (100 MHz, CDCl_3_, 298 K); δ = 159.2, 136.4, 132.9 (2 × C), 132.0, 129.3, 122.8, 120.4, 116.7, 114.1, 109.6, 97.5, 90.9, 81.6, 55.5, 33.2 ppm.

##### Compound **3c**(88)

Yield: 162 mg; yellow oil; 70%. *R_f_* = 0.33 (SiO_2_; 9:1 *n*-hexane/ethyl acetate); ^1^H NMR (400 MHz, CDCl_3_, 298 K): δ = 7.87 (d, *J* = 7.7 Hz, 1H), 7.61 (dd, *J* = 8.1, 1.3 Hz, 2H), 7.41–7.23 (m, 7H), 3.79 ppm (s, 3H); spectral data was consistent with the literature.^88^

##### Compound **3e**

Yield: 127 mg; yellow amorphous solid; 46%. *R_f_* = 0.28 (SiO_2_; 9:1 *n*-hexane/ethyl acetate); m.p. = 165–167 °C. ^1^H NMR (400 MHz, CDCl_3_, 298 K): δ = 8.21 (quasi d, XX’part of AA’XX’-system*, J* = 8.9 Hz, 2H); 7.81 (d, *J* = 7.6 Hz, 1H), 7.65 (quasi d, AA’part of AA’XX’-system, *J* = 8.9 Hz, 2H), 7.42 (s, 1H), 7.40–7.30 (m, 3H), 3.85 ppm (s, 3H); ^13^C{^1^H} NMR (100 MHz, CDCl_3_, 298 K); δ = 146.4, 136.5, 133.5, 131.7, 131.6, 129.1, 123.8, 123.3, 121.1, 120.2, 110.0, 96.3, 90.4, 90.1, 33.4 ppm. IR (ATR): ν̃ = 2188 (w), 1536 (s), 1506 (w), 1385 (s), 1328 (s), 1169 (m) cm^–1^; HRMS (ESI) *m*/*z*: [M + H]^+^ calcd for C_17_H_13_N_2_O_2_^+^ 277.0977; found 277.0988.

#### Synthesis of **3f, 3g, and 3h:** Condition (**ii**)

Iodo-indole **1** (258 mg, 1.0 mmol, 1 equiv), bis(triphenylphosphine)palladium(II) dichloride (0.090 mmol, 0.09 equiv), and copper iodide (0.090 mmol, 0.09 equiv) were added to a two-neck round-bottom flask and stirred for 30 min under nitrogen atmosphere. Then, toluene (6 mL per 1.0 mmol **1**) and diisopropylamine (3 mL per 1.0 mmol **1**) were added into the flask *via* a syringe and the solution was degassed for an additional 15 min with nitrogen. PAH-substituted alkynes **2f**, **2g**, and **2h** (1.75 mmol, 1.75 equiv) in toluene (6 mL per 1 mmol **1**) and diisopropylamine (3 mL per 1 mmol **1**) were added into the reaction medium. After stirring overnight at 25 °C, the reaction mixture was quenched with water, extracted with dichloromethane (3 × 50 mL), dried over MgSO_4_, and filtered. The solvent was removed under reduced pressure, and **3f**, **3g**, and **3h** were isolated by performing column chromatography (CC) (SiO_2_; 9:1 *n*-hexane/ethyl acetate).

##### Compound **3f**

Yield: 231 mg; yellow amorphous solid; 82%. *R_f_* = 0.26 (SiO_2_; 9:1 *n*-hexane/ethyl acetate); m.p. = 128–132 °C. ^1^H NMR (400 MHz, CDCl_3_, 298 K) δ = 8.56 (d, *J* = 8.3 Hz, 1H), 7.93 (d, *J* = 7.8 Hz, 1H), 7.87 (d, *J* = 8.2 Hz, 1H), 7.81 (d, *J* = 8.2 Hz, 1H), 7.78 (d, *J* = 7.1 Hz, 1H), 7.62 (t, *J* = 7.6 Hz, 1H), 7.58–7.50 (m, 1H), 7.48 (d, *J* = 7.9 Hz, 1H), 7.45 (s, 1H), 7.39 (d, *J* = 7.7 Hz, 1H), 7.35–7.27 (m, 2H), 3.86 ppm (s, 3H); ^13^C{^1^H} NMR (100 MHz, CDCl_3_, 298 K) δ = 136.5, 133.4, 133.2, 132.5, 129.7, 129.3, 128.4, 128.0, 126.7, 126.6, 126.4, 125.5, 122.9, 122.2, 120.6, 120.4, 109.8, 97.3, 89.3, 88.4, 33.2 ppm; IR (ATR): ν̃ = 2964 (w), 2197 (w), 1510 (w), 1271 (s), 742 (w) cm^–1^; HRMS (ESI) *m*/*z*: [M]^+^ calcd for C_21_H_15_N^+^ 281.1204; found 281.1212.

##### Compound **3g**

Yield: 239 mg; dark-yellow amorphous solid; 85%. *R_f_* = 0.33 (SiO_2_; 9:1 *n-*hexane/ethyl acetate); m.p. = 118–122 °C. ^1^H NMR (400 MHz, CDCl_3_, 298 K) δ = 8.06 (s, 1H), 7.88 (d, *J* = 7.8 Hz, 1H), 7.84–7.80 (m, 3H), 7.62 (dd, *J* = 8.5, 1.4 Hz, 1H), 7.52–7.46 (m, 2H), 7.39–7.35 (m, 2H), 7.34–7.29 (m, 1H), 7.28–7.23 (m, 1H), 3.84 ppm (s, 3H); ^13^C{^1^H} NMR (100 MHz, CDCl_3_, 298 K) δ = 136.3, 133.3, 132.50, 132.46, 130.6, 129.2, 128.6, 128.0, 127.8, 127.7, 126.5, 126.3, 122.8, 121.8, 120.5, 120.3, 109.7, 97.1, 91.6, 83.9, 33.1 ppm; IR (ATR): ν̃ = 2965 (w), 22202 (w), 1510 (w), 1270 (s), 818 (w) cm^–1^; HRMS (ESI) *m*/*z*: [M]^+^ calcd for C_21_H_15_N^+^ 281.1204; found 281.1204.

##### Compound **3h**

Yield: 186 mg; yellow amorphous solid; 56%. *R_f_* = 0.29 (SiO_2_; 9:1 *n-*hexane/ethyl acetate); m.p. = 146–150 °C; ^1^H NMR (400 MHz, CDCl_3_, 298 K) δ = 8.75–8.63 (m, 3H), 8.09 (s, 1H), 7.97 (d, *J* = 8.0 Hz, 1H), 7.89 (d, *J* = 7.8 Hz, 1H), 7.80–7.69 (m, 2H), 7.68–7.59 (m, 2H), 7.48 (s, 1H), 7.40 (d, *J* = 7.7 Hz, 1H), 7.36–7.28 (m, 2H), 3.87 ppm (s, 3H); ^13^C{^1^H} NMR (100 MHz, CDCl_3_, 298 K) δ = 136.5, 132.6, 131.7, 131.4, 130.9, 130.3, 130.1, 129.4, 128.5, 127.3, 127.14, 127.12, 127.08, 127.0, 122.93, 122.89, 122.7, 120.9, 120.7, 120.4, 109.8, 97.3, 89.5, 88.1, 33.3 ppm. IR (ATR): ν̃ = 3050 (w), 2195 (w), 1539 (w), 1326 (s), 875 (w) cm ^–1^; HRMS (ESI) *m*/*z*: [M]^+^ calcd for C_25_H_17_N^+^ 331.1361; found 331.1361.

#### Synthesis of **3d and3i:** Condition (**iii**)

Iodo-indole **1** (258 mg, 1.0 mmol, 1 equiv), bis(triphenylphosphine)palladium(II) dichloride (0.090 mmol, 0.09 equiv), and copper iodide (0.090 mmol, 0.09 equiv) were added to a two-neck round-bottom flask and stirred for 30 min under nitrogen atmosphere. Then, toluene (6 mL per 1.0 mmol **1**) and diisopropylamine (3 mL per 1.0 mmol **1**) were added into the flask *via* a syringe, and the solution was degassed for an additional 15 min with nitrogen. Alkynes **2d** and **2i** (1.75 mmol, 1.75 equiv) in toluene (6 mL per 1.0 mmol **1**) and diisopropylamine (3 mL per 1.0 mmol **1**) were added into the reaction medium. After stirring overnight at 60 °C in an oil bath, the reaction mixture was quenched with water, extracted with dichloromethane (3 × 50 mL), dried over MgSO_4_, and filtered. The solvent was removed under reduced pressure and **3d**, and **3i** were isolated by performing column chromatography (CC) (SiO_2_; 9:1 *n-*hexane/ethyl acetate).

##### Compound **3d**(89)

Yield: 164 mg; brown amorphous solid; 26%. *R_f_* = 0.67 (SiO_2_; 9:1 *n-*hexane/ethyl acetate); m.p. = 105–107 °C. ^1^H NMR (400 MHz, CDCl_3_, 298 K): δ = 8.66 (s, 1H), 7.76 (d, *J* = 8.2 Hz, 2H), 7.48 (d, *J* = 8.2 Hz, 1H), 7.41 (t, *J* = 7.6 Hz, 1H), 7.35 (d, *J* = 7.9 Hz, 3H), 7.29–7.26 (m, 1H), 4.00 (s, 3H), 2.45 ppm (s, 3H); ^13^C{^1^H} NMR (100 MHz, CDCl_3_, 298 K) δ = 137.6, 136.5, 132.1, 131.3, 129.4, 129.2, 122.8, 121.4, 120.5, 120.4, 109.7, 97.5, 91.2, 82.4, 33.19, 21.61 ppm; IR (ATR): ν̃ = 2917 (m), 2203 (w), 1504 (w), 1236(m) cm^–1^; HRMS (ESI) *m*/*z*: [M + H]^+^ calcd for C_18_H_16_N^+^ 246.1283; found 246.1273.

##### Compound **3i**

Yield: 169 mg; yellow amorphous solid; 55%. *R_f_* = 0.54 (SiO_2_; 9:1 *n-*hexane/ethyl acetate); m.p. = 180–182 °C. ^1^H NMR (400 MHz, CDCl_3_, 298 K): δ = 7.83 (d, *J* = 7.8 Hz, 1H), 7.64–7.57 (m, 6H), 7.45 (t, *J* = 7.4 Hz, 2H), 7.40–7.33 (m, 3H), 7.31 (d, *J* = 5.8 Hz, 1H), 7.22 (d, *J* = 7.8 Hz, 1H), 3.83 ppm (s, 3H); ^13^C{^1^H} NMR (100 MHz, CDCl_3_, 298 K) δ = 140.7, 140.3, 136.5, 132.4, 131.8, 129.4, 128.9, 127.6, 127.12, 127.10, 123.5, 122.9, 120.6, 120.4, 109.7, 97.3, 91.1, 84.1, 33.2 ppm; IR (ATR): ν̃ = 2199 (w), 1543 (w),1517(w), 1472 (w), 1385 (w), 841 (w) cm^–1^; HRMS (ESI) *m*/*z*: [M + H]^+^ calcd for C_23_H_18_N^+^ 308.1439; found 308.1432.

#### General Procedure for the Synthesis of **5a–i**

A solution of indole-substituted alkynes **3a**–**i** (1.0 mmol, 1 equiv) and TCNE (1.0 mmol, 1 equiv) in 1,2-dichloroethane (5 mL per 1.0 mmol **3a**–**i**) was stirred at 25 °C until complete consumption of starting materials based on TLC analysis (approximately 24 h). Evaporation and CC (SiO_2_; CH_2_Cl_2_) gave target products **5a**–**i**.

##### Compound **5a**

Yield: 349 mg; dark-red-orange amorphous solid; 76%. *R_f_* = 0.5 (SiO_2_; CH_2_Cl_2_); m.p. = 102–104 °C (decomposition). ^1^H NMR (400 MHz, CDCl_3_, 298 K); δ = 8.66 (s, 1H), 7.88 (quasi d, XX’part of AA’XX’-system, *J* = 8.8 Hz, 2H), 7.52 (quasi d, AA’part of AA’XX’-system, *J* = 8.8 Hz, 2H), 7.45 (d, *J* = 8.2 Hz, 1H), 7.40 (d, *J* = 7.1 Hz, 1H), 7.35 (d, *J* = 8.4 Hz, 1H), 7.24–7.20 (m, 1H), 3.99 (s, 3H), 3.82 (q, *J* = 7.2 Hz, 4H), 1.35 (t, *J* = 7.2 Hz, 3H), 1.22 ppm (t, *J* = 7.2 Hz, 3H); ^13^C{^1^H} NMR (100 MHz, CDCl_3_, 298 K); δ = 166.9, 160.9, 156.9, 138.0, 137.2, 131.7, 127.1, 125.4, 125.0, 124.6, 121.8, 120.7, 115.3, 113.3, 112.9, 112.2, 111.5, 109.9, 81.9, 74.0, 50.0, 42.3, 34.8, 14.5, 11.3 ppm; UV/vis (CH_2_Cl_2_): λ_max_ (ε) = 383 (2.08 × 10^4^), 442 nm (3.73 × 10^4^ M^–1^ cm^–1^); IR (ATR): ν̃ = 2924 (m), 2219 (m), 1595 (m), 1497 (m), 1452 (m), 1161 (m) cm^–1;^ HRMS (ESI-TOF) *m*/*z*: [M + H]^+^ calcd for C_27_H_23_N_8_^+^ 459.2046; found 459.2046.

##### Compound **5b**

Yield: 374 mg; dark-orange amorphous solid; 96%. *R_f_* = 0.36 (SiO_2_; CH_2_Cl_2_); m.p. = 179–180 °C. ^1^H NMR (400 MHz, CDCl_3_, 298 K); δ = 8.68 (s, 1H), 7.89 (quasi d, XX’part of AA’XX’-system, *J* = 9.1 Hz, 2H), 7.47 (d, *J* = 8.2 Hz, 1H), 7.40 (t, *J* = 7.6 Hz, 1H), 7.33 (d, *J* = 8.2 Hz, 1H), 7.30–7.25 (m, 1H), 7.02 (quasi d, AA’part of AA’XX’-system, *J* = 9.1 Hz, 2H), 4.00 (s, 3H), 3.91 ppm (s, 3H); ^13^C{^1^H} NMR (100 MHz, CDCl_3_, 298 K); δ = 167.0, 165.1, 160.6, 137.9, 137.4, 132.7, 125.3, 125.1, 124.7, 123.7, 120.5, 115.7, 115.2, 113.1, 112.9, 111.9, 111.6, 109.7, 82.4, 73.8, 56.0, 34.8 ppm; UV/vis (CH_2_Cl_2_): λ_max_ (ε) = 275 (1.20 × 10^4^), 428 nm (1.70 × 10^4^ M^–1^ cm^–1^); IR (ATR): ν̃ = 2917 (m), 2217 (w), 1600 (w), 1496 (w), 1453 (m), 1178 (s), 757 (w) cm^–1^; HRMS (ESI-TOF) *m*/*z*: [M + H]^+^ calcd for C_24_H_16_N_5_O^+^ 390.1355; found 390.1353.

##### Compound **5c**

Yield: 303 mg; dark-red-orange amorphous solid; 81%. *R_f_* = 0.27 (SiO_2_; CH_2_Cl_2_); m.p. = 178–182 ^ο^C; ^1^H NMR (400 MHz, CDCl_3_, 298 K); δ = 8.67 (s, 1H), 7.84 (d, *J* = 7.5 Hz, 2H), 7.66 (t, *J* = 7.5 Hz, 1H), 7.55 (t, *J* = 7.5 Hz, 2H), 7.49 (d, *J* = 8.2 Hz, 1H), 7.43 (t, *J* = 7.1 Hz, 1H), 7.38 (d, *J* = 8.1 Hz, 1H), 7.29 (t, *J* = 7.1 Hz, 1H), 4.01 ppm (s, 3H); ^13^C{^1^H} NMR (100 MHz, CDCl_3_, 298 K); δ = 168.1, 159.6, 137.7, 137.1, 134.4, 131.1, 129.8, 129.7, 125.0, 124.9, 124.5, 120.1, 114.8, 112.5, 111.9, 111.4, 110.9, 109.2, 86.6, 73.6, 34.6 ppm; UV/vis (CH_2_Cl_2_): λ_max_ (ε) = 277 (2.22 × 10^4^), 333 (1.81 × 10^4^), 387 (1.52 × 10^4^), 426 nm (1.53 × 10^4^ M^–1^ cm^–1^); IR (ATR): ν̃ = 2219 (w), 1496 (w), 1316 (s), 744 (w) cm^–1^; HRMS (ESI-TOF) *m*/*z*: [M]^+^ calcd for C_23_H_13_N_5_^+^ 359.1171; found 359.1183.

##### Compound **5d**

Yield: 388 mg; orange amorphous solid; 96%. *R_f_* = 0.62 (SiO_2_; CH_2_Cl_2_); m.p. = 235–237 °C. ^1^H NMR (400 MHz, CDCl_3_, 298 K); δ = 8.67 (s, 1H), 7.76 (quasi d, XX’part of AA’XX’-system, *J* = 7.9 Hz, 2H), 7.48 (d, *J* = 8.2 Hz, 1H), 7.41 (t, *J* = 7.1 Hz, 1H), 7.36–7.33 (m, 3H), 7.29–7.26 (m, 1H), 4.00 (s, 3H), 2.45 ppm (s, 3H); ^13^C{^1^H} NMR (100 MHz, CDCl_3_, 298 K); δ = 168.1, 160.3, 146.7, 138.0, 137.4, 130.8, 130.2, 128.8, 125.4, 125.2, 124.7, 120.5, 115.2, 112.9, 112.6, 111.7, 111.5, 109.6, 85.3, 73.9, 34.9, 22.1 ppm; UV/vis (CH_2_Cl_2_): λ_max_ (ε) = 278 (1.05 × 10^4^), 352 (1.29 × 10^4^), 427 nm (8.50 × 10^3^ M^–1^ cm^–1^); IR (ATR): ν̃ = 2916 (w), 2213 (w), 1497 (w), 1457 (m), 1400 (w) cm^–1^; HRMS (ESI-TOF) *m*/*z*: [M + H]^+^ calcd for C_24_H_16_N_5_^+^ 374.1406; found 374.1410.

##### Compound **5e**

Yield: 273 mg; yellow amorphous solid; 76%. *R_f_* = 0.46 (SiO_2_; CH_2_Cl_2_); m.p. = 207–208 °C; ^1^H NMR (400 MHz, CDCl_3_, 298 K); δ = 8.72 (s, 1H), 8.38 (quasi d, XX’ part of AA’XX’-system, *J* = 9.0 Hz, 2H), 7.96 (quasi d, AA’ part of AA’XX’-system, *J* = 9.0 Hz, 2H), 7.54 (d, *J* = 8.2 Hz, 1H), 7.47 (td, *J* = 6.3, 1.7 Hz, 1H), 7.37–7.28 (m, 2H), 4.04 ppm (s, 3H); ^13^C{^1^H} NMR (100 MHz, CDCl_3_, 298 K); δ = 166.2, 158.3, 150.6, 138.1, 137.8, 136.6, 131.2, 125.6, 125.3, 125.1, 125.0, 119.8, 114.7, 112.8, 112.2, 111.3, 110.4, 108.9, 90.9, 73.7, 35.1 ppm; UV/vis (CH_2_Cl_2_): λ_max_ (ε) = 275 (2.16 × 10^4^), 407 (1.48 × 10^4^), 482 nm (3.50 × 10^3^ M^–1^ cm^–1^); IR (ATR): ν̃ = 2916 (w), 2217 (w), 1573 (s), 1524 (w), 1498 (m), 1458 (m), 1355 (s) cm^–1^; HRMS (ESI-TOF) *m*/*z*: [M + H]^+^ calcd for C_23_H_13_N_6_O_2_^+^ 405.1100; found 405.1079.

##### Compound **5f**

Yield: 377 mg; dark-red-orange amorphous solid; 92%. *R_f_* = 0.33 (SiO_2_; CH_2_Cl_2_); m.p. = 146–150 °C; ^1^H NMR (400 MHz, CDCl_3_, 298 K); δ = 8.51 (s, 1H), 8.18 (d, *J* = 8.0 Hz, 1H), 8.08 (d, *J* = 8.0 Hz, 1H), 8.03–7.99 (m, 1H), 7.79 (d, *J* = 7.2 Hz, 1H), 7.73–7.61 (m, 2H), 7.59–7.52 (m, 3H), 7.46 (t, *J* = 7.6 Hz, 1H), 7.32 (t, *J* = 7.6 Hz, 1H), 4.03 ppm (s, 3H); ^13^C{^1^H} NMR (100 MHz, CDCl_3_, 298 K); δ = 167.2, 160.6, 138.2, 137.3, 135.7, 134.1, 131.9, 130.4, 129.9, 129.8, 128.9, 127.8, 125.4, 125.2, 125.1, 125.0, 124.6, 120.4, 115.2, 112.8, 112.2, 111.9, 111.2, 110.5, 91.3, 75.8, 34.9 ppm; UV/vis (CH_2_Cl_2_): λ_max_ (ε) = 258 (1.69 × 10^4^), 327 (1.15 × 10^4^), 367 (1.20 × 10^4^), 431 nm (1.06 × 10^4^ M^–1^ cm^–1^); IR (ATR): ν̃ = 2216 (w), 1607 (m), 1502 (w), 1357 (s), 745 (w) cm^–1^; HRMS (ESI-TOF) *m*/*z*: [M]^+^ calcd for C_27_H_15_N_5_^+^ 409.1327; found 409.1328.

##### Compound **5g**

Yield: 389 mg; dark-orange-red amorphous solid; 95%. *R_f_* = 0.52 (SiO_2_; CH_2_Cl_2_); m.p. = 140–144 °C; ^1^H NMR (400 MHz, CDCl_3_, 298 K); δ = 8.73 (s, 1H), 8.34 (s, 1H), 7.98 (d, *J* = 8.8 Hz, 1H), 7.92–7.88 (m, 3H), 7.69 (t, *J* = 7.6 Hz, 1H), 7.60 (dd, *J* = 8.0, 7.1 Hz, 1H), 7.50 (d, *J* = 8.2 Hz, 1H), 7.44–7.36 (m, 2H), 7.28–7.22 (m, 1H), 4.03 ppm (s, 3H); ^13^C{^1^H} NMR (100 MHz, CDCl_3_, 298 K); δ = 168.1, 160.1, 138.0, 137.5, 135.8, 132.61, 132.56, 130.3, 130.1, 130.0, 128.6, 128.1, 128.0, 125.2, 125.1, 124.7, 124.5, 120.4, 115.2, 113.0, 112.6, 111.7, 111.5, 109.6, 86.1, 73.7, 34.8 ppm; UV/vis (CH_2_Cl_2_): λ_max_ (ε) = 273 (2.50 × 10^4^), 359 (1.83 × 10^4^), 395 (1.72 × 10^4^ M^–1^ cm^–1^); IR (ATR): ν̃ = 2222 (w), 1506 (m), 1461 (w), 1320 (s), 747 (w) cm^–1^; HRMS (ESI-TOF) *m*/*z*: [M + H]^+^ calcd for C_27_H_16_N_5_^+^ 410.1406; found 410.1406.

##### Compound **5h**

Yield: 437 mg; dark-orange-red amorphous solid; 95%. *R_f_* = 0.43 (SiO_2_; CH_2_Cl_2_); m.p. = 238–244 °C (decomposition). ^1^H NMR (400 MHz, DMSO-*d*_6_, 298 K); δ = 9.00–8.86 (m, 2H), 8.73 (s, 1H), 8.54 (s, 1H), 8.37 (d, *J* = 6.6 Hz, 1H), 8.15 (d, *J* = 7.3 Hz, 1H), 7.89–7.74 (m, 5H), 7.65 (d, *J* = 6.2 Hz, 1H), 7.50–7.30 (m, 2H), 3.93 ppm (s, 3H); ^13^C{^1^H} NMR (100 MHz, DMSO-*d*_6_, 298 K) δ = 166.3, 159.2, 139.8, 138.1, 133.8, 131.6, 130.5, 130.4, 129.7, 129.5, 128.2, 128.0, 127.8, 127.2, 125.9, 124.4, 124.1, 124.0, 123.2, 122.9, 121.8, 114.6, 113.8, 112.7, 112.2, 111.9, 110.6, 93.8, 79.2, 77.5, 34.1 ppm; UV/vis (CH_2_Cl_2_): λ_max_ (ε) = 253 (4.55 × 10^4^), 321 (1.37 × 10^4^), 367 (1.04 × 10^4^), 434 nm (9.70 × 10^3^ M^–1^ cm^–1^); IR (ATR): ν̃ = 2202 (w), 1600 (m), 1507 (w), 1355 (s), 745 (w) cm^–1^; HRMS (ESI-TOF) *m*/*z*: [M + H]^+^ calcd for C_31_H_18_N_5_^+^ 460.1562; found 460.1562.

##### Compound **5i**

Yield: 414 mg; dark-red-orange amorphous solid; 95%. *R_f_* = 0.68 (SiO_2_; CH_2_Cl_2_); m.p. = 240–241.5 °C; ^1^H NMR (400 MHz, CDCl_3_, 298 K); δ = 8.71 (s, 1H), 7.94 (d, *J* = 8.6 Hz, 2H), 7.76 (d, *J* = 8.6 Hz, 2H), 7.62 (dd, *J* = 8.4, 1.5 Hz, 2H), 7.55–7.42 (m, 5H), 7.40 (d, *J* = 7.5 Hz, 1H), 7.30 (t, *J* = 7.1 Hz, 1H), 4.02 ppm (s, 3H); ^13^C{^1^H} NMR (100 MHz, CDCl_3_, 298 K); δ = 167.7, 160.1, 147.7, 138.8, 138.0, 137.5, 130.7, 130.0, 129.32, 129.26, 128.5, 127.4, 125.3, 125.2, 124.8, 120.5, 115.2, 112.9, 112.5, 111.7, 111.5, 109.6, 85.7, 73.8, 34.9 ppm; UV/vis (CH_2_Cl_2_): λ_max_ (ε) = 276 (2.24 × 10^4^), 378 nm (3.87 × 10^4^ M^–1^ cm^–1^); IR (ATR): ν̃ = 2917 (m), 2217 (w), 1601 (m), 1502 (w), 1456 (m), 1399 (w) cm^–1^; HRMS (ESI-TOF) *m*/*z*: [M + H]^+^ calcd for C_29_H_18_N_5_^+^ 436.1562; found 436.1558.

#### General Procedure for the Synthesis of **7a–i**

A solution of indole-substituted alkynes **3a**–**i** (1 mmol, 1 equiv) and TCNQ (1.5 mmol, 1.5 equiv) in 1,2-dichloroethane (5 mL per 1.0 mmol **3a**–**i**) was stirred at 25 °C (for **7a**–**d**, and **7i**) or 60 °C (for **7e**–**h**) in an oil bath until complete consumption of starting material based on TLC analysis (approximately 24 h). Evaporation and CC (SiO_2_; CH_2_Cl_2_) gave target products **7a**–**i**.

##### Compound **7a**

Yield: 487 mg; dark-green amorphous solid; 91%. *R_f_* = 0.30 (SiO_2_; CH_2_Cl_2_); m.p. = 149–152 °C (decomposition); ^1^H NMR (400 MHz, CDCl_3_, 298 K) δ = 7.78 (quasi d, XX’part of AA’XX’-system, *J* = 8.8 Hz, 2H), 7.68 (dd, *J* = 9.6, 1.9 Hz, 1H), 7.55 (d, *J* = 8.1 Hz, 1H), 7.46 (quasi d, AA’part of AA’XX’-system, *J* = 8.8 Hz, 2H), 7.42–7.35 (m, 3H), 7.31–7.23 (m, 2H), 7.14 (dd, *J* = 9.5, 1.9 Hz, 1H), 7.01 (dd, *J* = 9.5, 1.9 Hz, 1H), 3.91 (s, 3H), 3.80 (q, *J* = 7.1 Hz, 4H), 1.34 (t, *J* = 7.1 Hz, 3H), 1.21 ppm (t, *J* = 7.1 Hz, 3H); ^13^C{^1^H} NMR (100 MHz, CDCl_3_, 298 K) δ = 170.3, 155.8, 154.4, 146.7, 138.1, 136.4, 135.4, 133.9, 131.5, 131.3, 129.9, 126.2, 124.9, 124.54, 124.45, 123.2, 121.2, 120.2, 114.58, 114.55, 114.4, 113.7, 112.8, 110.7, 82.7, 71.9, 49.5, 41.8, 33.9, 14.1, 10.9 ppm; UV/vis (CH_2_Cl_2_): λ_max_ (ε) = 402 (2.91 × 10^4^), 612 nm (2.84 × 10^4^ M^–1^ cm^–1^); IR (ATR): ν̃ = 2200 (w), 1595 (m), 1457 (m), 1304 (s), 1506 (s) cm^–1^; HRMS (ESI-TOF) *m*/*z*: [M + H]^+^ calcd for C_33_H_27_N_8_^+^ 535.2359; found 535.2358.

##### Compound **7b**

Yield: 307 mg; dark-blue amorphous solid; 66%. *R_f_* = 0.20 (SiO_2_; CH_2_Cl_2_); m.p. = 151–153 °C; ^1^H NMR (400 MHz, CDCl_3_, 298 K); δ = 7.79 (d, *J* = 9.0 Hz, 2H), 7.67 (dd, *J* = 9.6, 1.8 Hz, 1H), 7.53 (d, *J* = 8.1 Hz, 1H), 7.45–7.37 (m, 3H), 7.29 (dd, *J* = 9.4, 1.5 Hz, 2H), 7.14 (dd, *J* = 9.6, 1.9 Hz, 1H), 7.00–6.90 (m, 3H), 3.92 (s, 3H), 3.87 ppm (s, 3H); ^13^C{^1^H} NMR (100 MHz, CDCl_3_, 298 K); δ = 170.5, 164.4, 154.6, 146.6, 138.4, 136.7, 135.7, 134.1, 132.5, 131.8, 126.8, 126.5, 125.3, 124.91, 124.86, 123.6, 120.4, 115.4, 114.8, 114.74, 114.68, 113.9, 112.9, 111.1, 83.3, 72.5, 55.9, 34.3 ppm; UV/vis (CH_2_Cl_2_): λ_max_ (ε) = 341 (2.55 × 10^4^), 409 (1.57 × 10^4^), 610 nm (3.25 × 10^4^ M^–1^ cm^–1^); IR (ATR): ν̃ = 2917 (m), 2204 (w), 1598 (m), 1507 (w), 1437 (m) cm^–1^; HRMS (ESI-TOF) *m*/*z*: [M + H]^+^ calcd for C_30_H_20_N_5_O^+^ 466.1668; found 466.1669.

##### Compound **7c**

Yield: 362 mg; dark-blue amorphous solid; 83%. *R_f_* = 0.12 (SiO_2_; CH_2_Cl_2_); m.p. = 183–187 °C; ^1^H NMR (400 MHz, CDCl_3_, 298 K); δ = 7.74 (d, *J* = 7.5 Hz, 2H), 7.66 (dd, *J* = 9.6, 1.9 Hz, 1H), 7.59–7.52 (m, 2H), 7.48 (t, *J* = 7.9 Hz, 2H), 7.44–7.37 (m, 3H), 7.32–7.26 (m, 2H), 7.15 (dd, *J* = 9.6, 1.8 Hz, 1H), 7.00 (dd, *J* = 9.6, 1.8 Hz, 1H), 3.92 ppm (s, 3H); ^13^C{^1^H} NMR (100 MHz, CDCl_3_, 298 K); δ = 171.4, 154.1, 145.4, 138.0, 136.3, 135.4, 134.3, 133.60, 133.55, 131.9, 129.6, 126.2, 125.2, 124.8, 124.6, 123.3, 120.0, 114.3, 114.2, 112.8, 111.9, 110.9, 86.8, 72.8, 33.9 ppm (25 out of 27 expected signals observed); UV/vis (CH_2_Cl_2_): λ_max_ (ε) = 283 (1.28 × 10^4^), 325 (1.17 × 10^4^), 434 (8.60 × 10^3^), 615 nm (1.88 × 10^4^ M^–1^ cm^–1^); IR (ATR): ν̃ = 2205 (w), 1603 (m) 1510 (w), 1251 (s), 756 (w) cm^–1^; HRMS (ESI-TOF) *m*/*z*: [M + H]^+^ calcd for C_29_H_18_N_5_^+^ 436.1562; found 436.1563.

##### Compound **7d**

Yield: 423 mg; dark-blue amorphous solid; 94%. *R_f_* = 0.37 (SiO_2_; CH_2_Cl_2_); m.p. = 230–232 °C; ^1^H NMR (400 MHz, CDCl_3_, 298 K); δ = 7.69–7.60 (m, 3H); 7.53 (d, *J* = 8.0 Hz, 1H), 7.43–7.37 (m, 3H), 7.31–7.26 (m, 4H), 7.14 (dd, *J* = 9.6, 1.4 Hz, 1H), 6.96 (dd, *J* = 9.5, 1.8 Hz, 1H), 3.92 (s, 3H), 2.40 ppm (s, 3H); ^13^C{^1^H} NMR (100 MHz, CDCl_3_, 298 K); δ = 171.5, 154.5, 146.1, 145.6, 138.4, 136.5, 135.7, 134.0, 132.1, 132.0, 130.7, 130.0, 126.6, 125.5, 125.0, 124.9, 123.6, 120.4, 114.71, 114.68, 114.6, 113.4, 112.5, 111.1, 85.8, 73.1 34.2, 22.0 ppm; UV/vis (CH_2_Cl_2_): λ_max_ (ε) = 328 (1.54 × 10^4^), 407 (9.90 × 10^3^), 432 (9.70 × 10^3^), 614 nm (2.32 × 10^4^ M^–1^ cm^–1^); IR (ATR): ν̃ = 2916 (m), 2200 (w), 1601 (m), 1508 (w), 1432 (m) cm^–1^; HRMS (ESI-TOF) *m*/*z*: [M + H]^+^ calcd for C_30_H_20_N_5_^+^ 450.1719; found 450.1710.

##### Compound **7e**

Yield: 192 mg; navy blue amorphous solid; 40%. *R_f_* = 0.38 (SiO_2_; CH_2_Cl_2_); m.p. = 158–159.5 °C; ^1^H NMR (400 MHz, CDCl_3_, 298 K); δ = 8.27 (quasi d, XX’ part of AA’XX’-system, *J* = 8.9 Hz, 2H), 7.85 (quasi d, AA’part of AA’XX’-system, *J* = 8.9 Hz, 2H), 7.63 (dd, *J* = 9.6, 1.9 Hz, 1H), 7.49 (d, *J* = 8.0 Hz, 1H), 7.43–7.38 (m, 3H), 7.35–7.30 (m, 2H), 7.27–7.20 (m, 1H), 7.03 (dd, *J* = 9.6, 1.9 Hz, 1H), 3.92 ppm (s, 3H); ^13^C{^1^H} NMR (100 MHz, CDCl_3_, 298 K); δ = 169.1, 154.0, 150.0, 143.4, 140.1, 138.4, 136.4, 135.5, 133.3, 133.2, 130.8, 126.31, 126.29, 126.0, 125.2, 124.8, 123.7, 119.9, 114.3, 114.2, 113.7, 112.4, 111.6, 111.4, 90.3, 74.9, 34.3 ppm; UV/vis (CH_2_Cl_2_): λ_max_ (ε) = 284 (2.33 × 10^4^), 328 (1.60 × 10^4^), 628 nm (2.40 × 10^4^ M^–1^ cm^–1^); IR (ATR): ν̃ = 2201 (w), 1600 (m), 1507 (w), 1343 (s) cm^–1^; HRMS (ESI-TOF) *m*/*z*: [M + H]^+^ calcd for C_29_H_17_N_6_O_2_^+^: 481.1413; found 481.1427.

##### Compound **7f**

Yield: 461 mg; dark-green amorphous solid; 95%. *R_f_* = 0.27 (SiO_2_; CH_2_Cl_2_); m.p. = 193–195 °C. ^1^H NMR (400 MHz, CDCl_3_, 298 K); δ = 8.05 (dd, *J* = 8.1, 2.8 Hz, 2H); 7.97–7.92 (m, 1H), 7.75 (d, *J* = 7.2 Hz, 1H), 7.65–7.51 (m, 5H), 7.44–7.36 (m, 3H), 7.30–7.27 (m, 1H), 7.20 (dd, *J* = 9.6, 1.8 Hz, 1H), 7.14 (dd, *J* = 9.6, 1.6 Hz, 1H), 7.04 (dd, *J* = 9.6, 1.6 Hz, 1H), 3.90 ppm (s, 3H); ^13^C{^1^H} NMR (100 MHz, CDCl_3_, 298 K) δ = 171.0, 154.0, 146.6, 138.2, 137.0, 136.5, 134.4, 134.2, 134.1, 133.9, 133.7, 130.7, 129.9, 129.8, 128.7, 127.4, 127.0, 125.7, 125.2, 124.9, 124.0, 123.4, 120.2, 115.6, 114.6, 114.5, 113.3, 112.2, 111.2, 91.6, 73.7, 34.2 ppm. (32 out of 33 signals expected); UV/vis (CH_2_Cl_2_): λ_max_ (ε) = 343 (1.25 × 10^4^), 423 (1.46 × 10^4^), 653 nm (1.88 × 10^4^ M^–1^ cm^–1^); IR (ATR): ν̃ = 2228 (w), 1600 (m), 1504 (w), 1332 (s), 754 (w) cm^–1^; HRMS (ESI-TOF) *m*/*z*: [M]^+^ calcd for C_33_H_19_N_5_^+^ 485.1640; found 485.1639.

##### Compound **7g**

Yield: 388 mg; dark-turquoise amorphous solid; 80%. *R_f_* = 0.38 (SiO_2_; CH_2_Cl_2_); m.p. = 175–179 °C; ^1^H NMR (400 MHz, CDCl_3_, 298 K); δ = 8.26 (s, 1H), 7.91 (d, *J* = 8.7 Hz, 1H), 7.86 (d, *J* = 8.7 Hz, 2H), 7.79 (dd, *J* = 8.7, 1.7 Hz, 1H), 7.71 (dd, *J* = 9.6, 1.7 Hz, 1H), 7.64 (t, *J* = 6.9 Hz, 1H), 7.62–7.54(m, 2H), 7.45 (s, 1H), 7.43–7.35 (m, 2H), 7.32–7.25 (m, 2H), 7.14 (dd, *J* = 9.5, 1.7 Hz, 1H), 7.05 (dd, *J* = 9.5, 1.7 Hz, 1H), 3.91 ppm (s, 3H); ^13^C{^1^H} NMR (100 MHz, CDCl_3_, 298 K); δ = 171.6, 154.4, 146.0, 138.4, 136.7, 135.7, 135.4, 134.0, 132.7, 132.3, 132.03, 131.96, 130.0, 129.9, 129.8, 128.1, 127.9, 126.5, 125.5, 125.1, 124.9, 124.8, 123.6, 120.3, 114.7, 114.6, 113.5, 112.5, 111.2, 86.7, 72.9, 34.3 ppm (32 out of 33 signals expected); UV/vis (CH_2_Cl_2_): λ_max_ (ε) = 276 (2.00 × 10^4^), 338 (1.80 × 10^4^), 433 (1.05 × 10^4^), 617 nm (1.95 × 10^4^ M^–1^ cm^–1^); IR (ATR): ν̃ = 2202 (w), 1600 (m), 1430 (w), 1337 (s), 740 (w) cm^–1^; HRMS (ESI-TOF) *m*/*z*: [M + H]^+^ calcd for C_33_H_20_N_5_^+^ 486.1719; found 486.1719.

##### Compound **7h**

Yield: 504 mg; dark-green amorphous solid; 94%. *R_f_* = 0.26 (SiO_2_; CH_2_Cl_2_); m.p. = 234–238 °C; ^1^H NMR (400 MHz, CDCl_3_, 298 K); δ = 8.76 (d, *J* = 8.4 Hz, 1H), 8.68 (d, *J* = 8.4 Hz, 1H), 8.13 (d, *J* = 8.2 Hz, 1H), 8.02 (s, 1H), 7.92 (d, *J* = 7.9 Hz, 1H), 7.80 (t, *J* = 7.7 Hz, 1H), 7.75 (t, *J* = 7.7 Hz, 1H), 7.71–7.63 (m, 2H), 7.61 (d, *J* = 8.0 Hz, 1H), 7.54 (dd, *J* = 9.5, 1.7 Hz, 1H), 7.42–7.34 (m, 3H), 7.31–7.24 (m, 2H), 7.22 (dd, *J* = 9.5, 1.6 Hz, 1H), 7.08–7.04 (m, 1H), 3.88 ppm (s, 3H); ^13^C{^1^H} NMR (100 MHz, CDCl_3_, 298 K) δ = 170.9, 153.9, 146.4, 138.2, 136.8, 136.5, 134.8, 133.7, 133.23, 133.18, 132.2, 131.1, 130.4, 130.2, 129.9, 128.3, 128.0, 127.94, 127.85, 127.1, 125.8, 125.4, 125.1, 124.9, 124.2, 123.5, 123.0, 120.1, 115.6, 114.5, 114.4, 113.3, 112.3, 111.2, 91.7, 74.1, 34.2 ppm; UV/vis (CH_2_Cl_2_): λ_max_ (ε) = 345 (1.11 × 10^4^), 422 (1.15 × 10^4^), 658 nm (1.40 × 10^4^ M^–1^ cm^–1^); IR (ATR): ν̃ = 2203 (w), 1598 (m), 1505 (w), 1345 (s), 738 (w) cm^–1^; HRMS (ESI-TOF) *m*/*z*: [M + H]^+^ calcd for C_37_H_22_N_5_^+^ 536.1875; found 536.1874.

##### Compound **7i**

Yield: 491 mg; dark-blue amorphous solid; 96%. *R_f_* = 0.55 (SiO_2_; CH_2_Cl_2_); m.p. = 220–221 °C; ^1^H NMR (400 MHz, CDCl_3_, 298 K); δ = 7.85 (d, *J* = 8.6 Hz, 2H); 7.71–7.66 (m, 3H), 7.60–7.56 (m, 3H), 7.48–7.38 (m, 6H), 7.33–7.27 (m, 2H), 7.18 (dd, *J* = 9.6, 1.9 Hz, 1H), 7.02 (dd, *J* = 9.6, 1.9 Hz, 1H), 3.93 ppm (s, 3H); ^13^C{^1^H} NMR (100 MHz, CDCl_3_, 298 K); δ = 171.0, 154.5, 150.9, 146.8, 145.9, 138.8, 138.4, 136.7, 135.7, 133.9, 133.2, 132.1, 131.3, 130.6, 129.3, 129.1, 128.3, 127.3, 126.5, 125.5, 125.1, 125.0, 123.6, 120.4, 114.6, 113.4, 112.5, 111.7, 111.2, 86.1, 72.9, 34.3 ppm; UV/vis (CH_2_Cl_2_): λ_max_ (ε) = 340 (2.00 × 10^4^), 402 (2.10 × 10^4^), 618 nm (2.49 × 10^4^ M^–1^ cm^–1^); IR (ATR): ν̃ = 2918 (m), 2198 (w), 1600 (m), 1508 (w), 1456 (m) cm^–1^; HRMS (ESI-TOF) *m*/*z*: [M + H]^+^ calcd for C_35_H_22_N_5_^+^ 512.1875; found 512.1883.

## Supporting Information Available

Theoretical calculations, HRMS data, and copies of UV/vis, ^1^H NMR, and ^13^C{^1^H} NMR spectra (PDF)

## References

[ref1] Gompper R.; Wagner H. Donor-Acceptor-Substituted Cyclic π-Electron Systems—Probes for Theories and Building Blocks for New Materials. Angew. Chem., Int. Ed. 1988, 27, 1437–1455. 10.1002/anie.198814371.

[ref2] Forrest S. R.; Thompson M. E. Introduction: Organic Electronics and Optoelectronics. Chem. Rev. 2007, 107, 923–925. 10.1021/cr0501590.

[ref3] Roncali J.; Leriche P.; Cravino A. From One- to Three-Dimensional Organic Semiconductors: In Search of the Organic Silicon?. Adv. Mater. 2007, 19, 2045–2060. 10.1002/adma.200700135.

[ref4] Kivala M.; Diederich F. Acetylene-Derived Strong Organic Acceptors for Planar and Nonplanar Push-Pull Chromophores. Acc. Chem. Res. 2009, 42, 235–248. 10.1021/ar8001238. 19061332

[ref5] Guo X.; Baumgarten M.; Müllen K. Designing π-Conjugated Polymers for Organic Electronics. Prog. Polym. Sci. 2013, 38, 1832–1908. 10.1016/j.progpolymsci.2013.09.005.

[ref6] Zhang G.; Zhao J.; Chow P. C. Y.; Jiang K.; Zhang J.; Zhu Z.; Zhang J.; Huang F.; Yan H. Nonfullerene Acceptor Molecules for Bulk Heterojunction Organic Solar Cells. Chem. Rev. 2018, 118, 3447–3507. 10.1021/acs.chemrev.7b00535. 29557657

[ref7] Ostroverkhova O. Organic Optoelectronic Materials: Mechanisms and Applications. Chem. Rev. 2016, 116, 13279–13412. 10.1021/acs.chemrev.6b00127. 27723323

[ref8] Dou L.; Liu Y.; Hong Z.; Li G.; Yang Y. Low-Bandgap Near-IR Conjugated Polymers/Molecules for Organic Electronics. Chem. Rev. 2015, 115, 12633–12665. 10.1021/acs.chemrev.5b00165. 26287387

[ref9] Rahman M. A.; Kumar P.; Park D. S.; Shim Y. B. Electrochemical Sensors Based on Organic Conjugated Polymers. Sensors 2008, 8, 118–141. 10.3390/s8010118. 27879698 PMC3681146

[ref10] Egorova K. S.; Ananikov V. P. Toxicity of Metal Compounds: Knowledge and Myths. Organometallics 2017, 36, 4071–4090. 10.1021/acs.organomet.7b00605.

[ref11] Moses J. E.; Moorhouse A. D. The Growing Applications of Click Chemistry. Chem. Soc. Rev. 2007, 36, 1249–1262. 10.1039/b613014n. 17619685

[ref12] Kolb H. C.; Finn M. G.; Sharpless K. B. Click Chemistry: Diverse Chemical Function from a Few Good Reactions. Angew. Chem., Int. Ed. 2001, 40, 2004–2021. 10.1002/1521-3773(20010601)40:11<2004::AID-ANIE2004>3.0.CO;2-5. 11433435

[ref13] Rostovtsev V. V.; Green L. G.; Fokin V. V.; Sharpless K. B. A Stepwise Huisgen Cycloaddition Process: Copper(I)-Catalyzed Regioselective “Ligation” of Azides and Terminal Alkynes. Angew. Chem., Int. Ed. 2002, 41, 2596–2599. 10.1002/1521-3773(20020715)41:14<2596::AID-ANIE2596>3.0.CO;2-4. 12203546

[ref14] Nicolaou K. C.; Snyder S. A.; Montagnon T.; Vassilikogiannakis G. The Diels-Alder Reaction in Total Synthesis. Angew. Chem., Int. Ed. 2002, 41, 1668–1698. 10.1002/1521-3773(20020517)41:10<1668::AID-ANIE1668>3.0.CO;2-Z. 19750686

[ref15] Lowe A. B. Thiol-Ene “Click” Reactions and Recent Applications in Polymer and Materials Synthesis. Polym. Chem. 2010, 1, 17–36. 10.1039/b9py00216b.

[ref16] Uygun M.; Tasdelen M. A.; Yagci Y. Influence of Type of Initiation on Thiol-Ene “Click” Chemistry. Macromol. Chem. Phys. 2010, 211, 103–110. 10.1002/macp.200900442.

[ref17] Michinobu T.; Diederich F. The [2+2] Cycloaddition-Retroelectrocyclization (CA-RE) Click Reaction: Facile Access to Molecular and Polymeric Push-Pull Chromophores. Angew. Chem., Int. Ed. 2018, 57, 3552–3577. 10.1002/anie.201711605. 29469183

[ref18] Kato S. I.; Diederich F. Non-Planar Push-Pull Chromophores. Chem. Commun. 2010, 46, 1994–2006. 10.1039/b926601a. 20221473

[ref19] Bruce M. I.; Rodgers J. R.; Snow M. R.; Swincer A. G. Cyclopentadienyl-Ruthenium and-Osmium Chemistry. Cleavage of Tetracyanoethylene under Mild Conditions: X-Ray Crystal Structures of [Ru{η-C(CN)2CPhC=C(CN)2}(PPh3)(η-C5H5)] and [Ru{C[=C(CN)2]CPh=C(CN)2}-(CNBut)(PPh3) (η-C5H5)]. 1981, 271–272. 10.1039/C39810000271.

[ref20] Michinobu T.; May J. C.; Lim J. H.; Boudon C.; Gisselbrecht J. P.; Seiler P.; Gross M.; Biaggio I.; Diederich F. A New Class of Organic Donor-Acceptor Molecules with Large Third-Order Optical Nonlinearities. Chem. Commun. 2005, 94, 737–739. 10.1039/b417393g. 15685321

[ref21] Wu X.; Wu J.; Liu Y.; Jen A. K. Y. Highly Efficient, Thermally and Chemically Stable Second Order Nonlinear Optical Chromophores Containing a 2-Phenyl-Tetracyanobutadienyl Acceptor. J. Am. Chem. Soc. 1999, 121, 472–473. 10.1021/ja983537.

[ref22] Cai C.; Liakatas I.; Wong M. S.; Bösch M.; Bosshard C.; Günter P.; Concilio S.; Tirelli N.; Suter U. W. Donor-Acceptor-Substituted Phenylethenyl Bithiophenes: Highly Efficient and Stable Nonlinear Optical Chromophores. Org. Lett. 1999, 1, 1847–1849. 10.1021/ol991118r.

[ref23] Mochida T.; Yamazaki S. Mono- and Diferrocenyl Complexes with Electron-Accepting Moieties Formed by the Reaction of Ferrocenylalkynes with Tetracyanoethylene. J. Chem. Soc., Dalton Trans. 2002, 18, 3559–3564. 10.1039/b204168e.

[ref24] Kivala M.; Boudon C.; Gisselbrecht J. P.; Seiler P.; Gross M.; Diederich F. Charge-Transfer Chromophores by Cycloaddition-Retro-Electrocyclization: Multivalent Systems and Cascade Reactions. Angew. Chem., Int. Ed. 2007, 46, 6357–6360. 10.1002/anie.200701733. 17659520

[ref25] Dengiz C.; Breiten B.; Gisselbrecht J. P.; Boudon C.; Trapp N.; Schweizer W. B.; Diederich F. Synthesis and Optoelectronic Properties of Janus-Dendrimer-Type Multivalent Donor-Acceptor Systems. J. Org. Chem. 2015, 80, 882–896. 10.1021/jo502367h. 25489964

[ref26] Rout Y.; Gautam P.; Misra R. Unsymmetrical and Symmetrical Push-Pull Phenothiazines. J. Org. Chem. 2017, 82, 6840–6845. 10.1021/acs.joc.7b00991. 28587457

[ref27] Patil Y.; Misra R. Diketopyrrolopyrrole-Based and Tetracyano-Bridged Small Molecules for Bulk Heterojunction Organic Solar Cells. Chem. – Asian J. 2018, 13, 220–229. 10.1002/asia.201701493. 29219247

[ref28] Koos C.; Vorreau P.; Vallaitis T.; Dumon P.; Bogaerts W.; Baets R.; Esembeson B.; Biaggio I.; Michinobu T.; Diederich F.; Freude W.; Leuthold J. All-Optical High-Speed Signal Processing with Silicon-Organic Hybrid Slot Waveguides. Nat. Photonics 2009, 3, 216–219. 10.1038/nphoton.2009.25.

[ref29] Beels M. T.; Biaggio I.; Reekie T.; Chiu M.; Diederich F. Two-Photon Absorption and Spectroscopy of the Lowest Two-Photon Transition in Small Donor-Acceptor-Substituted Organic Molecules. Phys. Rev. A 2015, 91, 043818 10.1103/PhysRevA.91.043818.

[ref30] Bui A. T.; Philippe C.; Beau M.; Richy N.; Cordier M.; Roisnel T.; Lemiègre L.; Mongin O.; Paul F.; Trolez Y. Synthesis, Characterization and Unusual near-Infrared Luminescence of 1,1,4,4-Tetracyanobutadiene Derivatives. Chem. Commun. 2020, 56, 3571–3574. 10.1039/c9cc09560h. 32104794

[ref31] Philippe C.; Bui A. T.; Batsongo-Boulingui S.; Pokladek Z.; Matczyszyn K.; Mongin O.; Lemiegre L.; Paul F.; Hamlin T. A.; Trolez Y. 1,1,4,4-Tetracyanobutadiene-Functionalized Anthracenes: Regioselectivity of Cycloadditions in the Synthesis of Small Near-IR Dyes. Org. Lett. 2021, 23, 2007–2012. 10.1021/acs.orglett.1c00136. 33635667 PMC8155560

[ref32] Winterfeld K. A.; Lavarda G.; Guilleme J.; Sekita M.; Guldi D. M.; Torres T.; Bottari G. Subphthalocyanines Axially Substituted with a Tetracyanobuta-1,3-Diene-Aniline Moiety: Synthesis, Structure, and Physicochemical Properties. J. Am. Chem. Soc. 2017, 139, 5520–5529. 10.1021/jacs.7b01460. 28322560

[ref33] Dar A. H.; Gowri V.; Gopal A.; Muthukrishnan A.; Bajaj A.; Sartaliya S.; Selim A.; Ali M. E.; Jayamurugan G. Designing of Push-Pull Chromophores with Tunable Electronic and Luminescent Properties Using Urea as the Electron Donor. J. Org. Chem. 2019, 84, 8941–8947. 10.1021/acs.joc.9b00841. 31240920

[ref34] Gowri V.; Jalwal S.; Dar A. H.; Gopal A.; Muthukrishnan A.; Bajaj A.; Ali M. E.; Jayamurugan G. A Subtle Change in Substituent Enabled Multi-Ways Fluorine Anion Signals Including Paper-Strip Colorimetric Detection Using Urea-Functionalized Push–Pull Chromophore Receptor. J. Photochem. Photobiol., A 2021, 410, 113163 10.1016/j.jphotochem.2021.113163.

[ref35] Xu J.; Liu X.; Lv J.; Zhu M.; Huang C.; Zhou W.; Yin X.; Liu H.; Li Y.; Ye J. Morphology Transition and Aggregation-Induced Emission of an Intramolecular Charge-Transfer Compound. Langmuir 2008, 24, 4231–4237. 10.1021/la703662w. 18312013

[ref36] Dar A. H.; Gowri V.; Mishra R. K.; Khan R.; Jayamurugan G. Nanotechnology-Assisted, Single-Chromophore-Based White-Light-Emitting Organic Materials with Bioimaging Properties. Langmuir 2022, 38, 430–438. 10.1021/acs.langmuir.1c02797. 34965146

[ref37] Li Y.; Ashizawa M.; Uchida S.; Michinobu T. Colorimetric Sensing of Cations and Anions by Clicked Polystyrenes Bearing Side Chain Donor-Acceptor Chromophores. Polym. Chem. 2012, 3, 1996–2005. 10.1039/c2py20230a.

[ref38] Ohshita J.; Kajihara T.; Tanaka D.; Ooyama Y. Preparation of Poly(Disilanylenetetracyanobutadienyleneoligothienylene)s as New Donor-Acceptor Type Organosilicon Polymers. J. Organomet. Chem. 2014, 749, 255–260. 10.1016/j.jorganchem.2013.10.007.

[ref39] Li Y.; Ashizawa M.; Uchida S.; Michinobu T. A Novel Polymeric Chemosensor: Dual Colorimetric Detection of Metal Ions through Click Synthesis. Macromol. Rapid Commun. 2011, 32, 1804–1808. 10.1002/marc.201100397. 21905148

[ref40] Jayamurugan G.; Gowri V.; Hernández D.; Martin S.; González-Orive A.; Dengiz C.; Dumele O.; Pérez-Murano F.; Gisselbrecht J.-P.; Boudon C.; Schweizer W. B.; Breiten B.; Finke A. D.; Jeschke G.; Bernet B.; Ruhlmann L.; Cea P.; Diederich F. Design and Synthesis of Aviram–Ratner-Type Dyads and Rectification Studies in Langmuir–Blodgett (LB) Films. Chem. – Eur. J. 2016, 22, 10539–10547. 10.1002/chem.201505216. 27363287

[ref41] Rout Y.; Chauhan V.; Misra R. Synthesis and Characterization of Isoindigo-Based Push-Pull Chromophores. J. Org. Chem. 2020, 85, 4611–4618. 10.1021/acs.joc.9b03267. 32126766

[ref42] Kivala M.; Boudon C.; Gisselbrecht J. P.; Seiler P.; Gross M.; Diederich F. A Novel Reaction of 7,7,8,8-Tetracyanoquinodimethane (TCNQ): Charge-Transfer Chromophores by [2 + 2] Cycloaddition with Alkynes. Chem. Commun. 2007, 4731–4733. 10.1039/b713683h. 18004423

[ref43] Dar A.; Gowri V.; Neethu K. M.; Jayamurugan G. Synthesis of 1,1,4,4-Tetracyanobuta-1,3-Dienes Using Tetracyanoethylene Oxide via [3 +2]-Cycloaddition-Ring Opening Reaction. ChemistrySelect 2020, 5, 12437–12441. 10.1002/slct.202003179.

[ref44] Hünig S.; Herberth E. N,N′-Dicyanoquinone Diimines (DCNQIs): Versatile Acceptors for Organic Conductors. Chem. Rev. 2004, 104, 5535–5563. 10.1021/cr030637b. 15535659

[ref45] Finke A. D.; Diederich F. 6,6-Dicyanopentafulvenes: Teaching an Old Dog New Tricks. Chem. Rec. 2015, 15, 19–30. 10.1002/tcr.201402060. 25308196

[ref46] Donckele E. J.; Finke A. D.; Ruhlmann L.; Boudon C.; Trapp N.; Diederich F. The [2 + 2] Cycloaddition-Retroelectrocyclization and [4 + 2] Hetero-Diels-Alder Reactions of 2-(Dicyanomethylene)Indan-1,3-Dione with Electron-Rich Alkynes: Influence of Lewis Acids on Reactivity. Org. Lett. 2015, 17, 3506–3509. 10.1021/acs.orglett.5b01598. 26135390

[ref47] Tchitchanov B. H.; Chiu M.; Jordan M.; Kivala M.; Schweizer W. B.; Diederich F. Platinum(II) Acetylides in the Formal [2+2] Cycloaddition- Retroelectrocyclization Reaction: Organodonor versus Metal Activation. Eur. J. Org. Chem. 2013, 2013, 3729–3740. 10.1002/ejoc.201300300.

[ref48] Michinobu T.; Boudon C.; Gisselbrecht J. P.; Seiler P.; Frank B.; Moonen N. N. P.; Gross M.; Diederich F. Donor-Substituted 1,1,4,4-Tetracyanobutadienes (TCBDs): New Chromophores with Efficient Intramolecular Charge-Transfer Interactions by Atom-Economic Synthesis. Chem. – Eur. J. 2006, 12, 1889–1905. 10.1002/chem.200501113. 16389622

[ref49] Niu S.; Ulrich G.; Retailleau P.; Ziessel R. Regioselective Synthesis of 5-Monostyryl and 2-Tetracyanobutadiene BODIPY Dyes. Org. Lett. 2011, 13, 4996–4999. 10.1021/ol201600s. 21902197

[ref50] Shoji T.; Higashi J.; Ito S.; Okujima T.; Yasunami M.; Morita N. Synthesis of Redox-Active, Intramolecular Charge-Transfer Chromophores by the [2+2] Cycloaddition of Ethynylated 2H-Cyclohepta[b]Furan-2-Ones with Tetracyanoethylene. Chem. – Eur. J. 2011, 17, 5116–5129. 10.1002/chem.201003628. 21452182

[ref51] Pengxia L.; Du Z.; Wang D.; Yang Z.; Sheng H.; Liang S.; Cao H.; He W.; Yang H. Optoelectronic and Self-Assembly Properties of Porphyrin Derivatives with Click Chemistry Modification. ChemPhysChem 2014, 15, 3523–3529. 10.1002/cphc.201402401. 25155781

[ref52] Kato S. ichiro.; Noguchi H.; Jin S.; Nakamura Y. Synthesis and Electronic, Optical, and Electrochemical Properties of a Series of Tetracyanobutadiene-Substituted Carbazoles. Asian J. Org. Chem. 2016, 5, 246–256. 10.1002/ajoc.201500431.

[ref53] Betou M.; Durand R. J.; Sallustrau D. A.; Gousset C.; Le Coz E.; Leroux Y. R.; Toupet D. L.; Trzop E.; Roisnel T.; Trolez Y. Reactivity of Functionalized Ynamides with Tetracyanoethylene: Scope, Limitations and Optoelectronic Properties of the Adducts. Chem. – Asian J. 2017, 12, 1338–1346. 10.1002/asia.201700353. 28407369

[ref54] Shoji T.; Takagaki S.; Ariga Y.; Yamazaki A.; Takeuchi M.; Ohta A.; Sekiguchi R.; Mori S.; Okujima T.; Ito S. Molecular Transformation to Pyrroles, Pentafulvenes, and Pyrrolopyridines by [2+2] Cycloaddition of Propargylamines with Tetracyanoethylene. Chem. – Eur. J. 2020, 26, 1931–1935. 10.1002/chem.201904926. 31750583

[ref55] Poddar M.; Rout Y.; Misra R. Donor-Acceptor Based 1,8-Naphthalimide Substituted Phenothiazines: Tuning of HOMO-LUMO Gap. Asian J. Org. Chem. 2021, 11, e202100628 10.1002/ajoc.202100628.

[ref56] Erden K.; Savaş İ.; Dengiz C. Synthesis of Triazene-Substituted Homoconjugated Push-Pull Chromophores by Formal [2 + 2] Cycloadditions. Tetrahedron Lett. 2019, 60, 1982–1985. 10.1016/j.tetlet.2019.06.046.

[ref57] Mammadova F.; Ozsinan S.; Okutan M.; Dengiz C. Synthesis, Characterization, and Theoretical Investigation of Optical and Nonlinear Optical (NLO) Properties of Triazene-Based Push–Pull Chromophores. J. Mol. Struct. 2020, 1220, 128726 10.1016/j.molstruc.2020.128726.

[ref58] Gautam P.; Maragani R.; Misra R. Tuning the HOMO-LUMO Gap of Donor-Substituted Benzothiazoles. Tetrahedron Lett. 2014, 55, 6827–6830. 10.1016/j.tetlet.2014.10.094.

[ref59] Shoji T.; Higashi J.; Ito S.; Okujima T.; Yasunami M.; Morita N. Synthesis of Donor-Acceptor Chromophores by the [2 + 2] Cycloaddition of Arylethynyl-2H-Cyclohepta[b]Furan-2-Ones with 7,7,8,8-Tetracyanoquinodimethane. Org. Biomol. Chem. 2012, 10, 2431–2438. 10.1039/c2ob06931h. 22331190

[ref60] Michinobu T.; Yamada N.; Washino Y.; Nakayama K. I. Novel Design of Carbazole-Based Donor-Acceptor Molecules for Fullerene-Free Organic Photovoltaic Devices. J. Nanosci. Nanotechnol. 2016, 16, 8662–8669. 10.1166/jnn.2016.11905.

[ref61] Vacher A.; Auffray M.; Barrière F.; Roisnel T.; Lorcy D. Electronic Interplay between TTF and Extended-TCNQ Electrophores along a Ruthenium Bis(Acetylide) Linker. Org. Lett. 2017, 19, 6060–6063. 10.1021/acs.orglett.7b02818. 29120184

[ref62] Shoji T.; Miura K.; Araki T.; Maruyama A.; Ohta A.; Sekiguchi R.; Ito S.; Okujima T. Synthesis of 2-Methyl-1-Azulenyl Tetracyanobutadienes and Dicyanoquinodimethanes: Substituent Effect of 2-Methyl Moiety on the Azulene Ring toward the Optical and Electrochemical Properties. J. Org. Chem. 2018, 83, 6690–6705. 10.1021/acs.joc.8b01067. 29799742

[ref63] Poddar M.; Jang Y.; Misra R.; D’Souza F. Excited-State Electron Transfer in 1,1,4,4-Tetracyanobuta-1,3-Diene (TCBD)- and Cyclohexa-2,5-Diene-1,4-Diylidene-Expanded TCBD-Substituted BODIPY-Phenothiazine Donor–Acceptor Conjugates. Chem. – Eur. J. 2020, 26, 6869–6878. 10.1002/chem.202000346. 32160356

[ref64] Abdul Raheem A.; Kumar C.; Shanmugam R.; Murugan P.; Praveen C. Molecular Engineering of Twisted Dipolar Chromophores for Efficiency Boosted BHJ Solar Cells. J. Mater. Chem. C 2021, 9, 4562–4575. 10.1039/d1tc00708d.

[ref65] Wen J.; Shi Z. From C4 to C7: Innovative Strategies for Site-Selective Functionalization of Indole C-H Bonds. Acc. Chem. Res. 2021, 54, 1723–1736. 10.1021/acs.accounts.0c00888. 33709705

[ref66] Kumar S.; Ritika A Brief Review of the Biological Potential of Indole Derivatives. Future J. Pharm. Sci. 2020, 6, 121 10.1186/s43094-020-00141-y.

[ref67] Li Q.; Li Z.; Ye C.; Qin J. New Indole-Based Chromophore-Containing Main-Chain Polyurethanes: Architectural Modification of Isolation Group, Enhanced Nonlinear Optical Property, and Improved Optical Transparency. J. Phys. Chem. B 2008, 112, 4928–4933. 10.1021/jp7105988. 18380500

[ref68] Washburn D. G.; Hoang T. H.; Campobasso N.; Smallwood A.; Parks D. J.; Webb C. L.; Frank K. A.; Nord M.; Duraiswami C.; Evans C.; Jaye M.; Thompson S. K. Synthesis and SAR of Potent LXR Agonists Containing an Indole Pharmacophore. Bioorg. Med. Chem. Lett. 2009, 19, 1097–1100. 10.1016/j.bmcl.2009.01.004. 19167885

[ref69] Baert F.; Cabanetos C.; Allain M.; Silvestre V.; Leriche P.; Blanchard P. Thieno[2,3-b]Indole-Based Small Push-Pull Chromophores: Synthesis, Structure, and Electronic Properties. Org. Lett. 2016, 18, 1582–1585. 10.1021/acs.orglett.6b00438. 27003243

[ref70] Thorley K. J.; Hales J. M.; Kim H.; Ohira S.; Brédas J. L.; Perry J. W.; Anderson H. L. Cyanine-like Dyes with Large Bond-Length Alternation. Chem. – Eur. J. 2013, 19, 10370–10377. 10.1002/chem.201300609. 23788404

[ref71] Sha F.; Tao Y.; Tang C. Y.; Zhang F.; Wu X. Y. Construction of Benzo[c]Carbazoles and Their Antitumor Derivatives through the Diels-Alder Reaction of 2-Alkenylindoles and Arynes. J. Org. Chem. 2015, 80, 8122–8133. 10.1021/acs.joc.5b01223. 26204058

[ref72] De Simone F.; Saget T.; Benfatti F.; Almeida S.; Waser J. Formal Homo-Nazarov and Other Cyclization Reactions of Activated Cyclopropanes. Chem. – Eur. J. 2011, 17, 14527–14538. 10.1002/chem.201102583. 22113928

[ref73] Erden K.; Savaş İ.; Dengiz C. Synthesis of Triazene-Substituted Homoconjugated Push-Pull Chromophores by Formal [2 + 2] Cycloadditions. Tetrahedron Lett. 2019, 60, 1982–1985. 10.1016/j.tetlet.2019.06.046.

[ref74] Dengiz Ç. Polycyclic Aromatic Hydrocarbon-Substituted Push–Pull Chromophores: An Investigation of Optoelectronic and Nonlinear Optical Properties Using Experimental and Theoretical Approaches. Turk. J. Chem. 2021, 45, 1375–1390. 10.3906/kim-2102-22. 34849054 PMC8596527

[ref75] Zhou N.; Wang L.; Thompson D. W.; Zhao Y. OPE/OPV H-Mers: Synthesis, Electronic Properties, and Spectroscopic Responses to Binding with Transition Metal Ions. Tetrahedron 2011, 67, 125–143. 10.1016/j.tet.2010.11.012.

[ref76] Sattler L. E.; Hilt G. Iodonium Cation-Pool Electrolysis for the Three-Component Synthesis of 1,3-Oxazoles. Chem. – Eur. J. 2021, 27, 605–608. 10.1002/chem.202004140. 33270278 PMC7839530

[ref77] González-Rodríguez E.; Guzmán-Juárez B.; Miranda-Olvera M.; Carreón-Castro M. d. P.; Maldonado-Domínguez M.; Arcos-Ramos R.; Farfán N.; Santillan R. Effect of the π-Bridge on the Light Absorption and Emission in Push-Pull Coumarins and on Their Supramolecular Organization. Spectrochim. Acta, Part A 2022, 267, 120520 10.1016/j.saa.2021.120520. 34739896

[ref78] Zhang X.; Xie X.; Liu Y. Nickel-Catalyzed Highly Regioselective Hydrocyanation of Terminal Alkynes with Zn(CN)2 Using Water as the Hydrogen Source. J. Am. Chem. Soc. 2018, 140, 7385–7389. 10.1021/jacs.8b02542. 29851478

[ref79] Perrin F. G.; Kiefer G.; Jeanbourquin L.; Racine S.; Perrotta D.; Waser J.; Scopelliti R.; Severin K. 1-Alkynyltriazenes as Functional Analogues of Ynamides. Angew. Chem., Int. Ed. 2015, 54, 13393–13396. 10.1002/anie.201507033. 26374083

[ref80] Reutenauer P.; Kivala M.; Jarowski P. D.; Boudon C.; Gisselbrecht J. P.; Gross M.; Diederich F. New Strong Organic Acceptors by Cycloaddition of TCNE and TCNQ to Donor-Substituted Cyanoalkynes. Chem. Commun. 2007, 40, 4898–4900. 10.1039/b714731g. 18361362

[ref81] Fesser P.; Iacovita C.; Wäckerlin C.; Vijayaraghavan S.; Ballav N.; Howes K.; Gisselbrecht J. P.; Crobu M.; Boudon C.; Stöhr M.; Jung T. A.; Diederich F. Visualizing the Product of a Formal Cycloaddition of 7,7,8,8-Tetracyano-p- Quinodimethane (TCNQ) to an Acetylene-Appended Porphyrin by Scanning Tunneling Microscopy on Au(111). Chem. – Eur. J. 2011, 17, 5246–5250. 10.1002/chem.201100733. 21484902

[ref82] Dengiz C.; Prange C.; Gawel P.; Trapp N.; Ruhlmann L.; Boudon C.; Diederich F. Push-Pull Chromophores by Reaction of 2,3,5,6-Tetrahalo-1,4-Benzoquinones with 4-(N,N-Dialkylanilino)Acetylenes. Tetrahedron 2016, 72, 1213–1224. 10.1016/j.tet.2016.01.017.

[ref83] Bureš F.; Pytela O.; Kivala M.; Diederich F. Solvatochromism as an Efficient Tool to Study N,N-Dimethylamino- and Cyano-Substituted π-Conjugated Molecules with an Intramolecular Charge-Transfer Absorption. J. Phys. Org. Chem. 2011, 24, 274–281. 10.1002/poc.1744.

[ref84] Frisch M. J.; Trucks G. W.; Schlegel H. B.; Scuseria G. E.; Robb M. A.; Cheeseman J. R.; Scalmani G.; Barone V.; Mennucci B.; Petersson G. A.; Nakatsuji H.; Caricato M.; Li X.; Hratchian H. P.; Izmaylov A. F.; Bloino J.; Zheng G.; Sonnenberg J. L.; Had M.; Fox D. J.; Frisch M. J.; Trucks G. W.; Schlegel H. B.; Scuseria G. E.; Robb M. A.; Cheeseman J. R.; Scalmani G.; Barone V.; Mennucci B.; Petersson G. A.; Nakatsuji H.; Caricato M.; Li X.; Hratchian H. P.; Izmaylov A. F.; Bloino J.; Zheng G.; Sonnenberg J. L.; Hada M.; Ehara M.; Toyota K.; Fukuda R.; Hasegawa J.; Ishida M.; Nakajima T.; Honda Y.; Kitao O.; Nakai H.; Vreven T.; Montgomery J. A. Jr.; Peralta J. E.; Ogliaro F.; Bearpark M.; Heyd J. J.; Brothers E.; Kudin K. N.; Staroverov V. N.; Kobayashi R.; Normand J.; Raghavachari K.; Rendell A.; Burant J. C.; Iyengar S. S.; Tomasi J.; Cossi M.; Rega N.; Millam J. M.; Klene M.; Knox J. E.; Cross J. B.; Bakken V.; Adamo C.; Jaramillo J.; Gomperts R.; Stratmann R. E.; Yazyev O.; Austin A. J.; Cammi R.; Pomelli C.; Ochterski J. W.; Martin R. L.; Morokuma K.; Zakrzewski V. G.; Voth G. A.; Salvador P.; Dannenberg J. J.; Dapprich S.; Daniels A. D.; Farkas Ö.; Foresman J. B.; Ortiz J. V.; Cioslowski J.; Fox D. J. Gaussian 09, revision D.01; Gaussian Inc.: Wallingford, 2013.

[ref85] Jamorski Jödicke C.; Lüthi H. P. Time-Dependent Density Functional Theory (TDDFT) Study of the Excited Charge-Transfer State Formation of a Series of Aromatic Donor-Acceptor Systems. J. Am. Chem. Soc. 2003, 125, 252–264. 10.1021/ja020361. 12515528

[ref86] Zouaoui-Rabah M.; Sekkal-Rahal M.; Djilani-Kobibi F.; Elhorri A. M.; Springborg M. Performance of Hybrid DFT Compared to MP2 Methods in Calculating Nonlinear Optical Properties of Divinylpyrene Derivative Molecules. J. Phys. Chem. A 2016, 120, 8843–8852. 10.1021/acs.jpca.6b08040. 27749050

[ref87] Gu Y.; Wang X. min. Direct Palladium-Catalyzed C-3 Alkynylation of Indoles. Tetrahedron Lett. 2009, 50, 763–766. 10.1016/j.tetlet.2008.11.097.

[ref88] Chatzopoulou E.; Davies P. W. Highly Regioselective Synthesis of 2,4,5-(Hetero)Aryl Substituted Oxazoles by Intermolecular [3+2]-Cycloaddition of Unsymmetrical Internal Alkynes. Chem. Commun. 2013, 49, 8617–8619. 10.1039/c3cc45410j. 23958931

[ref89] Brachet E.; Belmont P. Palladium-Catalyzed Regioselective Alkynylation of Pyrroles and Azoles under Mild Conditions: Application to the Synthesis of a Dopamine D-4 Receptor Agonist. J. Org. Chem. 2015, 80, 7519–7529. 10.1021/acs.joc.5b01093. 26176588

