# Supplementary material for: 3-Alkynylindoles as Building Blocks for the Synthesis of Electronically Tunable Indole-Based Push–Pull Chromophores
Source: J Org Chem. 2022 Mar 1;87(6):4385–99. doi: 10.1021/acs.joc.2c00067 (PMC8938952; doi:10.1021/acs.joc.2c00067)
Supplement: Supplementary file 1 — jo2c00067_si_001.pdf [file jo2c00067_si_001.pdf]

Supporting Information

The Journal of Organic Chemistry

3-Alkynylindoles as Building Blocks for the Synthesis of Electronically-Tunable Indole-Based Push-Pull Chromophores

Kübra Erden, Cagatay Dengiz*

Department of Chemistry, Middle East Technical University, 06800 Ankara, Turkey.

Corresponding author.
E-mail address: dengizc@metu.edu.tr (C. Dengiz)

Table of Contents

1. ***Theoretical Calculations*** ***S3***

2. ***^{1}H and ^{13}C NMR Spectra*** ***S41***

3. ***High-Resolution Mass Spectrometry (HR-MS) Data*** ***S68***

4. ***UV/Vis Spectra*** ***S81***

1. Theoretical Calculations

All structures are confirmed ground-state minima according to the analysis of their analytical frequencies computed at the same level, which show no imaginary frequencies. On the optimized molecular structures of **5a–i** and **7a–i** at the B3LYP/6-31G(d) level of theory with the CPCM solvation model in CH_2Cl_2, the vertical optical transitions were calculated by time-dependent density functional theory (TD-DFT) at the CAM-B3LYP/6–31G(d) level of theory, again with the CPCM solvation model in CH_2Cl_2 using the software package Gaussian 09.[1]

Table S1. Depiction of calculated HOMOs and LUMOs over optimized ground-state geometries, transition energies (*E*), and oscillator strengths (*f*) for **5a**.

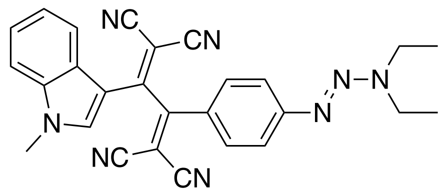

Exptl.: λ = 442 nm (in CH_2Cl_2)

Excited state	**Δ*E* (eV)**	***λ* (nm)**	***f***	**assignments**
1	2.88	431	0.4909	H➔L
2	3.05	407	0.5806	H–1➔L, H➔L
3	3.73	332	0.2054	H–1➔L+1, H➔L+1
4	3.75	331	0.0873	H–3➔L, H–3➔L+2

**Orbital**		***E* (eV)**
HOMO–1	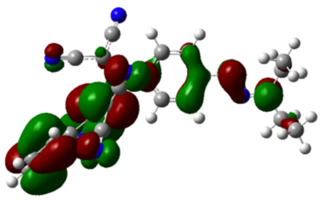	-6.09
HOMO	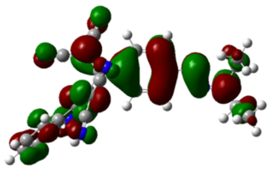	–5.94
LUMO	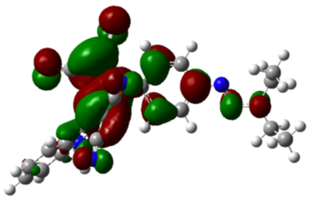	–3.18
LUMO+1	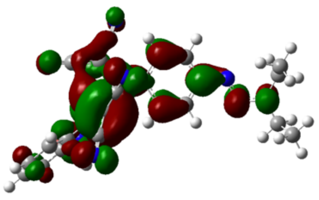	–2.34

Table S2. Depiction of calculated HOMOs and LUMOs over optimized ground-state geometries, transition energies (*E*), and oscillator strengths (*f*) for **5b**.

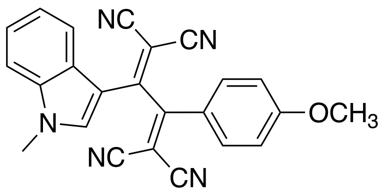

Exptl.: λ = 428 nm (in CH_2Cl_2)

Excited state	**Δ*E* (eV)**	***λ* (nm)**	***f***	**assignments**
1	2.92	424	0.2837	H➔L
2	3.36	369	0.3585	H–1➔L
3	3.79	327	0.3106	H–2➔L, H➔L+1
4	3.88	319	0.0387	H–2➔L, H–2➔L+1

**Orbital**		***E* (eV)**
HOMO–1	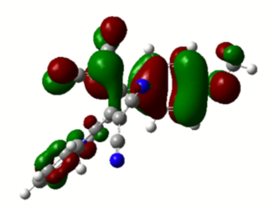	-6.46
HOMO	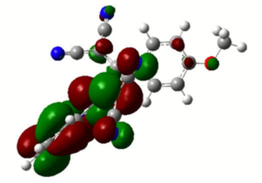	–6.07
LUMO	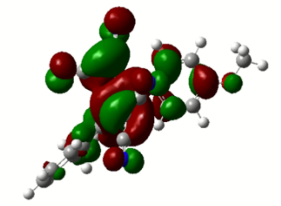	–3.18
LUMO+1	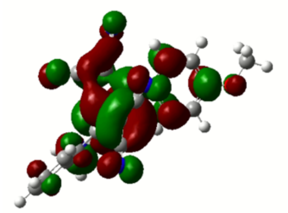	–2.27

Table S3. Depiction of calculated HOMOs and LUMOs over optimized ground-state geometries, transition energies (*E*), and oscillator strengths (*f*) for **5c**.

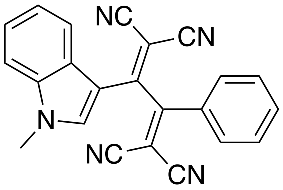

Exptl.: λ = 426 nm (in CH_2Cl_2)

**Excited state**	**Δ*E* (eV)**	**λ (nm)**	***f***	**assignments**
1	2.84	437	0.2309	H➔L
2	3.69	336	0.1674	H–1➔L, H➔L+1
3	3.82	325	0.1590	H–2➔L, H–1➔L
4	3.84	323	0.3743	H➔L+1

**Orbital**		***E* (eV)**
HOMO–1	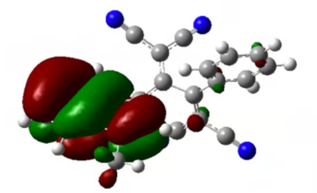	-6.74
HOMO	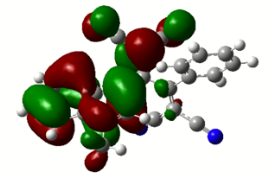	–6.12
LUMO	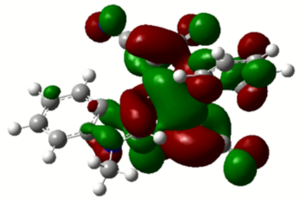	–3.31
LUMO+1	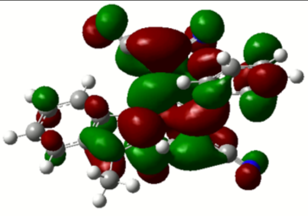	–2.32

Table S4. Depiction of calculated HOMOs and LUMOs over optimized ground-state geometries, transition energies (*E*), and oscillator strengths (*f*) for **5d**.

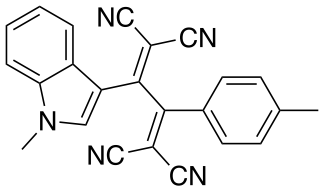

Exptl.: λ = 427 nm (in CH_2Cl_2)

**Excited state**	**Δ*E* (eV)**	***λ* (nm)**	***f***	**assignments**
1	2.86	433	0.2429	H➔L
2	3.60	345	0.2883	H–1➔L, H–2➔L
3	3.78	328	0.3453	H–2➔L, H➔L+1
4	3.85	323	0.0960	H–1➔L, H➔L+1

**Orbital**		***E* (eV)**
HOMO–1	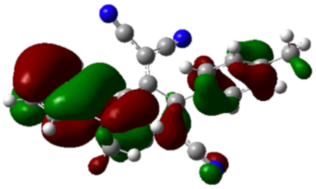	-6.71
HOMO	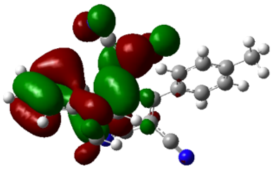	–6.10
LUMO	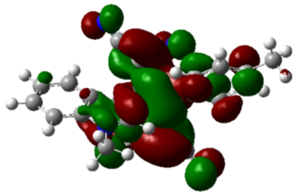	–3.26
LUMO+1	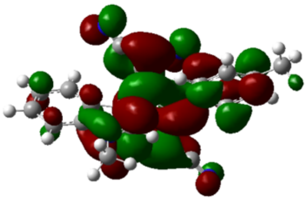	–2.30

Table S5. Depiction of calculated HOMOs and LUMOs over optimized ground-state geometries, transition energies (*E*), and oscillator strengths (*f*) for **5e**.

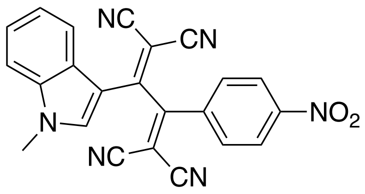

Exptl.: λ = 482 nm (in CH_2Cl_2)

**Excited state**	**ΔE (eV)**	**λ (nm)**	***f***	**assignments**
1	2.64	469	0.1704	H→L
2	3.56	348	0.1805	H–1→L, H→L+1
3	3.65	339	0.1237	H→L+1, H–1→L
4	3.88	320	0.0027	H–5→L+1

**Orbital**		***E* (eV)**
HOMO–1	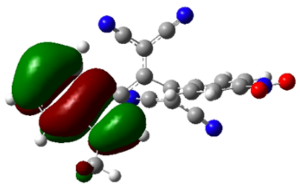	-6.80
HOMO	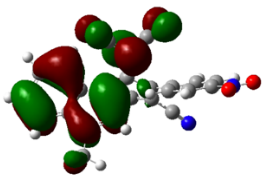	–6.19
LUMO	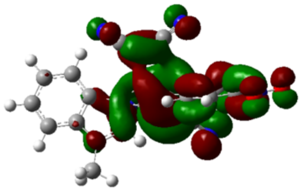	–3.67
LUMO+1	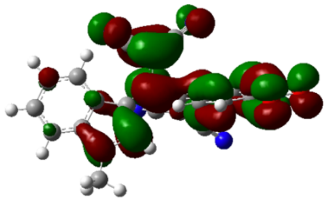	–2.88

Table S6. Depiction of calculated HOMOs and LUMOs over optimized ground-state geometries, transition energies (*E*), and oscillator strengths (*f*) for **5f**.

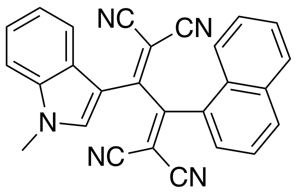

Exptl.: λ = 431 nm (in CH_2Cl_2)

**Excited state**	**Δ*E* (eV)**	**λ (nm)**	***f***	**assignments**
1	2.61	475	0.1850	H➔L
2	2.87	432	0.2100	H–1➔L
3	3.50	354	0.0176	H–2➔L
4	3.74	332	0.0529	H–3➔L

**Orbital**		***E* (eV)**
HOMO–1	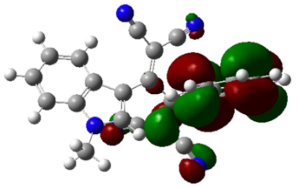	-6.33
HOMO	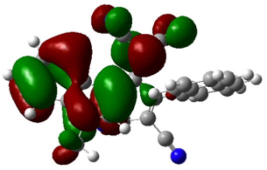	–6.12
LUMO	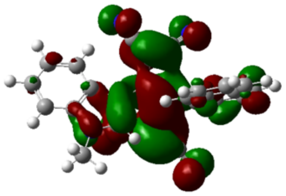	–3.52
LUMO+1	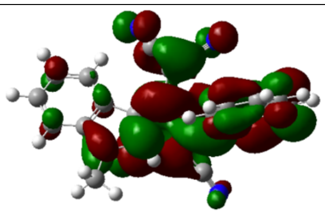	–2.17

Table S7. Depiction of calculated HOMOs and LUMOs over optimized ground-state geometries, transition energies (E), and oscillator strengths (f) for **5g**.

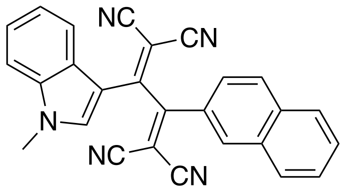

Exptl.: λ = 395 nm (in CH_2Cl_2)

**Excited state**	**ΔE (eV)**	**λ (nm)**	**f**	**assignments**
1	2.83	439	0.2340	H➔L
2	3.27	379	0.2566	H–1➔L
3	3.70	335	0.1986	H➔L+1
4	3.76	330	0.3543	H–3➔L

**Orbital**		**E (eV)**
HOMO–1	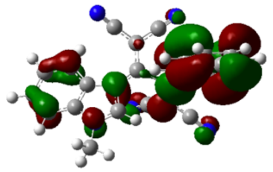	-6.36
HOMO	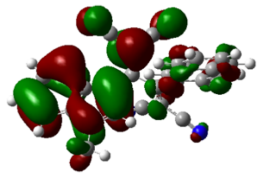	–6.08
LUMO	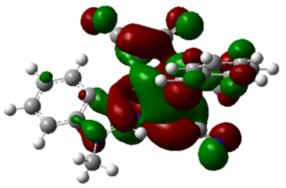	–3.29
LUMO+1	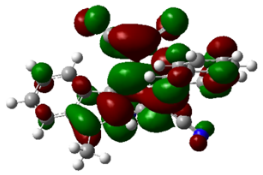	–2.37

Table S8. Depiction of calculated HOMOs and LUMOs over optimized ground-state geometries, transition energies (*E*), and oscillator strengths (*f*) for **5h**.

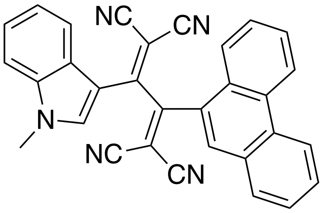

Exptl.: λ = 434 nm (in CH_2Cl_2)

Excited state	**Δ*E* (eV)**	***λ* (nm)**	***f***	**assignments**
1	2.70	460	0.2776	H➔L
2	2.93	422	0.1512	H–1➔L
3	3.42	362	0.0316	H–2➔L
4	3.63	342	0.0340	H–3➔L

**Orbital**		***E* (eV)**
HOMO–1	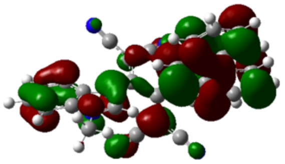	-6.27
HOMO	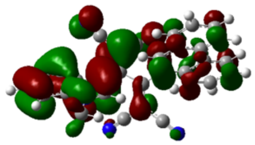	–6.07
LUMO	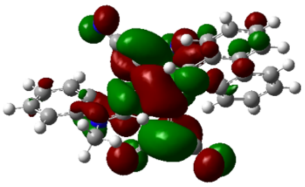	–3.40
LUMO+1	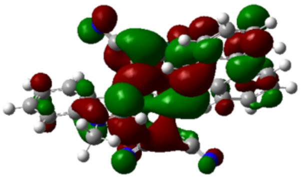	–2.26

Table S9. Depiction of calculated HOMOs and LUMOs over optimized ground-state geometries, transition energies (*E*), and oscillator strengths (*f*) for **5i**.

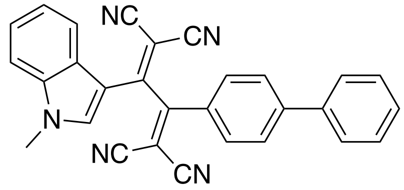

Exptl.: λ = 378 nm (in CH_2Cl_2)

**Excited state**	**ΔE (eV)**	**λ (nm)**	***f***	**assignments**
1	2.83	438	0.2358	H➔L
2	3.40	365	0.5212	H–1➔L
3	3.73	333	0.3339	H–2➔L, H➔L+1
4	3.81	326	0.0830	H–2➔L, H➔L+1

**Orbital**		***E* (eV)**
HOMO–1	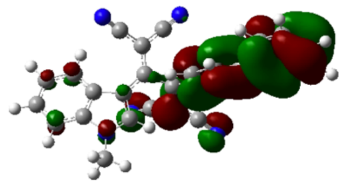	-6.47
HOMO	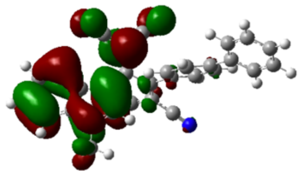	–6.10
LUMO	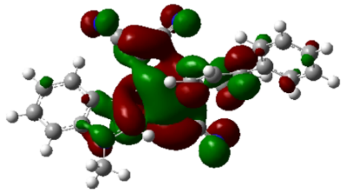	–3.31
LUMO+1	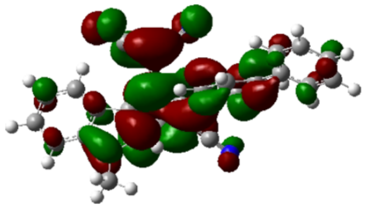	–2.37

Table S10. Depiction of calculated HOMOs and LUMOs over optimized ground-state geometries, transition energies (*E*), and oscillator strengths (*f*) for **7a**.

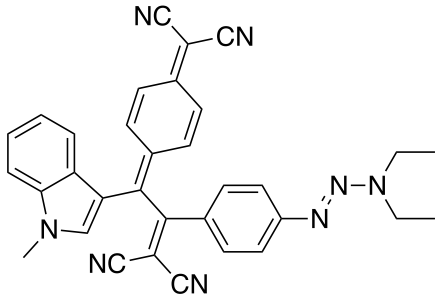

Exptl.: λ = 612 nm (in CH_2Cl_2)

**Excited state**	**Δ*E* (eV)**	**λ (nm)**	***f***	**assignments**
1	2.08	598	0.9186	H➔L
2	2.75	451	0.3579	H–1➔L
3	3.06	405	0.2208	H➔L+1
4	3.32	374	0.2927	H–2➔L

**Orbital**		***E* (eV)**
HOMO–1	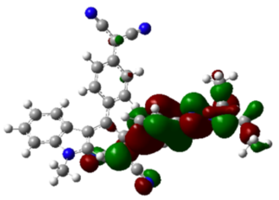	-5.95
HOMO	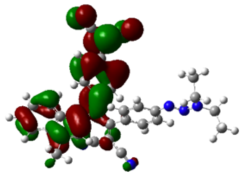	–5.48
LUMO	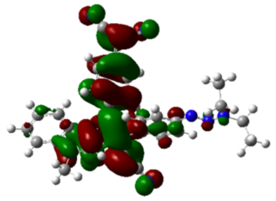	–-3.50
LUMO+1	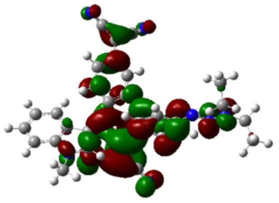	–2.55

Table S11. Depiction of calculated HOMOs and LUMOs over optimized ground-state geometries, transition energies (*E*), and oscillator strengths (*f*) for **7b**.

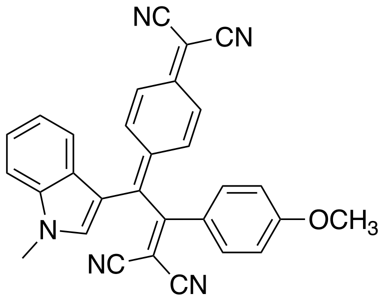

Exptl.: λ = 610 nm (in CH_2Cl_2)

Excited state	ΔE (eV)	λ (nm)	f	assignments
1	2.08	597	0.9444	H→L
2	3.04	408	0.1475	H–1→L
3	3.14	395	0.2424	H→L+1
4	3.33	373	0.2235	H–2→L

Orbital		E (eV)
HOMO–1	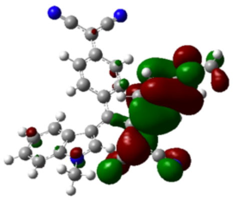	-6.43
HOMO	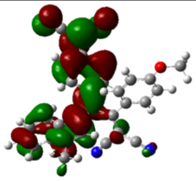	–5.51
LUMO	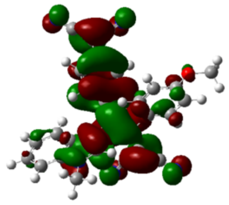	–3.52
LUMO+1	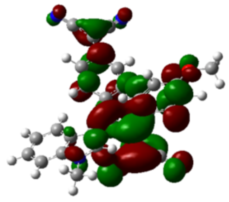	–2.46

Table S12. Depiction of calculated HOMOs and LUMOs over optimized ground-state geometries, transition energies (*E*), and oscillator strengths (*f*) for **7c**.

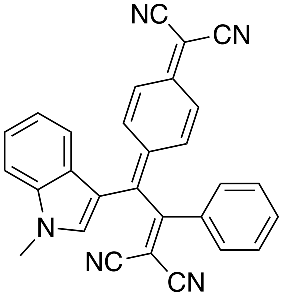

Exptl.: λ = 615 nm (in CH_2Cl_2)

**Excited state**	**Δ*E* (eV)**	***λ* (nm)**	***f***	**assignments**
1	2.03	611	0.9005	H➔L
2	3.09	401	0.2523	H➔L+1
3	3.26	380	0.2918	H–1➔L
4	3.34	371	0.0726	H–2➔L

**Orbital**		***E* (eV)**
HOMO–1	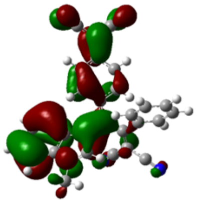	-6.69
HOMO	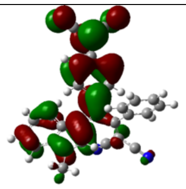	–5.53
LUMO	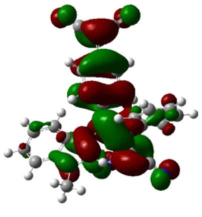	–3.61
LUMO+1	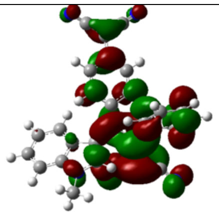	–2.52

Table S13. Depiction of calculated HOMOs and LUMOs over optimized ground-state geometries, transition energies (*E*), and oscillator strengths (*f*) for **7d**.

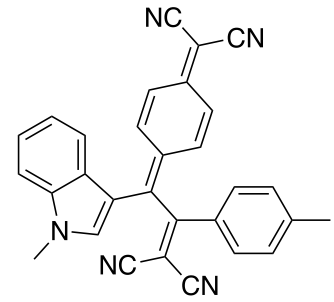

Exptl.: λ = 614 nm (in CH_2Cl_2)

Excited state	Δ*E* (eV)	*λ* (nm)	*f*	assignments
1	2.05	606	0.9134	H➔L
2	3.11	399	0.2265	H➔L+1
3	3.27	380	0.3189	H–1➔L
4	3.31	375	0.0579	H–2➔L

Orbital		*E* (eV)
HOMO–1	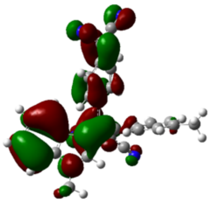	-6.67
HOMO	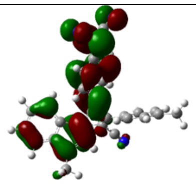	–5.52
LUMO	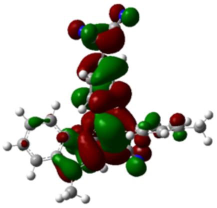	–3.58
LUMO+1	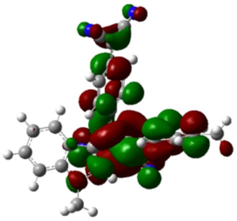	–2.50

Table S14. Depiction of calculated HOMOs and LUMOs over optimized ground-state geometries, transition energies (*E*), and oscillator strengths (*f*) for **7e**.

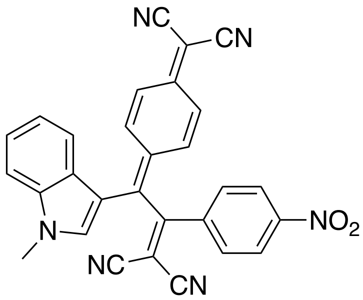

Exptl.: λ = 628 nm (in CH_2Cl_2)

**Excited state**	**Δ*E* (eV)**	**λ (nm)**	***f***	**assignments**
1	1.93	644	0.7587	H➔L
2	2.83	438	0.3486	H➔L+1
3	3.15	393	0.2877	H–1➔L
4	3.25	381	0.0861	H–2➔L

**Orbital**		***E* (eV)**
HOMO–1	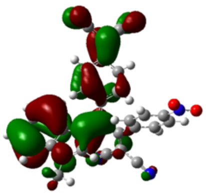	-6.74
HOMO	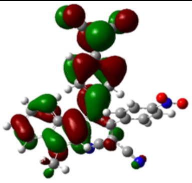	–5.60
LUMO	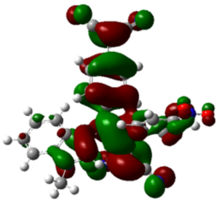	–3.82
LUMO+1	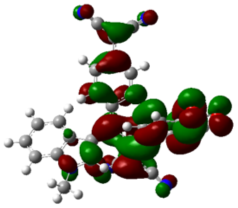	–3.09

Table S15. Depiction of calculated HOMOs and LUMOs over optimized ground-state geometries, transition energies (*E*), and oscillator strengths (*f*) for **7f**.

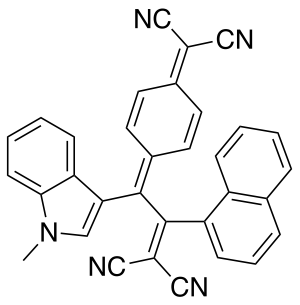

Exptl.: λ = 653 nm (in CH_2Cl_2)

**Excited state**	**Δ*E* (eV)**	***λ* (nm)**	***f***	**assignments**
1	1.95	637	0.8543	H➔L
2	2.70	459	0.0527	H–1➔L
3	3.11	398	0.2922	H–2➔L
4	3.18	390	0.1999	H➔L+1, H–3➔L

**Orbital**		***E* (eV)**
HOMO–1	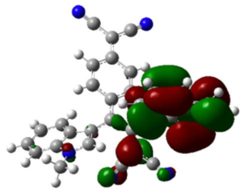	-6.31
HOMO	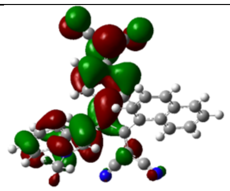	–5.55
LUMO	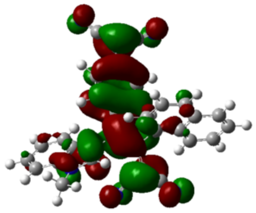	–3.71
LUMO+1	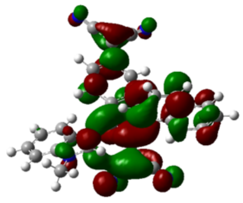	–2.41

Table S16. Depiction of calculated HOMOs and LUMOs over optimized ground-state geometries, transition energies (*E*), and oscillator strengths (*f*) for **7g**.

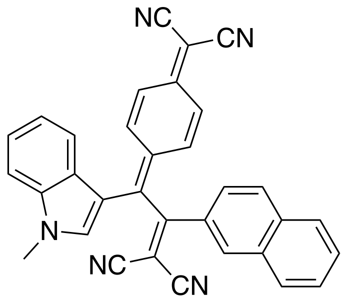

Exptl.: λ = 617 nm (in CH_2Cl_2)

Excited state	ΔE (eV)	λ (nm)	f	assignments
1	2.04	608	0.8947	H➔L
2	2.98	416	0.1525	H–1➔L
3	3.04	408	0.2469	H➔L+1
4	3.29	377	0.2354	H–2➔L

Orbital		*E* (eV)
HOMO–1	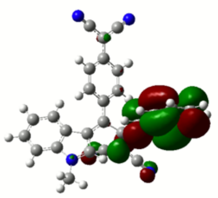	-6.33
HOMO	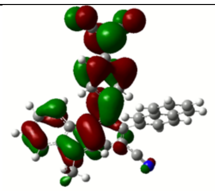	–5.52
LUMO	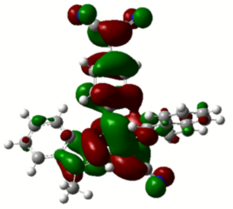	–3.58
LUMO+1	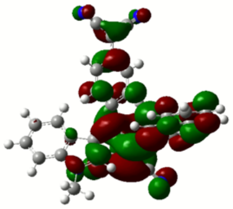	–2.58

Table S17. Depiction of calculated HOMOs and LUMOs over optimized ground-state geometries, transition energies (*E*), and oscillator strengths (*f*) for **7h**.

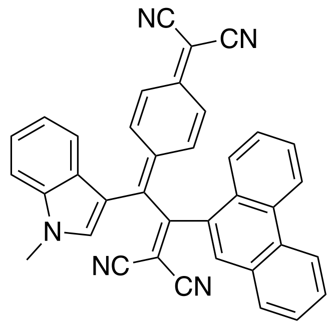

Exptl.: λ = 658 nm (in CH_2Cl_2)

Excited state	Δ*E* (eV)	*λ* (nm)	*f*	assignments
1	2.37	524	1.1596	H➔L
2	2.90	427	0.0100	H➔L+1
3	3.23	384	0.1752	H–1➔L
4	3.40	365	0.4012	H–1➔L+1

Orbital		*E* (eV)
HOMO–1	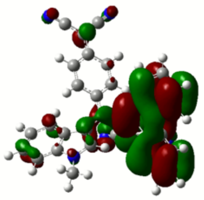	-6.25
HOMO	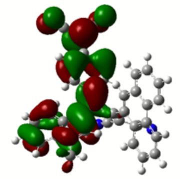	–5.59
LUMO	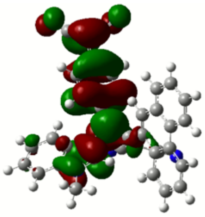	–3.26
LUMO+1	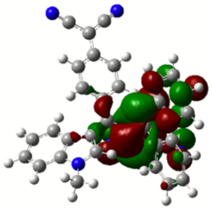	–2.86

Table S18. Depiction of calculated HOMOs and LUMOs over optimized ground-state geometries, transition energies (*E*), and oscillator strengths (*f*) for **7i**.

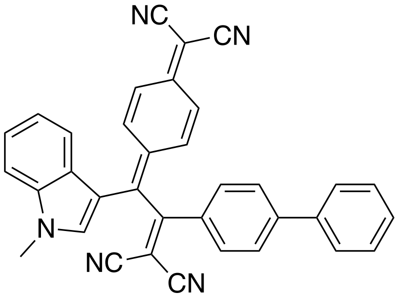

Exptl.: λ = 618 nm (in CH_2Cl_2)

**Excited state**	**Δ*E* (eV)**	**λ (nm)**	***f***	**assignments**
1	2.03	611	0.8807	H➔L
2	3.04	408	0.2236	H➔L+1
3	3.15	394	0.3063	H–1➔L
4	3.27	379	0.2416	H–2➔L

**Orbital**		***E* (eV)**
HOMO–1	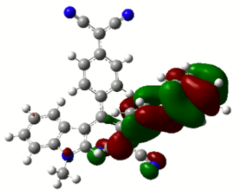	-6.45
HOMO	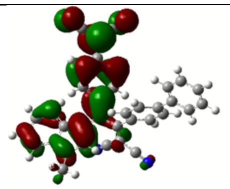	–5.53
LUMO	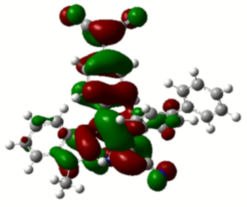	–3.60
LUMO+1	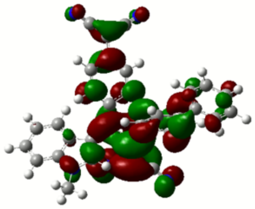	–2.58

Table S19. Dihedral angles (deviation from planarity) in between indole and quinoidal/dicyanoviynl units, obtained from optimized molecular geometries, B3LYP/6-31G(d) (CPCM solvation in DCM).

	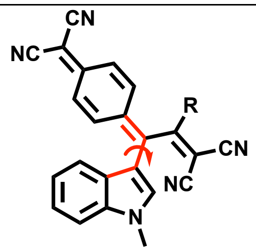		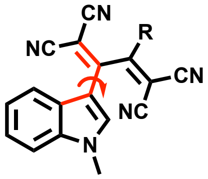
**5a**	27.8	**7a**	31.0°
**5b**	27.4°	**7b**	31.0°
**5c**	27.7°	**7c**	31.3°
**5d**	27.9°	**7d**	31.4°
**5e**	28.1°	**7e**	31.9°
**5f**	32.5°	**7f**	34.2°
**5g**	27.5°	**7g**	31.1°
**5h**	30.5°	**7h**	35.4°
**5i**	27.7°	**7i**	31.5°

5a

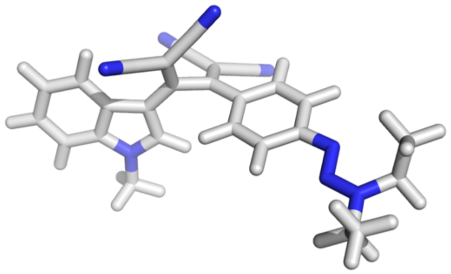

B3LYP/6-31G(d) (CPCM solvation in DCM)

Sum of electronic and zero-point Energies= -1479.577060
Sum of electronic and thermal Energies= -1479.544096
Sum of electronic and thermal Enthalpies= -1479.543152
Sum of electronic and thermal Enthalpies= -1479.645226
Imaginary Freq = 0

Atom type, (x,y,z) coordinates

C	6.09367600	-1.71535100	-1.15349400
C	6.49379400	-2.30282300	0.05972700
C	5.69033200	-2.21671000	1.19311600
C	4.47306700	-1.54709200	1.06245000
C	4.02418500	-0.98282900	-0.15890800
C	4.87535700	-1.05144300	-1.27366300
N	3.52989800	-1.25869700	2.05101600
C	2.53816200	-0.52611900	1.51037900
C	2.75072600	-0.33380100	0.13550300
C	1.82092700	0.37874200	-0.68846200
C	1.62471200	0.18977000	-2.05092800
C	0.94875500	1.40747700	-0.01119200
C	1.58341500	2.52177000	0.50333400
C	-0.48222200	1.14180000	0.06955800
C	3.62613200	-1.67303300	3.44521400
C	-1.45011900	2.17168700	0.12657500
C	-2.79808700	1.87286100	0.21204800
C	-3.24882100	0.53809400	0.26121900
C	-2.29112400	-0.49775900	0.18671200
C	-0.94606300	-0.19817600	0.07911500
N	-4.63523300	0.36635100	0.34847600
N	-4.98027600	-0.86156500	0.50560900
N	-6.27100800	-1.07246000	0.59054300
C	-6.67904500	-2.46230000	0.81716500
C	-7.26497300	0.01045300	0.54045700
C	-7.40419200	-3.08864700	-0.37575900
C	-7.55217300	0.52296100	-0.87495500
C	0.95143300	3.51323700	1.31823100
N	0.50027600	4.33577700	2.00808300
C	2.97498200	2.77048800	0.27112900
N	4.09848200	3.02133200	0.09635000
C	0.80709100	1.07662500	-2.81728900
N	0.15986500	1.79026700	-3.47269900
C	2.12730100	-0.92689900	-2.78065900
N	2.47647500	-1.84763900	-3.40378100
H	6.75030600	-1.77614100	-2.01633200
H	7.44859200	-2.81649100	0.11914800
H	6.00580000	-2.64004000	2.14120700
H	4.60554500	-0.59379400	-2.21660200
H	1.70485700	-0.20269700	2.11850100
H	4.53700600	-1.26712700	3.89427000
H	2.75909300	-1.29399700	3.98638200
H	3.64589200	-2.76431200	3.51235300
H	-1.14957100	3.20999300	0.05708700
H	-3.53725300	2.66724600	0.23245700
H	-2.61956000	-1.52989500	0.20779600
H	-0.23236200	-1.01396700	0.02328300
H	-5.76368500	-3.01201400	1.04488200
H	-7.31853300	-2.48534100	1.70778500
H	-8.17341200	-0.38459700	1.00260000
H	-6.88498600	0.82444800	1.16502100
H	-7.65117900	-4.12987800	-0.14272200
H	-8.33896700	-2.56638000	-0.60311100
H	-6.77026500	-3.07413600	-1.26810200
H	-8.29516600	1.32615300	-0.82291500
H	-6.64439800	0.92187700	-1.33337700
H	-7.95047300	-0.27037700	-1.51455600

5b

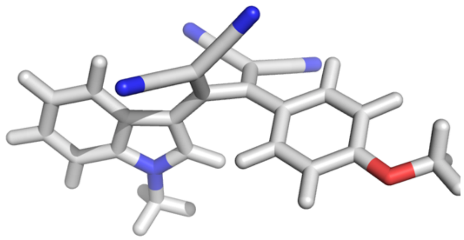

B3LYP/6-31G(d) (CPCM solvation in DCM)

Sum of electronic and zero-point Energies= -1272.149892
Sum of electronic and thermal Energies= -1272.123135
Sum of electronic and thermal Enthalpies= -1272.122190
Sum of electronic and thermal Enthalpies= -1272.208570
Imaginary Freq = 0

Atom type, (x,y,z) coordinate

C	5.14380200	1.25533000	0.08816000
C	5.72066400	0.33160500	-0.80125500
C	4.94290100	-0.64040500	-1.42403300
C	3.57592900	-0.63875400	-1.14417700
C	2.95978800	0.30749300	-0.28609400
C	3.77765000	1.24968900	0.35841800
N	2.60002700	-1.54026500	-1.57472900
C	1.42284500	-1.21579700	-1.00855500
C	1.54980300	-0.06255900	-0.21642200
C	2.83463600	-2.65945300	-2.47878500
C	0.43370700	0.52562200	0.46080500
C	0.29419400	1.86711300	0.79570800
C	-0.71635500	-0.38593300	0.81270000
C	-0.47106200	-1.36952700	1.74927200
C	-1.38676200	-2.41686600	2.08463900
N	-2.07868000	-3.30617600	2.37790100
C	-0.77182300	2.31991500	1.63276700
C	0.76507000	-1.43128200	2.47165000
C	-1.98670000	-0.17426000	0.12651700
C	-3.23105800	-0.49396600	0.71018100
C	-4.42507400	-0.28686000	0.03399700
C	-4.40778600	0.24547500	-1.26722300
C	-3.17697400	0.58437600	-1.86212900
C	-1.99606600	0.39204800	-1.17335700
C	1.11982300	2.90761600	0.27861300
N	1.74645500	-1.51238600	3.09257100
N	-1.62101600	2.71706900	2.32480500
N	1.73820300	3.79356800	-0.15798100
H	5.77712600	1.98792500	0.57961800
H	6.78885400	0.36524100	-0.99317800
H	5.38545200	-1.37610700	-2.08772100
H	3.36806500	1.96216900	1.06269700
H	0.53745500	-1.79839300	-1.22197300
H	3.57048200	-3.34307600	-2.04604000
H	1.89565600	-3.19176200	-2.63172200
H	3.20364800	-2.29326700	-3.44076200
H	-3.27741500	-0.86829900	1.72547500
H	-5.35936900	-0.52417400	0.52763900
H	-3.17882500	1.00054000	-2.86401400
H	-1.06101700	0.66047700	-1.65377400
O	-5.50443000	0.47943900	-2.01430900
C	-6.79317100	0.16366500	-1.47521800
H	-6.99577400	0.75007700	-0.57272600
H	-7.50819700	0.42987500	-2.25353500
H	-6.87352900	-0.90511700	-1.25055800

5c

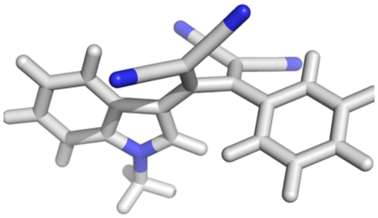

B3LYP/6-31G(d) (CPCM solvation in DCM)

Sum of electronic and zero-point Energies= -1157.654641
Sum of electronic and thermal Energies= -1157.630423
Sum of electronic and thermal Enthalpies= -1157.629479
Sum of electronic and thermal Enthalpies= -1157.710382
Imaginary Freq = 0

Atom type, (x,y,z) coordinates

C	4.57505100	1.25265700	0.61439400
C	5.27769500	0.26420400	-0.09707800
C	4.59999700	-0.74826300	-0.77044900
C	3.20580200	-0.72112400	-0.72511600
C	2.47019400	0.28727700	-0.05221700
C	3.18303800	1.27176600	0.65105400
N	2.30133600	-1.64822800	-1.24822800
C	1.05162700	-1.27906500	-0.91250000
C	1.06301600	-0.07195800	-0.19282800
C	2.66865000	-2.83259300	-2.01516100
C	-0.13884400	0.57045200	0.24717000
C	-0.30472000	1.93538900	0.45299600
C	-1.34503800	-0.30370300	0.46950800
C	-1.27493900	-1.26048600	1.45286600
C	-2.27886100	-2.25940300	1.67596300
N	-3.04218700	-3.11125200	1.88909900
C	-1.47516400	2.45942700	1.08256600
C	-0.16073300	-1.35058800	2.35281200
C	-2.51535900	-0.10009500	-0.39919000
C	-3.83278400	-0.25801800	0.07597100
C	-4.91514900	-0.06761400	-0.77737100
C	-4.70503600	0.27581600	-2.11584500
C	-3.40406900	0.44813200	-2.59610500
C	-2.31760500	0.27787200	-1.74380000
C	0.61028800	2.92556200	-0.00908600
N	0.70909000	-1.45465900	3.11841500
N	-2.41166600	2.91239000	1.60754000
N	1.30571900	3.76939400	-0.41191000
H	5.12894300	2.01724700	1.15091400
H	6.36329700	0.28003000	-0.10892800
H	5.13587900	-1.53238600	-1.29528200
H	2.67237500	2.03631000	1.22220600
H	0.20433000	-1.87098300	-1.22943200
H	3.30564200	-3.48589800	-1.41232300
H	1.76170100	-3.37045400	-2.29176800
H	3.20593900	-2.53988900	-2.92136100
H	-4.01240900	-0.48605300	1.12016200
H	-5.92473800	-0.17597700	-0.39329500
H	-5.55284400	0.42100300	-2.77876200
H	-3.23552300	0.72206400	-3.63295800
H	-1.31099000	0.41760500	-2.12492300

5d

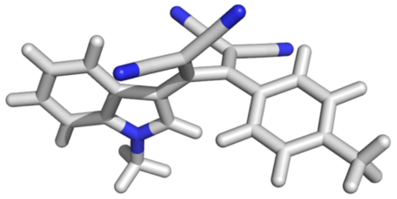

B3LYP/6-31G(d) (CPCM solvation in DCM)

Sum of electronic and zero-point Energies= -1196.946741
Sum of electronic and thermal Energies= -1196.920665
Sum of electronic and thermal Enthalpies= -1196.919720
Sum of electronic and thermal Enthalpies= -1197.005349
Imaginary Freq = 0

Atom type, (x,y,z) coordinates

C	4.85866300	1.26796200	0.32868400
C	5.49221600	0.34976000	-0.52725900
C	4.75768300	-0.62167000	-1.20144900
C	3.37593400	-0.62467500	-1.00744100
C	2.70520600	0.31665300	-0.18575500
C	3.47834300	1.25784200	0.51303100
N	2.43090900	-1.52701600	-1.50089400
C	1.21996300	-1.20713600	-1.00830200
C	1.29496100	-0.05685400	-0.20509800
C	2.72499000	-2.64380700	-2.39047400
C	0.13899700	0.52882500	0.40478800
C	-0.02155800	1.87175700	0.72512700
C	-1.02455600	-0.38506700	0.69205900
C	-0.82953100	-1.39312100	1.60843000
C	-1.77507400	-2.43553200	1.87662300
N	-2.48799500	-3.32311200	2.11817900
C	-1.12975500	2.32673200	1.50367300
C	0.37408100	-1.49348100	2.38223400
C	-2.27545400	-0.15824800	-0.04007500
C	-3.54061600	-0.42823800	0.52369900
C	-4.70033000	-0.20731600	-0.20600800
C	-4.65325500	0.27911700	-1.52341100
C	-3.39614400	0.56219600	-2.07705700
C	-2.22869100	0.36351100	-1.34982200
C	0.83083500	2.90998500	0.24853700
N	1.32312400	-1.60737900	3.04594900
N	-2.01434000	2.72401100	2.14976100
N	1.47312800	3.79315100	-0.15811900
H	5.45820700	1.99982800	0.86179100
H	6.57016800	0.38703100	-0.65217700
H	5.24318700	-1.35343100	-1.83891900
H	3.02347400	1.96581900	1.19373000
H	0.35029800	-1.78951300	-1.27919200
H	3.43645900	-3.32436100	-1.91434500
H	1.79978000	-3.18071300	-2.60039600
H	3.14995100	-2.27485900	-3.32796400
H	-3.62085500	-0.76639100	1.55001300
H	-5.66312600	-0.40235700	0.25858600
H	-3.33177300	0.95027800	-3.08973200
H	-1.27269000	0.59601200	-1.80795000
C	-5.91933600	0.48526100	-2.31433900
H	-6.31602000	-0.47589500	-2.66665000
H	-6.69968400	0.94914600	-1.70159700
H	-5.74659000	1.11510600	-3.19169000

5e

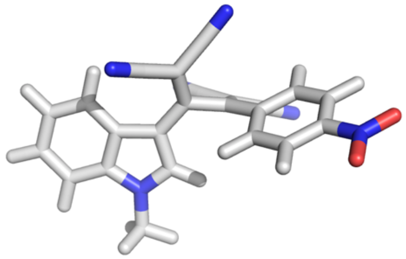

B3LYP/6-31G(d) (CPCM solvation in DCM)

Sum of electronic and zero-point Energies= -1362.151402
Sum of electronic and thermal Energies= -1362.124641
Sum of electronic and thermal Enthalpies= -1362.123697
Sum of electronic and thermal Enthalpies= -1362.211188
Imaginary Freq = 0

Atom type, (x,y,z) coordinates

C	5.25257400	-1.40948100	-0.40054800
C	5.86594300	-0.76096900	0.68568900
C	5.13310600	0.07218300	1.52650600
C	3.77175900	0.21153200	1.25590800
C	3.11815000	-0.46281600	0.19375900
C	3.89195900	-1.26496100	-0.66037100
N	2.83889700	1.03366400	1.89308200
C	1.65476200	0.92268500	1.26563000
C	1.73207500	-0.00808400	0.21386500
C	3.12451700	1.88690000	3.04071800
C	0.60179000	-0.35895700	-0.59234100
C	0.40623100	-1.58138600	-1.22783500
C	-0.48876400	0.66587700	-0.73288800
C	-0.18370700	1.88315800	-1.28434500
C	-1.09525800	2.99007200	-1.33516500
N	-1.78130100	3.92799500	-1.38246400
C	-0.63732400	-1.76821500	-2.18462800
C	1.09880900	2.16412800	-1.86644100
C	-1.83668600	0.32118700	-0.23092700
C	-3.00416300	0.71740000	-0.91061600
C	-4.25820900	0.39089900	-0.40896400
C	-4.33649000	-0.33199400	0.77956500
C	-3.20193500	-0.75433100	1.46942700
C	-1.95282300	-0.43618700	0.95145700
C	1.15920100	-2.75486500	-0.93368200
N	2.10811000	2.44148000	-2.37324600
N	-1.47290000	-1.93690200	-2.97918200
N	1.71870900	-3.74700900	-0.68796600
H	5.85251800	-2.03491700	-1.05480200
H	6.92835800	-0.89780100	0.86287000
H	5.60605700	0.60116800	2.34743000
H	3.45373100	-1.76352500	-1.51540700
H	0.79913100	1.48335300	1.61528000
H	3.89629500	2.61661900	2.78056000
H	2.21334000	2.41276100	3.32613200
H	3.47003200	1.27882800	3.88102700
H	-2.93822500	1.24796700	-1.85266800
H	-5.16175100	0.67950900	-0.93025500
H	-3.30198800	-1.32110900	2.38599800
H	-1.06334400	-0.76375100	1.47832200
N	-5.66231600	-0.67218700	1.31826900
O	-5.70927800	-1.31720300	2.36580400
O	-6.65217700	-0.29167700	0.69337200

5f

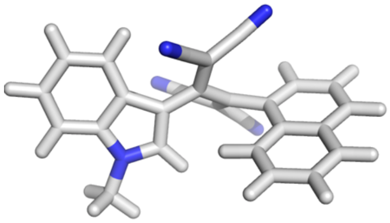

B3LYP/6-31G(d) (CPCM solvation in DCM)

Sum of electronic and zero-point Energies= -1311.245851
Sum of electronic and thermal Energies= -1311.219184
Sum of electronic and thermal Enthalpies= -1311.218239
Sum of electronic and thermal Enthalpies= -1311.303791
Imaginary Freq = 0

Atom type, (x,y,z) coordinates

C	1.49341500	-0.01006400	0.11159200
C	2.84768800	-0.42630300	-0.22823200
C	3.65737800	-0.10166000	0.88949200
N	2.84288300	0.47749200	1.86460800
C	1.58241800	0.55122200	1.39640300
C	3.47374500	-0.92902900	-1.38062400
C	4.85039500	-1.13592100	-1.36422100
C	5.62318100	-0.84272100	-0.22620100
C	5.03697900	-0.30874500	0.91807200
C	3.29928800	0.93600000	3.17115400
C	0.26525200	-0.10060800	-0.63249600
C	-0.76390600	0.96232800	-0.41848700
C	-0.01317200	-1.13756000	-1.52022500
C	-0.36275700	2.28169200	-0.40824600
C	-2.19404800	0.61756800	-0.26923500
C	-2.66382100	-0.41306200	0.62532300
C	-4.06989300	-0.70614500	0.64304300
C	-4.95298500	0.01523000	-0.20272000
C	-4.48354300	1.00332800	-1.03800500
C	-3.10891400	1.30275000	-1.06470100
C	-1.82754600	-1.12974000	1.52140400
C	-2.33994200	-2.09739100	2.36005200
C	-3.72134400	-2.39607800	2.36036900
C	-4.56628200	-1.70848900	1.51968400
C	0.72569900	-2.35728700	-1.53312300
N	1.27260500	-3.38632300	-1.54340800
C	-1.08431900	-1.10835000	-2.46472500
N	-1.91656000	-1.13822400	-3.27983100
C	-1.22959700	3.35622000	-0.01732800
N	-1.86331500	4.27093900	0.32332400
C	0.93934700	2.72868500	-0.81591100
N	1.95413500	3.17748900	-1.16702900
H	0.79791500	0.97164600	2.00964600
H	2.90740300	-1.14919100	-2.27723600
H	5.33738300	-1.53071300	-2.25090300
H	6.69416200	-1.02051000	-0.24419200
H	5.63229100	-0.05187000	1.78822500
H	4.06186300	1.71052200	3.05033700
H	2.45106300	1.34912200	3.71733200
H	3.71988500	0.09945000	3.73589100
H	-6.01175100	-0.22867100	-0.17955900
H	-5.16047900	1.54457300	-1.69108000
H	-2.75106900	2.04688900	-1.76805700
H	-0.76630000	-0.91833000	1.55879800
H	-1.67319500	-2.63128400	3.03090900
H	-4.11086500	-3.15942300	3.02722100
H	-5.63283800	-1.91702900	1.51636900

5g

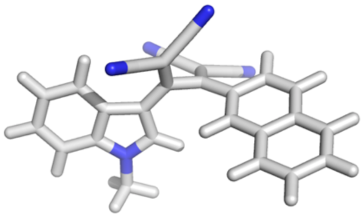

B3LYP/6-31G(d) (CPCM solvation in DCM)

Sum of electronic and zero-point Energies= -1311.253157
Sum of electronic and thermal Energies= -1311.226385
Sum of electronic and thermal Enthalpies= -1311.225441
Sum of electronic and thermal Enthalpies= -1311.311968
Imaginary Freq = 0

Atom type, (x,y,z) coordinates

C	-5.19224700	-0.96701800	1.13703400
C	-5.69887500	-1.40809000	-0.09819400
C	-4.90227200	-1.40108000	-1.23976400
C	-3.58553500	-0.96292500	-1.09526700
C	-3.03448400	-0.55507100	0.14621100
C	-3.87525000	-0.53465500	1.27083300
N	-2.61358200	-0.79138900	-2.08354400
C	-1.50337000	-0.27808600	-1.52229600
C	-1.66808800	-0.12720700	-0.13505800
C	-2.79271000	-1.10420400	-3.49614100
C	-0.61774500	0.35218700	0.71211300
C	-0.44862900	0.04696600	2.05747200
C	0.42361700	1.24094200	0.08113500
C	0.01174100	2.46659500	-0.39031200
C	1.79796300	0.73369500	-0.01195200
C	2.93512800	1.59958600	0.04263200
C	4.20418300	1.09260600	-0.07264100
C	4.43172100	-0.29968200	-0.25964200
C	3.30091300	-1.18001700	-0.29921400
C	2.00346800	-0.63676400	-0.15674300
C	5.73275600	-0.84759700	-0.39300400
C	5.90648900	-2.20506300	-0.56318800
C	4.78909500	-3.07642100	-0.60456400
C	3.51328300	-2.57592600	-0.47489700
C	-1.14126400	-1.00154400	2.72988400
N	-1.64784000	-1.87902200	3.30548400
C	0.52275900	0.72363000	2.85732100
N	1.29716400	1.27090700	3.53451300
C	0.83178600	3.35889000	-1.15435500
N	1.43582800	4.11264400	-1.80349600
C	-1.31813400	2.95642500	-0.16912300
N	-2.37836700	3.40547000	-0.00048100
H	-5.84241900	-0.95923000	2.00679900
H	-6.72936300	-1.74306000	-0.16771100
H	-5.29545800	-1.71012100	-2.20280100
H	-3.52131400	-0.18473600	2.23199900
H	-0.63054000	-0.07473500	-2.12696200
H	-3.61591000	-0.51413900	-3.90861400
H	-1.87392800	-0.86238700	-4.03067400
H	-3.01339400	-2.16798900	-3.61943300
H	2.80015700	2.65914100	0.22191200
H	5.05978100	1.75901300	-0.00683000
H	1.15657500	-1.31580000	-0.18635000
H	6.59047000	-0.18123100	-0.35970100
H	6.90722500	-2.61454500	-0.66632200
H	4.94532400	-4.14269800	-0.73832600
H	2.65258800	-3.23876900	-0.50399100

5h

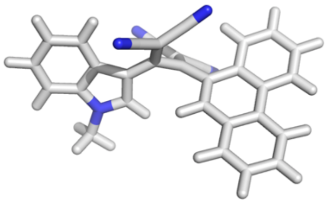

B3LYP/6-31G(d) (CPCM solvation in DCM)

Sum of electronic and zero-point Energies= -1464.845318
Sum of electronic and thermal Energies= -1464.815895
Sum of electronic and thermal Enthalpies= -1464.814950
Sum of electronic and thermal Enthalpies= -1464.906556
Imaginary Freq = 0

Atom type, (x,y,z) coordinates

C	5.74852400	0.10521600	-1.39160800
C	6.40246200	-0.53582800	-0.32429200
C	5.68827200	-0.99614700	0.77850500
C	4.30516500	-0.81290000	0.76345700
C	3.61478200	-0.20800200	-0.31728600
C	4.36711500	0.27998500	-1.39816800
N	3.37631300	-1.11333200	1.76206100
C	2.15711200	-0.70209000	1.36562000
C	2.21175100	-0.15203800	0.07406100
C	3.69316300	-1.75631600	3.03161100
C	1.04890100	0.32304600	-0.61953100
C	0.88532800	0.29300400	-2.00059000
C	-0.08981700	0.84556000	0.20848000
C	0.17227200	1.83584000	1.12923700
C	-1.40992100	0.19944800	0.06428600
C	-2.67075900	0.92677800	-0.00842600
C	-3.89760900	0.19310400	0.03420100
C	-3.87799300	-1.26206200	0.12286900
C	-2.62241800	-1.93620800	0.08984400
C	-1.41834600	-1.17698200	0.04480900
C	-5.04603300	-2.05333900	0.21437000
C	-4.97449200	-3.43552600	0.25772900
C	-3.73059800	-4.09549200	0.21201600
C	-2.57124400	-3.35158600	0.13207400
C	-2.72094900	2.32694200	-0.20455700
C	-3.92442500	2.99948300	-0.29824400
C	-5.13175000	2.28765300	-0.20478800
C	-5.11214800	0.91388900	-0.05260700
C	1.71917300	-0.46405700	-2.87417400
N	2.34796400	-1.10601800	-3.61615100
C	-0.18463400	0.97745800	-2.65300200
N	-1.02950500	1.54315600	-3.22250500
C	-0.76694900	2.24674100	2.12979700
N	-1.47586100	2.58871500	2.98661500
C	1.42205900	2.53731300	1.18772300
N	2.39945000	3.16411600	1.26638700
H	6.33302200	0.47518300	-2.22872100
H	7.48074800	-0.66027100	-0.35211300
H	6.19004600	-1.46407900	1.61926300
H	3.89434300	0.79263000	-2.22662700
H	1.29715100	-0.85195600	2.00344700
H	4.40727000	-1.14634600	3.59199500
H	2.77701600	-1.86227300	3.61282400
H	4.12456300	-2.74509800	2.85333600
H	-0.47976000	-1.72380100	0.05483200
H	-6.02255200	-1.58513000	0.25482900
H	-5.89012900	-4.01557800	0.32831800
H	-3.68710200	-5.17992600	0.24429400
H	-1.60103800	-3.84058500	0.10427700
H	-1.79843300	2.88559300	-0.30945800
H	-3.93227900	4.07424400	-0.45277100
H	-6.08111900	2.81060100	-0.27290000
H	-6.05625900	0.38299900	-0.02016700

5i

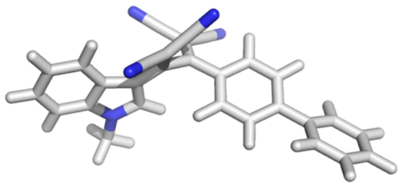

B3LYP/6-31G(d) (CPCM solvation in DCM)

Sum of electronic and zero-point Energies= -1388.633233
Sum of electronic and thermal Energies= -1388.604348
Sum of electronic and thermal Enthalpies= -1388.603404
Sum of electronic and thermal Enthalpies= -1388.695638
Imaginary Freq = 0

Atom type, (x,y,z) coordinates

C	-5.86426700	-1.47517800	0.60220800
C	-6.32529100	-1.53330700	-0.72487300
C	-5.50368400	-1.15495600	-1.78302000
C	-4.20855200	-0.74266400	-1.46808500
C	-3.70224100	-0.71585600	-0.14374800
C	-4.56849400	-1.06458400	0.90513400
N	-3.22223500	-0.24808600	-2.32426600
C	-2.14651800	0.10515500	-1.59692200
C	-2.34841100	-0.17912300	-0.23555800
C	-3.35733700	-0.12222900	-3.77038300
C	-1.34033700	0.05489000	0.75423900
C	-1.19096500	-0.64092300	1.94821500
C	-0.32414600	1.12845000	0.46031500
C	-0.77842800	2.42382400	0.36002400
C	0.02278200	3.53199900	-0.06695200
N	0.61219800	4.46632700	-0.43310100
C	-0.27525400	-0.20603000	2.95508500
C	-2.13329700	2.77933700	0.66858900
C	1.07198400	0.72080400	0.27190100
C	2.15773300	1.55129500	0.61986200
C	3.46422300	1.13503600	0.41077100
C	3.75201500	-0.12035000	-0.15940500
C	2.66706300	-0.95385800	-0.49052600
C	1.35816700	-0.55232600	-0.26681600
C	-1.85141500	-1.86969600	2.24009500
N	-3.21574500	3.12162100	0.92421800
N	0.45205100	0.13488300	3.79922800
N	-2.33227000	-2.89937500	2.49786100
H	-6.53389600	-1.75238800	1.41093100
H	-7.34030900	-1.86150000	-0.92730500
H	-5.86236300	-1.16662600	-2.80710000
H	-4.25066600	-1.01338900	1.93852700
H	-1.26976400	0.51004600	-2.08297700
H	-4.19055500	0.54373100	-4.01200500
H	-2.43499000	0.29347800	-4.17638900
H	-3.53856400	-1.10403000	-4.21616600
H	1.98438100	2.50463000	1.10453300
H	4.27707200	1.77961400	0.72904200
H	2.84993600	-1.92050600	-0.94837900
H	0.54766200	-1.21915000	-0.54270700
C	5.15015600	-0.55258200	-0.39149500
C	5.52255700	-1.90156800	-0.24849700
C	6.13760700	0.37806700	-0.76271200
C	6.83881000	-2.30506800	-0.46773400
H	4.78438400	-2.63441700	0.06378500
C	7.45229700	-0.02765700	-0.98708900
H	5.86893900	1.42096700	-0.90340900
C	7.80848200	-1.37044500	-0.83940100
H	7.10829900	-3.34996100	-0.34172600
H	8.19747500	0.70522000	-1.28349700
H	8.83354100	-1.68606500	-1.01206200

7a

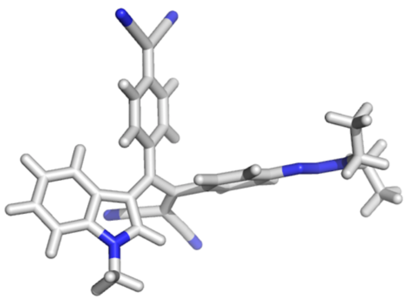

B3LYP/6-31G(d) (CPCM solvation in DCM)

**Sum of electronic and zero-point Energies=**	**-1710.541355**
**Sum of electronic and thermal Energies=**	**-1710.503619**
**Sum of electronic and thermal Enthalpies=**	**-1710.502675**
**Sum of electronic and thermal Enthalpies=**	**-1710.616537**

Imaginary Freq = 0

Atom type, (x,y,z) coordinates

C	6.32322800	-0.16319900	-0.64497100
C	6.78965500	-1.30385400	-1.32306800
C	5.92603700	-2.34826700	-1.64142900
C	4.58725300	-2.20296400	-1.27548800
C	4.08134400	-1.04969800	-0.62512200
C	4.98358800	-0.02696500	-0.29105500
N	3.54201900	-3.11911400	-1.41478500
C	2.42919000	-2.60317000	-0.85931800
C	2.66584200	-1.30789300	-0.36400400
C	1.65923600	-0.52389400	0.28852300
C	1.61328300	0.88564200	0.30130400
C	0.56373800	-1.28285600	0.98030100
C	0.91983400	-2.17412700	1.97930800
C	-0.81291900	-1.06432900	0.54099700
C	-1.92117700	-1.17788200	1.41523500
C	-3.21405600	-0.97305900	0.96949200
C	-3.46487200	-0.65725700	-0.38512100
C	-2.36678100	-0.53511300	-1.25882900
C	-1.07168300	-0.71713300	-0.80541800
C	0.82740800	1.60346100	1.26814000
C	0.78281700	2.96783500	1.29541700
C	1.49634200	3.75536700	0.33197200
C	2.25430500	3.04659600	-0.65974900
C	2.31828900	1.68292800	-0.66426800
C	1.44262700	5.16502000	0.34740800
C	3.65527500	-4.42608000	-2.04992200
C	2.14155900	5.95071300	-0.60597400
N	2.71782600	6.59352900	-1.39256700
C	0.68493200	5.87418800	1.31585600
N	0.06050300	6.45422900	2.11434700
C	0.02591600	-3.11570900	2.58000800
N	-0.64485500	-3.92360700	3.08423700
C	2.25111500	-2.23749100	2.50418500
N	3.31022300	-2.31564800	2.98215900
H	7.02185200	0.62874500	-0.39184700
H	7.83901800	-1.37737400	-1.59216200
H	6.28425300	-3.24241800	-2.14121100
H	4.65081400	0.86070600	0.23502200
H	1.50501700	-3.16371900	-0.87052100
H	-1.76208100	-1.38313600	2.46770600
H	-4.04440100	-1.04081600	1.66208800
H	-2.55934300	-0.28862000	-2.29813700
H	-0.24716900	-0.61544000	-1.50323400
H	0.28394900	1.04895600	2.02526100
H	0.20008600	3.47177800	2.05971300
H	2.76916700	3.61115200	-1.43052800
H	2.87136300	1.18511200	-1.45179300
H	3.95735800	-4.31091200	-3.09464300
H	4.39727700	-5.03426300	-1.52474400
H	2.68682200	-4.92486900	-2.00710300
N	-4.72494800	-0.43405800	-0.95490400
N	-5.68581300	-0.54521300	-0.10919700
C	-7.14658100	-0.05409000	-2.01197100
H	-6.45891900	-0.65709400	-2.60968300
H	-8.16731100	-0.38460400	-2.22027100
C	-7.99474400	-0.49567300	0.35214000
H	-8.71060000	0.30721300	0.14557900
H	-7.57747300	-0.32558400	1.34681900
C	-6.98057000	1.43405400	-2.33632400
H	-7.66697800	2.04716600	-1.74291800
H	-5.95669700	1.76092700	-2.13758000
H	-7.19978100	1.60399900	-3.39588200
C	-8.67810200	-1.86349400	0.27886200
H	-9.50037400	-1.90267700	1.00121900
H	-9.09397100	-2.05549600	-0.71593000
H	-7.96785300	-2.66154300	0.51782600
N	-6.88666900	-0.35680700	-0.59725800

7b

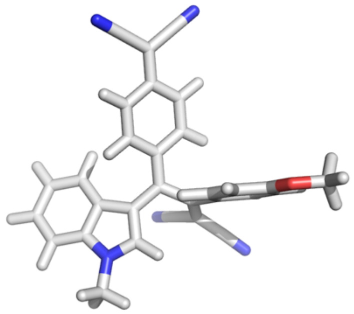

B3LYP/6-31G(d) (CPCM solvation in DCM)

Sum of electronic and zero-point Energies= -1503.113659
Sum of electronic and thermal Energies= -1503.082142
Sum of electronic and thermal Enthalpies= -1503.081198
Sum of electronic and thermal Enthalpies= -1503.179450
Imaginary Freq = 0

Atom type, (x,y,z) coordinates

C	4.69965700	2.16008100	0.04056500
C	5.72674000	1.39897300	-0.54625300
C	5.48787300	0.10291200	-0.99478900
C	4.19129300	-0.39281400	-0.85140700
C	3.12823600	0.36359400	-0.29809300
C	3.40808200	1.65689700	0.17137400
N	3.70861300	-1.66882400	-1.15175100
C	2.41521400	-1.74667900	-0.78563900
C	1.96328200	-0.51969200	-0.26623000
C	4.49507900	-2.73886600	-1.75235100
C	0.62816200	-0.31424000	0.21183800
C	-0.05067900	0.92227000	0.20613000
C	-0.09326000	-1.52634800	0.72479800
C	0.47794800	-2.23406400	1.76769700
C	0.02063100	-3.51148100	2.22259000
N	-0.28683500	-4.56343200	2.61675500
C	1.60631600	-1.73848400	2.49856600
C	-1.34471500	-1.91355300	0.07307400
C	-2.39627200	-2.55157600	0.76304300
C	-3.57278000	-2.91494100	0.12098100
C	-3.72554500	-2.65880300	-1.25247300
C	-2.69120500	-2.01327600	-1.95719200
C	-1.53430600	-1.63651000	-1.30334600
N	2.50696700	-1.37403600	3.14031500
C	-1.20288500	1.15201900	1.03517300
C	-1.86085100	2.34835300	1.04411900
C	-1.45210300	3.42921000	0.19409400
C	-0.32420600	3.19694100	-0.66271600
C	0.34794100	2.00886700	-0.64552600
C	-2.13872500	4.66137000	0.18753000
C	-1.74253200	5.73379600	-0.65383900
N	-1.41310100	6.61343000	-1.34783000
C	-3.26478400	4.89448400	1.01980400
N	-4.19094000	5.08206300	1.70602700
H	4.91728300	3.16191400	0.39872500
H	6.72211600	1.82227000	-0.64102700
H	6.28139800	-0.49947500	-1.42489100
H	2.63514300	2.26310800	0.63026500
H	1.85875800	-2.66039000	-0.94117800
H	5.34052900	-2.99059300	-1.10563200
H	4.87007700	-2.42518500	-2.73051700
H	3.86121900	-3.61739600	-1.87385800
H	-2.31908200	-2.72964600	1.82925100
H	-4.36559100	-3.38039900	0.69353400
H	-2.82537500	-1.81683000	-3.01577400
H	-0.75017100	-1.14028000	-1.86536000
H	-1.53773200	0.36806300	1.70549700
H	-2.70857200	2.49123500	1.70637200
H	-0.01642300	3.97844900	-1.35002600
H	1.17156400	1.86082400	-1.33368100
O	-4.81883500	-2.97700800	-1.97512100
C	-5.91223400	-3.63426900	-1.32516000
H	-6.33237400	-3.00684300	-0.53178900
H	-6.66076600	-3.79335100	-2.10132100
H	-5.60048200	-4.59884300	-0.91040400

7c

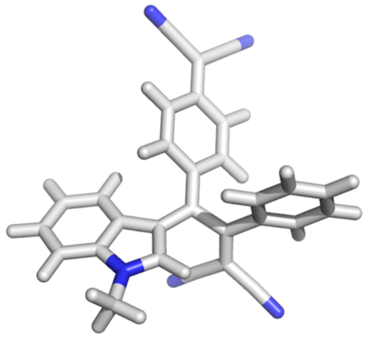

B3LYP/6-31G(d) (CPCM solvation in DCM)

Sum of electronic and zero-point Energies= -1388.618520
Sum of electronic and thermal Energies= -1388.589647
Sum of electronic and thermal Enthalpies= -1388.588703
Sum of electronic and thermal Enthalpies= -1388.680819
Imaginary Freq = 0

Atom type, (x,y,z) coordinates

C	-1.63012800	-4.38266900	0.62981000
C	-2.90101000	-4.67372700	0.10184800
C	-3.67313400	-3.67867100	-0.49118600
C	-3.12500100	-2.39678400	-0.55121100
C	-1.83349400	-2.08190000	-0.06015800
C	-1.09118200	-3.10101700	0.55794500
N	-3.70925900	-1.22095100	-1.02773000
C	-2.85870800	-0.19612700	-0.82935200
C	-1.65606400	-0.64358300	-0.25196900
C	-5.03466100	-1.13098900	-1.62767200
C	-0.56770800	0.22926400	0.07925900
C	0.79115900	-0.15061100	0.09779500
C	-0.91934600	1.65113900	0.38976200
C	-1.84048900	1.91172200	1.38185000
C	-2.39811000	3.20632700	1.64035700
N	-2.90847000	4.22424600	1.88188300
C	-2.33124000	0.89294400	2.26470500
C	-0.29312700	2.71725300	-0.41491400
C	0.07629700	3.95692600	0.14393300
C	0.66046600	4.94089600	-0.64879600
C	0.87524800	4.71200900	-2.01062800
C	0.51905700	3.48464100	-2.57601600
C	-0.04610500	2.48942800	-1.78429000
N	-2.74535300	0.11663200	3.02649600
C	1.77790900	0.61663400	0.80842300
C	3.09247600	0.24975700	0.84288500
C	3.56076900	-0.90818100	0.13702100
C	2.58830900	-1.66064300	-0.60378800
C	1.27016400	-1.30604800	-0.60941600
C	4.92027300	-1.28226100	0.15618000
C	5.39221800	-2.42258500	-0.54551200
N	5.77651900	-3.36096400	-1.12469900
C	5.89119300	-0.53762300	0.87614000
N	6.68681100	0.07737700	1.46978500
H	-1.05604500	-5.17375100	1.10299500
H	-3.29099700	-5.68497300	0.16674000
H	-4.66484800	-3.89089500	-0.87722100
H	-0.11017800	-2.90225400	0.97473700
H	-3.12933800	0.80378300	-1.13841100
H	-5.79373400	-1.45358000	-0.90923600
H	-5.08710600	-1.76493000	-2.51711900
H	-5.22484300	-0.09563900	-1.91089600
H	-0.05482900	4.13716900	1.20505300
H	0.95654300	5.88419700	-0.20016500
H	1.32821500	5.48346200	-2.62637900
H	0.68772800	3.29988000	-3.63251600
H	-0.31663600	1.53725700	-2.22913600
H	1.46678800	1.48982700	1.37128900
H	3.80082500	0.83965400	1.41549900
H	2.91679300	-2.51674500	-1.18435400
H	0.57372700	-1.87996700	-1.20904300

7d

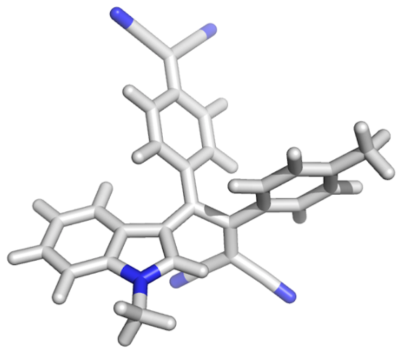

B3LYP/6-31G(d) (CPCM solvation in DCM)

Sum of electronic and zero-point Energies= -1427.910578
Sum of electronic and thermal Energies= -1427.879815
Sum of electronic and thermal Enthalpies= -1427.878871
Sum of electronic and thermal Enthalpies= -1427.975686
Imaginary Freq = 0

Atom type, (x,y,z) coordinates

C	-2.52123700	-4.23811200	0.25495100
C	-3.80493200	-4.23620600	-0.32020600
C	-4.35964200	-3.06350000	-0.82518400
C	-3.58296700	-1.90651500	-0.74992500
C	-2.27276900	-1.88590500	-0.21007600
C	-1.75306400	-3.07874300	0.31784600
N	-3.92383500	-0.60256400	-1.11707200
C	-2.90544100	0.22179400	-0.80626700
C	-1.82605300	-0.49473400	-0.25760500
C	-5.18797500	-0.21013000	-1.72735200
C	-0.60651000	0.12416900	0.17322000
C	0.65728400	-0.50297800	0.17444700
C	-0.70014500	1.55151800	0.61952600
C	-1.58158000	1.87412900	1.63178000
C	-1.91234700	3.21447600	2.01371300
N	-2.24441400	4.27824600	2.35073700
C	-2.25883000	0.87645300	2.40792400
C	0.12342600	2.55453600	-0.07287300
C	0.67152300	3.67165800	0.59086700
C	1.43822000	4.59998600	-0.10166400
C	1.67870700	4.46608400	-1.47895400
C	1.14273100	3.34839700	-2.13568400
C	0.39217500	2.40166100	-1.44845600
N	-2.81614800	0.11261700	3.08681400
C	1.74652200	0.00091300	0.96636100
C	2.96878800	-0.60700900	0.98569400
C	3.23625000	-1.76598300	0.18358100
C	2.16566500	-2.25393300	-0.63883500
C	0.93684900	-1.65954600	-0.63113000
C	4.50112000	-2.38964500	0.18960900
C	4.77477500	-3.53391200	-0.60474600
N	4.99592600	-4.47505800	-1.25979700
C	5.56972900	-1.90632200	0.98954200
N	6.44616400	-1.50585500	1.64922800
H	-2.11958600	-5.16307300	0.65800600
H	-4.37656400	-5.15841400	-0.36136600
H	-5.35936000	-3.05029100	-1.24702100
H	-0.76750100	-3.10609100	0.76892900
H	-2.97659900	1.27891000	-1.02101100
H	-6.01917000	-0.47355400	-1.06712400
H	-5.31541300	-0.71732300	-2.68783900
H	-5.18398100	0.86834300	-1.88631700
H	0.53734700	3.79181200	1.66007600
H	1.86865400	5.43950000	0.43762400
H	1.32230200	3.21473600	-3.19897900
H	-0.00598500	1.54591400	-1.98407200
H	1.58449200	0.86502900	1.60127700
H	3.75601600	-0.21266100	1.62003900
H	2.34854300	-3.10121300	-1.29192200
H	0.16603400	-2.03677000	-1.29252500
C	2.48274600	5.49925200	-2.22611300
H	1.86114300	6.37092300	-2.46987400
H	3.32325900	5.86128200	-1.62489400
H	2.87440200	5.10013300	-3.16630700

7e

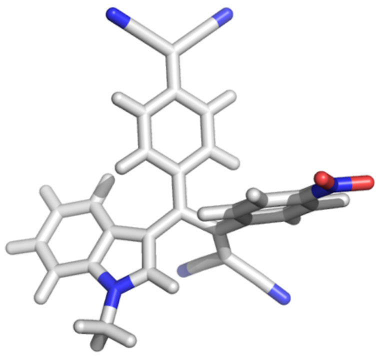

B3LYP/6-31G(d) (CPCM solvation in DCM)

Sum of electronic and zero-point Energies= -1593.115705
Sum of electronic and thermal Energies= -1593.084222
Sum of electronic and thermal Enthalpies= -1593.083278
Sum of electronic and thermal Enthalpies= -1593.182000
Imaginary Freq = 0

Atom type, (x,y,z) coordinates

C	5.03666000	1.67800700	-0.02832100
C	5.96156900	0.82303500	-0.65481000
C	5.57868600	-0.43758400	-1.10464600
C	4.24404500	-0.80132700	-0.92155900
C	3.28257100	0.05196300	-0.32517400
C	3.70612500	1.30618200	0.14338500
N	3.62294200	-2.01627800	-1.22048800
C	2.34054300	-1.96588200	-0.81451500
C	2.03572400	-0.70741500	-0.26303800
C	4.27463600	-3.15266600	-1.86011300
C	0.74367600	-0.36083300	0.25653400
C	0.20333700	0.94335700	0.25372000
C	-0.08395000	-1.47979600	0.79691900
C	0.44485300	-2.33391500	1.73773500
C	-0.19662600	-3.54013300	2.17273500
N	-0.65820800	-4.54096800	2.54598000
C	1.70186800	-2.09001500	2.38619000
C	-1.44792800	-1.67285000	0.24908000
C	-2.53301800	-2.04191800	1.06651700
C	-3.79843300	-2.22696500	0.52195800
C	-3.96993400	-2.04768300	-0.84905700
C	-2.92061900	-1.67437100	-1.68660100
C	-1.66371500	-1.47588700	-1.12886700
N	2.69578500	-1.93572800	2.97089000
C	-0.87439500	1.31549700	1.12959600
C	-1.39283600	2.57814500	1.14285500
C	-0.90368700	3.59222700	0.25304400
C	0.15130900	3.22066500	-0.64720400
C	0.68495700	1.96467400	-0.63522000
C	-1.44595700	4.89288500	0.25164200
C	-0.97163900	5.90039600	-0.62949100
N	-0.57903100	6.72626300	-1.35544900
C	-2.49734900	5.26656500	1.12994200
N	-3.36143500	5.57007900	1.85408000
H	5.36626100	2.64925800	0.32843300
H	6.99088500	1.14495500	-0.78040100
H	6.29289900	-1.11157500	-1.56631300
H	3.01362600	1.98307600	0.63120400
H	1.68907700	-2.81527000	-0.96522400
H	5.11325400	-3.49562100	-1.24760100
H	4.64323700	-2.86584800	-2.84888000
H	3.55118800	-3.96131300	-1.96527100
H	-2.40168500	-2.15291000	2.13649000
H	-4.64170600	-2.49766400	1.14415300
H	-3.09248900	-1.54066900	-2.74682200
H	-0.83938400	-1.18325600	-1.76970300
H	-1.25942700	0.58868700	1.83648100
H	-2.18630500	2.82669300	1.84010200
H	0.51418200	3.95189000	-1.36229200
H	1.45666400	1.71378800	-1.35312300
N	-5.30565300	-2.24914200	-1.43009400
O	-5.43981100	-2.06936100	-2.64071200
O	-6.21692900	-2.58813300	-0.67492400

7f

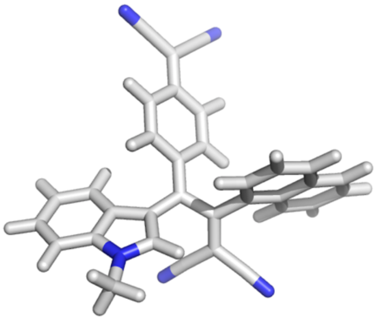

B3LYP/6-31G(d) (CPCM solvation in DCM)

Sum of electronic and zero-point Energies= -1542.210755
Sum of electronic and thermal Energies= -1542.179274
Sum of electronic and thermal Enthalpies= -1542.178330
Sum of electronic and thermal Enthalpies= -1542.275669
Imaginary Freq = 0

Atom type, (x,y,z) coordinates

C	4.87620700	1.56578400	1.08254500
C	5.93554500	0.79140700	0.57468800
C	5.68808300	-0.36734800	-0.15619700
C	4.35302800	-0.71240400	-0.37123900
C	3.26644200	0.06463400	0.10148500
C	3.54842000	1.21469300	0.85643200
N	3.83487500	-1.84050200	-1.01156000
C	2.49046400	-1.81287000	-0.93040400
C	2.04989800	-0.65287800	-0.26889600
C	0.66581500	-0.34675200	-0.02871100
C	0.15189400	0.96928000	0.00073700
C	-0.26142400	-1.49893900	0.14957500
C	0.09610000	-2.54625600	0.97759700
C	-1.09695400	1.28424900	0.63909900
C	-1.58376000	2.55884900	0.68734500
C	-0.88250800	3.64742700	0.06987600
C	0.34989300	3.33742600	-0.59769800
C	0.84527900	2.06559900	-0.61727900
C	-1.38957500	4.96249300	0.10265700
C	-1.50214900	-1.53890300	-0.65954100
C	-2.80348100	-1.84302300	-0.11635900
C	-3.90949900	-1.94865400	-1.02708500
C	-3.70264400	-1.74311200	-2.41649300
C	-2.46027400	-1.40570800	-2.90431500
C	-1.36952600	-1.28713300	-2.02161700
C	-3.07310300	-1.97310800	1.27341400
C	-4.34701600	-2.23299200	1.73249700
C	-5.42486000	-2.37964100	0.82915200
C	-5.20720100	-2.23334500	-0.52147200
C	4.63409100	-2.87707000	-1.65279200
C	-0.60265600	-3.79593700	0.99718400
N	-1.11578700	-4.84032100	1.02568700
C	1.19550200	-2.48928400	1.89634500
N	2.04729600	-2.50009300	2.68981000
C	-0.70535300	6.04404300	-0.51201800
N	-0.13969300	6.93134900	-1.01815600
C	-2.61332600	5.27687400	0.75038000
N	-3.62002600	5.53162600	1.28428100
H	5.09843100	2.45701200	1.66191400
H	6.96088200	1.09540400	0.76246400
H	6.50086100	-0.98073900	-0.53132100
H	2.74900500	1.82775200	1.25792000
H	1.90163600	-2.60154500	-1.37743000
H	-1.65344600	0.49903900	1.13761000
H	-2.51666100	2.75874200	1.20442500
H	0.88399300	4.13026800	-1.11149300
H	1.76270900	1.86678300	-1.15840400
H	-4.55081400	-1.83919900	-3.08913700
H	-2.31087700	-1.23304000	-3.96539400
H	-0.39415000	-1.02937000	-2.42239600
H	-2.27045600	-1.85470400	1.99299400
H	-4.52329200	-2.32251200	2.80037900
H	-6.42120800	-2.59201500	1.20511400
H	-6.02993000	-2.31875900	-1.22640300
H	5.29980300	-3.34260700	-0.92038000
H	3.96665900	-3.63444100	-2.06429100
H	5.23207700	-2.44489000	-2.45997100

7g

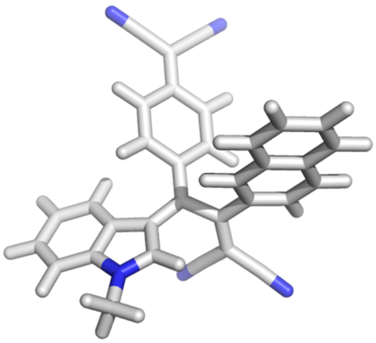

B3LYP/6-31G(d) (CPCM solvation in DCM)

Sum of electronic and zero-point Energies= -1542.217047
Sum of electronic and thermal Energies= -1542.185528
Sum of electronic and thermal Enthalpies= -1542.184584
Sum of electronic and thermal Enthalpies= -1542.282697
Imaginary Freq = 0

Atom type, (x,y,z) coordinates

C	-4.84284800	-2.07310300	-0.46948600
C	-5.77149700	-1.23801000	-1.11663700
C	-5.45027900	0.08009800	-1.42884000
C	-4.17143700	0.52258100	-1.08813200
C	-3.20264100	-0.30635200	-0.46925300
C	-3.56740100	-1.62215800	-0.14077400
N	-3.62668300	1.80110900	-1.22764500
C	-2.38721500	1.81009900	-0.70171900
C	-2.03103100	0.53426200	-0.22764800
C	-4.30612400	2.93705600	-1.83766000
C	-0.76602800	0.25047600	0.38465100
C	-0.11589000	-1.00075100	0.35541700
C	-0.08627200	1.39486500	1.07485400
C	-0.75754800	2.04709200	2.08961700
C	1.25197700	1.79316400	0.61102700
C	2.24312200	2.31458100	1.50041500
C	3.47463500	2.69460300	1.02991400
C	3.80107700	2.59704100	-0.35151600
C	2.82034700	2.06467100	-1.25161100
C	1.56642400	1.65869600	-0.73857900
C	5.06188600	2.99347000	-0.86625600
C	5.33813900	2.87283100	-2.21178400
C	4.36846900	2.34863400	-3.10327400
C	3.13620500	1.95266900	-2.63435000
C	-0.43816100	-2.00722000	-0.61827400
C	0.20674100	-3.20975700	-0.65380400
C	1.22570000	-3.53907500	0.30227500
C	1.55878700	-2.53796600	1.27460700
C	0.93034400	-1.32616400	1.28683800
C	1.88273100	-4.78647700	0.27570800
C	-0.31619400	3.26675600	2.69687100
N	-0.02622500	4.27174600	3.20810900
C	-1.99029400	1.55417400	2.63117700
N	-2.97812600	1.18757400	3.12586700
C	2.90099100	-5.11570000	1.20862200
N	3.73813500	-5.38266300	1.97774500
C	1.56394000	-5.77952300	-0.68742000
N	1.29901200	-6.59341100	-1.48183700
H	-5.12557800	-3.09172300	-0.22085500
H	-6.75607700	-1.62159200	-1.36647800
H	-6.17061500	0.73754700	-1.90477300
H	-2.87192900	-2.28569700	0.36091500
H	-1.79526000	2.71444100	-0.71899400
H	-5.22398400	3.16368400	-1.28772800
H	-3.64442300	3.80280400	-1.80546900
H	-4.55551300	2.71049700	-2.87796800
H	2.03927900	2.37079200	2.56319200
H	4.22572300	3.06762600	1.72084600
H	0.83296800	1.25557000	-1.43028800
H	5.80643400	3.39411300	-0.18353400
H	6.30620000	3.18095200	-2.59620400
H	4.60364300	2.26044300	-4.15980600
H	2.38933800	1.54864600	-3.31262600
H	-1.17591800	-1.78321800	-1.37929800
H	-0.03768000	-3.92784600	-1.42997800
H	2.32394800	-2.75501000	2.01283200
H	1.20272200	-0.60589300	2.05068600

7h

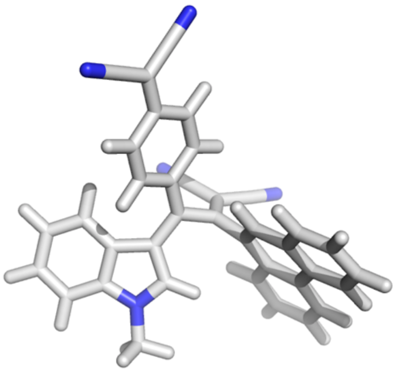

B3LYP/6-31G(d) (CPCM solvation in DCM)

Sum of electronic and zero-point Energies= -1695.806827
Sum of electronic and thermal Energies= -1695.772491
Sum of electronic and thermal Enthalpies= -1695.771547
Sum of electronic and thermal Enthalpies= -1695.875352
Imaginary Freq = 0

Atom type, (x,y,z) coordinates

C	-3.21215600	4.44784700	-0.54570100
C	-2.47436300	5.46330200	-1.18213200
C	-1.13035600	5.28132400	-1.49300400
C	-0.55871700	4.05073200	-1.16282400
C	-1.28827900	2.99552100	-0.55940700
C	-2.63442600	3.22217900	-0.22837300
N	0.76551800	3.63868700	-1.30372200
C	0.89749800	2.39551000	-0.79454600
C	-0.33651500	1.90772200	-0.33641000
C	1.82811000	4.43089700	-1.90936100
C	-0.51617800	0.63200700	0.30179900
C	-1.67750100	-0.15509200	0.25066500
C	0.67383300	0.14416600	1.08459100
C	0.69225800	0.36014200	2.44073300
C	1.73796500	-0.56816300	0.34524800
C	3.15983500	-0.27646100	0.50093900
C	4.11542300	-1.11570700	-0.15499100
C	3.66709500	-2.23293800	-0.97731600
C	2.26877800	-2.42170800	-1.17519300
C	1.34083300	-1.56222300	-0.51903400
C	4.54954800	-3.13517900	-1.61460000
C	4.07213700	-4.16006600	-2.41409200
C	2.68865600	-4.33331200	-2.61533500
C	1.80101700	-3.47452300	-1.99975800
C	3.62517400	0.85621100	1.21152300
C	4.97448500	1.13491900	1.32408300
C	5.91582700	0.28577400	0.72009300
C	5.48858400	-0.80809100	-0.00928200
C	-2.69598000	0.06969300	-0.74495800
C	-3.80631600	-0.71551200	-0.83435000
C	-4.02558200	-1.80887000	0.07192100
C	-3.00213900	-2.06285800	1.04672900
C	-1.88303000	-1.28723400	1.12056800
C	-5.18115300	-2.60805800	-0.00676200
C	1.67512500	-0.19609100	3.32263500
N	2.43151300	-0.65431200	4.07847300
C	-0.33933500	1.11567100	3.09013000
N	-1.15701200	1.73330100	3.64147700
C	-5.39702600	-3.70125500	0.87424100
N	-5.57143900	-4.59910000	1.59979400
C	-6.19440800	-2.36621400	-0.97238100
N	-7.02458100	-2.16556400	-1.76838100
H	-4.25437900	4.62334500	-0.29609300
H	-2.95578400	6.40620400	-1.42328300
H	-0.54916600	6.06942000	-1.96080400
H	-3.22316000	2.45919600	0.26865900
H	1.85669600	1.89965000	-0.82473500
H	1.58674100	4.65096300	-2.95317300
H	1.95292800	5.37005700	-1.36275900
H	2.75889300	3.86494700	-1.86649900
H	0.28455000	-1.75442600	-0.67335300
H	5.62090200	-3.03918800	-1.48259600
H	4.77509800	-4.83797400	-2.88957200
H	2.32615600	-5.13985900	-3.24537500
H	0.72958800	-3.59675700	-2.13407800
H	2.91238600	1.53502300	1.66530400
H	5.30048400	2.01246300	1.87423200
H	6.97758300	0.49592500	0.80835800
H	6.23236300	-1.42937500	-0.49399000
H	-2.55095900	0.85829600	-1.47288200
H	-4.53482600	-0.52738700	-1.61646600
H	-3.12648500	-2.89172800	1.73590400
H	-1.13992500	-1.52425600	1.87317900

7i

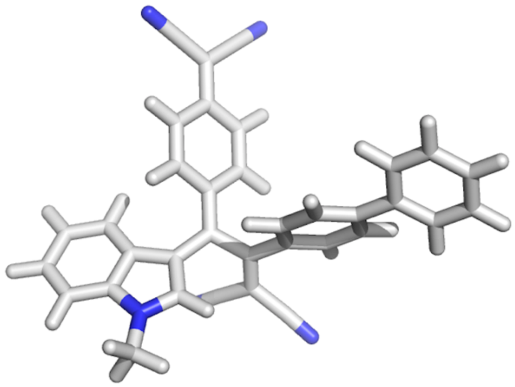

B3LYP/6-31G(d) (CPCM solvation in DCM)

Sum of electronic and zero-point Energies= -1619.597057
Sum of electronic and thermal Energies= -1619.563474
Sum of electronic and thermal Enthalpies= -1619.562530
Sum of electronic and thermal Enthalpies= -1619.665850
Imaginary Freq = 0

Atom type, (x,y,z) coordinates

C	-3.21215600	4.44784700	-0.54570100
C	5.83731000	0.27703500	-0.63878900
C	6.39849000	-0.81709900	-1.32182700
C	5.62725100	-1.93082300	-1.64266400
C	4.28182400	-1.90274800	-1.27321700
C	3.68094500	-0.80006400	-0.61639500
C	4.49197200	0.29605800	-0.28118400
N	3.31821100	-2.90416200	-1.41497800
C	2.16667700	-2.48818200	-0.85455200
C	2.29435300	-1.18032200	-0.35166300
C	3.54123600	-4.19123800	-2.06163700
C	1.22984700	-0.48226200	0.30794200
C	1.06773100	0.91932000	0.31227200
C	0.20775400	-1.32461200	1.00825200
C	0.62962100	-2.21911200	1.97151800
C	-0.20985600	-3.21462100	2.56801700
N	-0.83250900	-4.06196100	3.06780500
C	1.97347900	-2.23208800	2.47157000
C	-1.20029500	-1.18931600	0.60552700
C	-2.26594500	-1.32568700	1.51851700
C	-3.58240400	-1.19937400	1.09738200
C	-3.89944100	-0.94655000	-0.25110500
C	-2.83306500	-0.80339600	-1.15883000
C	-1.51400300	-0.90558500	-0.74001600
N	3.04105600	-2.27277700	2.93369300
C	0.24628400	1.57897300	1.29078400
C	0.09069300	2.93520000	1.30909700
C	0.71664900	3.76947600	0.32408800
C	1.51192200	3.11635900	-0.67678700
C	1.68801200	1.76269200	-0.67193400
C	0.54504800	5.16922400	0.32847800
C	1.15634200	6.00093900	-0.64612000
N	1.66067400	6.68154000	-1.44997600
C	-0.25069100	5.82224800	1.30615300
N	-0.90603000	6.35587600	2.11203400
H	6.46564100	1.12563900	-0.38505600
H	7.44959200	-0.79906900	-1.59350000
H	6.06021400	-2.78856000	-2.14697900
H	4.08466600	1.15035000	0.24779500
H	1.29330600	-3.12507100	-0.86721900
H	4.34272500	-4.73210000	-1.55060000
H	3.81752200	-4.04226900	-3.10923200
H	2.62326400	-4.77684100	-2.01010900
H	-2.06566500	-1.47943300	2.57282200
H	-4.37715300	-1.26385600	1.83347300
H	-3.03988000	-0.62953000	-2.20985300
H	-0.71678700	-0.79717800	-1.46813700
H	-0.23183400	0.98966700	2.06554000
H	-0.51448000	3.39692700	2.08252500
H	1.96390900	3.71395300	-1.46194200
H	2.26677300	1.30460800	-1.46510100
C	-5.30798200	-0.82360200	-0.69602500
C	-5.66520000	0.06609900	-1.72543100
C	-6.32142500	-1.59505500	-0.09867200
C	-6.99078900	0.18154700	-2.14131600
H	-4.90549900	0.69201300	-2.18431400
C	-7.64601300	-1.48192100	-0.51830300
H	-6.06743800	-2.30770500	0.68061500
C	-7.98652800	-0.59253400	-1.54041500
H	-7.24668100	0.88240500	-2.93102200
H	-8.41171600	-2.09422500	-0.05021700
H	-9.01912100	-0.50322400	-1.86583900

2. ^{1}H and ^{13}C NMR Spectra

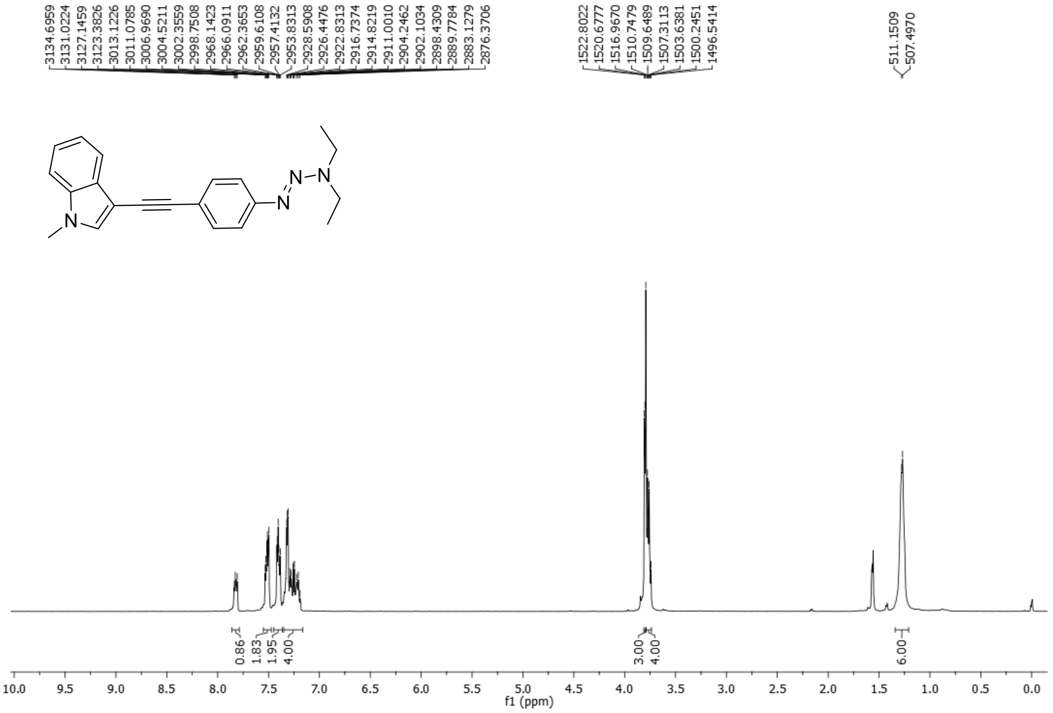

Figure S1. ^{1}H NMR spectrum of **3a** in $CDCl_3$ solution (400 MHz).

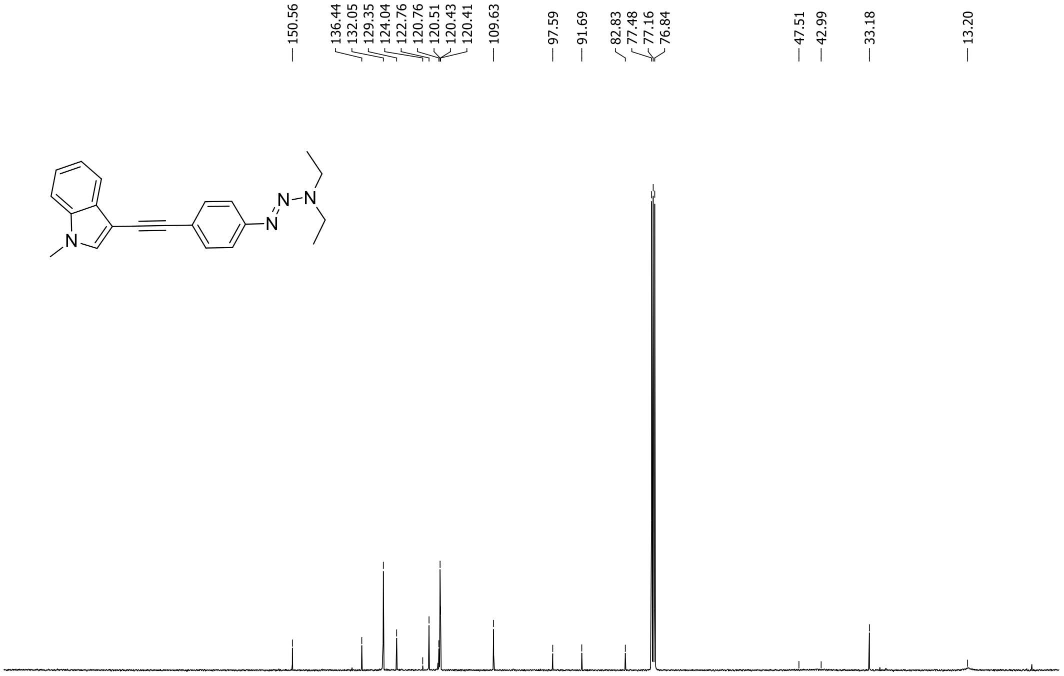

Figure S2. $^{13}C\{^{1}H\}$ NMR spectrum of **3a** in $CDCl_3$ solution (100 MHz).

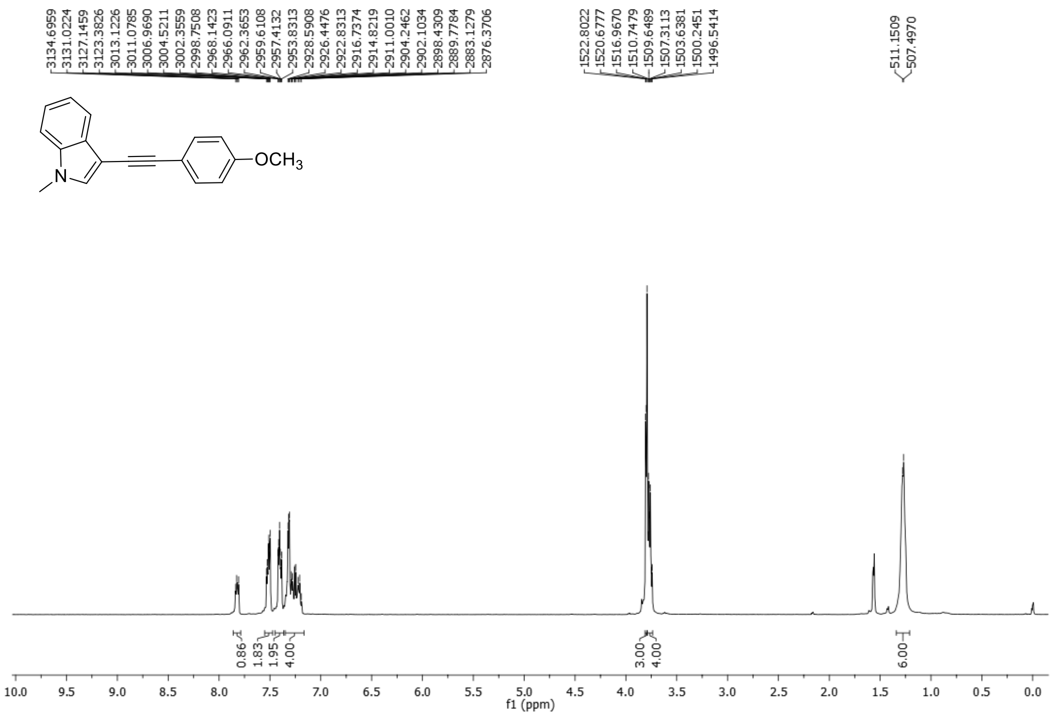

Figure S3. ^{1}H NMR spectrum of **3b** in $CDCl_3$ solution (400 MHz).

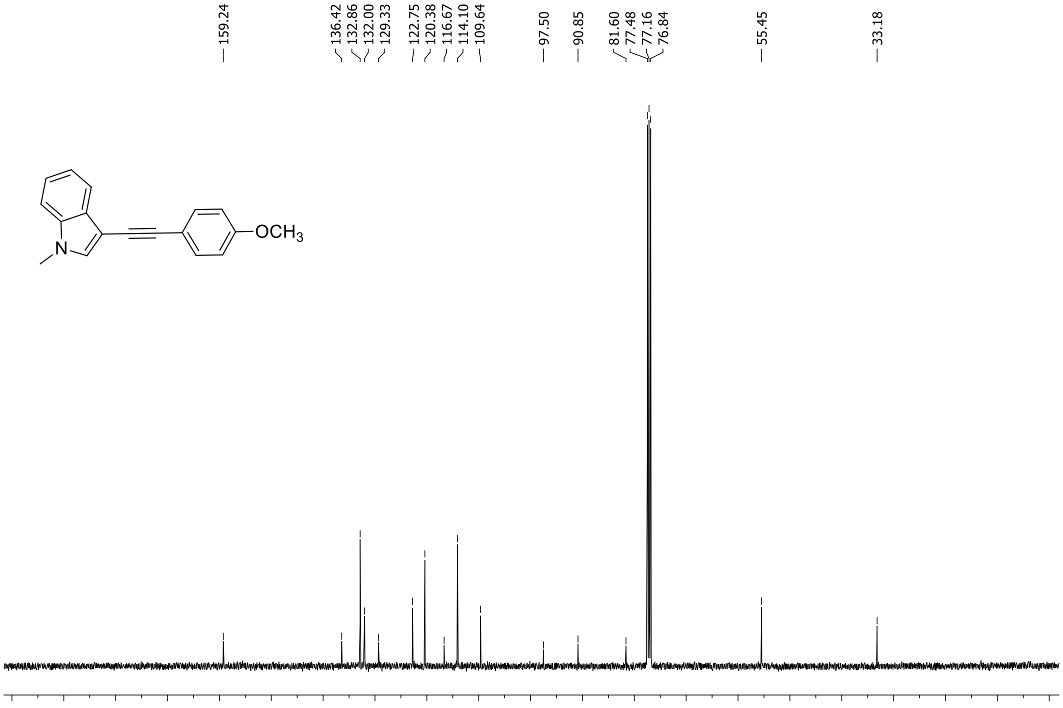

Figure S4. $^{13}C\{^{1}H\}$ NMR spectrum of **3b** in $CDCl_3$ solution (100 MHz).

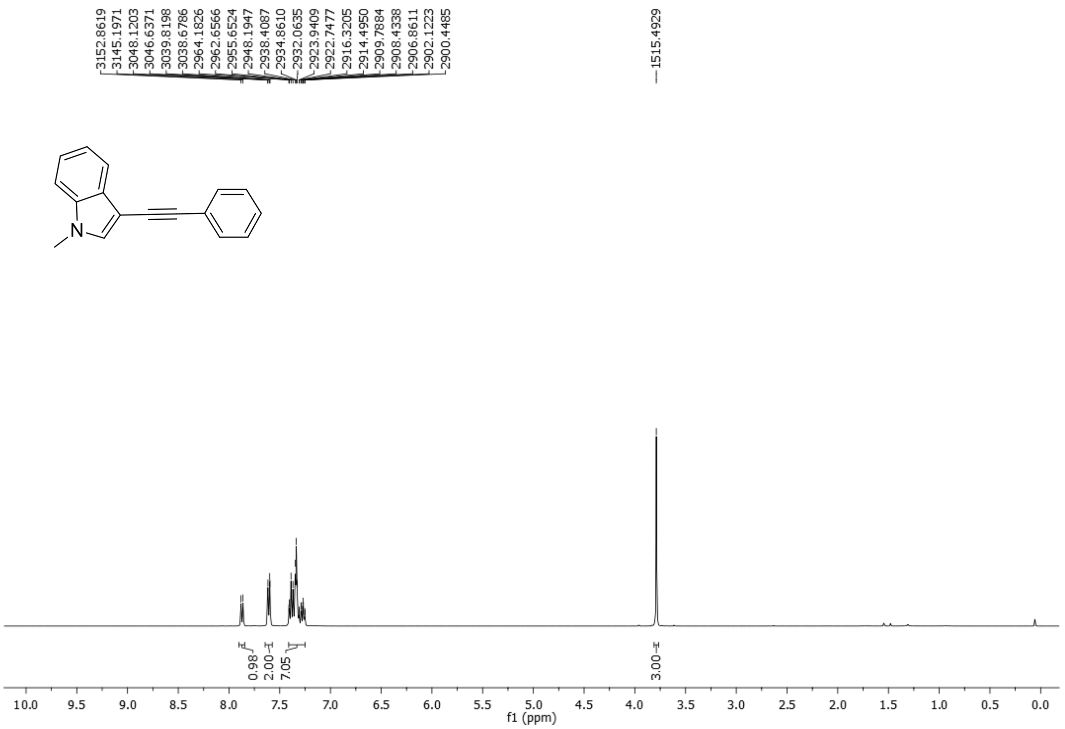

Figure S5. ^{1}H NMR spectrum of **3c** in $CDCl_3$ solution (400 MHz).

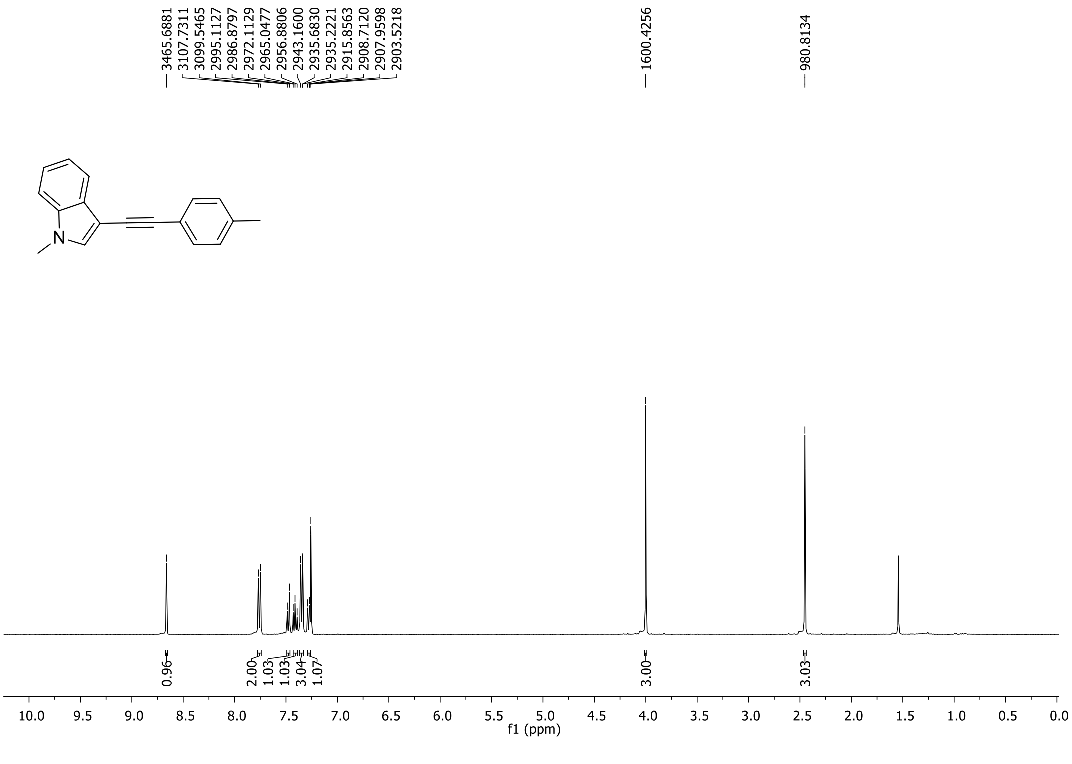

Figure S6. ^{1}H NMR spectrum of **3d** in $CDCl_3$ solution (400 MHz).

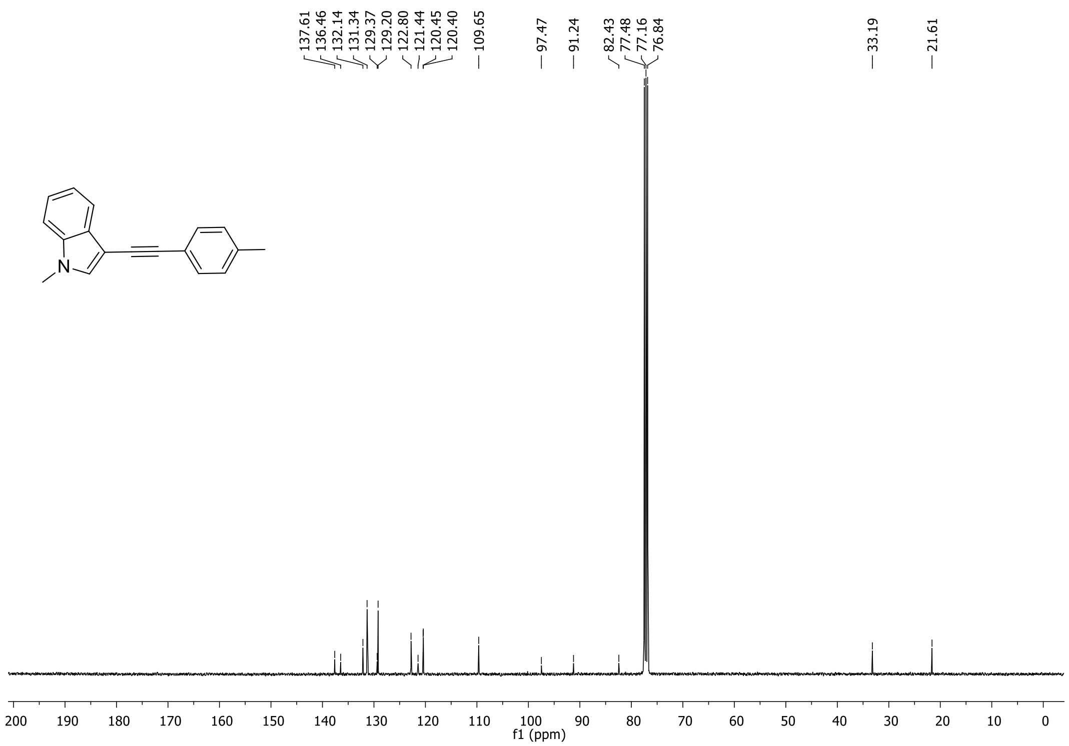

Figure S7. $^{13}C\{^1H\}$ NMR spectrum of **3d** in $CDCl_3$ solution (100 MHz).

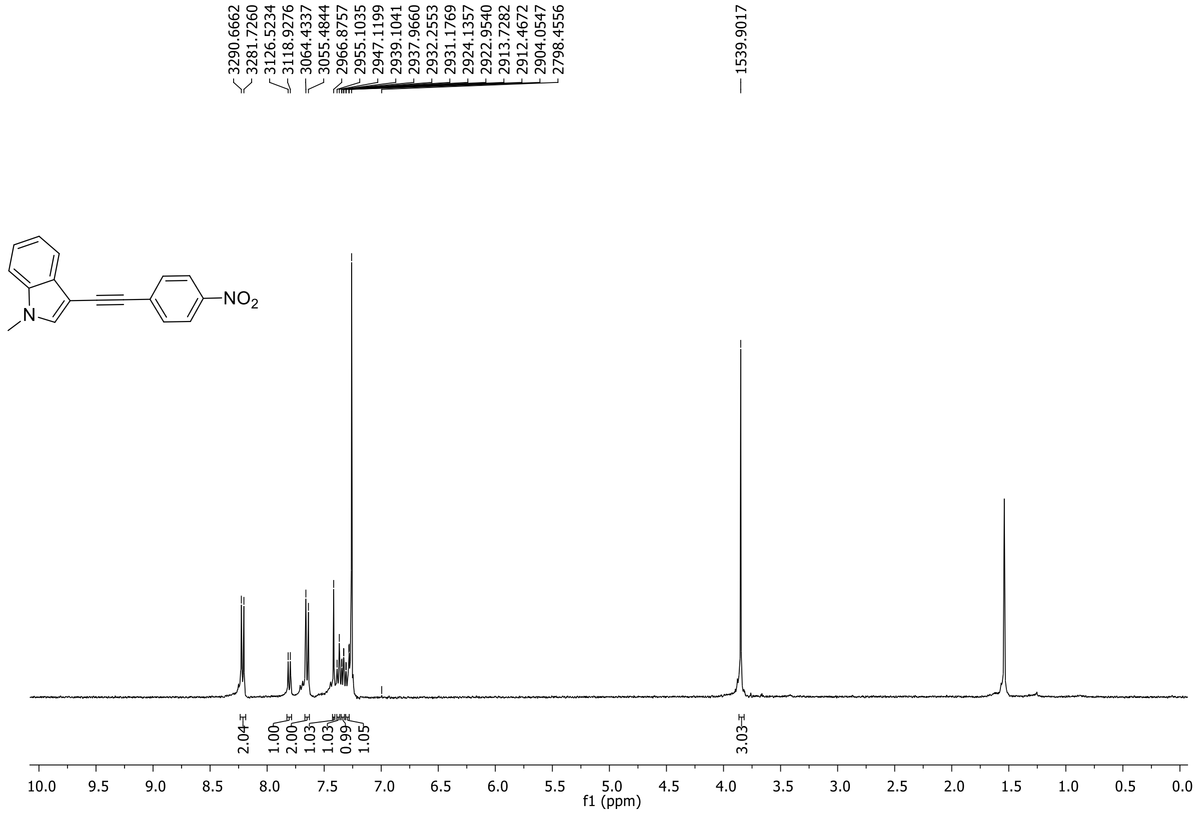

Figure S8. ^1H NMR spectrum of **3e** in $CDCl_3$ solution (400 MHz).

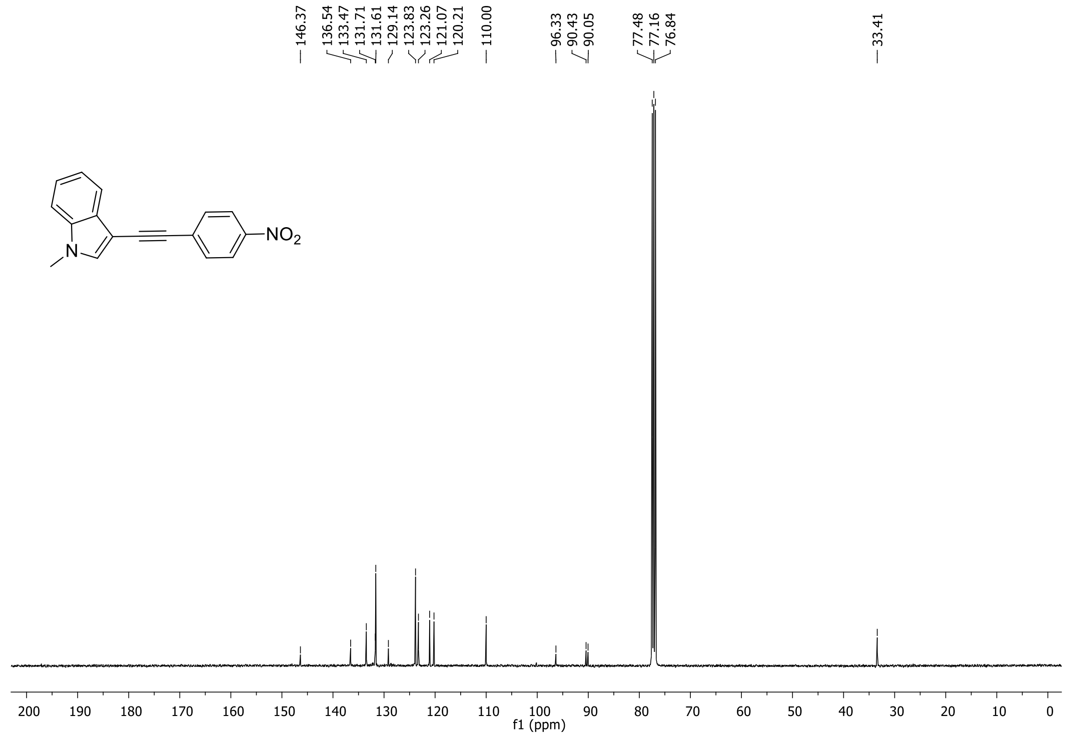

Figure S9. $^{13}C\{^{1}H\}$ NMR spectrum of **3e** in $CDCl_3$ solution (100 MHz).

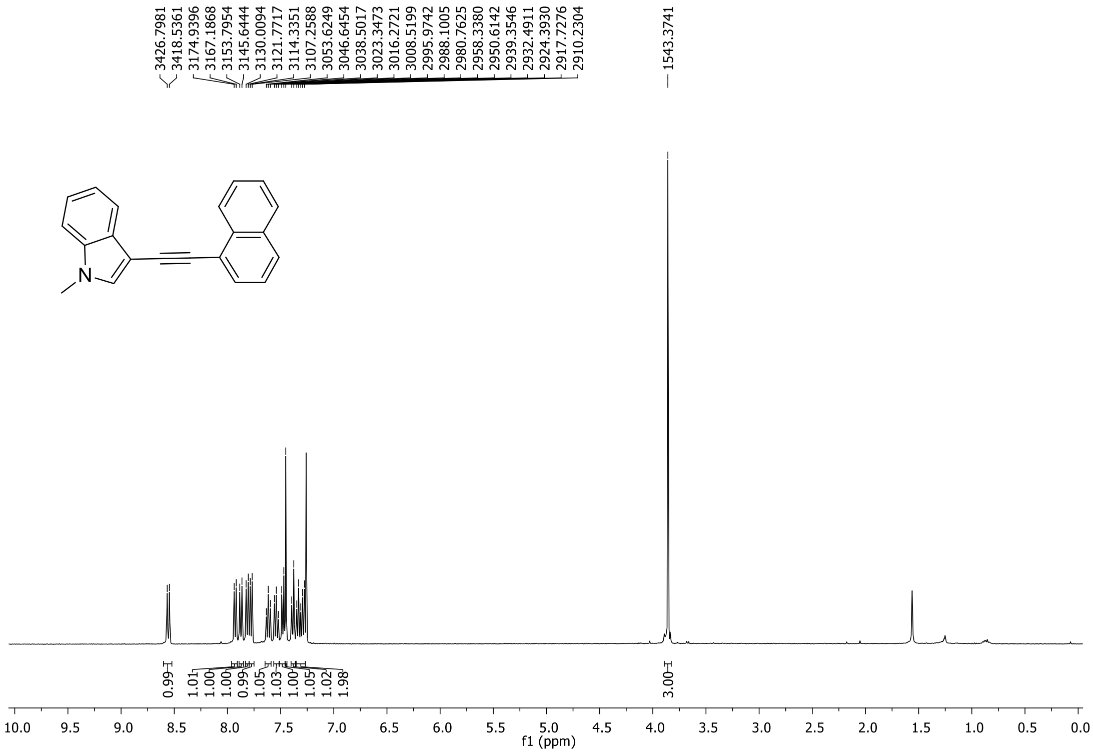

Figure S10. ^{1}H NMR spectrum of **3f** in $CDCl_3$ solution (400 MHz).

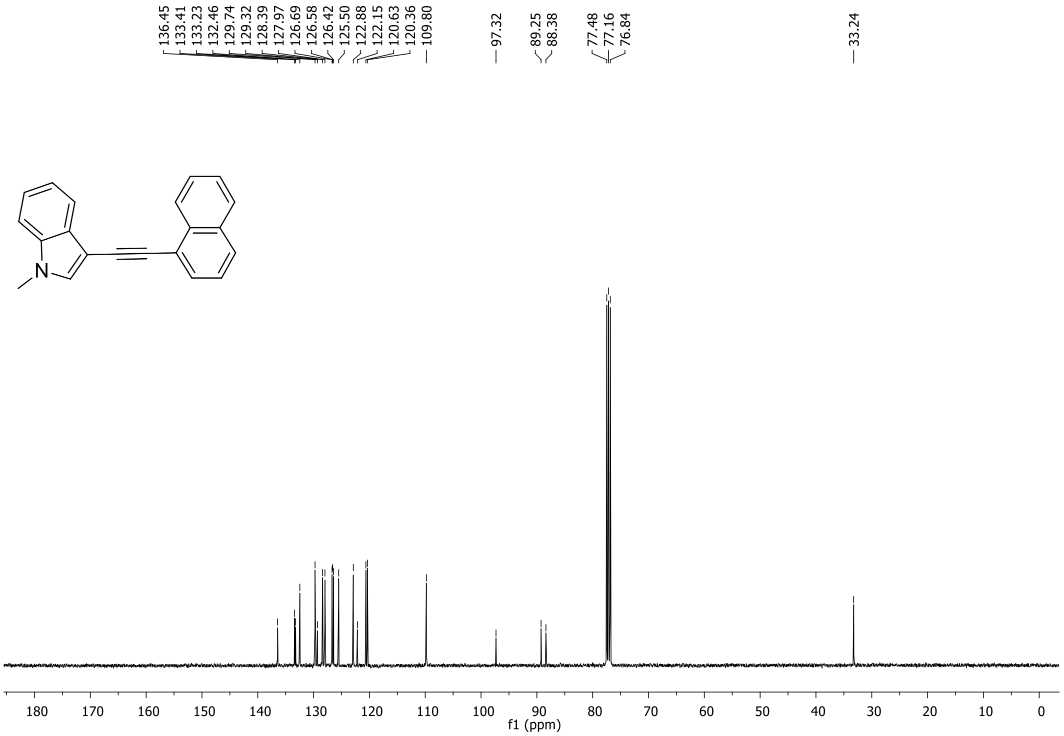

Figure S11. $^{13}C\{^{1}H\}$ NMR spectrum of **3f** in $CDCl_3$ solution (100 MHz).

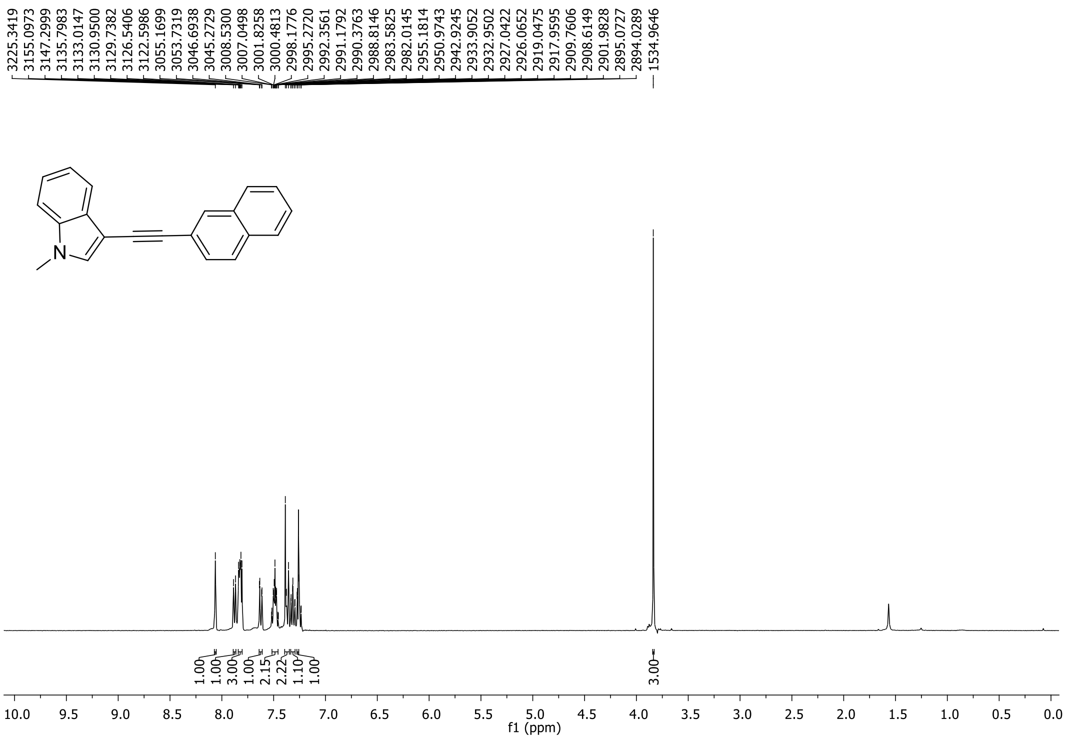

Figure S12. ^{1}H NMR spectrum of **3g** in $CDCl_3$ solution (400 MHz).

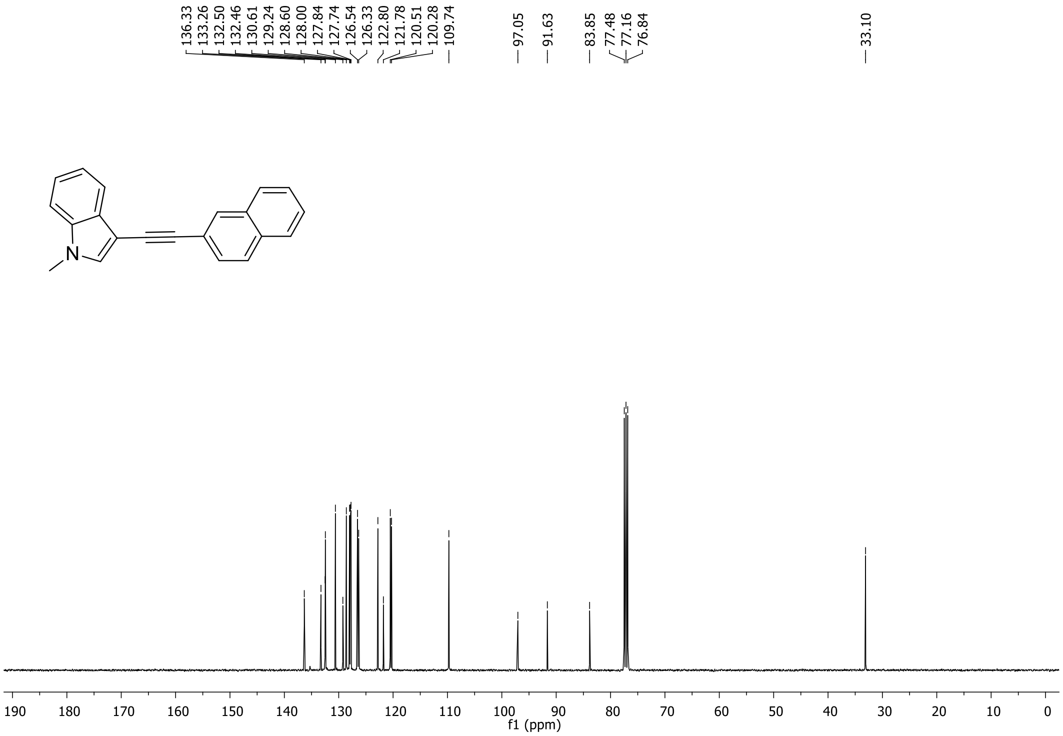

Figure S13. $^{13}C\{^{1}H\}$ NMR spectrum of **3g** in $CDCl_3$ solution (100 MHz).

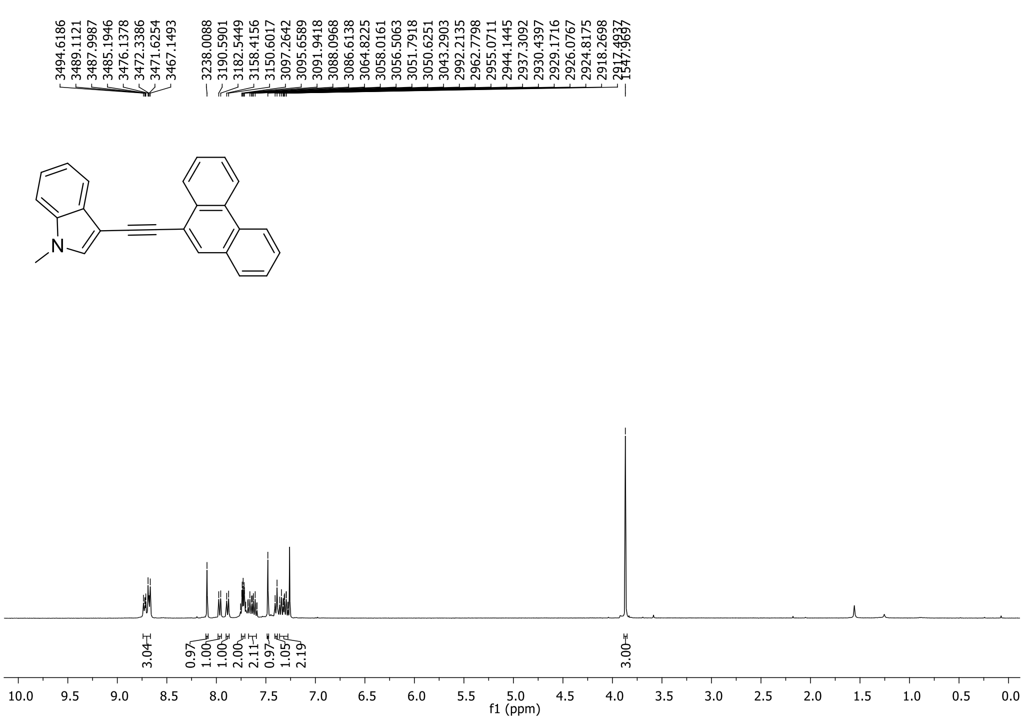

Figure S14. ^{1}H NMR spectrum of **3h** in $CDCl_3$ solution (400 MHz).

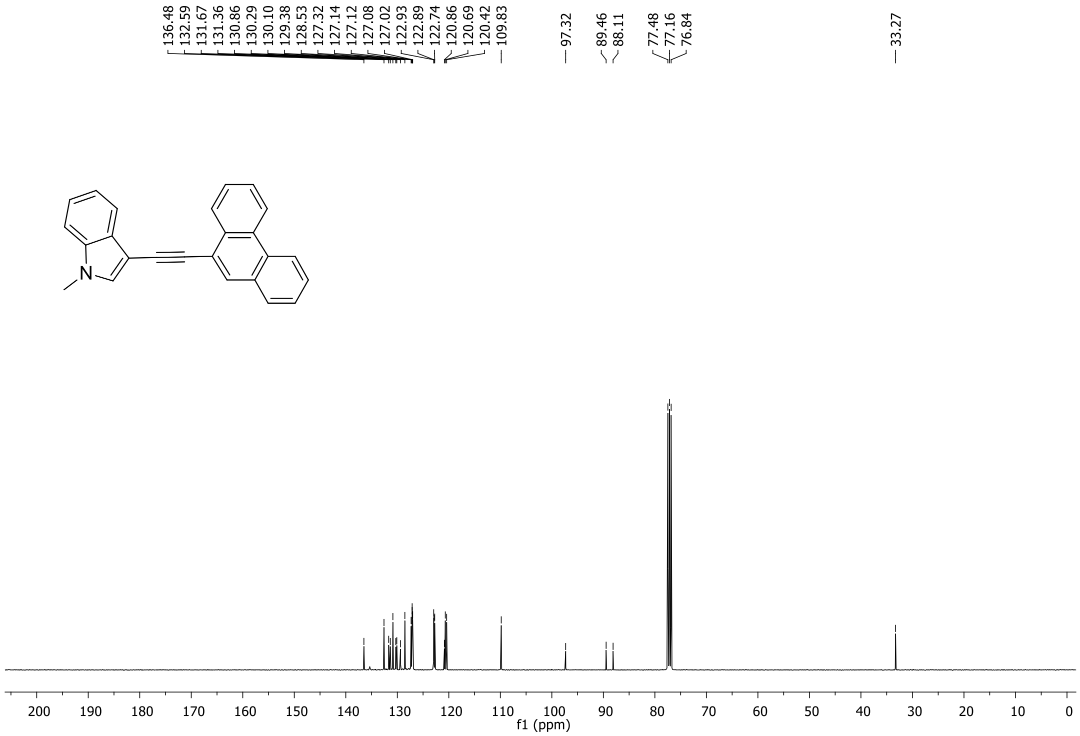

Figure S15. $^{13}C\{^{1}H\}$ NMR spectrum of **3h** in $CDCl_3$ solution (100 MHz).

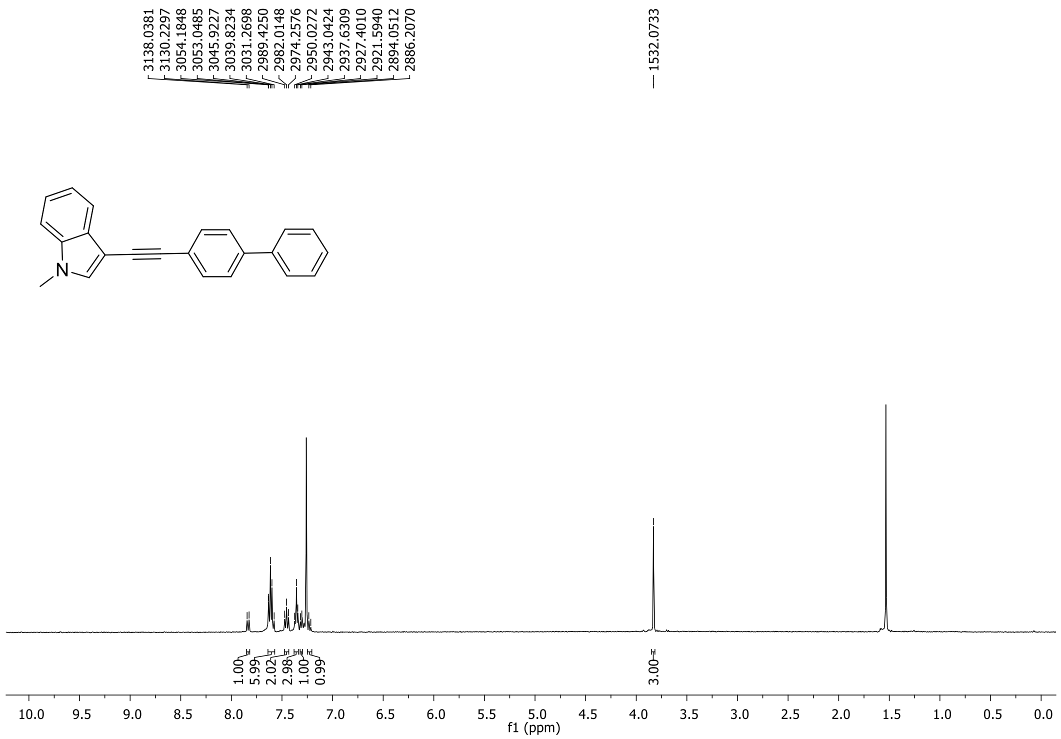

Figure S16. ^{1}H NMR spectrum of **3i** in $CDCl_3$ solution (400 MHz).

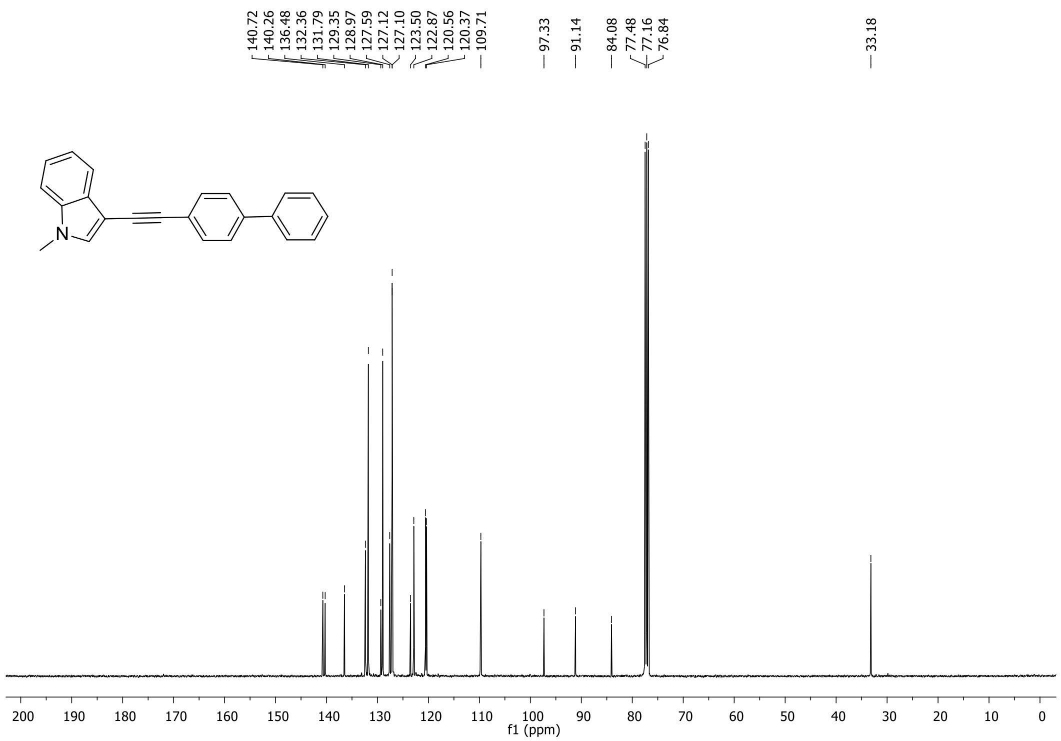

Figure S17. $^{13}C\{^{1}H\}$ NMR spectrum of **3i** in $CDCl_3$ solution (100 MHz).

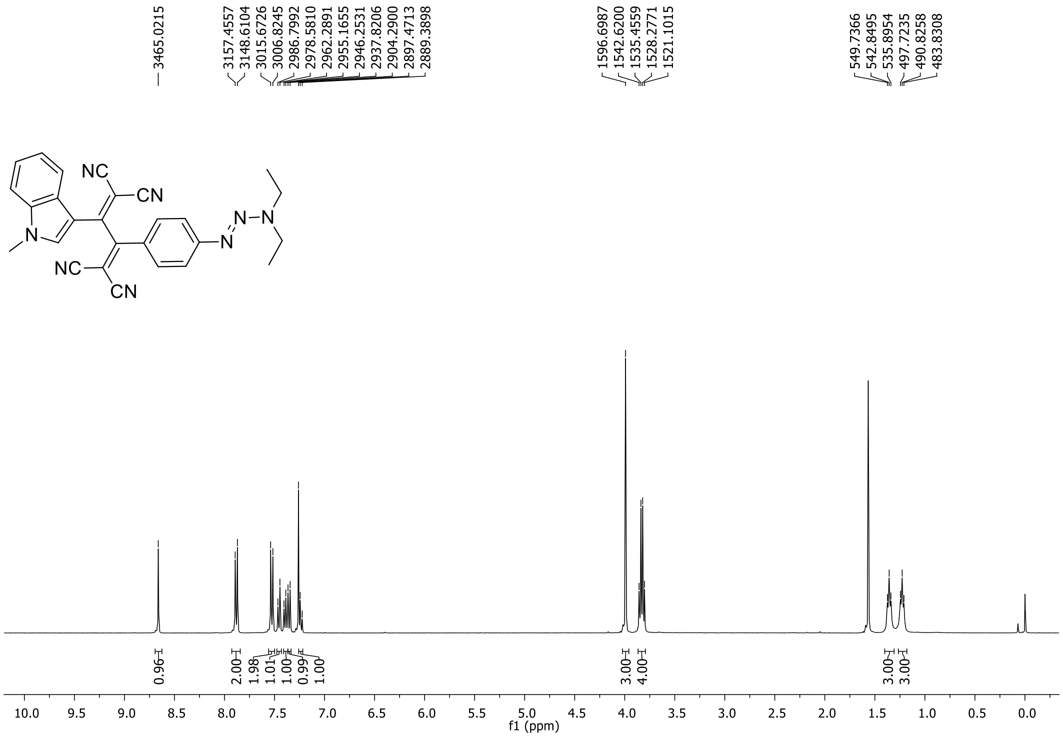

Figure S18. ^{1}H NMR spectrum of **5a** in $CDCl_3$ solution (400 MHz).

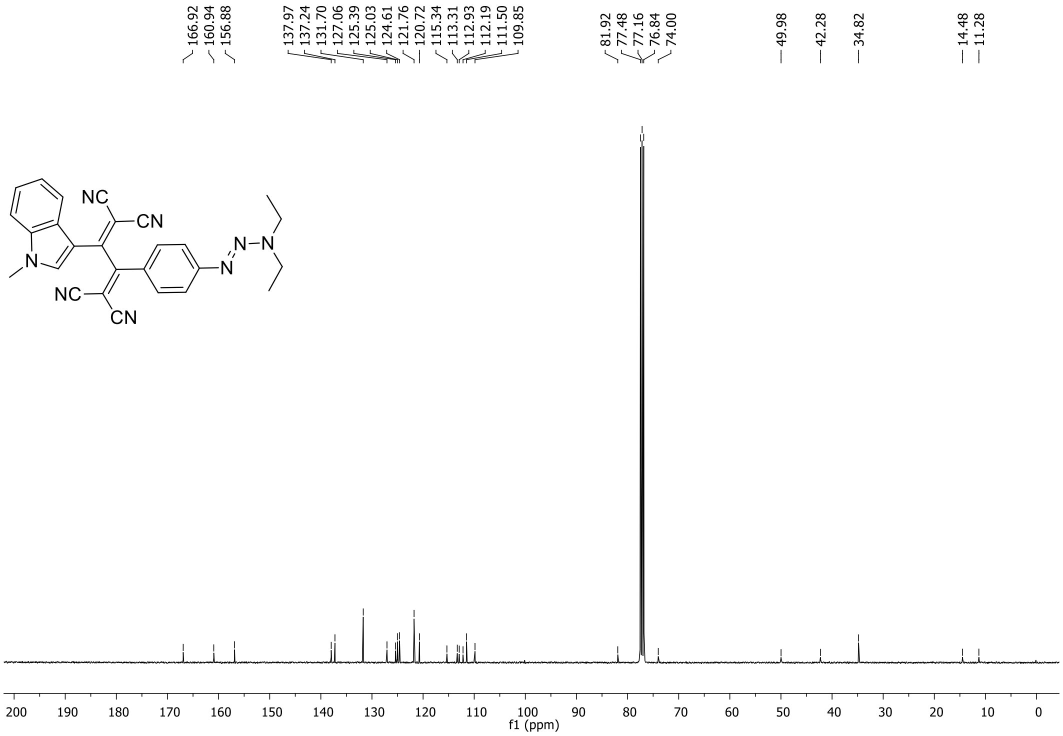

Figure S19. $^{13}C\{^{1}H\}$ NMR spectrum of **5a** in $CDCl_3$ solution (100 MHz).

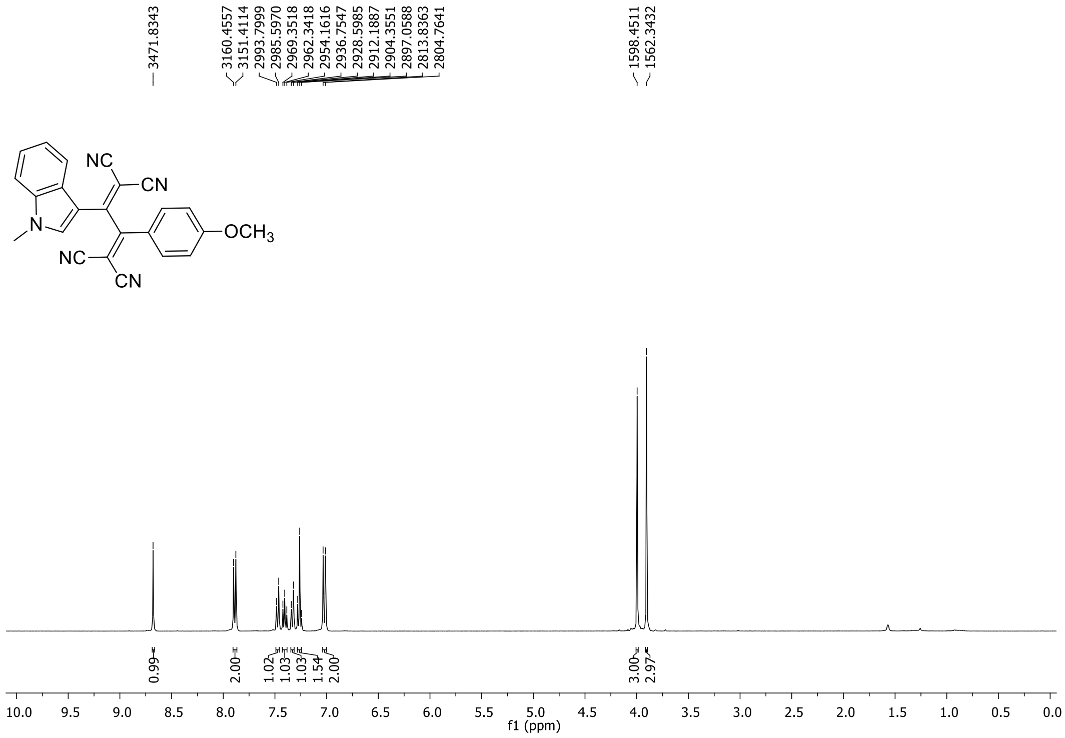

Figure S20. ^{1}H NMR spectrum of **5b** in $CDCl_3$ solution (400 MHz).

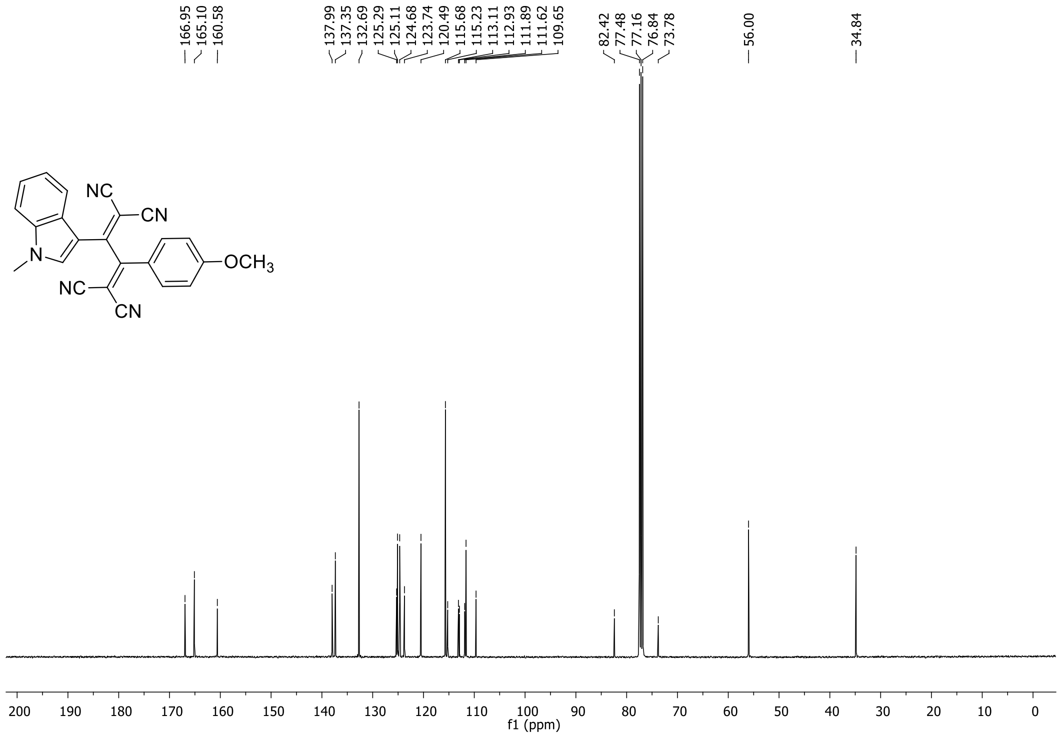

Figure S21. $^{13}C\{^{1}H\}$ NMR spectrum of **5b** in $CDCl_3$ solution (100 MHz).

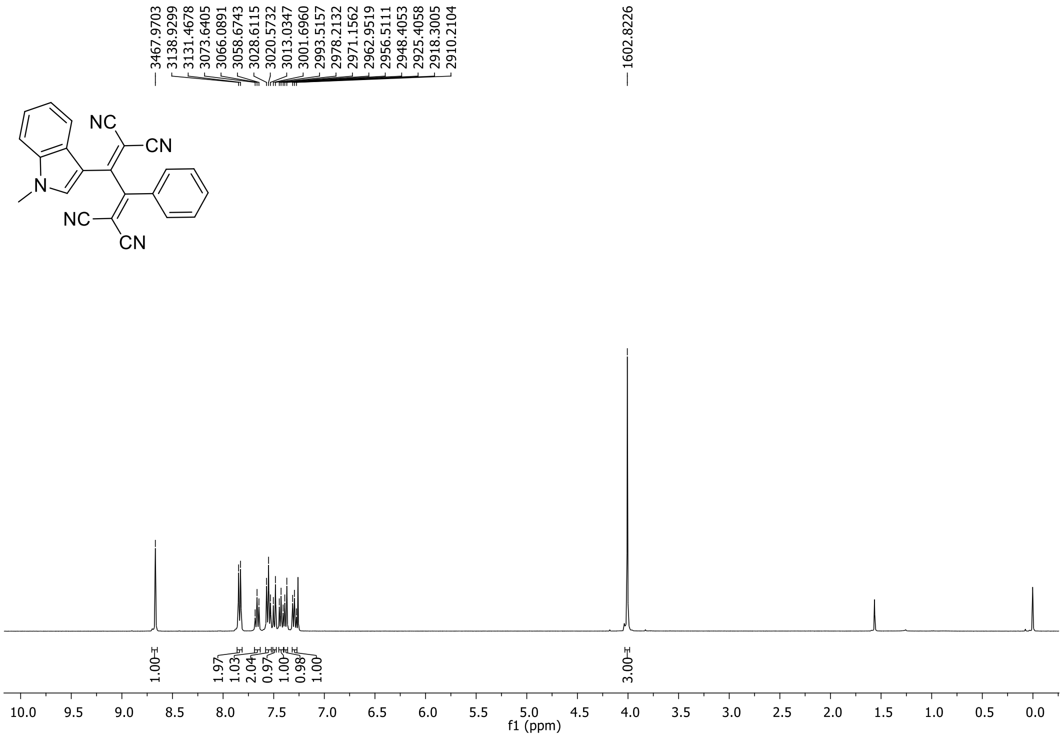

Figure S22. ^{1}H NMR spectrum of **5c** in $CDCl_3$ solution (400 MHz).

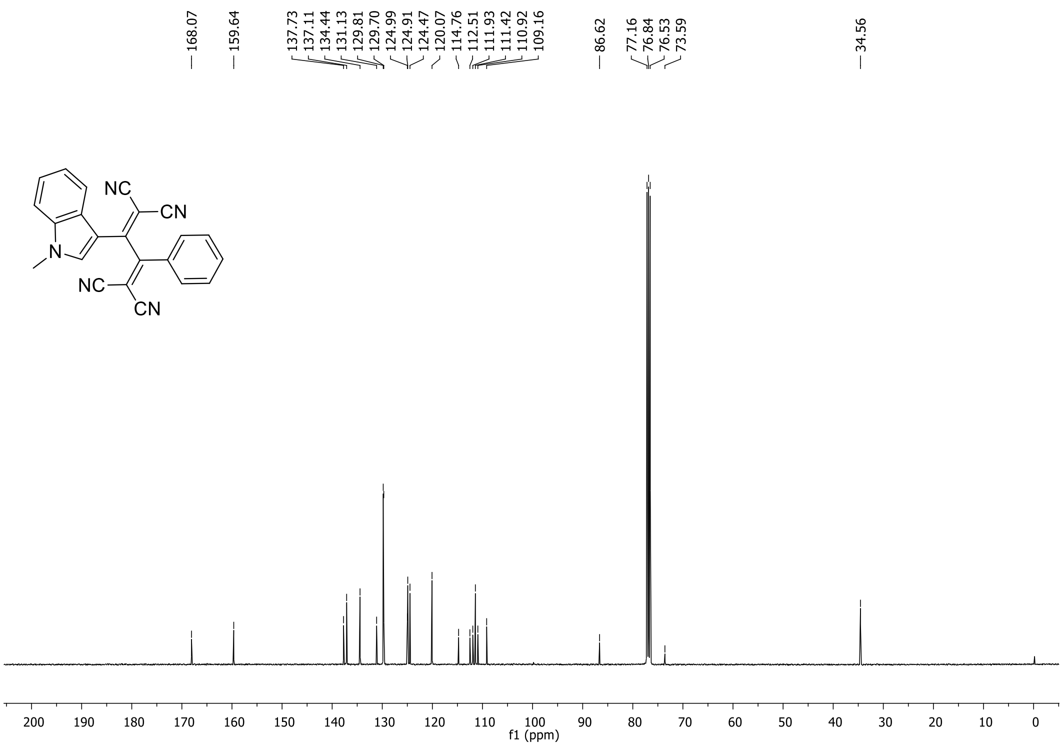

Figure S23. $^{13}C\{^{1}H\}$ NMR spectrum of **5c** in $CDCl_3$ solution (100 MHz).

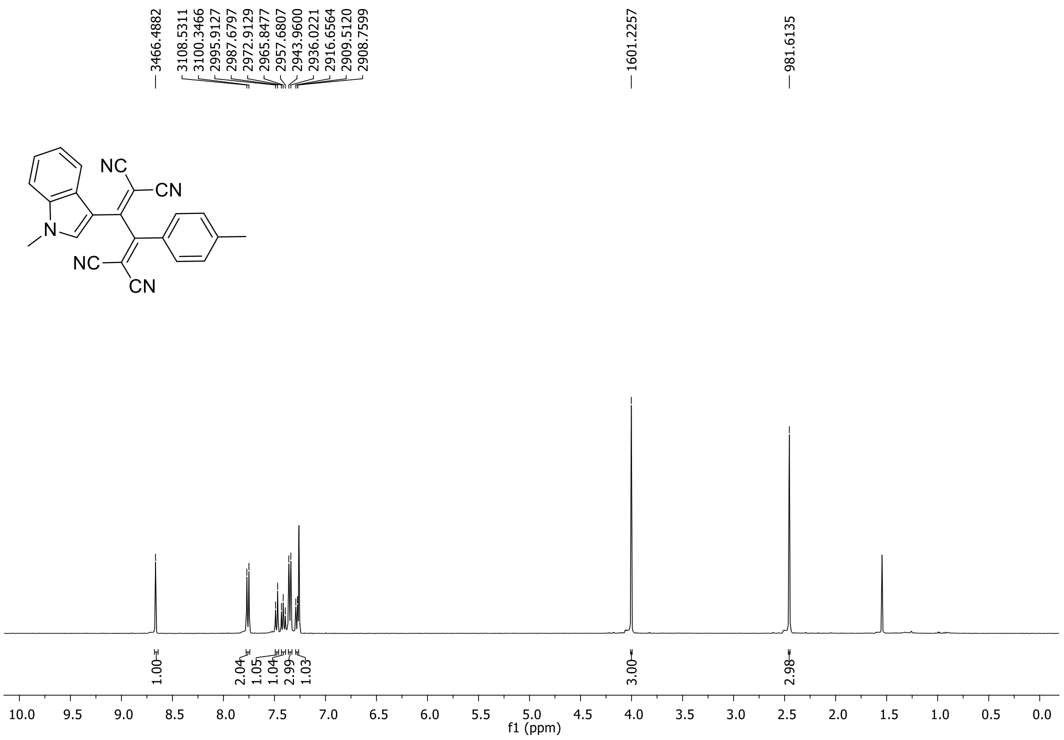

Figure S24. ^{1}H NMR spectrum of **5d** in $CDCl_3$ solution (400 MHz).

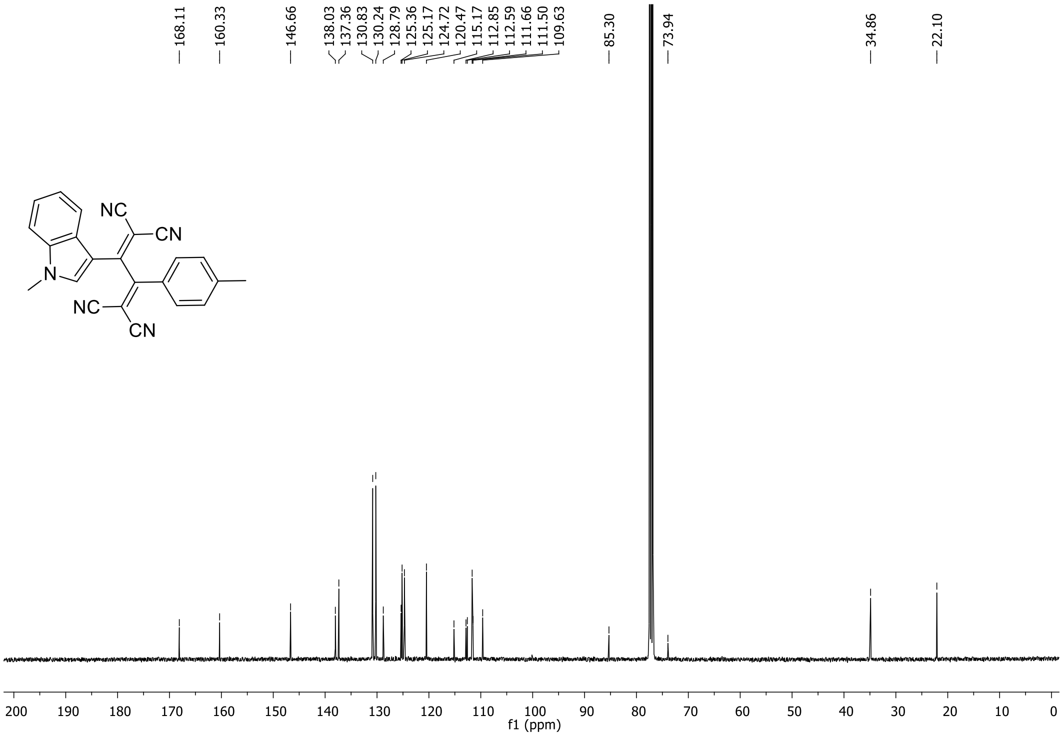

Figure S25. $^{13}C\{^1H\}$ NMR spectrum of **5d** in $CDCl_3$ solution (100 MHz).

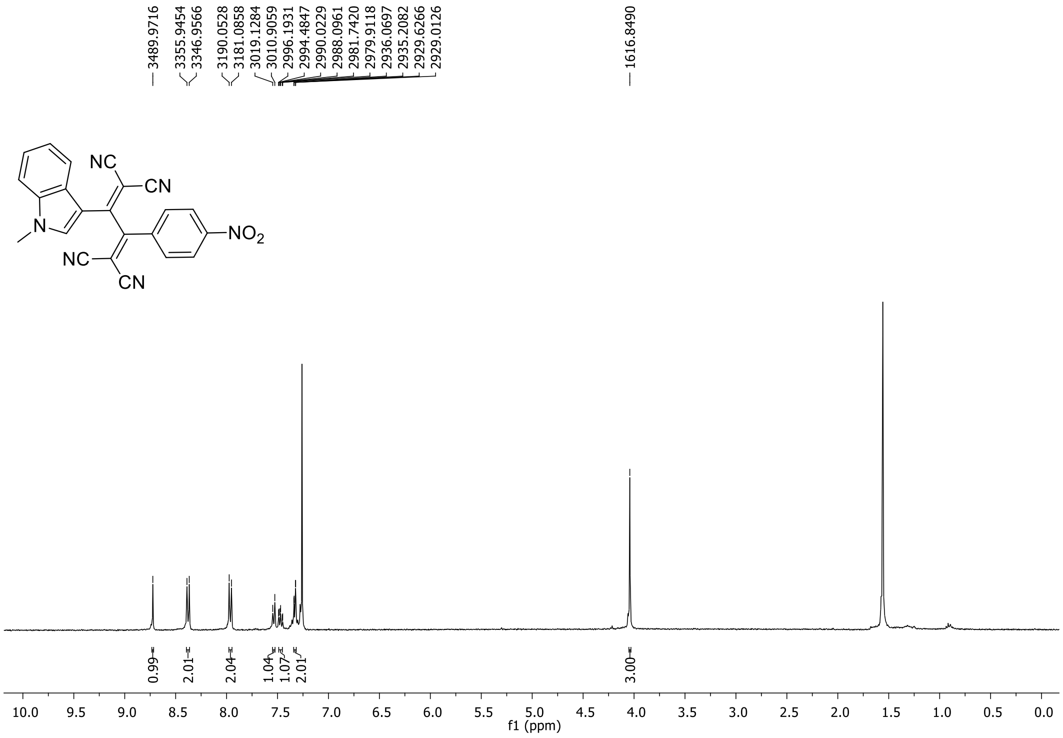

Figure S26. ^1H NMR spectrum of **5e** in $CDCl_3$ solution (400 MHz).

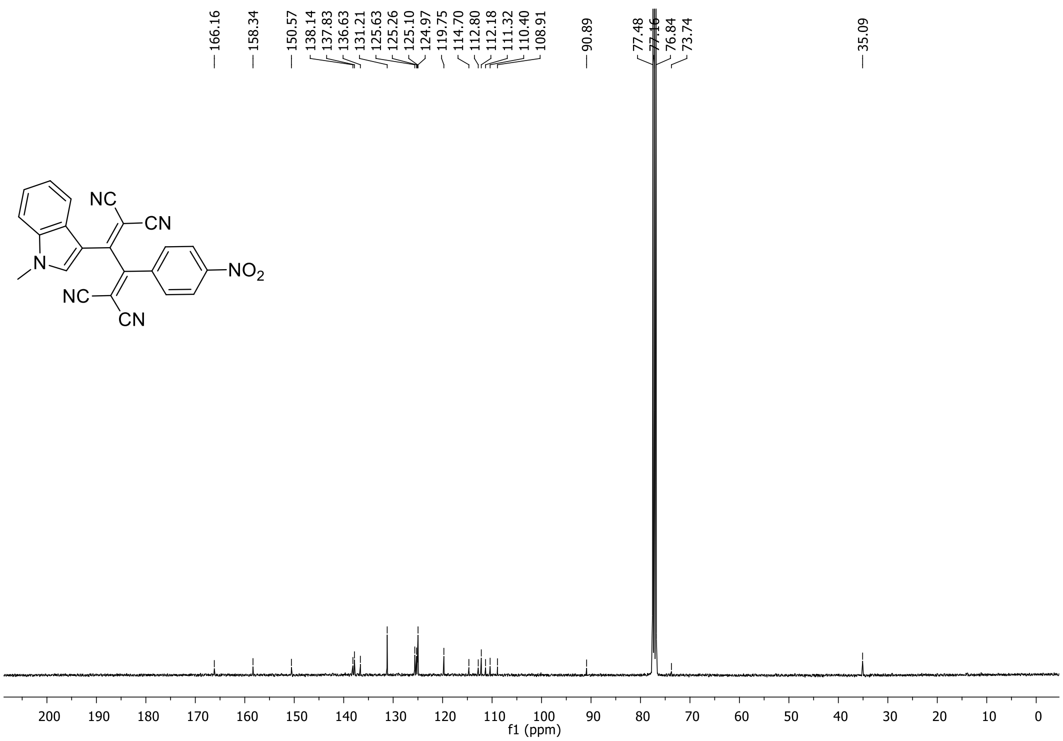

Figure S27. $^{13}C\{^1H\}$ NMR spectrum of **5e** in $CDCl_3$ solution (100 MHz).

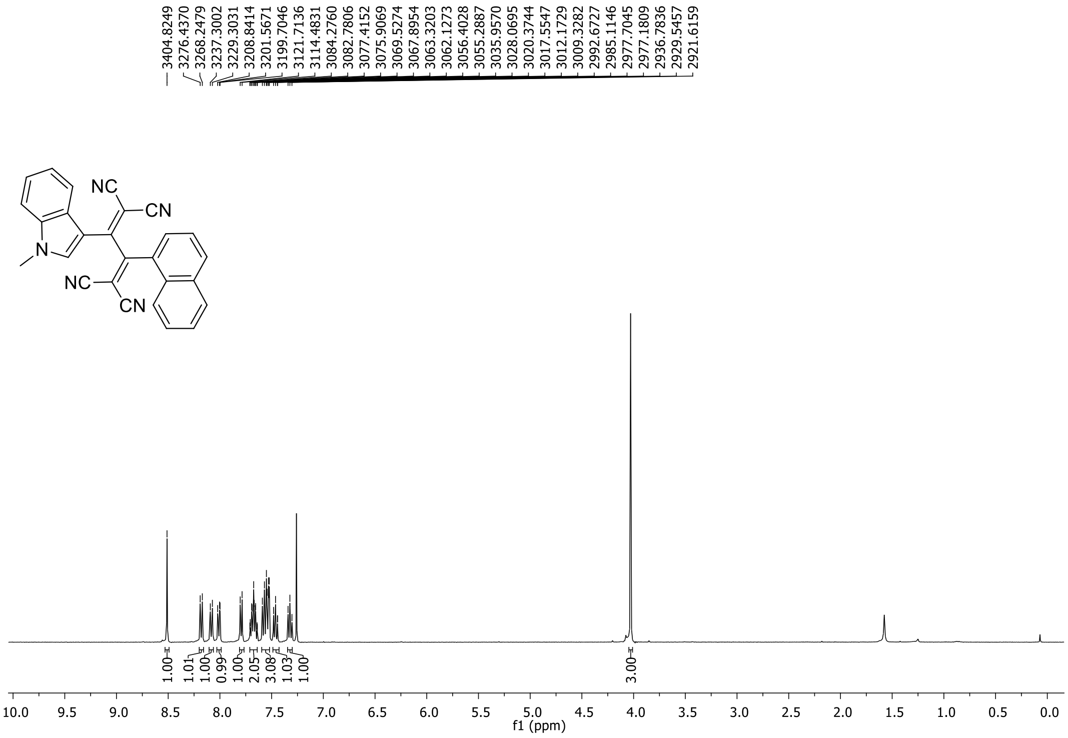

Figure S28. ^1H NMR spectrum of **5f** in $CDCl_3$ solution (400 MHz).

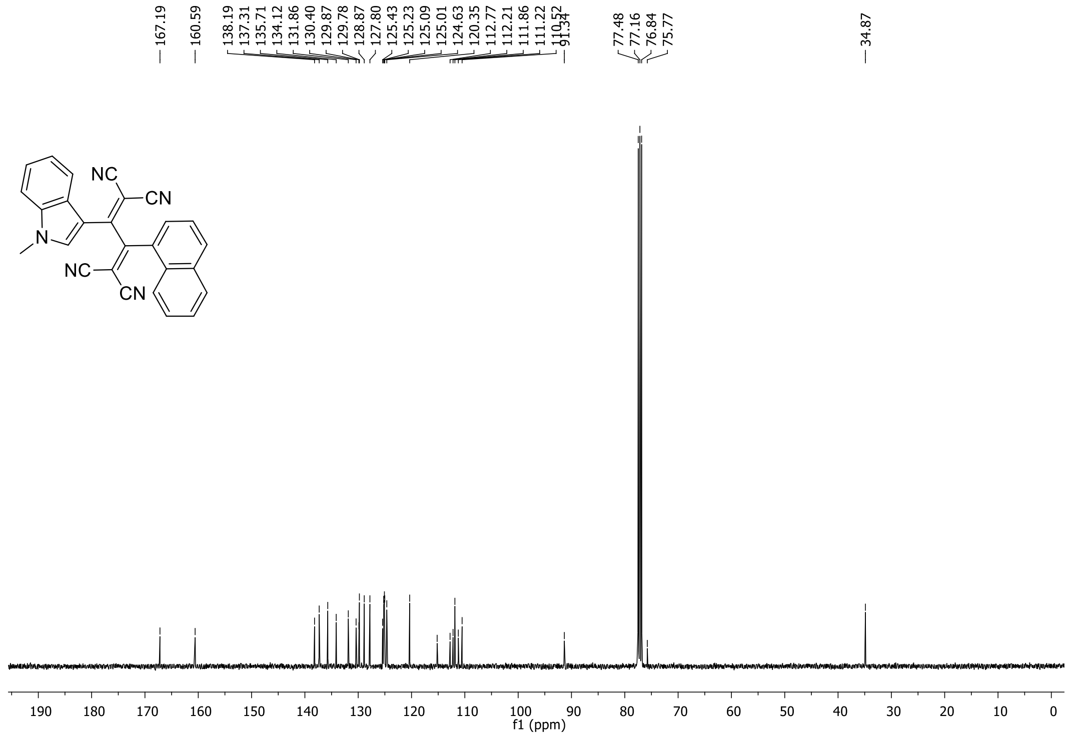

Figure S29. $^{13}C\{^{1}H\}$ NMR spectrum of **5f** in $CDCl_3$ solution (100 MHz).

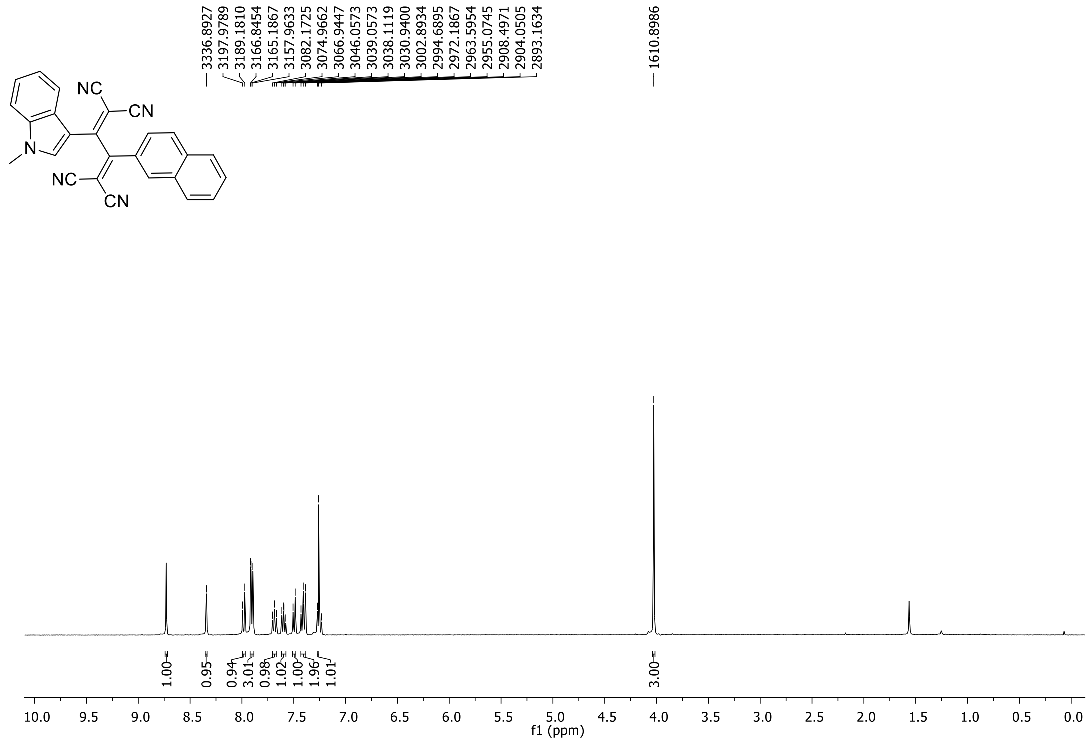

Figure S30. ^{1}H NMR spectrum of **5g** in $CDCl_3$ solution (400 MHz).

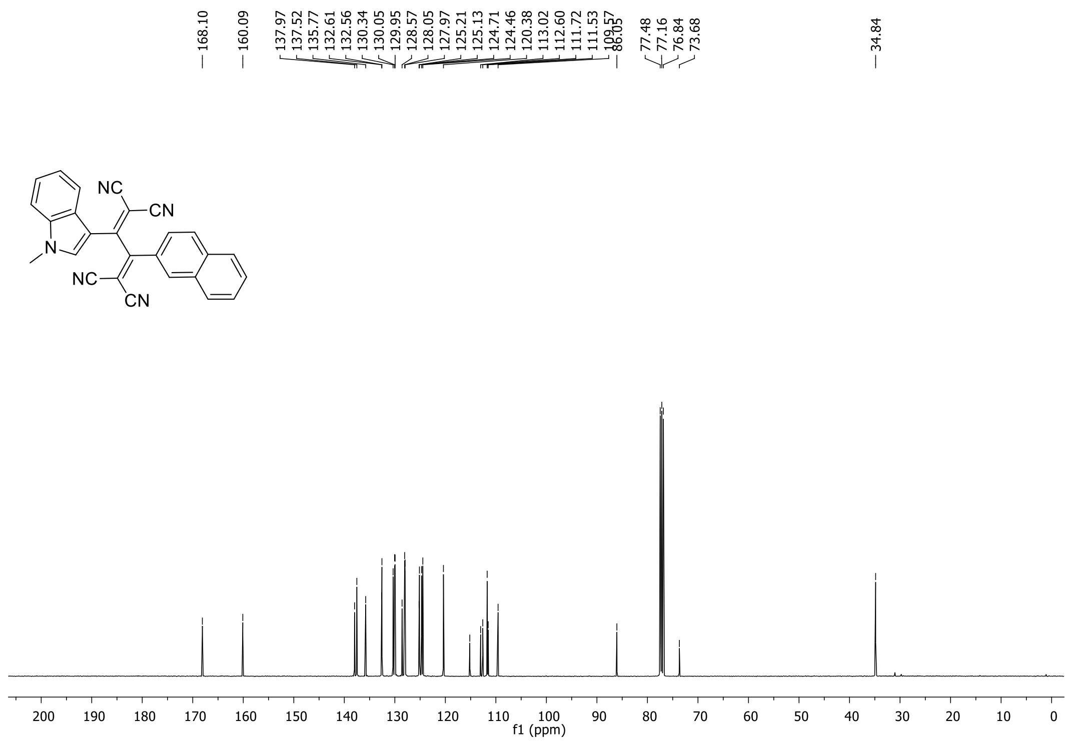

Figure S31. $^{13}C\{^{1}H\}$ NMR spectrum of **5g** in $CDCl_3$ solution (100 MHz).

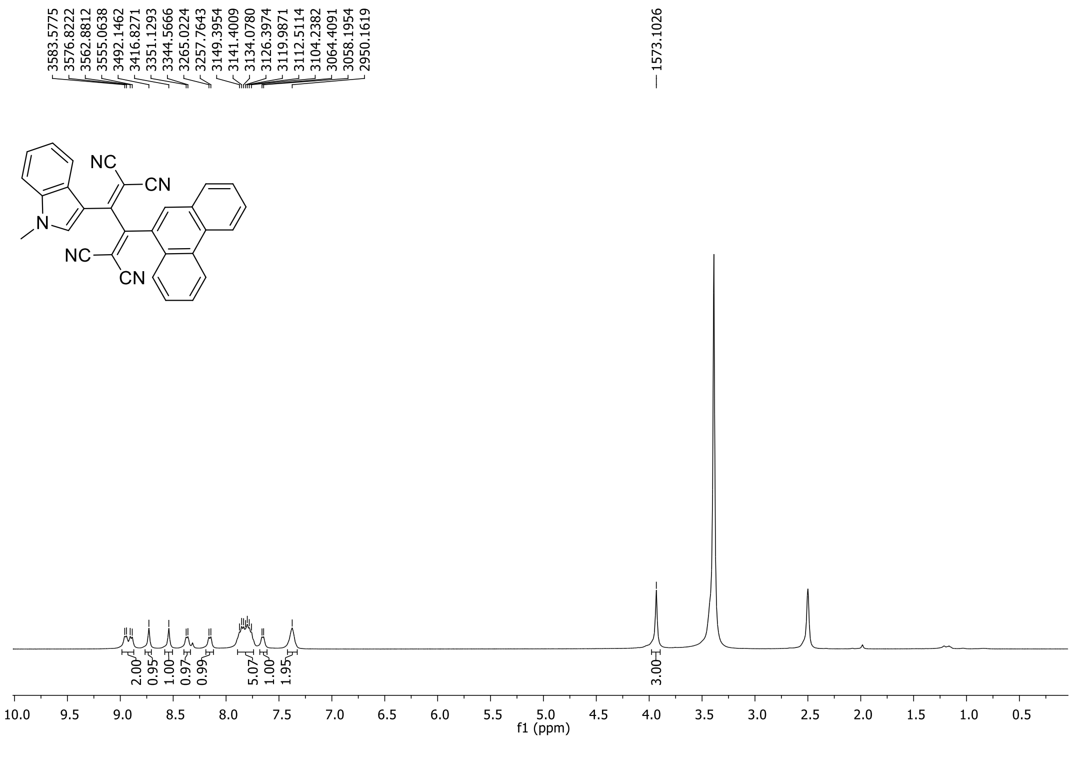

Figure S32. ^{1}H NMR spectrum of **5h** in DMSO-d6 solution (400 MHz).

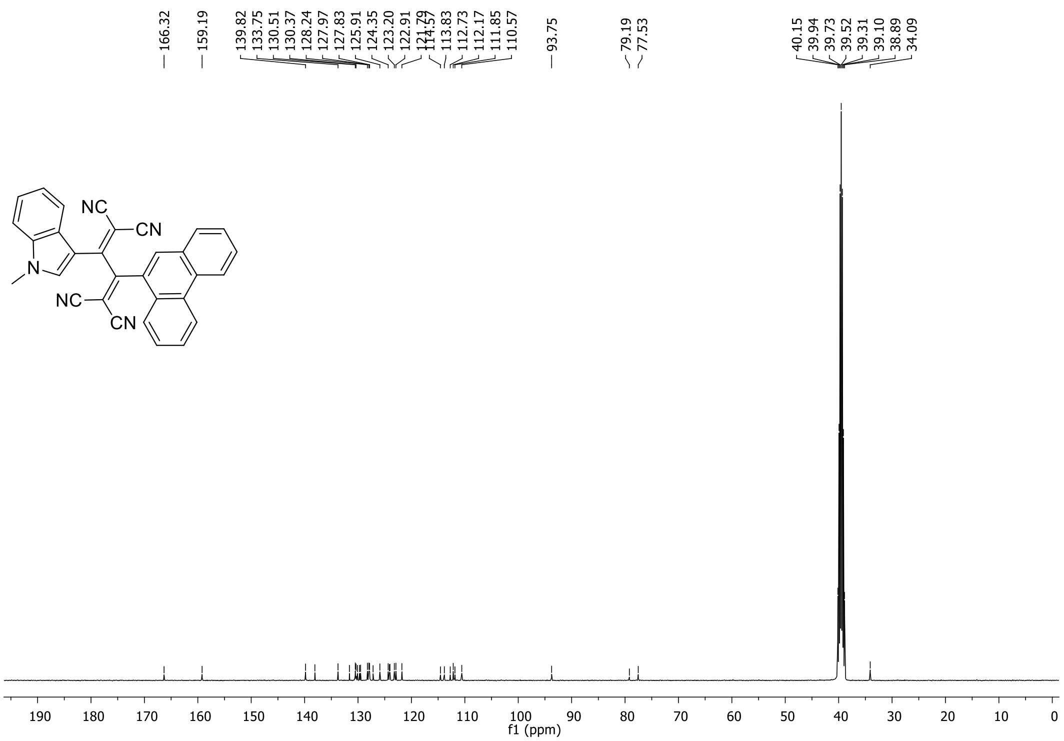

Figure S33. $^{13}C\{^{1}H\}$ NMR spectrum of **5h** in DMSO-d6 solution (100 MHz).

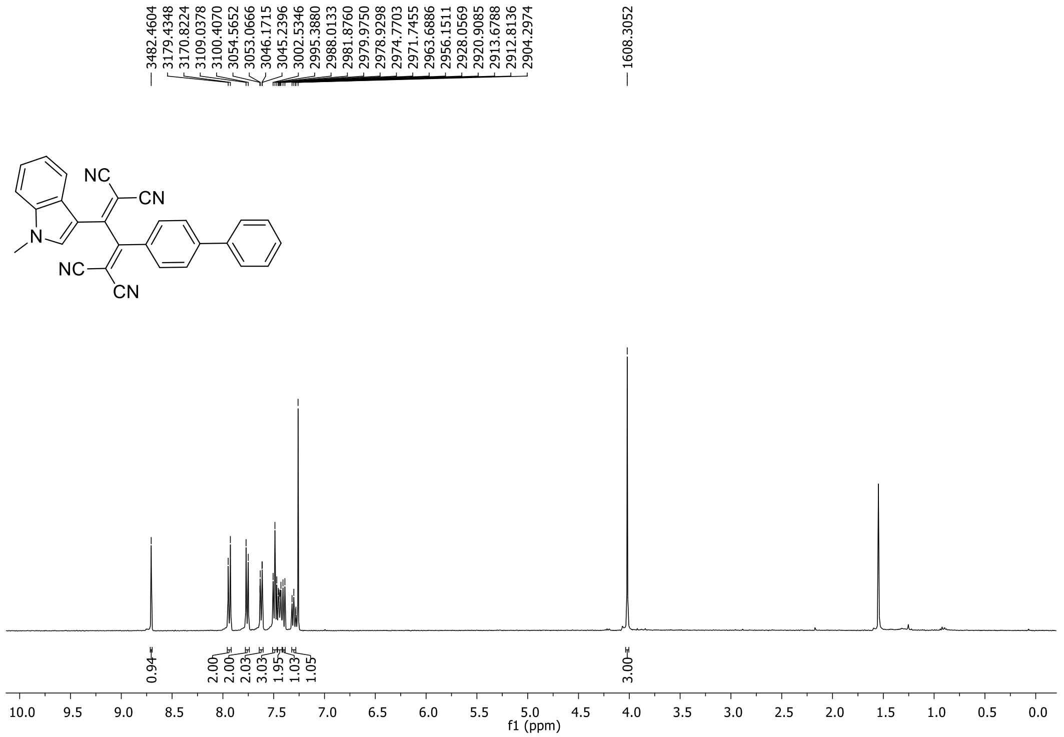

Figure S34. ^{1}H NMR spectrum of **5i** in $CDCl_3$ solution (400 MHz).

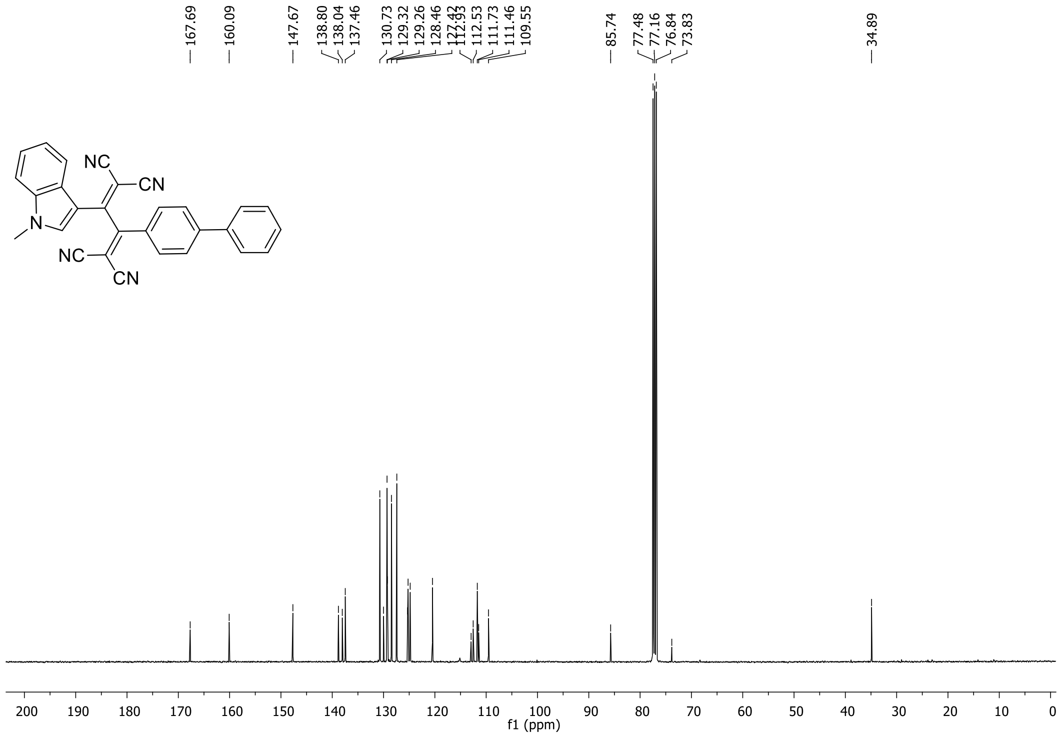

Figure S35. $^{13}C\{^{1}H\}$ NMR spectrum of **5i** in $CDCl_3$ solution (100 MHz).

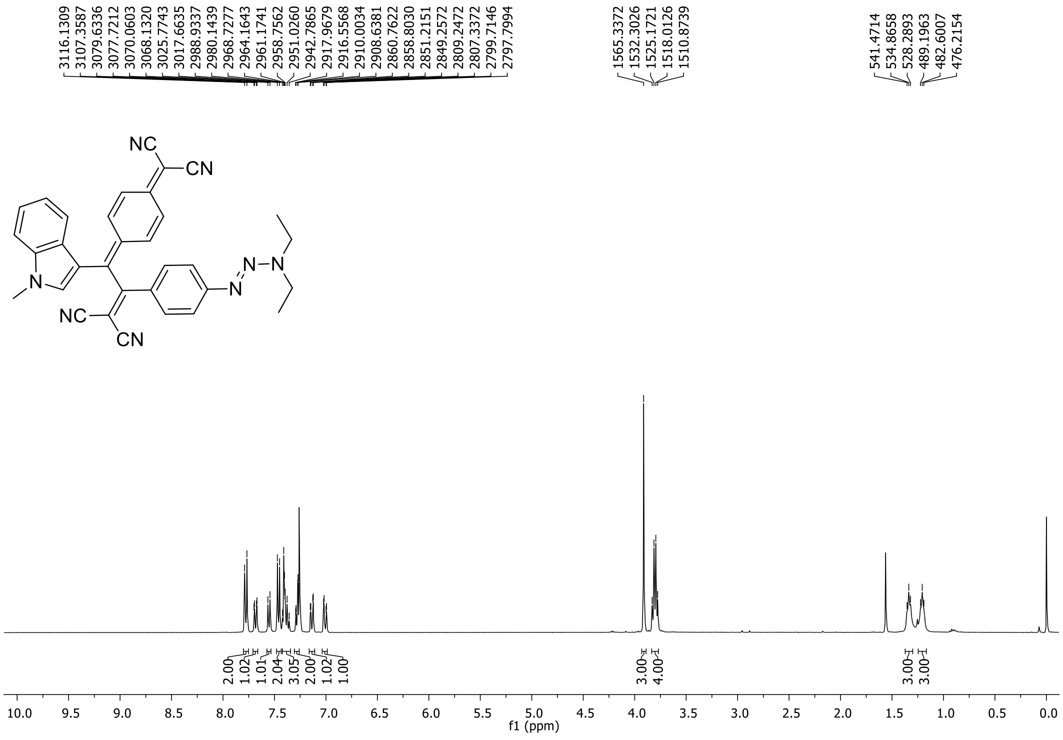

Figure S36. ^{1}H NMR spectrum of **7a** in $CDCl_3$ solution (400 MHz).

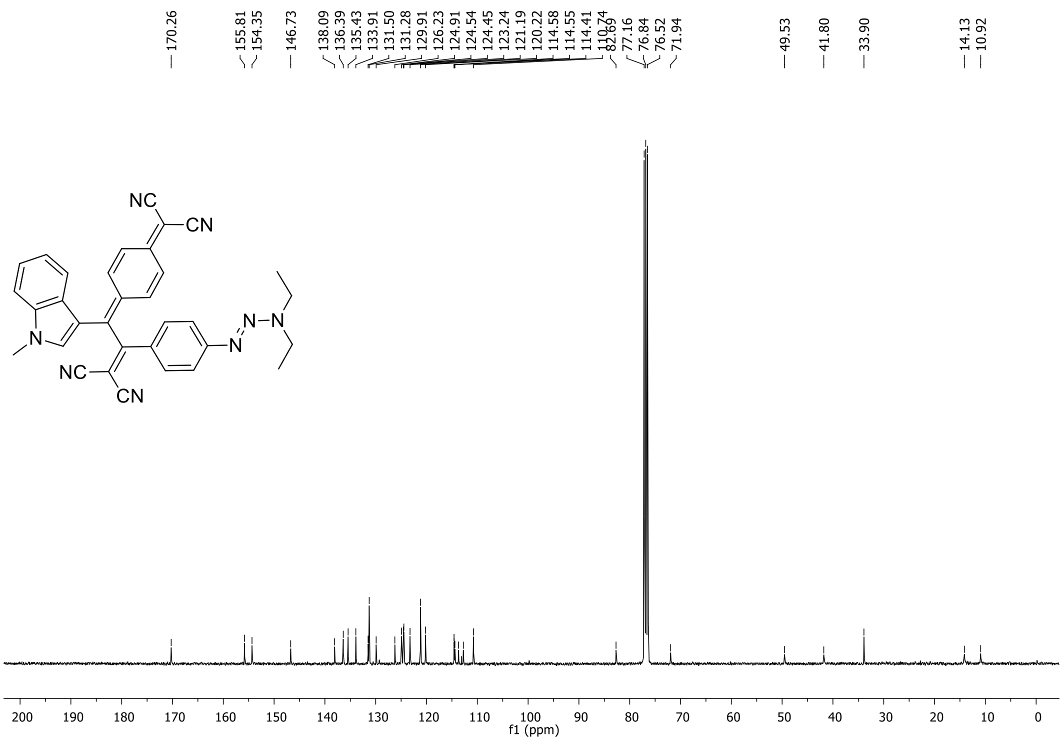

Figure S37. $^{13}C\{^1H\}$ NMR spectrum of **7a** in $CDCl_3$ solution (100 MHz).

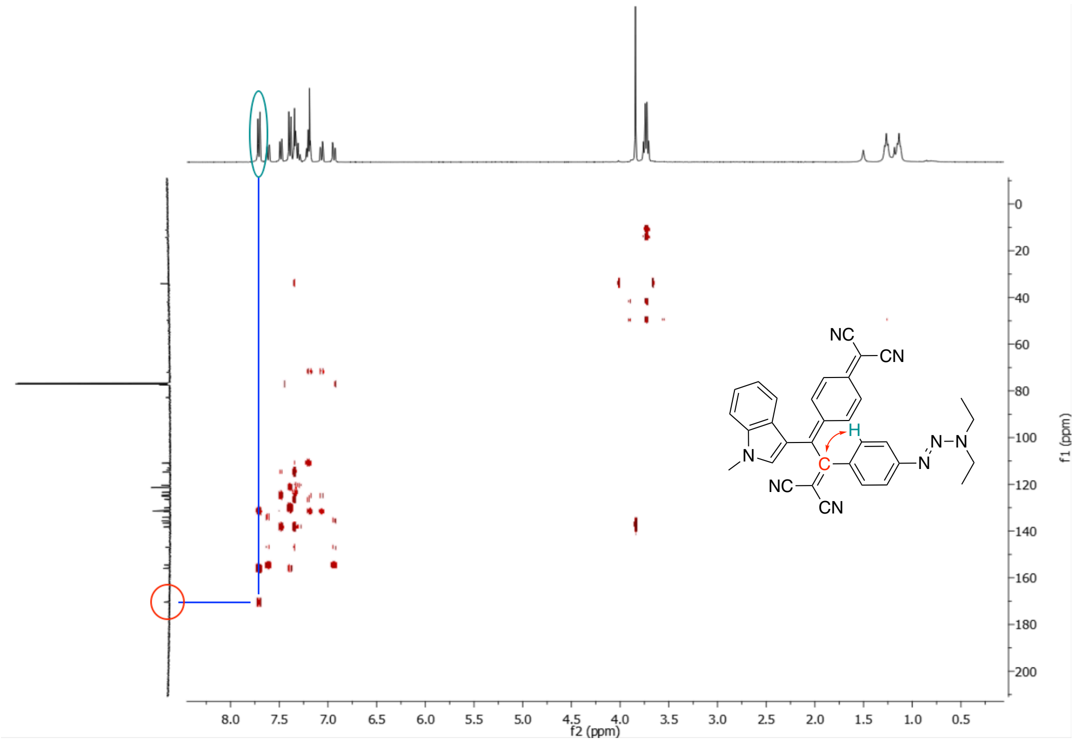

Figure S38. HMBC spectrum of **7a** in $CDCl_3$ solution.

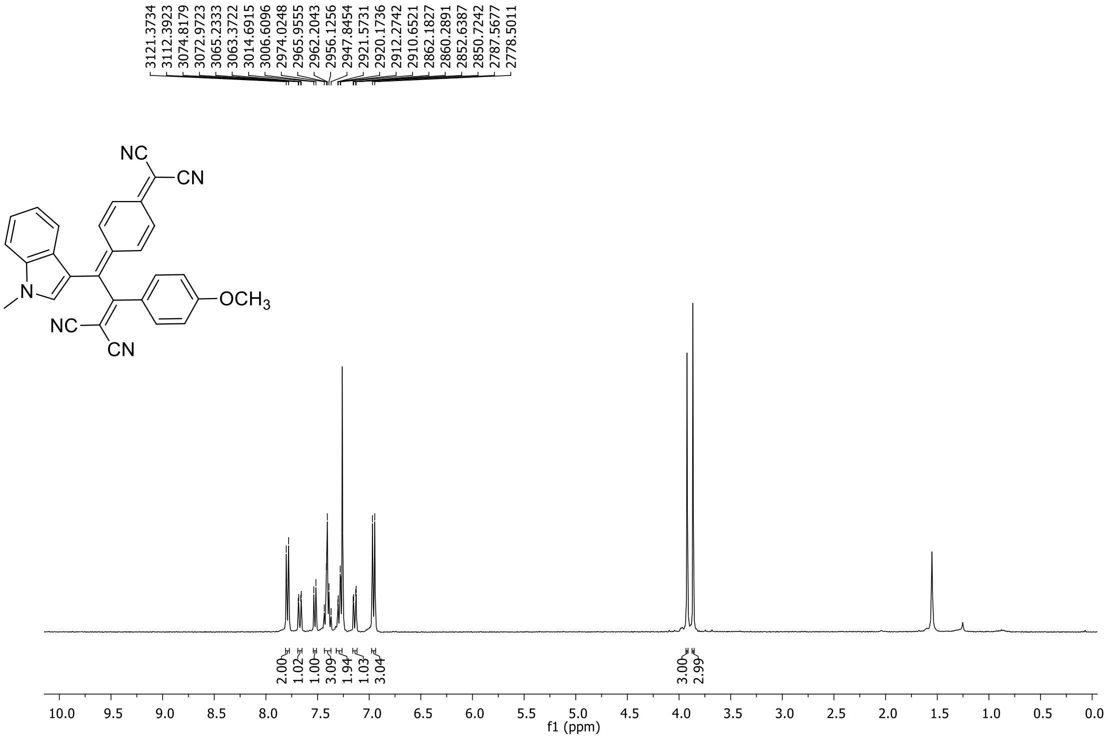

Figure S39. ^{1}H NMR spectrum of **7b** in $CDCl_3$ solution (400 MHz).

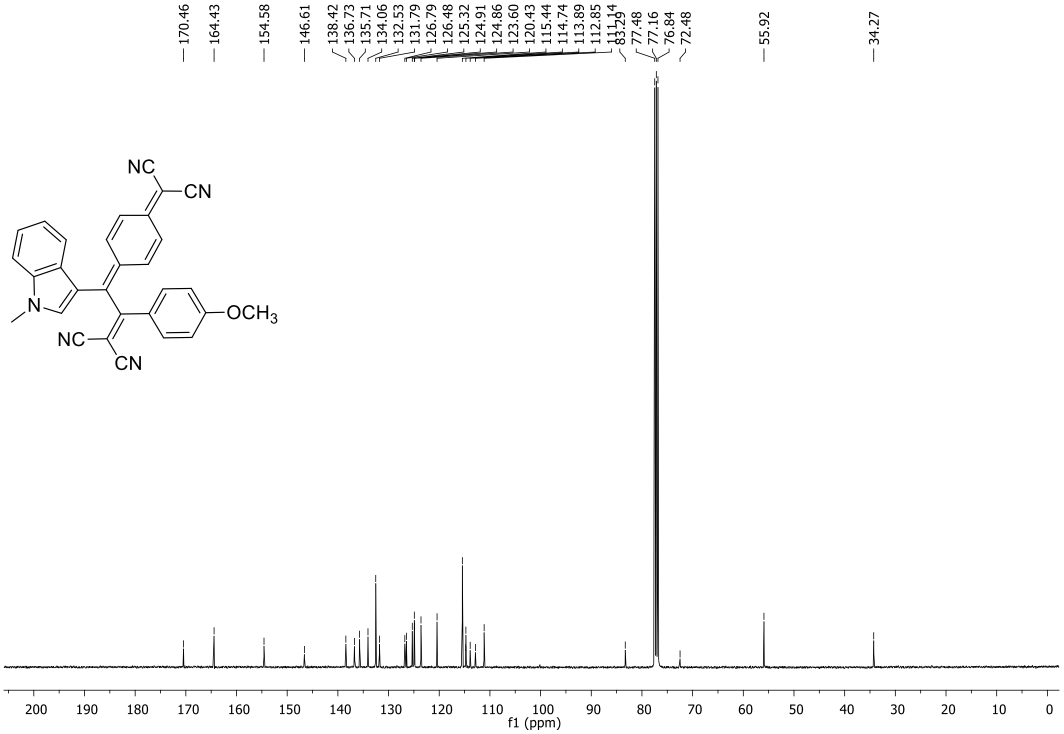

Figure S40. $^{13}C\{^{1}H\}$ NMR spectrum of **7b** in $CDCl_3$ solution (100 MHz).

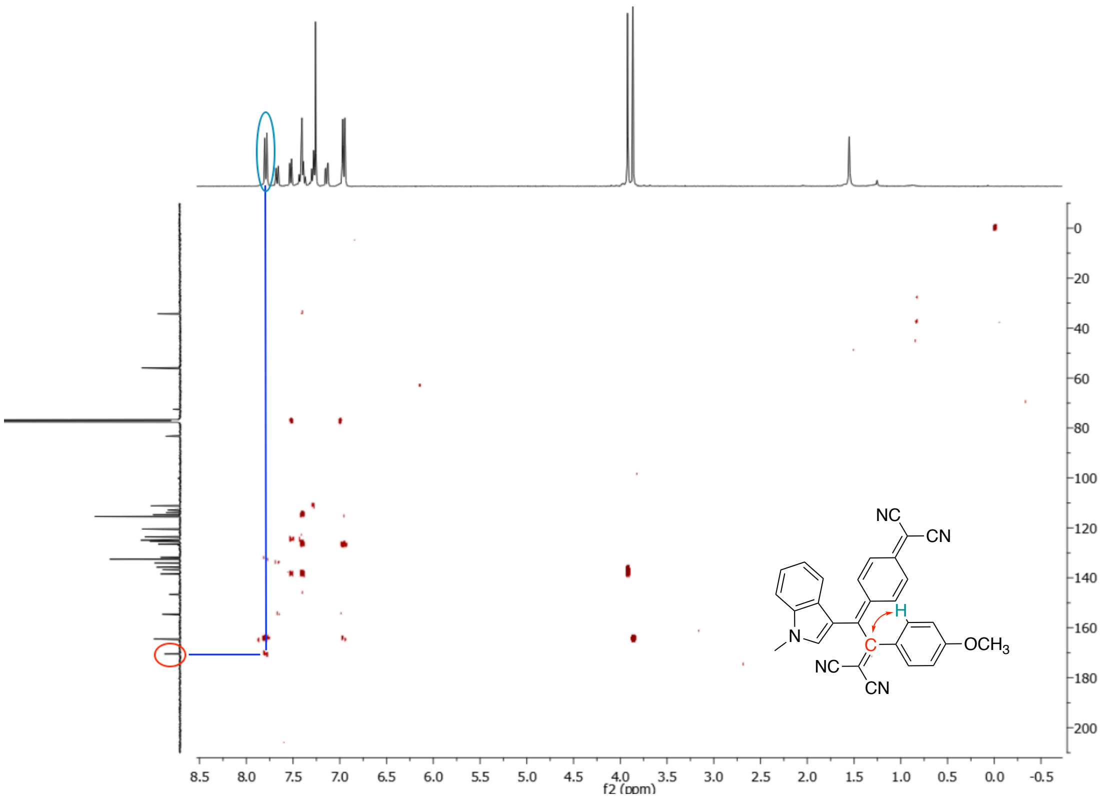

Figure S41. HMBC spectrum of **7b** in $CDCl_3$ solution.

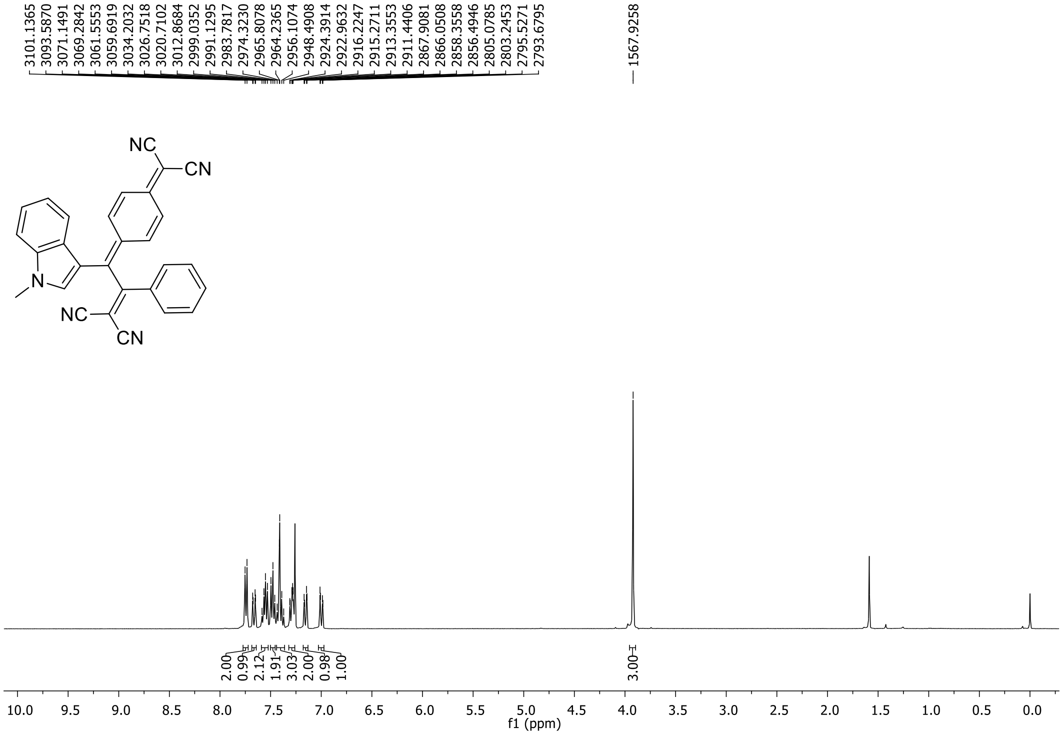

Figure S42. ^{1}H NMR spectrum of **7c** in $CDCl_3$ solution (400 MHz).

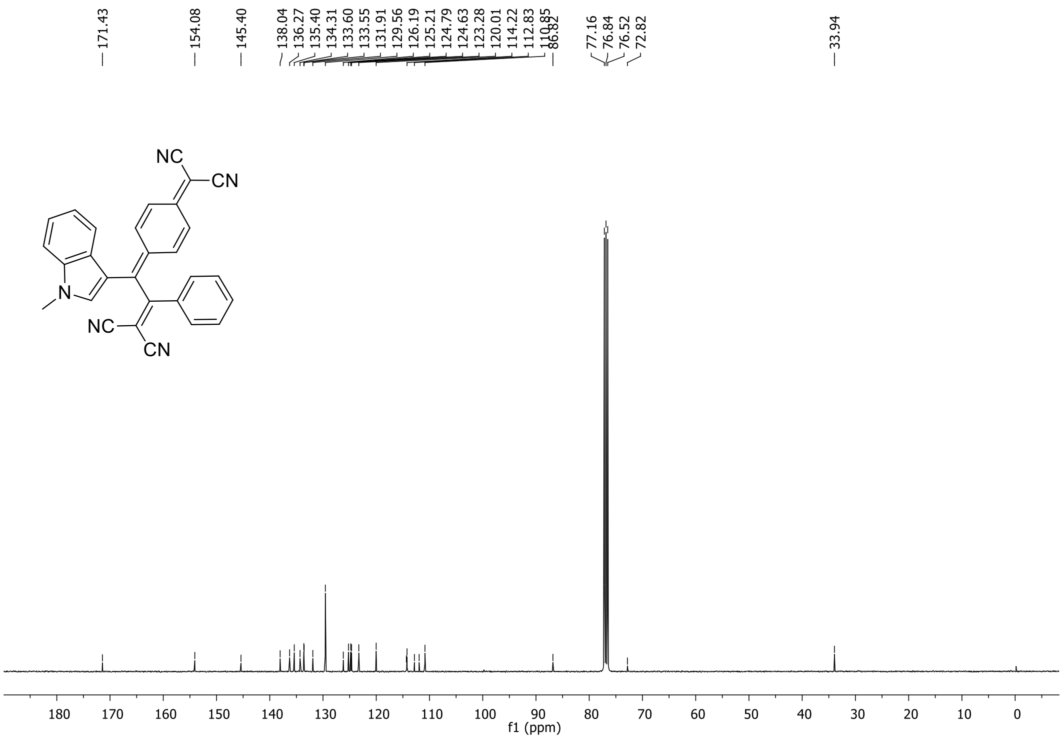

Figure43. $^{13}C\{^1H\}$ NMR spectrum of **7c** in $CDCl_3$ solution (100 MHz).

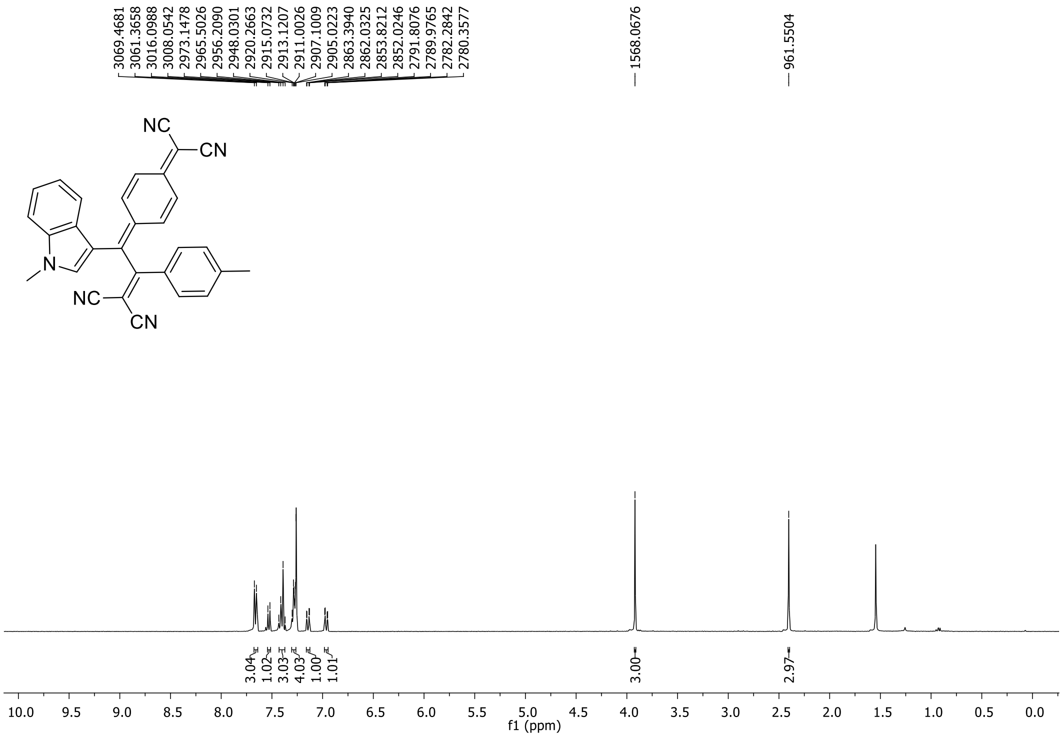

Figure S44. ^1H NMR spectrum of **7d** in $CDCl_3$ solution (400 MHz).

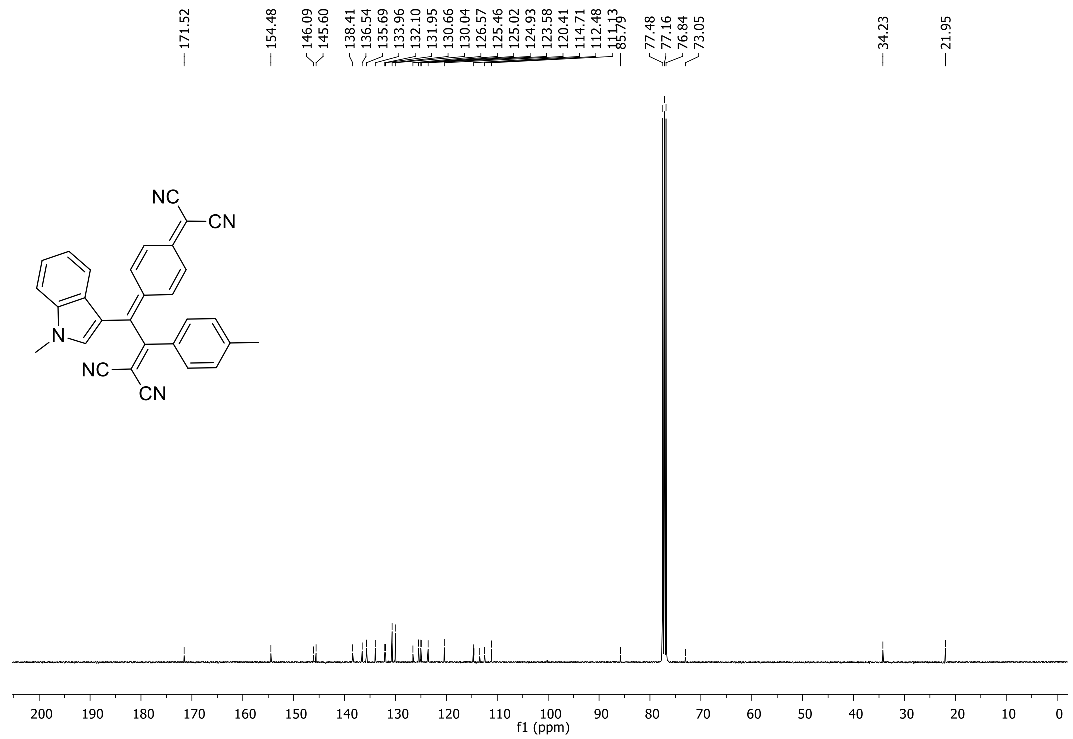

Figure S45. $^{13}C\{^1H\}$ NMR spectrum of **7d** in $CDCl_3$ solution (100 MHz).

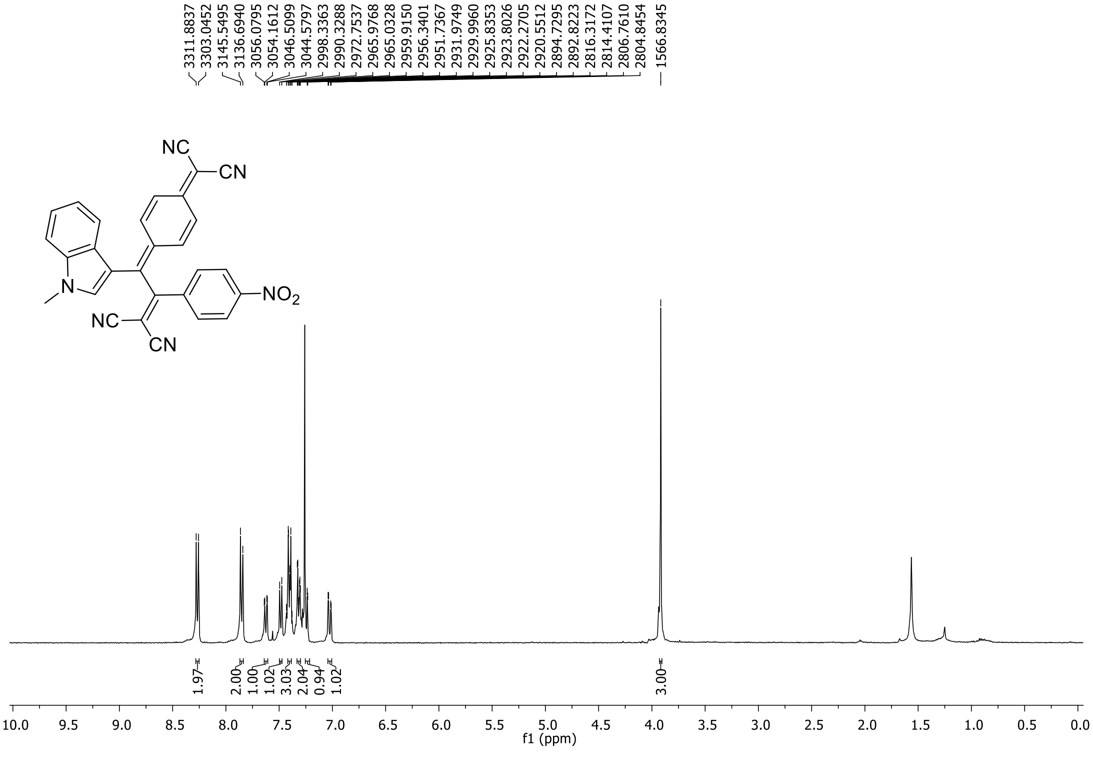

Figure S46. ^1H NMR spectrum of **7e** in $CDCl_3$ solution (400 MHz).

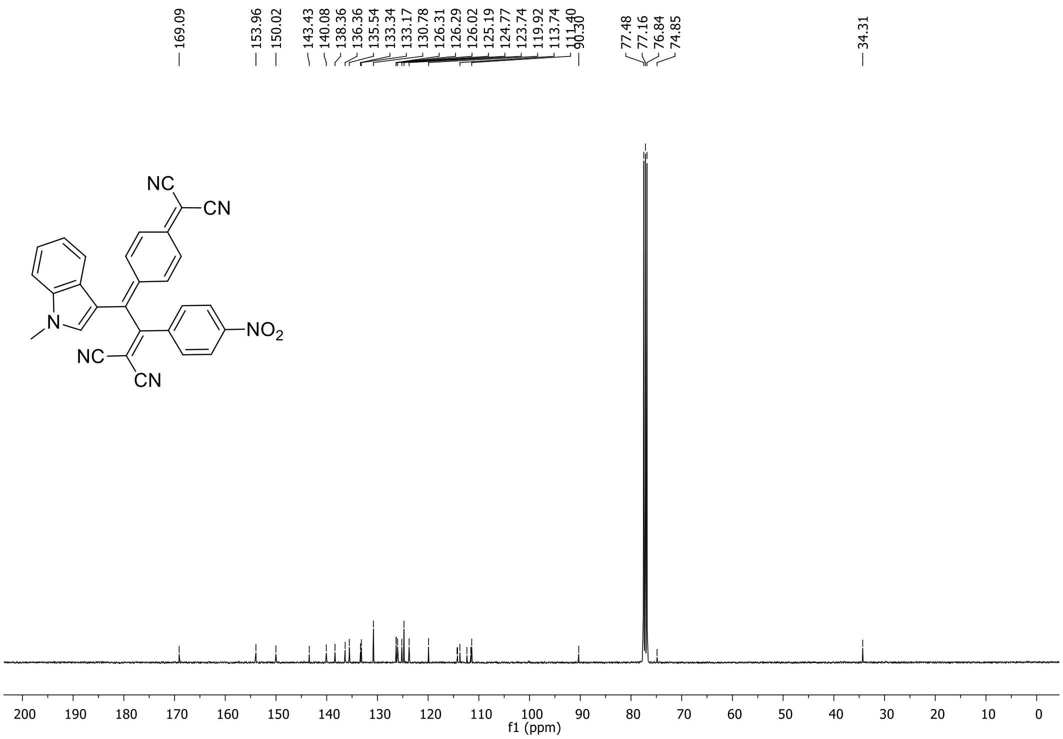

Figure S47. $^{13}C\{^{1}H\}$ NMR spectrum of **7e** in $CDCl_3$ solution (100 MHz).

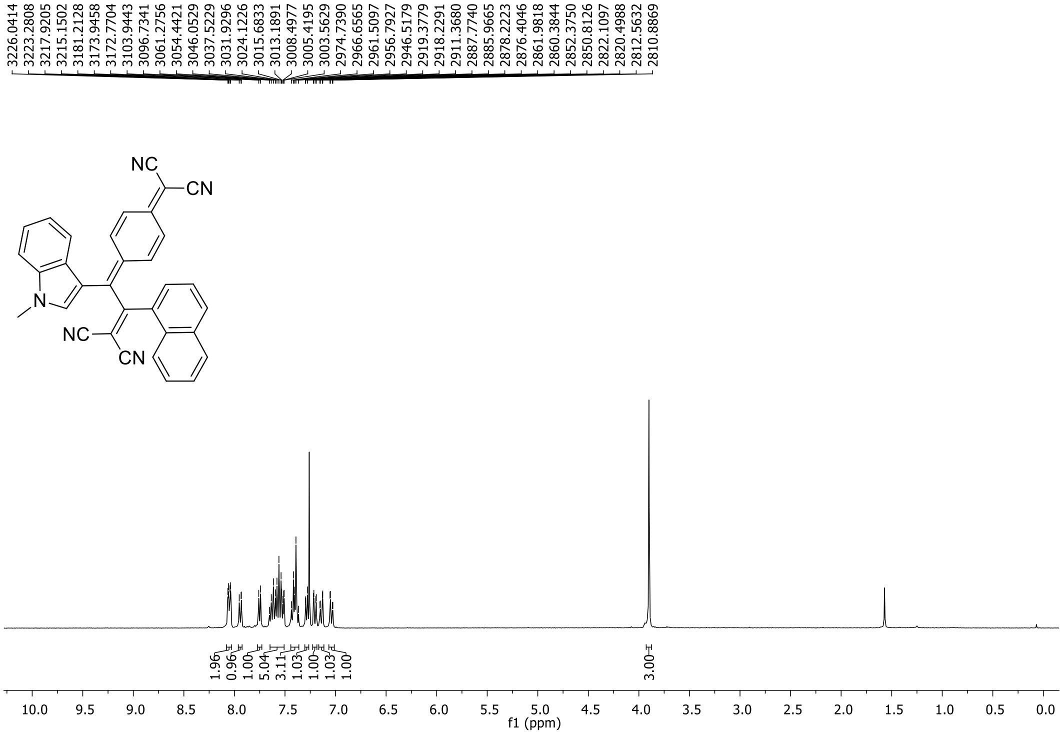

Figure S48. ^{1}H NMR spectrum of **7f** in $CDCl_3$ solution (400 MHz).

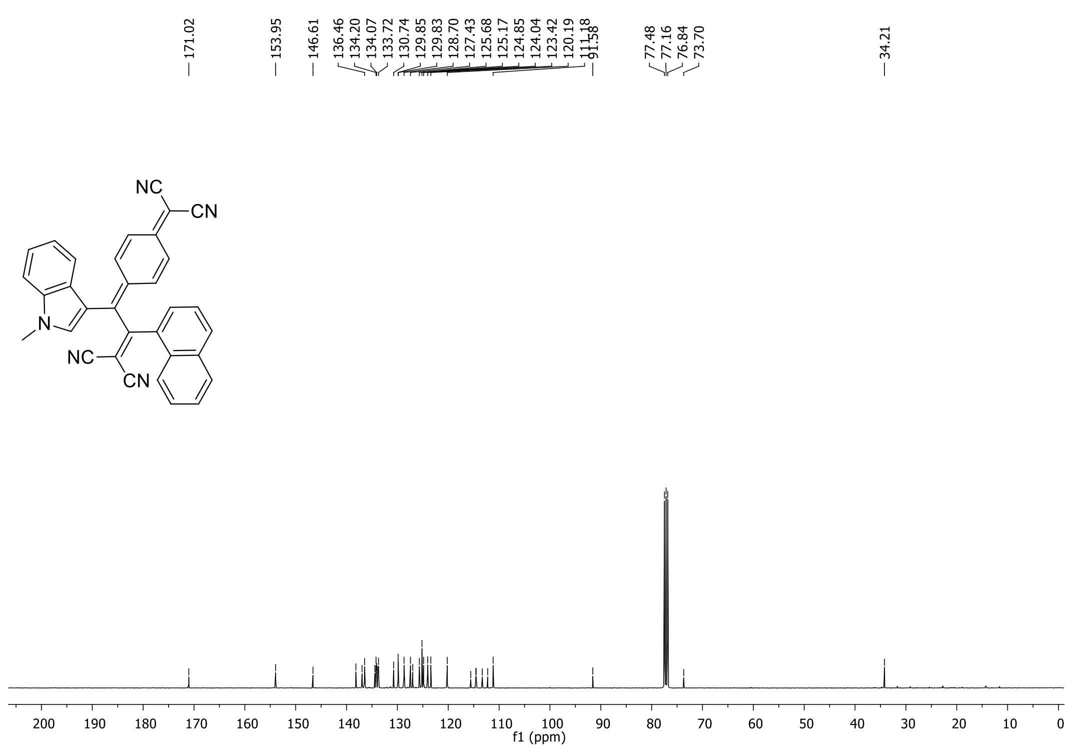

Figure S49. $^{13}C\{^{1}H\}$ NMR spectrum of **7f** in $CDCl_3$ solution (100 MHz).

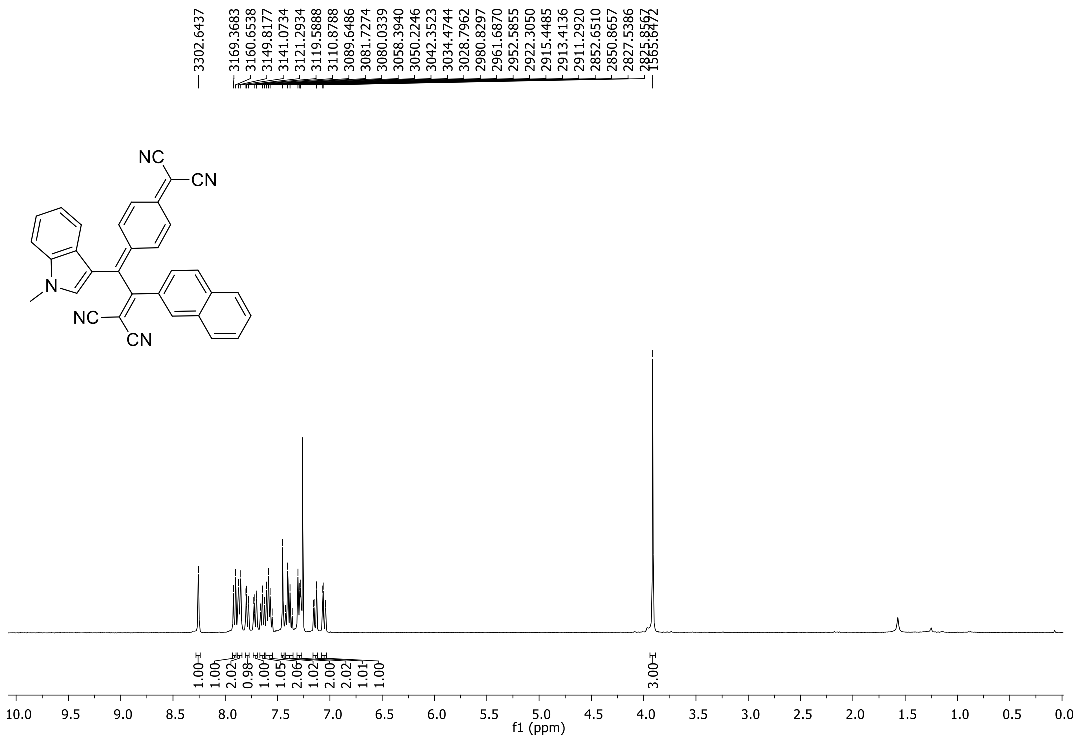

Figure S50. ^{1}H NMR spectrum of **7g** in $CDCl_3$ solution (400 MHz).

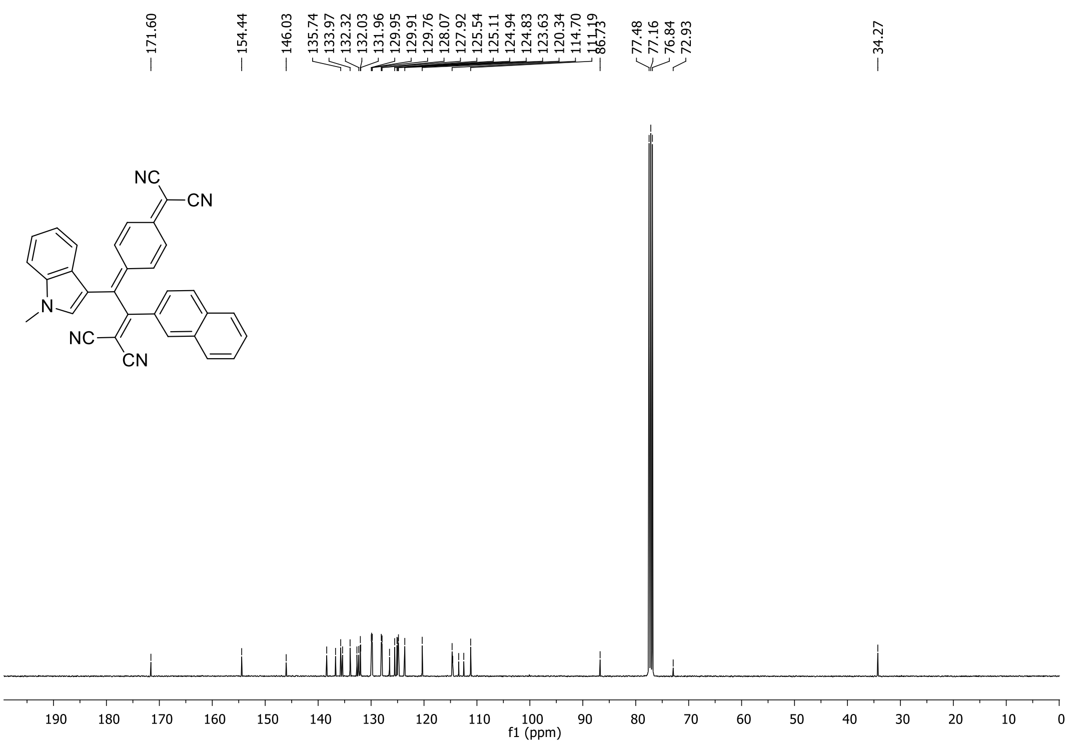

Figure S51. $^{13}C\{^{1}H\}$ NMR spectrum of **7g** in $CDCl_3$ solution (100 MHz).

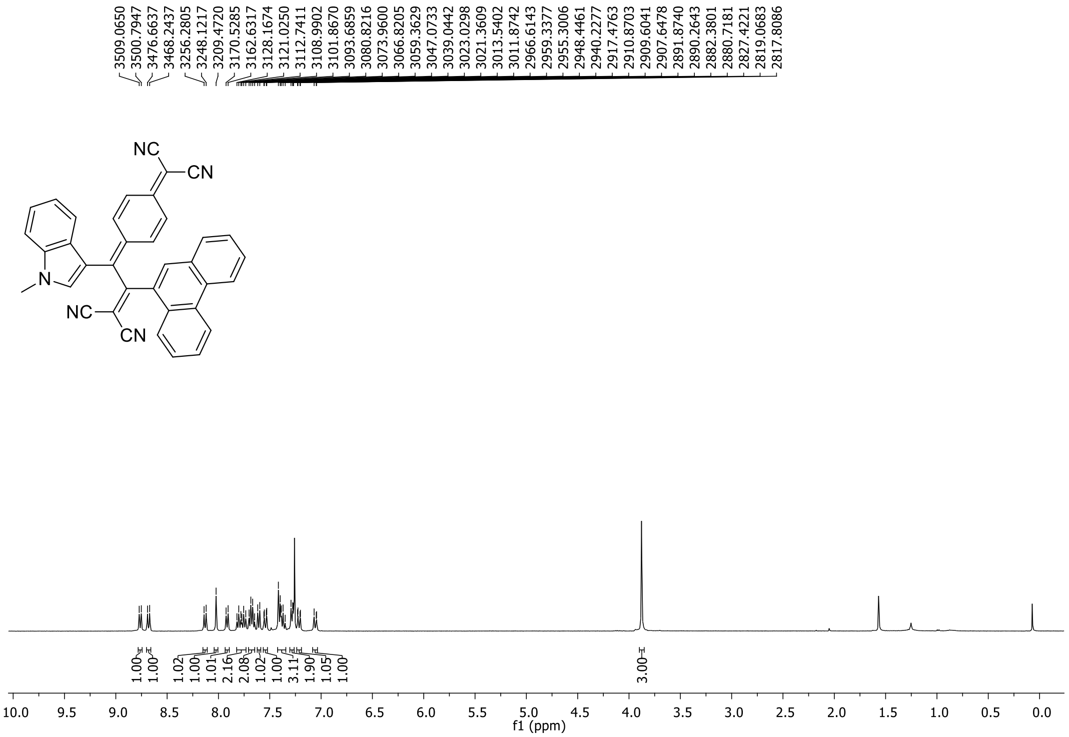

Figure S52. ^{1}H NMR spectrum of **7h** in $CDCl_3$ solution (400 MHz).

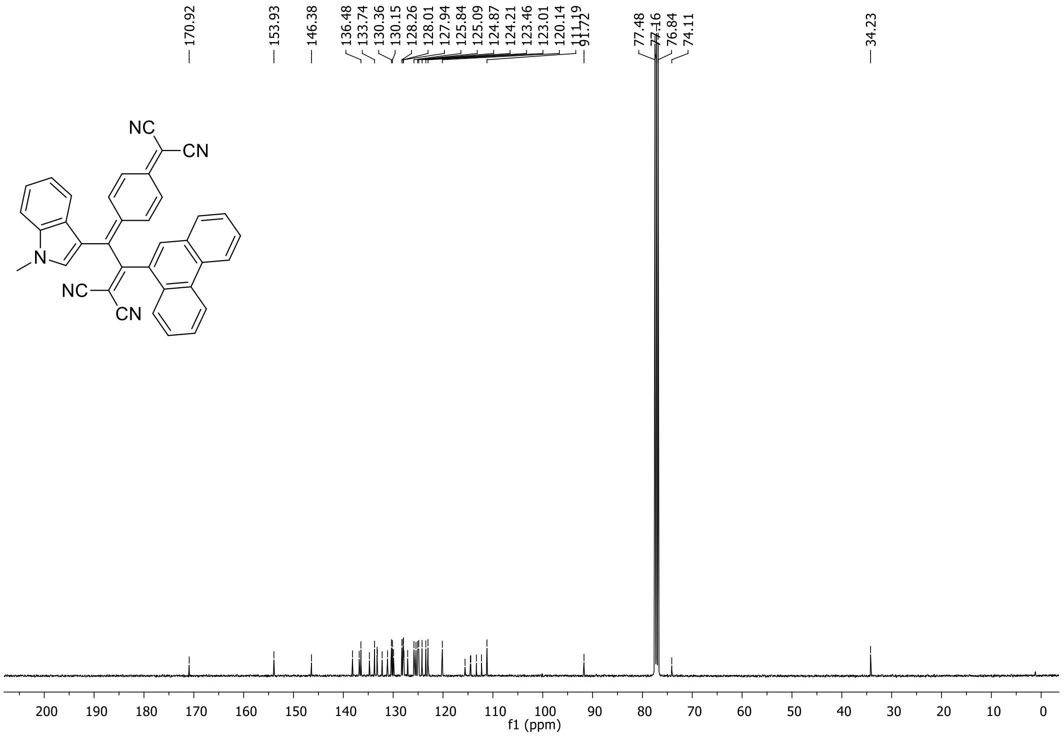

Figure S53. $^{13}C\{^{1}H\}$ NMR spectrum of **7h** in $CDCl_3$ solution (100 MHz).

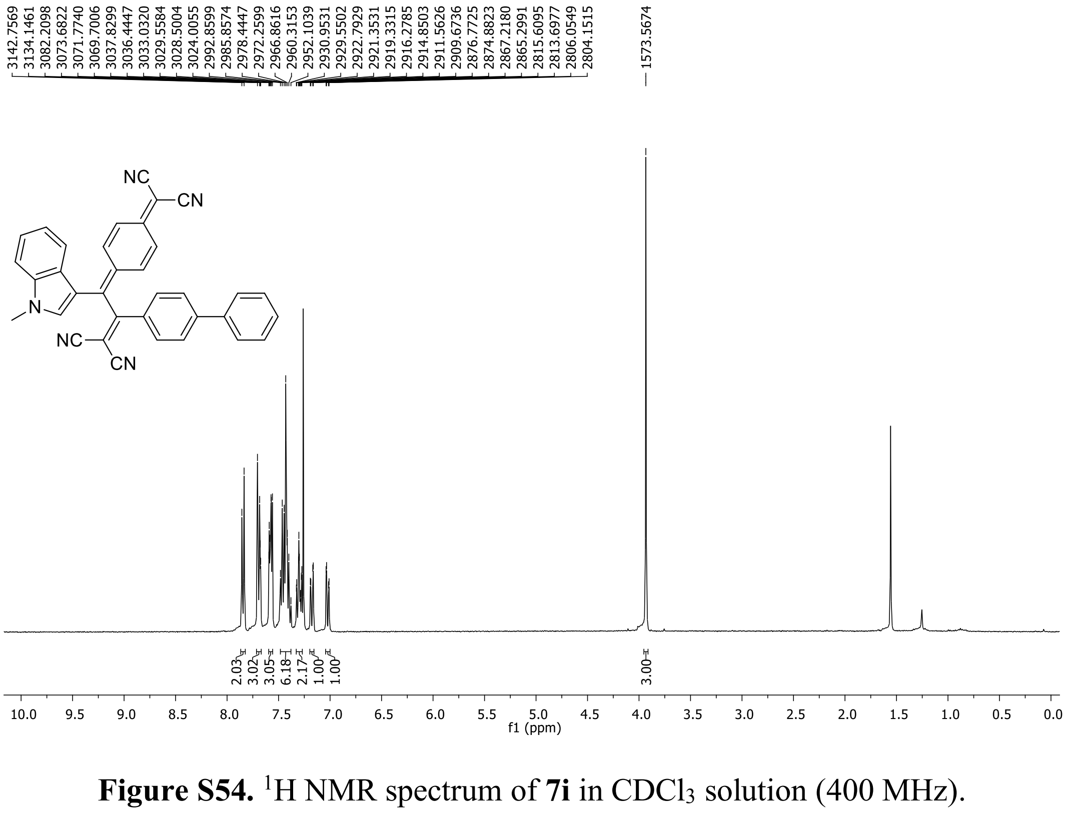

Figure S54. ^{1}H NMR spectrum of **7i** in $CDCl_3$ solution (400 MHz).

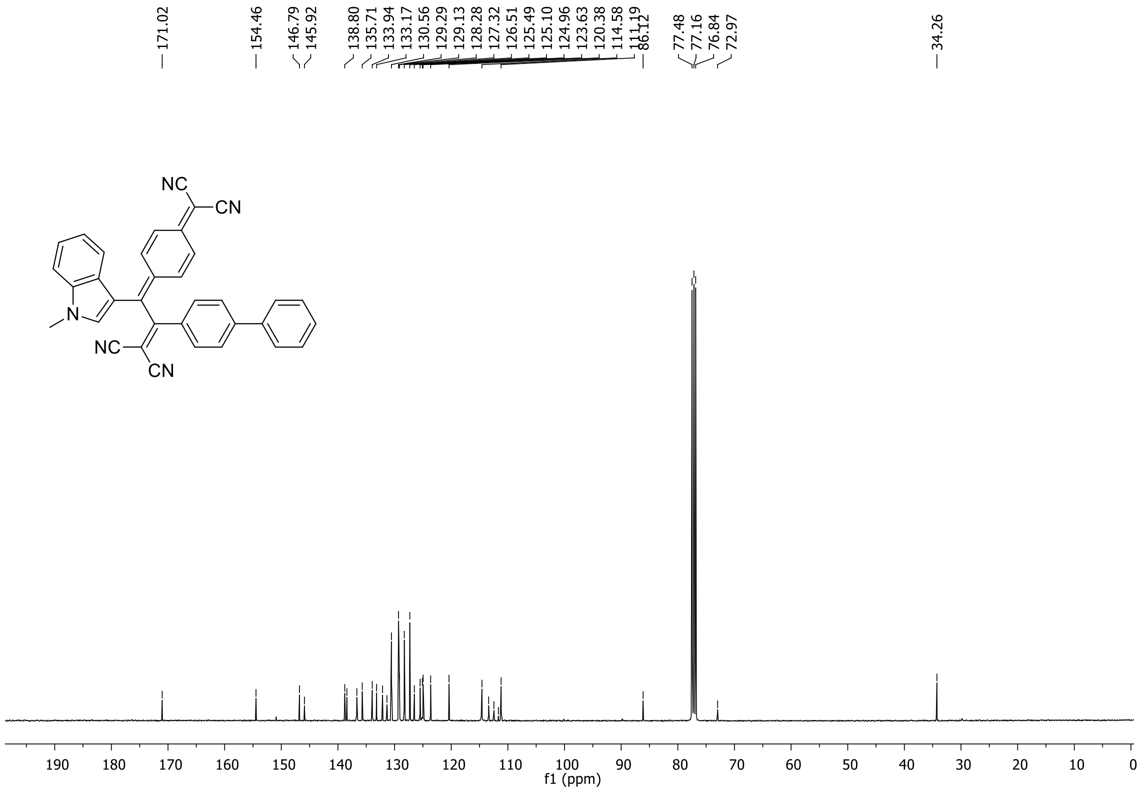

Figure S55. $^{13}C\{^1H\}$ NMR spectrum of **7i** in $CDCl_3$ solution (100 MHz).

3. High-Resolution Mass Spectrometry (HR-MS) Data

Elemental Composition Report **Page 1**

Single Mass Analysis
Tolerance = 1000.0 PPM / DBE: min = -5.5, max = 1000.0
Element prediction: Off
Number of isotope peaks used for i-FIT = 3

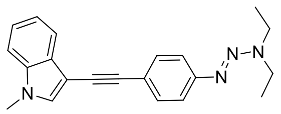

Monoisotopic Mass, Odd and Even Electron Ions
1 formula(e) evaluated with 1 results within limits (all results (up to 1000) for each mass)
Elements Used:
C: 21-21 H: 22-23 N: 4-4
Cagatay Dengiz
32149_20210326_01-03 2 (0.104) Cm (1:7)

1: TOF MS ES+
4.04e+004

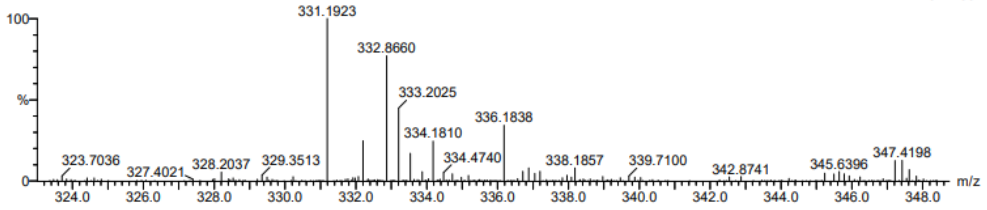

Minimum:				-5.5			
Maximum:		1000.0	1000.0	1000.0			
Mass	Calc. Mass	mDa	PPM	DBE	i-FIT	i-FIT (Norm)	Formula
331.1923	331.1923	0.0	0.0	12.5	334.6	0.0	C21 H23 N4

Figure S56. HR-MS spectrum of **3a**

Elemental Composition Report **Page 1**

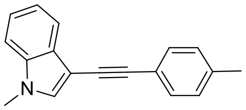

Single Mass Analysis
Tolerance = 1000.0 PPM / DBE: min = -5.5, max = 1000.0
Element prediction: Off
Number of isotope peaks used for i-FIT = 3

Monoisotopic Mass, Even Electron Ions
1 formula(e) evaluated with 1 results within limits (all results (up to 1000) for each mass)
Elements Used:
C: 18-18 H: 15-17 N: 1-1
Cagatay Dengiz
34145_20211105_06-04 20 (0.775) Cm (17:25)

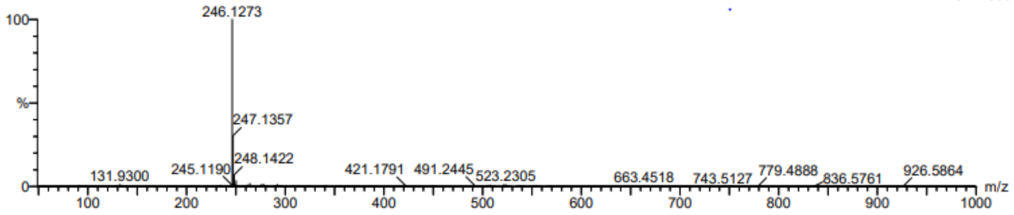

Minimum:				-5.5			
Maximum:		1000.0	1000.0	1000.0			
Mass	Calc. Mass	mDa	PPM	DBE	i-FIT	i-FIT (Norm)	Formula
246.1273	246.1283	-1.0	-4.1	11.5	748.9	0.0	C18 H16 N

Figure S57. HR-MS spectrum of **3d**

Elemental Composition Report **Page 1**

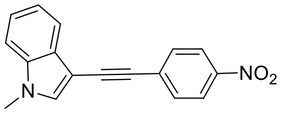

Single Mass Analysis
Tolerance = 1000.0 PPM / DBE: min = -5.5, max = 1000.0
Element prediction: Off
Number of isotope peaks used for i-FIT = 3

Monoisotopic Mass, Even Electron Ions
1 formula(e) evaluated with 1 results within limits (all results (up to 1000) for each mass)
Elements Used:
C: 17-17 H: 12-13 N: 2-2 O: 2-2
Cagatay Dengiz
34145_20211105_03-04 19 (0.741) Cm (11:25)

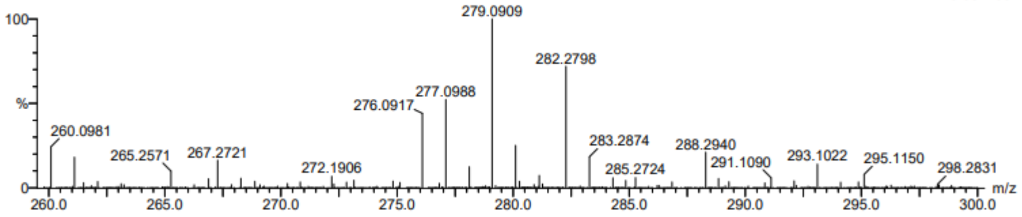

Minimum:				-5.5			
Maximum:		1000.0	1000.0	1000.0			
Mass	Calc. Mass	mDa	PPM	DBE	i-FIT	i-FIT (Norm)	Formula
277.0988	277.0977	1.1	4.0	12.5	464.8	0.0	C17 H13 N2 O2

Figure S58. HR-MS spectrum of **3e**

Elemental Composition Report

Page 1

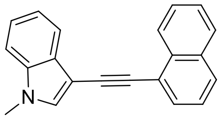

Single Mass Analysis
Tolerance = 1000.0 PPM / DBE: min = -5.5, max = 1000.0
Element prediction: Off
Number of isotope peaks used for i-FIT = 3

Monoisotopic Mass, Odd and Even Electron Ions
1 formula(e) evaluated with 1 results within limits (all results (up to 1000) for each mass)
Elements Used:
C: 21-21 H: 15-16 N: 1-1
Cagatay Dengiz
32149_20210326_09-03 5 (0.206) Cm (1:13)

1: TOF MS ES+
9.33e+005

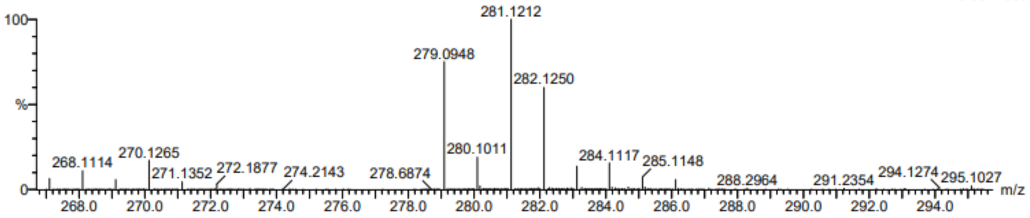

Minimum:				-5.5			
Maximum:		1000.0	1000.0	1000.0			
Mass	Calc. Mass	mDa	PPM	DBE	i-FIT	i-FIT (Norm)	Formula
281.1212	281.1204	0.8	2.8	15.0	788.4	0.0	C21 H15 N

Figure S59. HR-MS spectrum of **3f**

Elemental Composition Report

Page 1

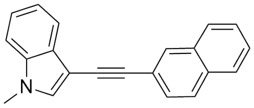

Single Mass Analysis
Tolerance = 1000.0 PPM / DBE: min = -5.5, max = 1000.0
Element prediction: Off
Number of isotope peaks used for i-FIT = 3

Monoisotopic Mass, Odd and Even Electron Ions
1 formula(e) evaluated with 1 results within limits (all results (up to 1000) for each mass)
Elements Used:
C: 21-21 H: 15-16 N: 1-1
Cagatay Dengiz
32149_20210326_12-03 7 (0.294) Cm (5:20)

1: TOF MS ES+
6.10e+005

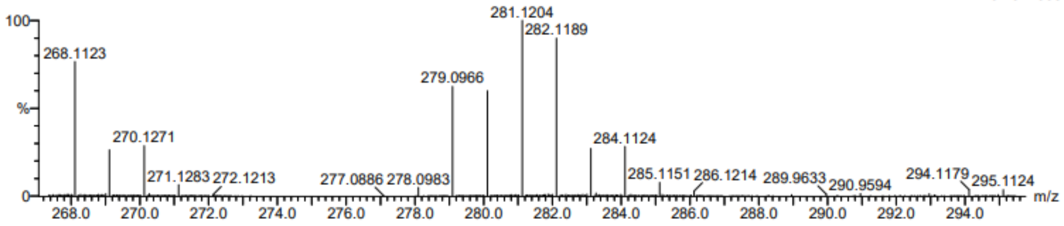

Minimum:				-5.5			
Maximum:		1000.0	1000.0	1000.0			
Mass	Calc. Mass	mDa	PPM	DBE	i-FIT	i-FIT (Norm)	Formula
281.1204	281.1204	0.0	0.0	15.0	782.1	0.0	C21 H15 N

Figure S60. HR-MS spectrum of **3g**

Elemental Composition Report **Page 1**

Single Mass Analysis
Tolerance = 1000.0 PPM / DBE: min = -5.5, max = 1000.0
Element prediction: Off
Number of isotope peaks used for i-FIT = 3

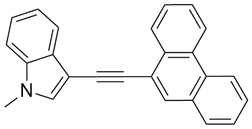

Monoisotopic Mass, Odd and Even Electron Ions
1 formula(e) evaluated with 1 results within limits (all results (up to 1000)) for each mass)
Elements Used:
C: 25-25 H: 17-18 N: 1-1
Cagatay Dengiz
32149_20210326_15-02 7 (0.294) Cm (1:10)
1: TOF MS ES+
2.70e+005

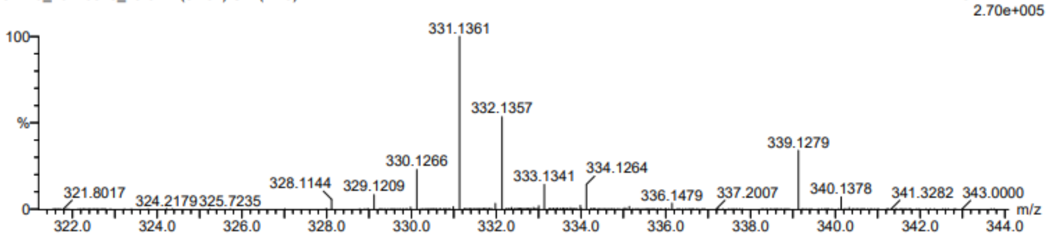

Minimum:				-5.5			
Maximum:		1000.0	1000.0	1000.0			
Mass	Calc. Mass	mDa	PPM	DBE	i-FIT	i-FIT (Norm)	Formula
331.1361	331.1361	0.0	0.0	18.0	649.0	0.0	C25 H17 N

Figure S61. HR-MS spectrum of **3h**

Elemental Composition Report **Page 1**

Single Mass Analysis
Tolerance = 1000.0 PPM / DBE: min = -5.5, max = 1000.0
Element prediction: Off
Number of isotope peaks used for i-FIT = 3

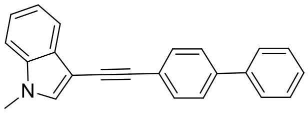

Monoisotopic Mass, Even Electron Ions
1 formula(e) evaluated with 1 results within limits (all results (up to 1000)) for each mass)
Elements Used:
C: 23-23 H: 17-18 N: 1-1
Cagatay Dengiz
34145_20211108_09-04 14 (0.552) Cm (1:16)
1: TOF MS ES+
7.41e+004

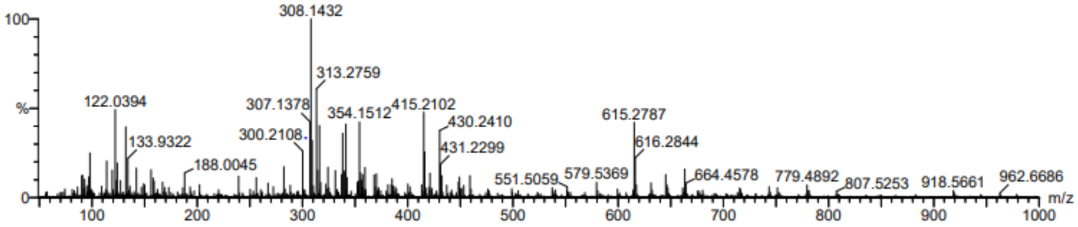

Minimum:				-5.5			
Maximum:		1000.0	1000.0	1000.0			
Mass	Calc. Mass	mDa	PPM	DBE	i-FIT	i-FIT (Norm)	Formula
308.1432	308.1439	-0.7	-2.3	15.5	454.6	0.0	C23 H18 N

Figure S62. HR-MS spectrum of **3i**

Elemental Composition Report

Page 1

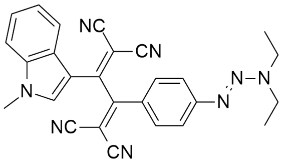

Single Mass Analysis

Tolerance = 1000.0 PPM / DBE: min = -5.5, max = 1000.0
Element prediction: Off
Number of isotope peaks used for i-FIT = 3

Monoisotopic Mass, Odd and Even Electron Ions
1 formula(e) evaluated with 1 results within limits (all results (up to 1000) for each mass)
Elements Used:
C: 27-27 H: 22-23 N: 8-8
Cagatay Dengiz
32149_20210326_02-06 21 (0.829) Cm (16:22)

1: TOF MS ES+
2.68e+005

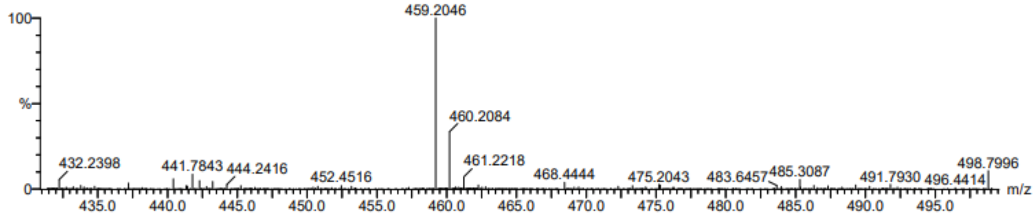

Minimum:				-5.5			
Maximum:		1000.0	1000.0	1000.0			
Mass	Calc. Mass	mDa	PPM	DBE	i-FIT	i-FIT (Norm)	Formula
459.2046	459.2046	0.0	0.0	20.5	515.5	0.0	C27 H23 N8

Figure S63. HR-MS spectrum of **5a**

Elemental Composition Report

Page 1

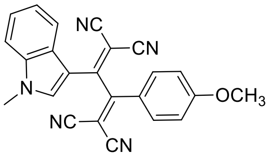

Single Mass Analysis

Tolerance = 1000.0 PPM / DBE: min = -5.5, max = 1000.0
Element prediction: Off
Number of isotope peaks used for i-FIT = 3

Monoisotopic Mass, Even Electron Ions
1 formula(e) evaluated with 1 results within limits (all results (up to 1000) for each mass)
Elements Used:
C: 24-24 H: 15-16 N: 5-5 O: 1-1
Cagatay Dengiz
34145_20211105_01-04 7 (0.294) Cm (1:25)

1: TOF MS ES+
3.04e+005

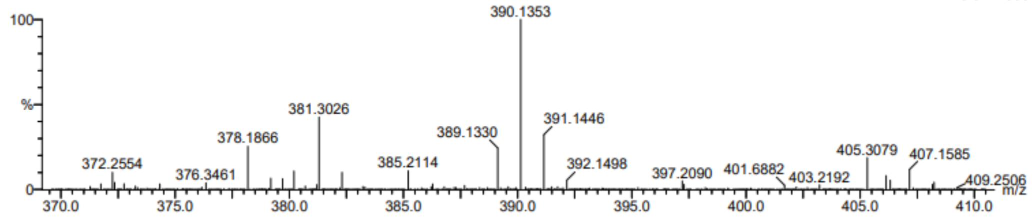

Minimum:				-5.5			
Maximum:		1000.0	1000.0	1000.0			
Mass	Calc. Mass	mDa	PPM	DBE	i-FIT	i-FIT (Norm)	Formula
390.1353	390.1355	-0.2	-0.5	19.5	456.5	0.0	C24 H16 N5 O

Figure S64. HR-MS spectrum of **5b**

Elemental Composition Report **Page 1**

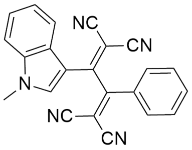

Single Mass Analysis
Tolerance = 1000.0 PPM / DBE: min = -5.5, max = 1000.0
Element prediction: Off
Number of isotope peaks used for i-FIT = 3

Monoisotopic Mass, Odd and Even Electron Ions
1 formula(e) evaluated with 1 results within limits (all results (up to 1000) for each mass)
Elements Used:
C: 23-23 H: 13-14 N: 5-5
Cagatay Dengiz
32149_20210326_04-02 3 (0.138) Cm (1:6)

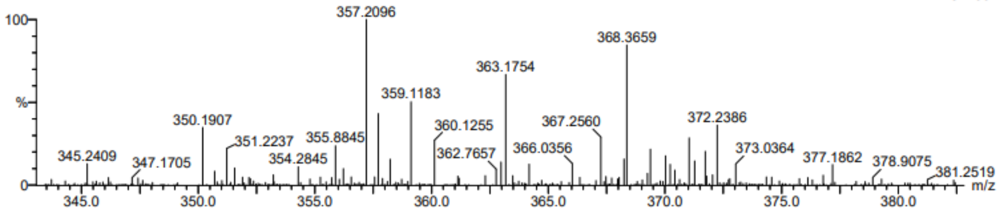

Minimum:				-5.5			
Maximum:		1000.0	1000.0	1000.0			
Mass	Calc. Mass	mDa	PPM	DBE	i-FIT	i-FIT (Norm)	Formula
359.1183	359.1171	1.2	3.3	20.0	155.4	0.0	C23 H13 N5

Figure S65. HR-MS spectrum of **5c**

Elemental Composition Report **Page 1**

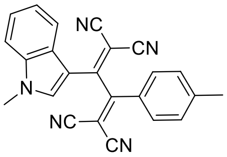

Single Mass Analysis
Tolerance = 1000.0 PPM / DBE: min = -5.5, max = 1000.0
Element prediction: Off
Number of isotope peaks used for i-FIT = 3

Monoisotopic Mass, Even Electron Ions
1 formula(e) evaluated with 1 results within limits (all results (up to 1000) for each mass)
Elements Used:
C: 24-24 H: 15-16 N: 5-5
Cagatay Dengiz
34145_20211108_07-03 14 (0.552) Cm (10:20)

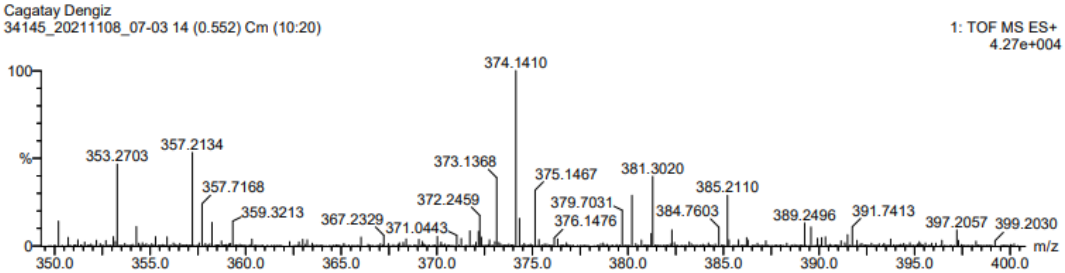

Minimum:				-5.5			
Maximum:		1000.0	1000.0	1000.0			
Mass	Calc. Mass	mDa	PPM	DBE	i-FIT	i-FIT (Norm)	Formula
374.1410	374.1406	0.4	1.1	19.5	321.2	0.0	C24 H16 N5

Figure S66. HR-MS spectrum of **5d**

Elemental Composition Report **Page 1**

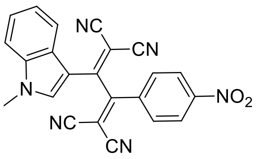

Single Mass Analysis
Tolerance = 1000.0 PPM / DBE: min = -5.5, max = 1000.0
Element prediction: Off
Number of isotope peaks used for i-FIT = 3

Monoisotopic Mass, Even Electron Ions
1 formula(e) evaluated with 1 results within limits (all results (up to 1000) for each mass)
Elements Used:
C: 23-23 H: 12-14 N: 6-6 O: 2-2
Cagatay Dengiz
34145_20211105_04-N03 6 (0.260) Cm (1:15)

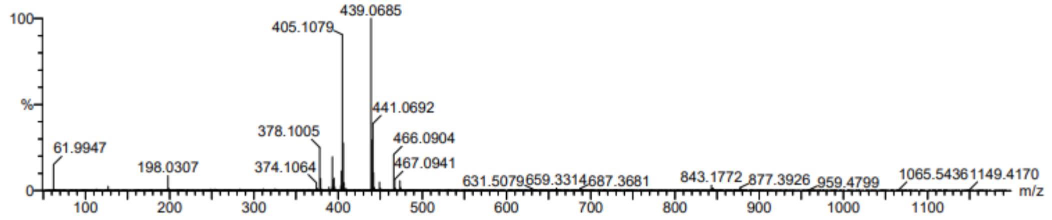

Minimum:				-5.5			
Maximum:		1000.0	1000.0	1000.0			
Mass	Calc. Mass	mDa	PPM	DBE	i-FIT	i-FIT (Norm)	Formula
405.1079	405.1100	-2.1	-5.2	20.5	303.0	0.0	C23 H13 N6 O2

Figure S67. HR-MS spectrum of **5e**

Elemental Composition Report **Page 1**

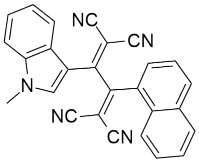

Single Mass Analysis
Tolerance = 1000.0 PPM / DBE: min = -5.5, max = 1000.0
Element prediction: Off
Number of isotope peaks used for i-FIT = 3

Monoisotopic Mass, Odd and Even Electron Ions
1 formula(e) evaluated with 1 results within limits (all results (up to 1000) for each mass)
Elements Used:
C: 27-27 H: 15-16 N: 5-5
Cagatay Dengiz
32149_20210326_10-03 8 (0.328) Cm (1:12)

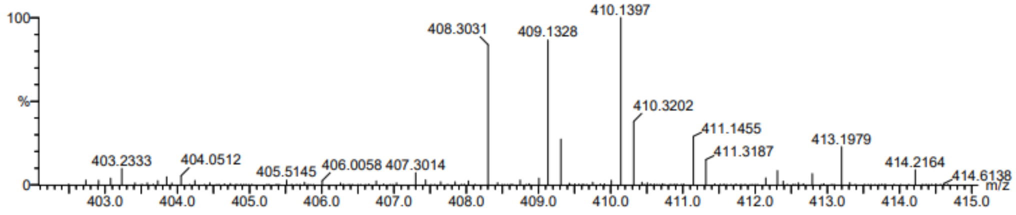

Minimum:				-5.5			
Maximum:		1000.0	1000.0	1000.0			
Mass	Calc. Mass	mDa	PPM	DBE	i-FIT	i-FIT (Norm)	Formula
409.1328	409.1327	0.1	0.2	23.0	344.7	0.0	C27 H15 N5

Figure S68. HR-MS spectrum of **5f**

Elemental Composition Report **Page 1**

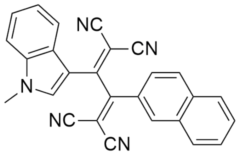

Single Mass Analysis
Tolerance = 1000.0 PPM / DBE: min = -5.5, max = 1000.0
Element prediction: Off
Number of isotope peaks used for i-FIT = 3

Monoisotopic Mass, Odd and Even Electron Ions
1 formula(e) evaluated with 1 results within limits (all results (up to 1000) for each mass)
Elements Used:
C: 27-27 H: 15-16 N: 5-5
Cagatay Dengiz
32149_20210326_13-02 4 (0.172) Cm (1:13)

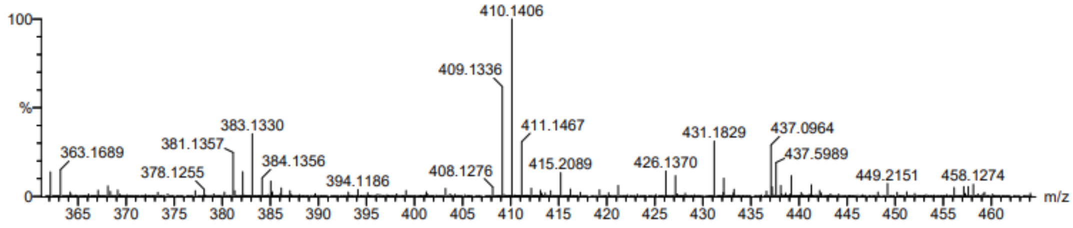

Minimum:				-5.5			
Maximum:		1000.0	1000.0	1000.0			
Mass	Calc. Mass	mDa	PPM	DBE	i-FIT	i-FIT (Norm)	Formula
410.1406	410.1406	0.0	0.0	22.5	469.3	0.0	C27 H16 N5

Figure S69. HR-MS spectrum of **5g**

Elemental Composition Report **Page 1**

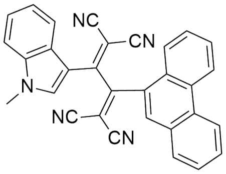

Single Mass Analysis
Tolerance = 1000.0 PPM / DBE: min = -5.5, max = 1000.0
Element prediction: Off
Number of isotope peaks used for i-FIT = 3

Monoisotopic Mass, Odd and Even Electron Ions
1 formula(e) evaluated with 1 results within limits (all results (up to 1000) for each mass)
Elements Used:
C: 31-31 H: 17-18 N: 5-5
Cagatay Dengiz
32149_20210326_16-03 25 (0.965) Cm (7:25)

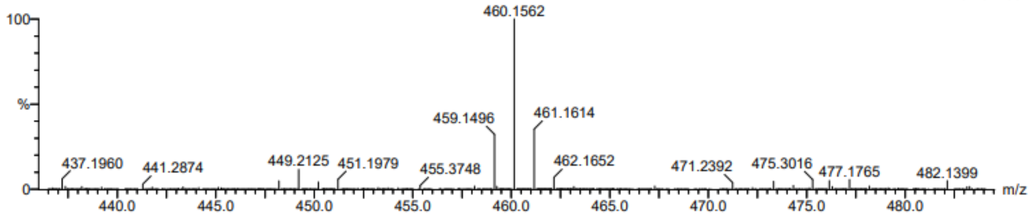

Minimum:				-5.5			
Maximum:		1000.0	1000.0	1000.0			
Mass	Calc. Mass	mDa	PPM	DBE	i-FIT	i-FIT (Norm)	Formula
460.1562	460.1562	0.0	0.0	25.5	479.0	0.0	C31 H18 N5

Figure S70. HR-MS spectrum of **5h**

Elemental Composition Report

Page 1

Single Mass Analysis
Tolerance = 1000.0 PPM / DBE: min = -5.5, max = 1000.0
Element prediction: Off
Number of isotope peaks used for i-FIT = 3

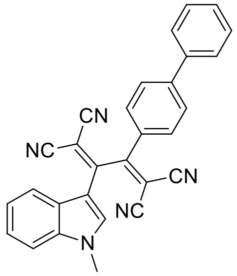

Monoisotopic Mass, Even Electron Ions
1 formula(e) evaluated with 1 results within limits (all results (up to 1000) for each mass)
Elements Used:
C: 29-29 H: 17-18 N: 5-5
Cagatay Dengiz
34145_20211108_10-07 2 (0.104) Cm (2:16)

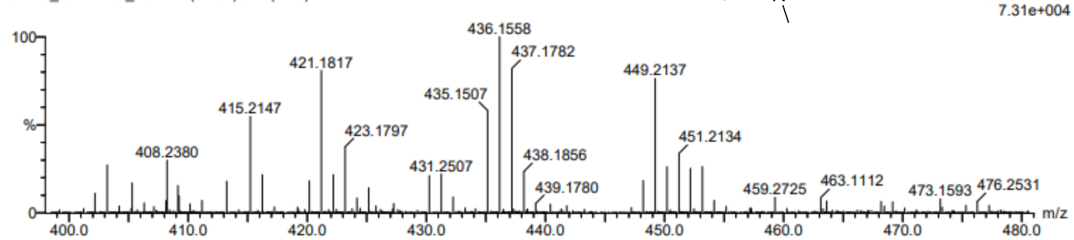

Minimum:				-5.5			
Maximum:		1000.0	1000.0	1000.0			
Mass	Calc. Mass	mDa	PPM	DBE	i-FIT	i-FIT (Norm)	Formula
436.1558	436.1562	-0.4	-0.9	23.5	387.1	0.0	C29 H18 N5

Figure S71. HR-MS spectrum of **5i**

Elemental Composition Report

Page 1

Single Mass Analysis
Tolerance = 1000.0 PPM / DBE: min = -5.5, max = 1000.0
Element prediction: Off
Number of isotope peaks used for i-FIT = 3

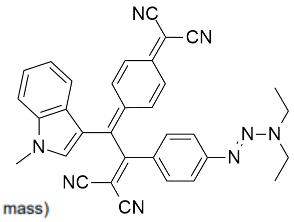

Monoisotopic Mass, Odd and Even Electron Ions
1 formula(e) evaluated with 1 results within limits (all results (up to 1000) for each mass)
Elements Used:
C: 33-33 H: 26-27 N: 8-8
Cagatay Dengiz
32149_20210326_03-04 25 (0.965) Cm (17:25)

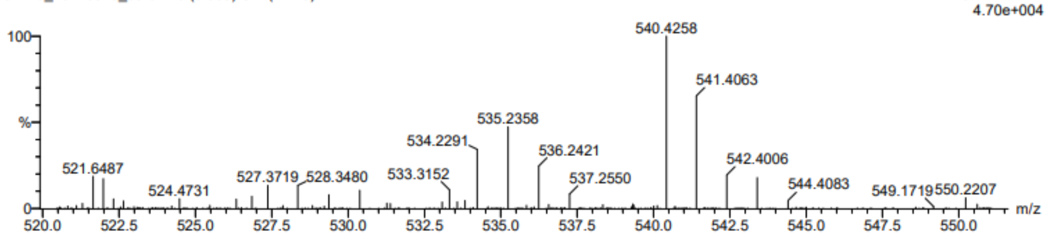

Minimum:				-5.5			
Maximum:		1000.0	1000.0	1000.0			
Mass	Calc. Mass	mDa	PPM	DBE	i-FIT	i-FIT (Norm)	Formula
535.2358	535.2359	-0.1	-0.2	24.5	243.2	0.0	C33 H27 N8

Figure S72. HR-MS spectrum of **7a**

Elemental Composition Report

Page 1

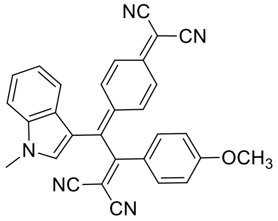

Single Mass Analysis

Tolerance = 1000.0 PPM / DBE: min = -5.5, max = 1000.0
Element prediction: Off
Number of isotope peaks used for i-FIT = 3

Monoisotopic Mass, Even Electron Ions
1 formula(e) evaluated with 1 results within limits (all results (up to 1000)) for each mass)
Elements Used:
C: 30-30 H: 19-20 N: 5-5 O: 1-1
Cagatay Dengiz
34145_20211105_02-03 12 (0.484) Cm (1:25)

1: TOF MS ES+
1.05e+005

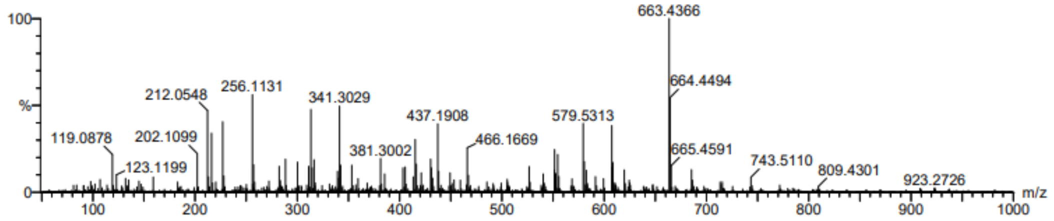

Minimum:				-5.5			
Maximum:		1000.0	1000.0	1000.0			

Mass	Calc. Mass	mDa	PPM	DBE	i-FIT	i-FIT (Norm)	Formula
466.1669	466.1668	0.1	0.2	23.5	307.1	0.0	C30 H20 N5 O

Figure S73. HR-MS spectrum of **7b**

Elemental Composition Report

Page 1

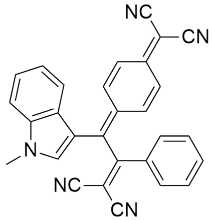

Single Mass Analysis

Tolerance = 1000.0 PPM / DBE: min = -5.5, max = 1000.0
Element prediction: Off
Number of isotope peaks used for i-FIT = 3

Monoisotopic Mass, Even Electron Ions
1 formula(e) evaluated with 1 results within limits (all results (up to 1000)) for each mass)
Elements Used:
C: 29-29 H: 17-18 N: 5-5
Cagatay Dengiz
32149_20210326_05-02 16 (0.639) Cm (1:16)

1: TOF MS ES+
1.34e+004

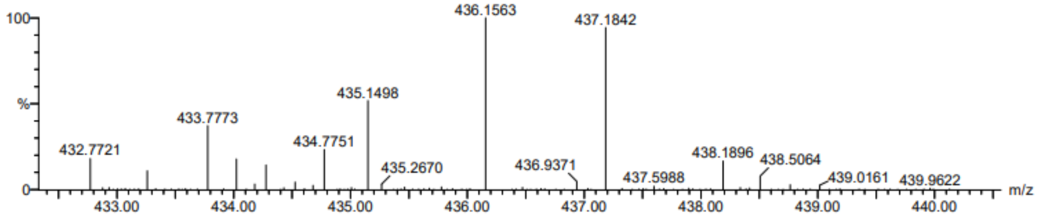

Minimum:				-5.5			
Maximum:		1000.0	1000.0	1000.0			

Mass	Calc. Mass	mDa	PPM	DBE	i-FIT	i-FIT (Norm)	Formula
436.1563	436.1562	0.1	0.2	23.5	244.6	0.0	C29 H18 N5

Figure S74. HR-MS spectrum of **7c**

Elemental Composition Report Page 1

Single Mass Analysis
Tolerance = 1000.0 PPM / DBE: min = -5.5, max = 1000.0
Element prediction: Off
Number of isotope peaks used for i-FIT = 3

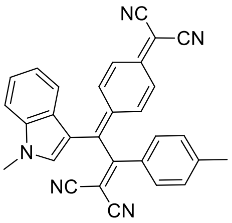

Monoisotopic Mass, Even Electron Ions
1 formula(e) evaluated with 1 results within limits (all results (up to 1000) for each mass)
Elements Used:
C: 30-30 H: 19-20 N: 5-5
Cagatay Dengiz
34145_20211108_08-02 10 (0.396) Cm (1:12)

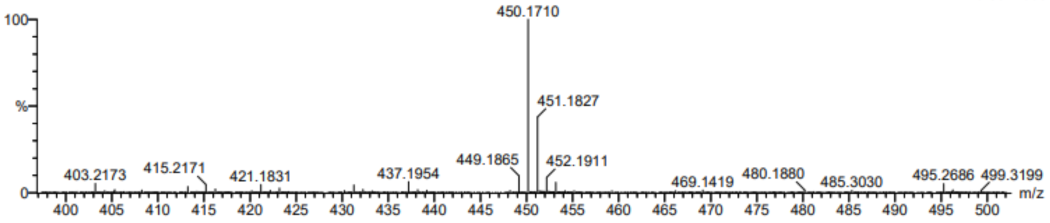

Minimum:				-5.5			
Maximum:		1000.0	1000.0	1000.0			
Mass	Calc. Mass	mDa	PPM	DBE	i-FIT	i-FIT (Norm)	Formula
450.1710	450.1719	-0.9	-2.0	23.5	505.8	0.0	C30 H20 N5

Figure S75. HR-MS spectrum of **7d**

Elemental Composition Report Page 1

Single Mass Analysis
Tolerance = 1000.0 PPM / DBE: min = -5.5, max = 1000.0
Element prediction: Off
Number of isotope peaks used for i-FIT = 3

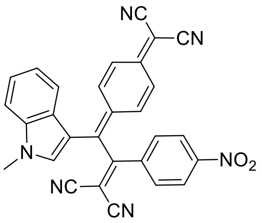

Monoisotopic Mass, Even Electron Ions
1 formula(e) evaluated with 1 results within limits (all results (up to 1000) for each mass)
Elements Used:
C: 29-29 H: 16-17 N: 6-6 O: 2-2
Cagatay Dengiz
34145_20211105_05-03 19 (0.741) Cm (10:20)

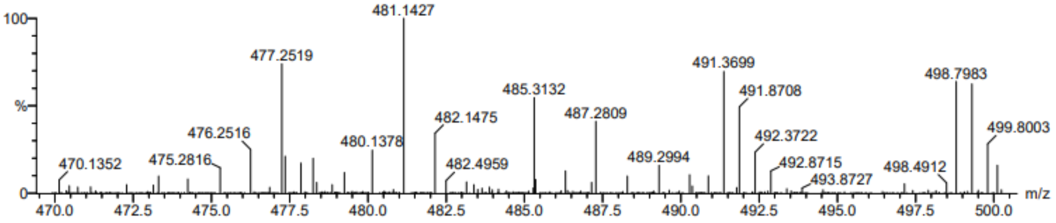

Minimum:				-5.5			
Maximum:		1000.0	1000.0	1000.0			
Mass	Calc. Mass	mDa	PPM	DBE	i-FIT	i-FIT (Norm)	Formula
481.1427	481.1413	1.4	2.9	24.5	171.3	0.0	C29 H17 N6 O2

Figure S76. HR-MS spectrum of **7e**

Elemental Composition Report

Page 1

Single Mass Analysis
Tolerance = 1000.0 PPM / DBE: min = -5.5, max = 1000.0
Element prediction: Off
Number of isotope peaks used for i-FIT = 3

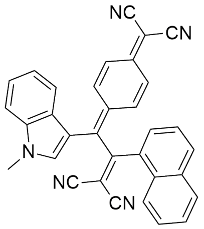

Monoisotopic Mass, Odd and Even Electron Ions
1 formula(e) evaluated with 1 results within limits (all results (up to 1000) for each mass)
Elements Used:
C: 33-33 H: 19-20 N: 5-5
Cagatay Dengiz
32149_20210326_11-02 4 (0.172) Cm (1:12)

1: TOF MS ES+
1.60e+005

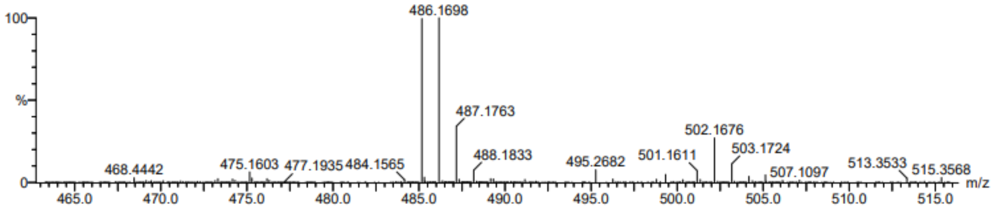

Minimum:				-5.5			
Maximum:		1000.0	1000.0	1000.0			
Mass	Calc. Mass	mDa	PPM	DBE	i-FIT	i-FIT (Norm)	Formula
485.1639	485.1640	-0.1	-0.2	27.0	460.1	0.0	C33 H19 N5

Figure S77. HR-MS spectrum of **7f**

Elemental Composition Report

Page 1

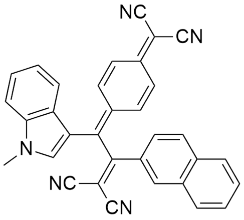

Single Mass Analysis
Tolerance = 1000.0 PPM / DBE: min = -5.5, max = 1000.0
Element prediction: Off
Number of isotope peaks used for i-FIT = 3

Monoisotopic Mass, Even Electron Ions
1 formula(e) evaluated with 1 results within limits (all results (up to 1000) for each mass)
Elements Used:
C: 33-33 H: 19-20 N: 5-5
Cagatay Dengiz
32149_20210326_14-03 4 (0.172) Cm (3:17)

1: TOF MS ES+
3.09e+004

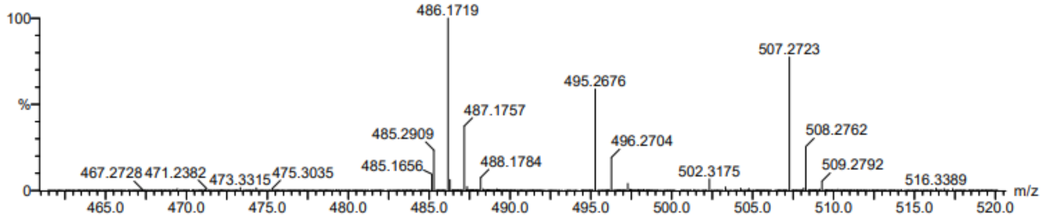

Minimum:				-5.5			
Maximum:		1000.0	1000.0	1000.0			
Mass	Calc. Mass	mDa	PPM	DBE	i-FIT	i-FIT (Norm)	Formula
486.1719	486.1719	0.0	0.0	26.5	368.1	0.0	C33 H20 N5

Figure S78. HR-MS spectrum of **7g**

Elemental Composition Report | **Page 1**

Single Mass Analysis
Tolerance = 1000.0 PPM / DBE: min = -5.5, max = 1000.0
Element prediction: Off
Number of isotope peaks used for i-FIT = 3

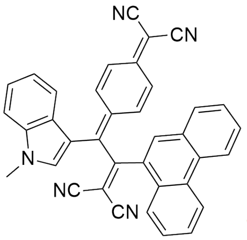

Monoisotopic Mass, Odd and Even Electron Ions
1 formula(e) evaluated with 1 results within limits (all results (up to 1000) for each mass)
Elements Used:
C: 37-37 H: 21-22 N: 5-5
Cagatay Dengiz
32149_20210326_17-01 21 (0.829) Cm (1:25)

1: TOF MS ES+
2.26e+005

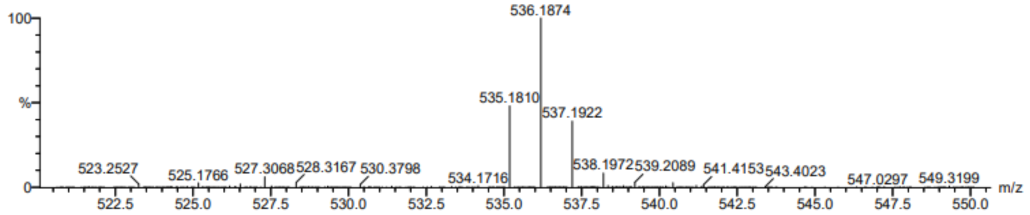

Minimum:				-5.5			
Maximum:		1000.0	1000.0	1000.0			
Mass	Calc. Mass	mDa	PPM	DBE	i-FIT	i-FIT (Norm)	Formula
536.1874	536.1875	-0.1	-0.2	29.5	457.7	0.0	C37 H22 N5

Figure S79. HR-MS spectrum of **7h**

Elemental Composition Report | **Page 1**

Single Mass Analysis
Tolerance = 1000.0 PPM / DBE: min = -5.5, max = 1000.0
Element prediction: Off
Number of isotope peaks used for i-FIT = 3

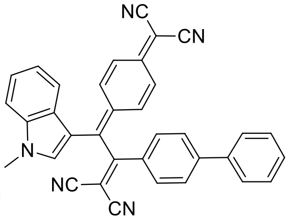

Monoisotopic Mass, Even Electron Ions
1 formula(e) evaluated with 1 results within limits (all results (up to 1000) for each mass)
Elements Used:
C: 35-35 H: 21-22 N: 5-5
Cagatay Dengiz
34145_20211108_11-04 14 (0.552) Cm (13:24)

1: TOF MS ES+
2.31e+004

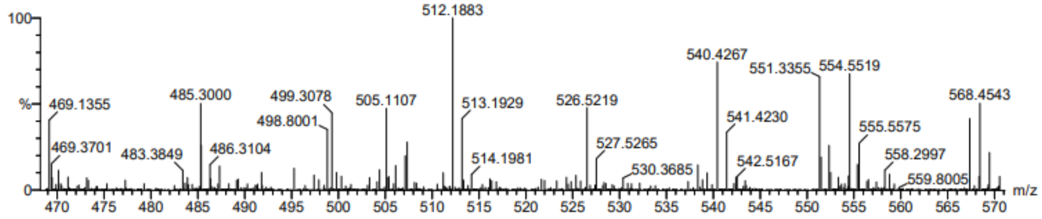

Minimum:				-5.5			
Maximum:		1000.0	1000.0	1000.0			
Mass	Calc. Mass	mDa	PPM	DBE	i-FIT	i-FIT (Norm)	Formula
512.1883	512.1875	0.8	1.6	27.5	217.6	0.0	C35 H22 N5

Figure S80. HR-MS spectrum of **7i**

4. UV/Vis Spectra

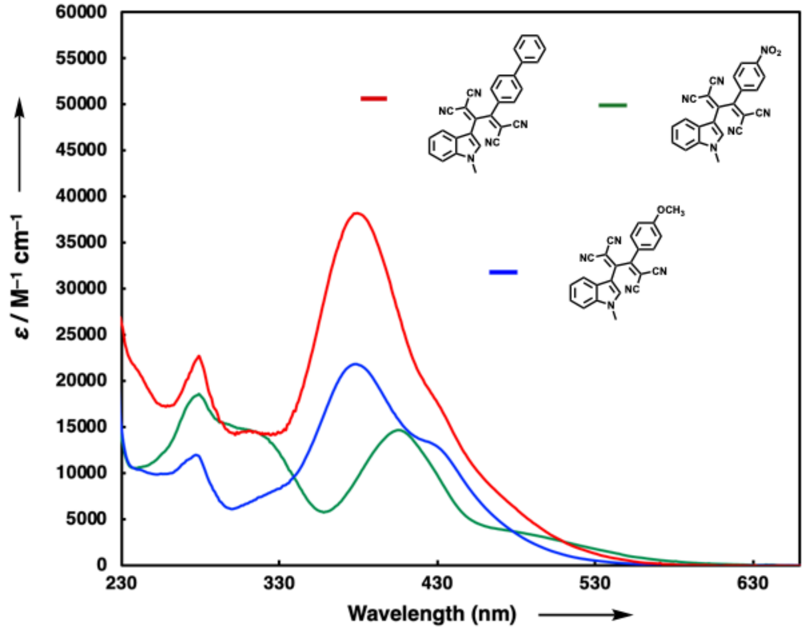

Figure S81. UV/Vis spectra (CH_2Cl_2, 25 ºC) of chromophores **5b**, **5e**, and **5i**.

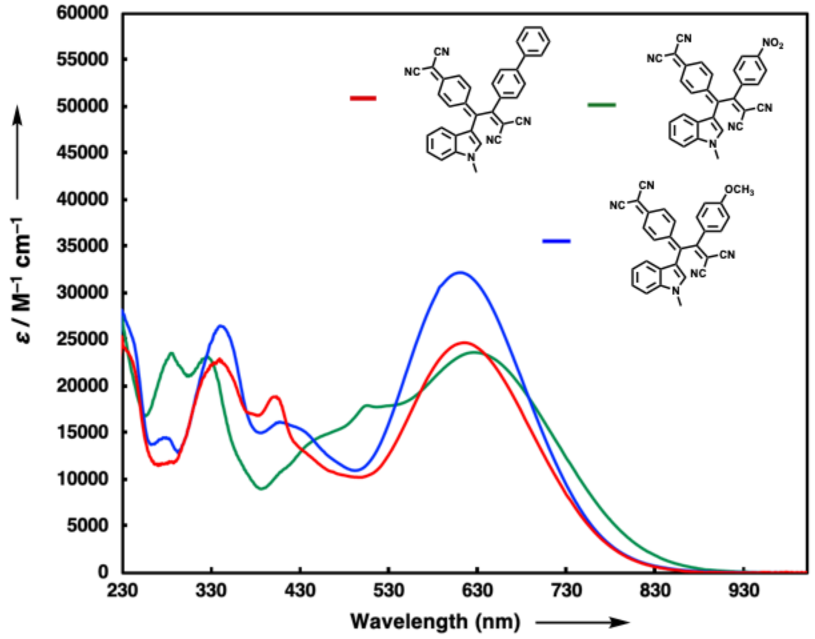

Figure S82. UV/Vis spectra (CH_2Cl_2, 25 ºC) of chromophores **7b**, **7e**, and **7i**.

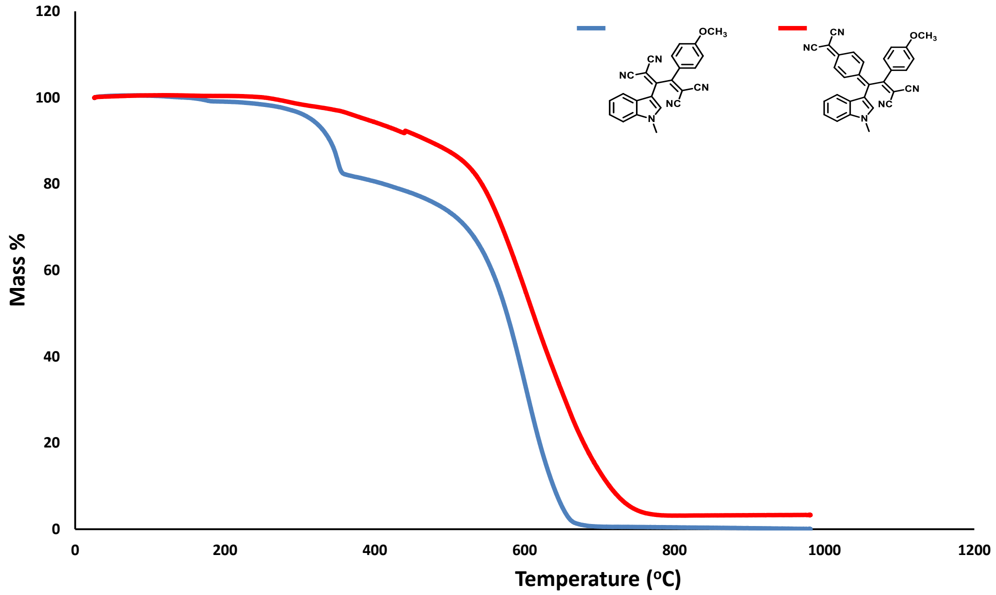

Figure S83. Thermogravimetric analysis (TGA) curves of chromophores **5b** and **7b**.
